# Global, regional, and national burden of hyperglycemia-associated colorectal cancer, 1990-2021: a systemic analysis for the Global Burden of Disease study

**DOI:** 10.3389/fonc.2025.1633508

**Published:** 2025-09-25

**Authors:** Yijuan Liu, Kaiyan Wei, Ye Xu, Dan Li

**Affiliations:** ^1^ Department of Gastroenterology, Fujian Medical University Union Hospital, Fuzhou, China; ^2^ The First Clinical College of Medicine, Fujian Medical University, Fuzhou, China; ^3^ Fujian Clinical Research Center for Digestive System Tumors and Upper Gastrointestinal Diseases, Department of Gastroenterology, Fujian Medical University Union Hospital, Fuzhou, China; ^4^ Department of Gastroenterology, The First Affiliated Hospital of Fujian Medical University, Fuzhou, China

**Keywords:** colorectal cancer, hyperglycemia, GBD, 2021, health inequality

## Abstract

**Background:**

Colorectal cancer (CRC) is a leading gastrointestinal malignancy with rising incidence. Hyperglycemia emerges as a critical driver of CRC progression, yet its global disease burden, particularly regarding health inequality and years lived with disability (YLDs), remains underexplored.

**Methods:**

This study leverages the Global Burden of Disease (GBD) 2021 dataset to analyze hyperglycemia-associated CRC burden across 21 regions and 204 countries from 1990 to 2021. It incorporates deaths, disability-adjusted life years (DALYs), YLDs, and years of life lost (YLLs), employing hierarchical clustering and health inequality metrics like the slope index of inequality and concentration index. Projections for 2022–2040 are generated using age-period-cohort models.

**Results:**

Globally, age-standardized rates (ASRs) of deaths, YLDs, YLLs, and DALYs attributed to hyperglycemia-associated CRC showed upward trends from 1990 to 2021, with YLDs exhibiting the highest consistent increase (EAPC = 1.47, 95% UI:1.35–1.60). Significant health disparities persisted across the 30 years, with higher burdens concentrated in high-socio-demographic index (SDI) regions. Notably, while mortality-related burdens slightly decreased in high-SDI areas, YLDs continued to rise, indicating unmitigated disability burdens. Projections suggest stable death rates but increasing YLDs, DALYs, and YLLs through 2040.

**Conclusion:**

Hyperglycemia-associated CRC imposes an escalating global burden, marked by persistent health inequalities and rising long-term disabilities. Urgent strategies to enhance glycemic control, expand CRC screening, and address cross-national disparities are imperative to alleviate this burden.

## 1 Introduction

Colorectal cancer (CRC) is a common malignant tumor in the gastrointestinal tract, the early diagnosis rate is low (1, 2). In recent years, the diagnosis of CRC has increased significantly due to advances in diagnostic techniques and cancer screening (3). According to the World Health Organization, there were 1,926,425 new cases with CRC and 904,019 new deaths associated with CRC in 2022. By 2035, the global incidence of CRC is expected to increase to 2.5 million new cases (4). The prognosis for treatment of patients with early-stage CRC is significantly better than that of patients with intermediate and late-stage disease (5). Therefore, early detection, screening and treatment can effectively reduce the burden of CRC and decrease the mortality rate of CRC.

While CRC arises from a complex interplay of genetic and environmental factors, emerging evidence highlights metabolic dysregulation—particularly hyperglycemia—as a critical driver of CRC progression. Traditional risk factors such as hereditary syndromes and lifestyle behaviors (e.g., smoking, alcohol consumption) contribute to CRC pathogenesis (6, 7). However, the escalating global prevalence of type 2 diabetes mellitus (T2DM) has drawn attention to hyperglycemia as a modifiable metabolic risk factor. Clinical studies indicate that CRC patients with T2DM exhibit an 18% reduction in 5-year survival compared to non-diabetic counterparts (8), while mechanistic research reveals that hyperglycemia promotes tumor proliferation via BMP4 and PI3K/AKT/mTOR pathways (9, 10). These findings underscore the need to quantify the disease burden specifically attributable to hyperglycemia, independent of other confounders.

Previous study have indicated a notable increase in CRC deaths and disease burden linked to metabolic risks (11, 12), particularly hyperglycemia (12), and highlight the significance of targeted cancer screening for high risk populations and the growing impact of hyperglycemia on CRC burden. However, these studies primarily focused on mortality and DALYs, with less emphasis on other dimensions of disease burden such as years lived with disability (YLDs) and years of life lost (YLLs). Furthermore, they did not extensively explore health inequality issues related to hyperglycemia-associated CRC across different countries.

To bridge these gaps, the present study provides a more nuanced analysis of the hyperglycemia-related CRC burden. Using a similar dataset, this study goes further in its analysis by incorporating not only deaths and DALYs but also YLDs and YLLs, offering a multi-faceted view of the disease’s impact. Additionally, the study introduces a novel perspective on global health inequality regarding hyperglycemia-associated CRC. Importantly, the study also employs hierarchical clustering to reveal variation in disease burden across different regions and countries, providing further evidence of the disparities that exist.

## 2 Methods

### 2.1 Data sources

The primary data source for this study was the Global Burden of Disease (GBD) 2021, which collected data from censuses, household surveys, civil registration and vital statistics, disease registries, health service use, satellite imaging, and disease notifications, and used Bayesian meta-regression modeling tools to model and estimate the burden under several scenarios for each country and region from 1990 to 2021 (13–16). Incidence rates per 100,000 population were age-standardized based on the GBD world population, and 95% uncertainty intervals (95% UI) were reported for estimation, including measurement error, potential bias, and sources of modeling-induced uncertainty (17).

### 2.2 Statistical analysis

The analysis encompassed 21 GBD regions and 204 countries/territories, with stratification by sex, age group, and Socio-demographic Index (SDI) region. SDI, a composite metric reflecting fertility, education, and per capita income, was used to classify countries and regions into five development levels (18). Age-standardized rates (ASRs) of CRC deaths, YLDs, YLLs, and DALYs were analyzed to assess disease burden dynamics. Subgroup analyses were conducted across the defined stratification dimensions.

Hierarchical clustering was applied to visualize regional variations in the burden of hyperglycemia-associated CRC, grouping the 21 GBD regions into four clusters based on their alignment with the expected trend curve. To assess temporal trends in CRC deaths and DALYs attributable to hyperglycemia from 1990 to 2021, estimated annual percentage change (EAPC) was calculated, with 95% confidence intervals (CIs) accounting for measurement errors, bias, and modeling uncertainty. A positive EAPC indicates an increasing trend, a negative EAPC indicates a decreasing trend, and a 95% CI that includes zero suggests no significant change, indicating a stable trend.

Pearson’s correlation coefficient was used to evaluate the association between EAPC, ASR, and SDI, providing insights into the relationship between disease burden and socio-demographic development. Health inequality analysis was performed to identify disparities in hyperglycemia-associated CRC burden across SDI levels, using the slope index of inequality and the concentration index as metrics.

Projections of hyperglycemia-associated CRC burden from 2022 to 2040 were conducted using the Nordpred package in R, which applies age-period-cohort models to forecast disease trends. All statistical analyses were performed using R software (version 4.4.2), with data visualization conducted using the ggplot2 package. Statistical significance was defined as a p-value < 0.05.

## 3 Results

### 3.1 Overall trend for hyperglycemia-associated CRC burden globally

**Figure 1:**
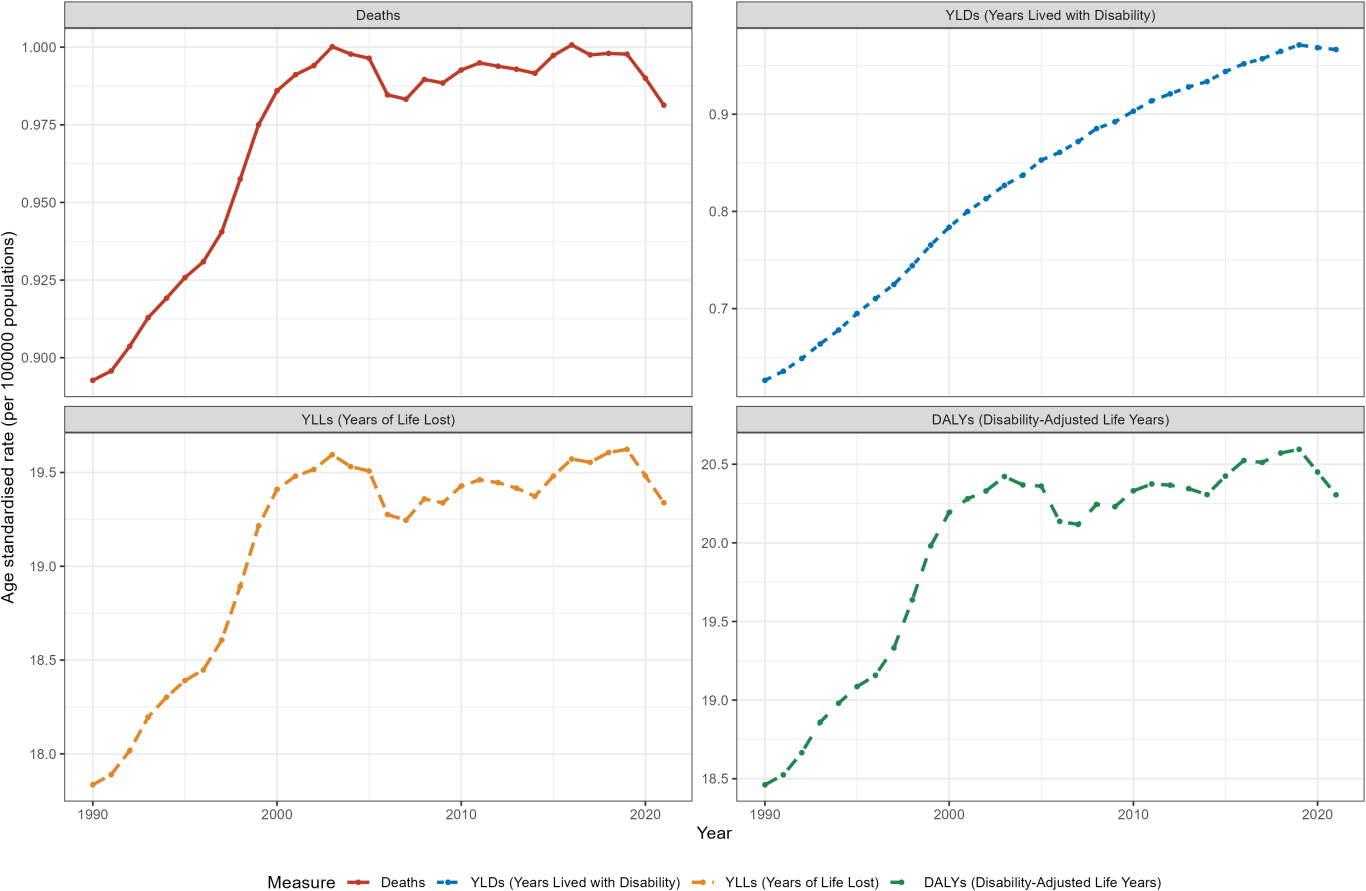
The trend of hyperglycemia-associated colorectal cancer-related ASRs of deaths, YLDs, YLLs and DALYs between 1990 and 2021.

Over the past 32 years, the global ASR of deaths attributable to hyperglycemia -associated CRC increased from 0.893 (95% UI: 0.451-1.342)in 1990 to 0.981 (95% UI: 0.505-1.492) in 2021, with an EAPC of 0.32 (95% UI:0.23-0.40). The ASRs of YLLs and DALYs also increased from 1990 to 2021, displaying a similar trend as that of deaths (**Table 1**). Meanwhile, the ASR of YLDs rose from 0.626 (95% UI: 0.297-1.342) to 0.967 (95% UI: 0.457-1.571). Notably, The ASR curves of deaths, YLLs and DALYs fluctuated in recent years, after reaching its peak in 2002 (**Figure 1**). While the ASR curves of YLDs exhibited a continuous rise, revealing a steady upward trend (EAPC = 1.47, 95% UI:1.35-1.60).

**Table 1:** The EAPC of hyperglycemia-associated colorectal cancer-related ASRs of deaths, YLDs, YLLs and DALYs between 1990 and 2021.

Location	Measure	Sex	Cause	Age	EAPC (95% CI)
Global	Deaths	Both	Colon and rectum cancer	Age-standardized	0.32 (0.23,0.4)
Global	DALYs	Both	Colon and rectum cancer	Age-standardized	0.31 (0.24,0.38)
Global	YLDs	Both	Colon and rectum cancer	Age-standardized	1.47 (1.35,1.6)
Global	YLLs	Both	Colon and rectum cancer	Age-standardized	0.26 (0.19,0.33)

EAPC, estimated annual percentage change; ASR, age-standardized rate; YLDs, Years Lived with Disability; YLLs, Years of Life Lost; DALYs, disability-adjusted-life-years.

In terms of gender, the ASRs of deaths, YLLs and DALYs were consistently higher for males than females during the past decades (**Supplementary Figure S1**, **Supplementary Table S1**). When stratified by age, the number of deaths, YLDs, YLLs, and DALYs in 2021 first increased and then decreased with age, presenting a nearly synchronous pattern. The highest number of both deaths and YLDs were observed in the 70–74 age group, with the least burdensome in the 25–29 age group (**Supplementary Figure S2**, **Supplementary Table S2**). At the SDI regional level, the ASRs of deaths, YLDs, YLLs and DALYs were higher in the high SDI region than that in the Lower SDI region. All of them displayed a slowly increasing trend in the Low SDI, Low-middle SDI, as well as the High-middle SDI region (**Supplementary Figure S3**, **Supplementary Table S3**). In contrast, the ASR of deaths showed an increase first and eventually a decline in high SDI region, with an EAPC of -0.08 (95% UI:-0.18-0.02). The ASR of YLLs, and DALYs presented a similar pattern while that of YLDs showed an upward trend (EAPC = 1.12, 95% UI: 0.93-1.31).

### 3.2 The temporal trends of hyperglycemia-associated CRC burden by GBD region from 1990 to 2021

**Figure 2:**
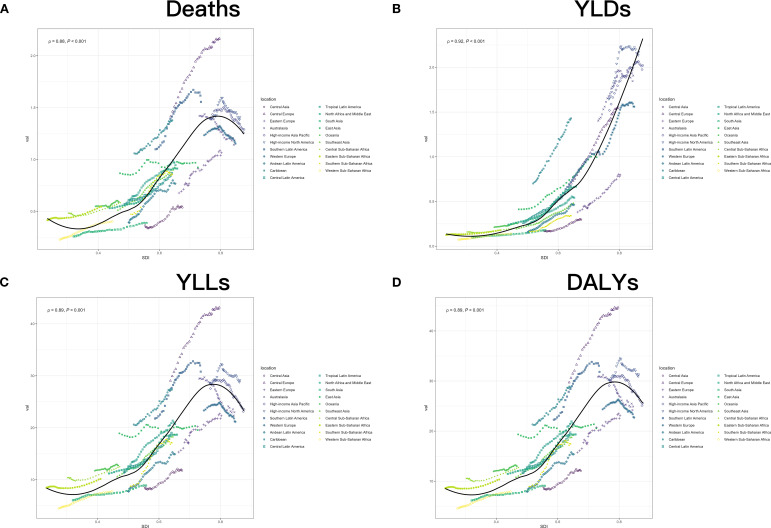
The trend of hyperglycemia-associated colorectal cancer-related ASRs of deaths **(A)**, YLDs **(B)**, YLLs **(C)** and DALYs **(D)** for different GBD regions between 1990 and 2021.

A stratified cluster analysis was conducted to better visualize the change in the burden of hyperglycemia-associated CRC from 1990 to 2021 in these GBD regions (**Figure 2**; **Supplementary Figure S4**, **Supplementary Table S4**). In terms of death, the North Africa and Middle East, Southern Sub-Saharan Africa, Southeast Asia and Central Latin America closely followed expected trends over the study period. The trend in high-income areas is in line with expectations but at a slightly higher level. Western Europe, Eastern Europe, Central Asia and Andean Latin America were all below expectations. Caribbean, Central Europe and Southern Latin America were all above expectations, and the rest of the region fluctuated. For the YLDs, most of the regions are trending in line with the predicted trend, with Central Latin America, North Africa, Tropic Latin America, Western Sub-Saharan Africa, South Aisa and Eastern Sub-Saharan Africa are almost in line with the forecast trend. Only Eastern Europe and Central Asia and Caribbean were well below or above expectations. Regarding the YLLs, Central Latin America, Southeast Asia, Southern Sub-Saharan Africa, and North Africa and Middle East nearly overlap with the expected line, and the rest of the region is almost evenly distributed above and below the expected curve. Similar to the YLLs, the DALYs results have the best fit for Central Latin America, Southeast Asia, Southern Sub-Saharan Africa and North Africa and Middle East. Southern Asia and Western Sub-Saharan have a tendency to be close to expectations but far from them. Caribbean and Central Europe are well above expected values and Central Asia is well below expectations. Overall, disease burden exhibits a significant positive correlation with SDI, peaking at an SDI of approximately 0.8. While other mortality-related indicators such as YLL, DALYs, and deaths are somewhat controlled in areas of extremely high SDI, YLDs, as a disability-related indicator, show no signs of improvement. This suggests that the disease-related disability burden remains uncontrolled in regions with very high SDI, regardless of the level of regional development.

### 3.3 The temporal trends of hyperglycemia-associated CRC burden by country from 1990 to 2021

**Figure 3:**
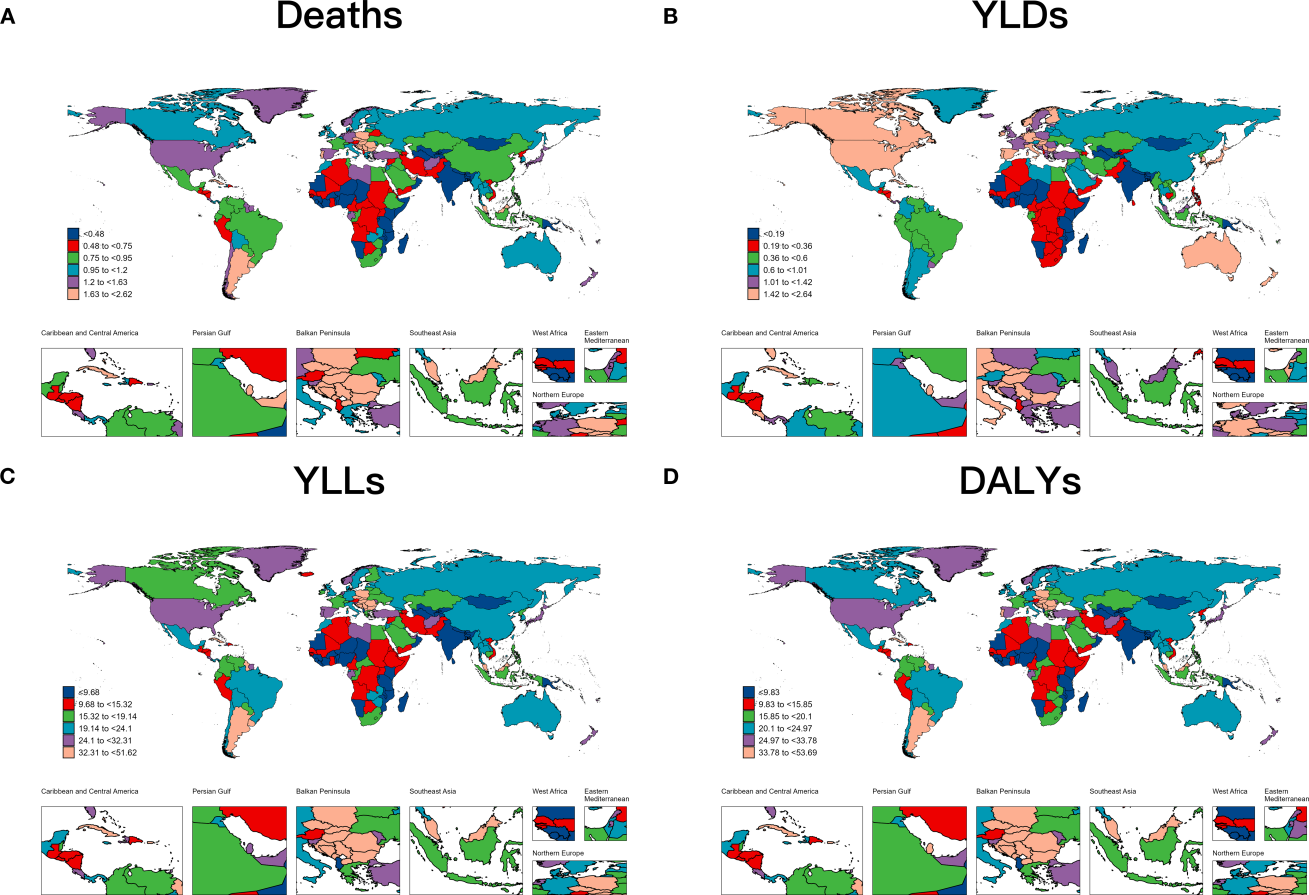
The trend of hyperglycemia-associated colorectal cancer-related ASRs of deaths **(A)**, YLDs **(B)**, YLLs **(C)** and DALYs **(D)** for different countries between 1990 and 2021.

Singapore had the largest decline in ASR of death with an EAPC value of -1.92 (95UI%: -2.17 to -1.76), while several countries with the largest increases included Egypt, Lesotho, Georgia, and Cabo Verde. Only one country, Ethiopia, showed a decreasing trend in YLDs, with the worst burden increases in Egypt, Lesotho and El Salvador. Singapore continues to have the largest decline in ASR for YLLs, along with Ethiopia, Maldives, and Germany; while the biggest climber was Lesotho; and the most significant increase was in the ASR of YLLs, followed by Egypt. Singapore, Ethiopia and Maldives continued to be the top three countries with the largest declines in ASR of DALYs, while Lesotho and Egypt held the top conditions (**Figure 3**, **Table 2**; **Supplementary Table S5**).

**Table 2:** The countries with the highest and lowest EAPC in deaths, YLDs, YLLs and DALYs between 1990 and 2021.

Location	Measure	Direction of trend	EAPC (95% CI)
Egypt	Deaths	largest increase	5.50 (5.08,5.93)
Tuvalu	Deaths	smallest increase	0.08 (0.03,0.13)
Singapore	Deaths	largest decline	-1.96 (-2.17,-1.7)
Czechia	Deaths	smallest decline	-0.30 (-0.54,-0.06)
Lesotho	YLLs	largest increase	5.59 (5.03,6.16)
Palau	YLLs	smallest increase	0.10 (0.04,0.17)
Singapore	YLLs	largest decline	-2.27 (-2.48,-2.06)
United Kingdom	YLLs	smallest decline	-0.17 (-0.25,-0.10)
Egypt	YLDs	largest increase	6.20 (5.87,6.52)
Nauru	YLDs	smallest increase	0.39 (0.19,0.59)
Ethiopia	YLDs	largest decline	-0.63 (-0.90,-0.35)
Ethiopia	YLDs	smallest decline	-0.63 (-0.90,-0.35)
Lesotho	DALYs	largest increase	5.59 (5.02,6.15)
Palau	DALYs	smallest increase	0.12 (0.06,0.18)
Singapore	DALYs	largest decline	-2.12 (-2.33,-1.91)
United Kingdom	DALYs	smallest decline	-0.08 (-0.15,0.005)

EAPC, estimated annual percentage change; ASR, age-standardized rate; YLDs, Years Lived with Disability; YLLs, Years of Life Lost; DALYs, disability-adjusted-life-years.

### 3.4 Cross-country inequalities in hyperglycemia-associated CRC from 1990 to 2021

**Figure 4:**
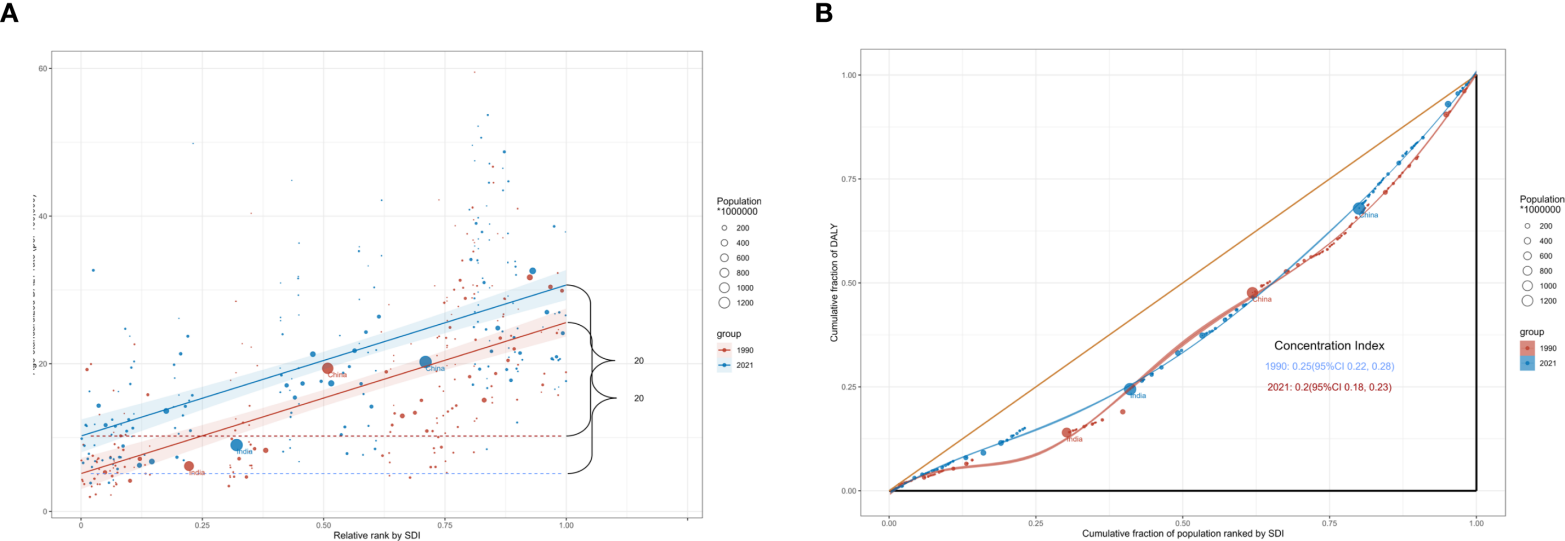
Health inequality regression curves **(A)** and concentration curves **(B)**.

The inequality slope index showed that the difference in the rate of DALYs between countries with the highest and lowest SDI was 20.46 (95% CI: 17.17 to 23.75) in 1990, and was virtually unchanged in 2021, with a difference of 20.45 (95% CI: 16.72 to 24.17). In addition, the concentration index was 0.248 (95% CI: 0.218 to 0.277) in 1990 and 0.205 (95% CI: 0.180 to 0.230) in 2021 (**Figure 4**). These findings indicate that from 1990 to 2021, the burden of CRC attributable to hyperglycemia remained concentrated in higher SDI countries, with no substantial reduction in inequality over time.

### 3.5 Prediction of disease burden for hyperglycemia-associated CRC from 2022 to 2040

**Figure 5:**
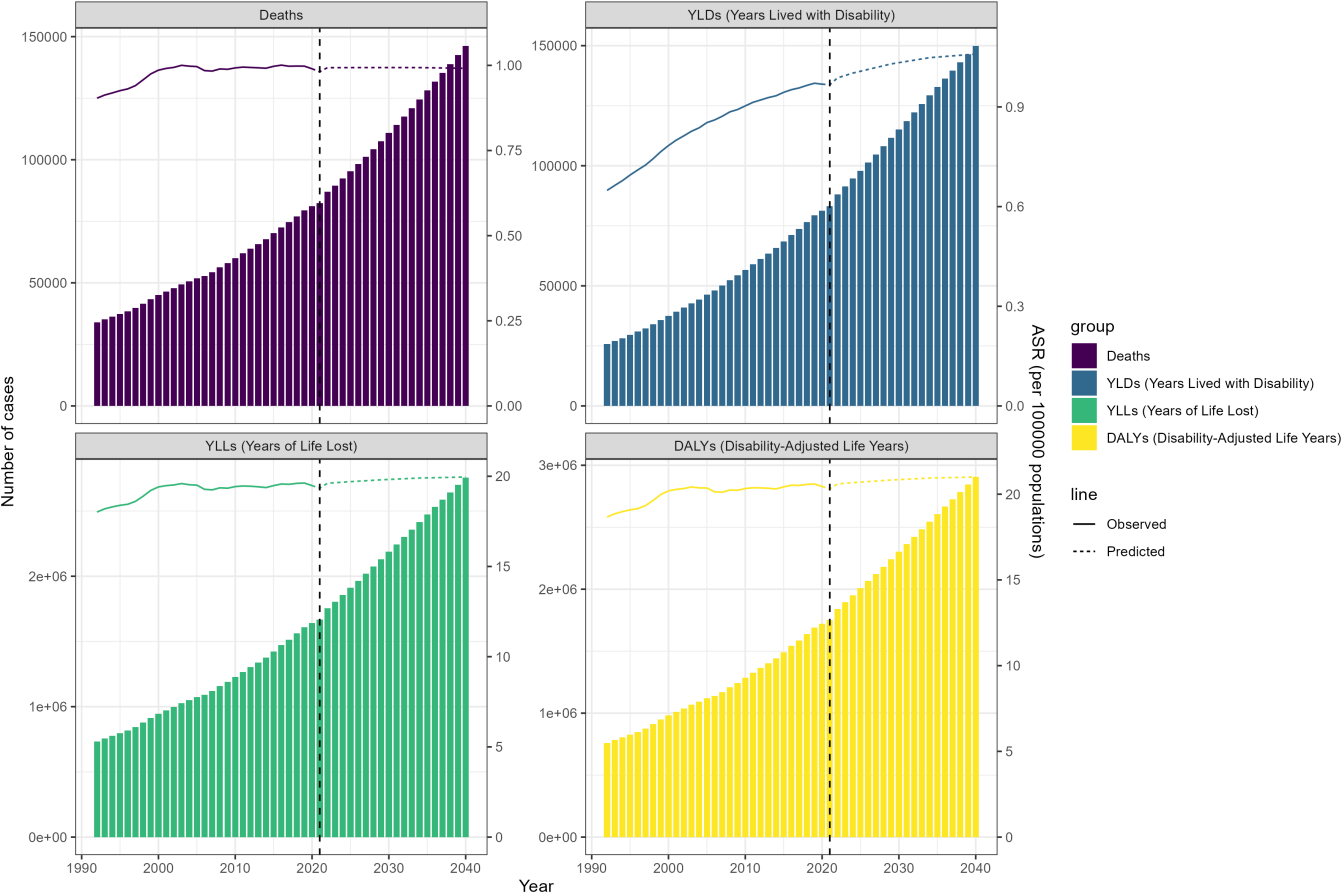
The predicted results in hyperglycemia-associated colorectal cancer-related ASRs of deaths, YLDs, YLLs and DALYs from 2022 to 2040.

According to the prediction model, from 2022 to 2040, the number of deaths due to hyperglycemia-associated CRC will gradually stabilize over the following decades. Concurrently, the number of YLLs and DALYs and YLDs showed an upward trend with a more pronounced increasing rate in that of YLDs (**Figure 5**). Notably, the ASRs of DALYs, YLLs, and YLDs show an upward trend, while the ASR of death does not.

## 4 Discussion

This study provides a systematic and comprehensive description of the global burden of hyperglycemia-associated CRC disease based on the GBD 2021 dataset, and gives the global burden of disease for different SDI levels, geographic regions, sexes, and age groups over a 30-year period from 1990 to 2021, with cross-national inequality analysis. The burden of hyperglycemia-associated CRC was also projected for the years 2022 to 2040.

Previous studies have revealed a notable increase in CRC deaths and disease burden associated with hyperglycemia (12), and underscored the significance of targeted cancer screening for high risk populations and the growing impact of hyperglycemia on CRC burden. Consistently, we found that the ASR of deaths, YLDs, YLLs, and DALYs attributed to hyperglycemia-associated CRC increased significantly globally between 1990 and 2021, with an overall upward trend despite fluctuating in recent years. Mechanically, hyperglycemia triggers counter-regulatory upregulation of insulin and insulin-like growth factor levels, which promotes neoplastic proliferation primarily through PI3K/AKT/mTOR signaling pathway (19). In addition, hyperglycemia can facilitate CRC tumorigenesis via the induction of inflammatory responses, oxidative stress, immunomodulation, and angiogenesis (20). Moreover, elevated blood glucose levels has been demonstrated to increase oxaliplatin chemo-resistance in CRC patients, potentially through the phosphorylation of SMAD3 and MYC and the upregulation of EHMT2 expression (21). Given the ongoing significance of hyperglycemia-associated CRC, these findings offer a mechanistic rationale for developing more effective clinical management strategies to address the public health concern.

In terms of gender differences, the results showed that the four ASRs were higher in male than in female, and that female showed flat or decreasing levels of ASRs compared to 1990, while the increase in male was still greater, suggesting that the male population is at higher risk for CRC. Evidence from population-based studies also suggests that hyperglycemia or diabetes is significantly associated with the risk of CRC in male. It has also been shown that alcohol consumption and smoking have a greater impact on CRC DALYs in men than in women (22–26). This gender difference may be related to differences in the tumor microenvironment, immune response levels and hormone levels, such as the expression of immune-related genomes in female patients is higher than that in males, and endogenous estrogen secretion in females has a protective effect on the development of CRC (27, 28). These results suggest that gender-regulated biological clocks differ significantly in males and females, and that in-depth studies of the mechanisms that contribute to the gender differences in disease burden are needed to provide gender-differentiated pathways for disease prevention, diagnosis, and treatment.

Similar to previous results, our age-stratified analysis of the burden of hyperglycemia-associated CRC in 2021 showed that the burden of disease increased significantly with age (29), with the highest number of death- and disability-adjusted years in the age group 65–74 years. This positive correlation of disease burden with age implies a cumulative effect of hyperglycemic risk exposure. These results illustrate the value of timely control of risk factors in effectively reducing the overall disease burden of CRC, especially in the elderly population.

This study also analyzed the burden of disease of hyperglycemia-associated CRC in different SDI regions. The burden of disease indicators (ASRs of deaths, YLDs, YLLs, and DALYs) had a positive association with SDI levels. The reason for this is hypothesized to be that high SDI regions have fast economic and medical development and more early detection of CRC through screening, cancer registries, and technological improvements, resulting in a higher number of diagnosed cases in High SDI regions, and a higher burden of disease at the time of the data count. However, the overall trend of ASR in these areas is slowly decreasing and the burden is decreasing due to early detection, intervention and treatment. Conversely, in contrast to the overall trend, there is a slow upward trend in disease ASR in Low-middle SDI and Low SDI regions. This may be due to horizontal developmental constraints, as these regions are experiencing a prevariant phase of disease burden in High SDI regions. To further analyze changes in burden trends across districts, we conducted a cluster analysis of burden across the 21 GBD districts. Our findings show that the countries with the highest number of hyperglycemia-associated CRC deaths and increased disease burden are Lesotho and Egypt, in large part because the burden of disease caused by diabetes or hyperglycemia is increasing significantly in these regions. According to a survey, Egypt ranks first in the MENA region in terms of the number of people with diabetes, which itself has the highest prevalence of diabetes in the world. Singapore, on the other hand, has the highest level of decline in ASR. This decline may be largely attributed to the widespread use of early screening programs such as fecal occult blood tests and colonoscopy. Screening programs aim to identify CRCs in the precancerous polyp stage and early malignant stage, which can help reduce CRC morbidity and mortality by removing precancerous lesions before malignant transformation or at an early stage (4, 30). In a meta-analysis of observational studies, screening colonoscopy was associated with a 70% reduction in CRC risk and 68% reduction in mortality. While the efficacy of screening colonoscopy in reducing CRC burden is clear, maximizing its benefit hinges on accurate and efficient histological assessment of detected lesions. Accumulating evidence suggests that deep learning algorithms, particularly convolutional neural networks, play a transformative role in the automated tissue classification and diagnosis of CRC histopathology images (31). These models demonstrate high accuracy in distinguishing between benign and malignant tissues, grading tumor malignancy, and identifying specific histological features related to prognosis, thus assisting pathologists by increasing diagnostic efficiency and reducing subjectivity (31, 32). Leveraging deep learning to automate CRC histopathological diagnosis represents a promising avenue for advancing cancer screening strategies. Overall, it is crucial to develop and implement CRC screening strategies and procedures, which are essential for early detection and improved prognostic survival (33).

To develop more effective treatment options for hyperglycemia-associated CRC, we projected the burden of disease from 2022-2040. According to the results, the ASR of YLDs for hyperglycemia-associated CRC will increase yearly, but other indicators are more stable. As the burden of diabetes is currently increasing and the impact of disability due to diabetes-related complications is growing, there is an urgent need for more complete strategies to mitigate the threat.

In the past 30 years, the health inequality index of hyperglycemia-related CRC has not improved. It implies that there has been a persistent neglect in addressing the uneven distribution of disease burden across different populations, which has contributed to the lack of progress in reducing these health disparities. Moreover, this study shows a split trend: while death-related burdens have lessened, disability burdens, measured by YLDs, are rising, even in high-SDI regions. This highlights the urgent need for targeted interventions. The increase in YLDs suggests more long-term disability from hyperglycemia-related CRC, possibly due to delayed diagnosis, suboptimal treatment, and the disease’s biological traits. Despite advanced medical resources in high-SDI areas, persistent disability burdens may stem from an aging population with higher hyperglycemia and CRC rates and challenges in managing chronic conditions. Current strategies may not effectively prevent or manage this disability, necessitating the development of better interventions to improve overall prognosis.

Different common preventive approaches have been suggested for both diabetes and CRC, focusing on modifiable lifestyle factors, including dietary interventions (e.g. lower dietary glycemic load and total carbohydrate intake) and the promotion of physical activity, as well as the hypoglycemic drugs (34). However, evidence of the effects of the hypoglycemic drugs, for instance, metformin, for chemoprevention remains controversial (35). Notably, a recent large-scale cohort study revealed a significant inverse association between optimal glycemic control (HbA1c <7%) and the risk of left-sided CRC, as well as both non-advanced and advanced colonic adenomas, in diabetic patients (36). Thus, incorporating glycemic management into preventive frameworks for CRC screening may represent a viable strategy for mitigating the associated disease burden. In clinical settings, managing CRC patients with hyperglycemia is often complicated and there are little agreed evidence based guidelines on the best management on this issue (37). The co-existence of both conditions poses a challenge to determining an appropriate glycemic target and the optimal combination therapy due to heterogeneity in patient status and drug-drug reactions. Multidisciplinary collaboration among surgeons, medical oncologists, and endocrinologists is essential for developing effective patient management strategies.

Beyond the imperative for integrated management of hyperglycemia and CRC, other pivotal issues, influencing the long term quality of life and functional outcomes of CRC patients, need to be addressed (38). Specifically, mental health disorders, particularly depression and anxiety, are increasingly recognized as significant predictors of adverse postoperative outcomes in CRC patients (39, 40). A growing body of evidence from observational studies suggests that pre-existing and post-diagnosis depression is associated with a higher risk of postoperative complications, including wound infections and paralytic ileus, potentially through dysregulation of the hypothalamic-pituitary-adrenal axis and impaired immune function (40–42). Therefore, systematic screening for psychological distress and integrating psychosocial interventions into standard perioperative care pathways are not merely supportive measures but crucial components for improving overall prognosis of CRC.

There are some limitations in this study. First, the data for GBD were obtained from cancer registries, among others, but the completeness of the data varies from country to country due to differences in the level of development. Second, due to the wide time frame covered by the study, changes in diagnostic and therapeutic approaches to CRC, and many social and economic factors, the incidence of the disease is more likely to be underestimated in its early stages, and this should be taken into account. Third, despite the finding of a significant increase in the burden of CRC associated with hyperglycemia, the analysis should be interpreted in conjunction with a comprehensive interpretation of multidimensional data to improve the accuracy and robustness of the predictions.

## 5 Conclusion

In summary, hyperglycemia-associated CRC poses a growing global burden, with YLDs rising even in high-SDI regions, indicating increasing long-term disability. Despite declining mortality, persistent health inequalities remain across SDI levels and countries. These findings underscore the urgent need for improved glycemic control, early CRC screening, and targeted interventions to reduce disability and address cross-national disparities in disease burden.

## Data Availability

Publicly available datasets were analyzed in this study. This data can be found here: https://vizhub.healthdata.org/gbd-results/.
