# Supplementary material for: Global, regional, and national burden of hyperglycemia-associated colorectal cancer, 1990-2021: a systemic analysis for the Global Burden of Disease study
Source: Front Oncol. 2025 Sep 25;15:1633508. doi: 10.3389/fonc.2025.1633508 (PMC12507591; doi:10.3389/fonc.2025.1633508)
Supplement: Supplementary file 1 [file DataSheet1.zip › Supplemental figure legends.docx]

**Figure legends**

**Figure S1.** The trend of hyperglycemia-associated colorectal cancer-related ASRs of deaths, YLDs, YLLs and DALYs for both sexes between 1990 and 2021. Abbreviations: EAPC, estimated annual percentage change; ASR, age-standardized rate; YLDs, Years Lived with Disability; YLLs, Years of Life Lost; DALYs, disability-adjusted-life-years.

**Figure S2.** The trend of hyperglycemia-associated colorectal cancer-related ASRs of deaths, YLDs, YLLs and DALYs for different age groups between 1990 and 2021. Abbreviations: EAPC, estimated annual percentage change; ASR, age-standardized rate; YLDs, Years Lived with Disability; YLLs, Years of Life Lost; DALYs, disability-adjusted-life-years.

**Figure S3.** The trend of hyperglycemia-associated colorectal cancer-related ASRs of deaths, YLDs, YLLs and DALYs for different SDI regions between 1990 and 2021. Abbreviations: EAPC, estimated annual percentage change; ASR, age-standardized rate; YLDs, Years Lived with Disability; YLLs, Years of Life Lost; DALYs, disability-adjusted-life-years.

**Figure S4.** Results of cluster analysis based on the EAPC values of hyperglycemia-associated colorectal cancer-related ASRs for deaths, YLDs, YLLs and DALYs from 1990 to 2021. Abbreviations: EAPC, estimated annual percentage change; ASR, age-standardized rate; YLDs, Years Lived with Disability; YLLs, Years of Life Lost; DALYs, disability-adjusted-life-years.
