# Supplementary material for: Global, regional, and national burden of hyperglycemia-associated colorectal cancer, 1990-2021: a systemic analysis for the Global Burden of Disease study
Source: Front Oncol. 2025 Sep 25;15:1633508. doi: 10.3389/fonc.2025.1633508 (PMC12507591; doi:10.3389/fonc.2025.1633508)
Supplement: Supplementary file 1 [file DataSheet1.zip › Table S1.docx]

**Table S1.** The EAPC of hyperglycemia-associated colorectal cancer-related ASRs of deaths, YLDs, YLLs and DALYs for both sexes between 1990 and 2021. Abbreviations: EAPC, estimated annual percentage change; ASR, age-standardized rate; YLDs, Years Lived with Disability; YLLs, Years of Life Lost; DALYs, disability-adjusted-life-years.

|  | **location** | **measure** | **sex** | **cause** | **age** | **EAPC** | **LCI** | **UCI** | **EAPC_95CI** |
| --- | --- | --- | --- | --- | --- | --- | --- | --- | --- |
| 1 | Global | Deaths | Male | Colon and rectum cancer | Age-standardized | 0.552920415 | 0.458057164 | 0.647873247 | 0.55(0.46,0.65) |
| 2 | Global | Deaths | Female | Colon and rectum cancer | Age-standardized | -0.029320195 | -0.108107576 | 0.049529328 | -0.03(-0.11,0.05) |
| 3 | Global | DALYs | Male | Colon and rectum cancer | Age-standardized | 0.548085955 | 0.465360473 | 0.630879555 | 0.55(0.47,0.63) |
| 4 | Global | DALYs | Female | Colon and rectum cancer | Age-standardized | -0.057454351 | -0.12209152 | 0.007224649 | -0.06(-0.12,0.01) |
| 5 | Global | YLDs | Male | Colon and rectum cancer | Age-standardized | 1.743552047 | 1.604985438 | 1.88230763 | 1.74(1.6,1.88) |
| 6 | Global | YLDs | Female | Colon and rectum cancer | Age-standardized | 1.047949247 | 0.935248722 | 1.16077561 | 1.05(0.94,1.16) |
| 7 | Global | YLLs | Male | Colon and rectum cancer | Age-standardized | 0.496492104 | 0.415077084 | 0.577973135 | 0.5(0.42,0.58) |
| 8 | Global | YLLs | Female | Colon and rectum cancer | Age-standardized | -0.103511684 | -0.167808636 | -0.039173323 | -0.1(-0.17,-0.04) |
