# Supplementary material for: Global, regional, and national burden of hyperglycemia-associated colorectal cancer, 1990-2021: a systemic analysis for the Global Burden of Disease study
Source: Front Oncol. 2025 Sep 25;15:1633508. doi: 10.3389/fonc.2025.1633508 (PMC12507591; doi:10.3389/fonc.2025.1633508)
Supplement: Supplementary file 1 [file DataSheet1.zip › Table S2.docx]

**Table S2.** The EAPC of hyperglycemia-associated colorectal cancer-related ASRs of deaths, YLDs, YLLs and DALYs for different age groups between 1990 and 2021. Abbreviations: EAPC, estimated annual percentage change; ASR, age-standardized rate; YLDs, Years Lived with Disability; YLLs, Years of Life Lost; DALYs, disability-adjusted-life-years.

| **measure** | **location** | **sex** | **age** | **cause** | **rei** | **metric** | **year** | **val** | **upper** | **lower** |
| --- | --- | --- | --- | --- | --- | --- | --- | --- | --- | --- |
| Deaths | Global | Male | 95+ years | Colon and rectum cancer | High fasting plasma glucose | Number | 1990 | 56.18005987 | 87.37502545 | 28.91340806 |
| Deaths | Global | Female | 95+ years | Colon and rectum cancer | High fasting plasma glucose | Number | 1990 | 142.0303772 | 225.3700977 | 70.28213269 |
| Deaths | Global | Both | 95+ years | Colon and rectum cancer | High fasting plasma glucose | Number | 1990 | 198.2104371 | 305.4607755 | 98.27626934 |
| Deaths | Global | Male | 95+ years | Colon and rectum cancer | High fasting plasma glucose | Rate | 1990 | 21.58851878 | 33.57592323 | 11.11066194 |
| Deaths | Global | Female | 95+ years | Colon and rectum cancer | High fasting plasma glucose | Rate | 1990 | 18.74109406 | 29.73787919 | 9.273819342 |
| Deaths | Global | Both | 95+ years | Colon and rectum cancer | High fasting plasma glucose | Rate | 1990 | 19.46891898 | 30.00342049 | 9.653037213 |
| Deaths | Global | Male | 95+ years | Colon and rectum cancer | High fasting plasma glucose | Number | 1991 | 60.02023023 | 93.4761073 | 30.61854988 |
| Deaths | Global | Female | 95+ years | Colon and rectum cancer | High fasting plasma glucose | Number | 1991 | 151.9892708 | 239.8896674 | 75.8313371 |
| Deaths | Global | Both | 95+ years | Colon and rectum cancer | High fasting plasma glucose | Number | 1991 | 212.0095011 | 328.3222844 | 105.0281909 |
| Deaths | Global | Male | 95+ years | Colon and rectum cancer | High fasting plasma glucose | Rate | 1991 | 22.13531546 | 34.47376184 | 11.292047 |
| Deaths | Global | Female | 95+ years | Colon and rectum cancer | High fasting plasma glucose | Rate | 1991 | 19.27339223 | 30.41982915 | 9.615988646 |
| Deaths | Global | Both | 95+ years | Colon and rectum cancer | High fasting plasma glucose | Rate | 1991 | 20.00565573 | 30.98117092 | 9.91067767 |
| Deaths | Global | Male | 95+ years | Colon and rectum cancer | High fasting plasma glucose | Number | 1992 | 63.74517157 | 99.32922493 | 32.62082093 |
| Deaths | Global | Female | 95+ years | Colon and rectum cancer | High fasting plasma glucose | Number | 1992 | 163.998195 | 257.8271535 | 80.95731123 |
| Deaths | Global | Both | 95+ years | Colon and rectum cancer | High fasting plasma glucose | Number | 1992 | 227.7433666 | 350.6653454 | 111.757079 |
| Deaths | Global | Male | 95+ years | Colon and rectum cancer | High fasting plasma glucose | Rate | 1992 | 22.45654934 | 34.99232311 | 11.49186765 |
| Deaths | Global | Female | 95+ years | Colon and rectum cancer | High fasting plasma glucose | Rate | 1992 | 19.89524195 | 31.27798815 | 9.821237936 |
| Deaths | Global | Both | 95+ years | Colon and rectum cancer | High fasting plasma glucose | Rate | 1992 | 20.55132694 | 31.64367976 | 10.08484376 |
| Deaths | Global | Male | 95+ years | Colon and rectum cancer | High fasting plasma glucose | Number | 1993 | 66.59446982 | 104.8490338 | 33.58846289 |
| Deaths | Global | Female | 95+ years | Colon and rectum cancer | High fasting plasma glucose | Number | 1993 | 176.6497838 | 279.3434917 | 87.37817832 |
| Deaths | Global | Both | 95+ years | Colon and rectum cancer | High fasting plasma glucose | Number | 1993 | 243.2442536 | 373.8449347 | 119.9566662 |
| Deaths | Global | Male | 95+ years | Colon and rectum cancer | High fasting plasma glucose | Rate | 1993 | 22.42201555 | 35.30213054 | 11.30906274 |
| Deaths | Global | Female | 95+ years | Colon and rectum cancer | High fasting plasma glucose | Rate | 1993 | 20.54430131 | 32.4875397 | 10.16204824 |
| Deaths | Global | Both | 95+ years | Colon and rectum cancer | High fasting plasma glucose | Rate | 1993 | 21.0263766 | 32.3156838 | 10.36922353 |
| Deaths | Global | Male | 95+ years | Colon and rectum cancer | High fasting plasma glucose | Number | 1994 | 70.84872228 | 111.4205408 | 35.9090458 |
| Deaths | Global | Female | 95+ years | Colon and rectum cancer | High fasting plasma glucose | Number | 1994 | 187.3910982 | 295.8102488 | 93.24743388 |
| Deaths | Global | Both | 95+ years | Colon and rectum cancer | High fasting plasma glucose | Number | 1994 | 258.2398205 | 400.0224294 | 128.0136905 |
| Deaths | Global | Male | 95+ years | Colon and rectum cancer | High fasting plasma glucose | Rate | 1994 | 22.75761683 | 35.78986176 | 11.53449602 |
| Deaths | Global | Female | 95+ years | Colon and rectum cancer | High fasting plasma glucose | Rate | 1994 | 20.82804106 | 32.87855221 | 10.36421367 |
| Deaths | Global | Both | 95+ years | Colon and rectum cancer | High fasting plasma glucose | Rate | 1994 | 21.32407813 | 33.03173586 | 10.57069329 |
| Deaths | Global | Male | 95+ years | Colon and rectum cancer | High fasting plasma glucose | Number | 1995 | 76.41111741 | 120.2533484 | 38.9318883 |
| Deaths | Global | Female | 95+ years | Colon and rectum cancer | High fasting plasma glucose | Number | 1995 | 202.0713694 | 318.2036754 | 100.4710281 |
| Deaths | Global | Both | 95+ years | Colon and rectum cancer | High fasting plasma glucose | Number | 1995 | 278.4824869 | 430.6531421 | 138.2572052 |
| Deaths | Global | Male | 95+ years | Colon and rectum cancer | High fasting plasma glucose | Rate | 1995 | 23.11657701 | 36.38012219 | 11.77802425 |
| Deaths | Global | Female | 95+ years | Colon and rectum cancer | High fasting plasma glucose | Rate | 1995 | 21.27707851 | 33.50521453 | 10.57908381 |
| Deaths | Global | Both | 95+ years | Colon and rectum cancer | High fasting plasma glucose | Rate | 1995 | 21.75201336 | 33.63792462 | 10.79914435 |
| Deaths | Global | Male | 95+ years | Colon and rectum cancer | High fasting plasma glucose | Number | 1996 | 81.93359633 | 127.9885935 | 41.91723175 |
| Deaths | Global | Female | 95+ years | Colon and rectum cancer | High fasting plasma glucose | Number | 1996 | 217.7786797 | 340.961668 | 106.6830778 |
| Deaths | Global | Both | 95+ years | Colon and rectum cancer | High fasting plasma glucose | Number | 1996 | 299.712276 | 458.5765311 | 148.6209929 |
| Deaths | Global | Male | 95+ years | Colon and rectum cancer | High fasting plasma glucose | Rate | 1996 | 23.08920609 | 36.06768341 | 11.81243892 |
| Deaths | Global | Female | 95+ years | Colon and rectum cancer | High fasting plasma glucose | Rate | 1996 | 21.47105287 | 33.6158067 | 10.5180085 |
| Deaths | Global | Both | 95+ years | Colon and rectum cancer | High fasting plasma glucose | Rate | 1996 | 21.8904474 | 33.49360783 | 10.85501092 |
| Deaths | Global | Male | 95+ years | Colon and rectum cancer | High fasting plasma glucose | Number | 1997 | 88.33073189 | 137.3293883 | 45.02894936 |
| Deaths | Global | Female | 95+ years | Colon and rectum cancer | High fasting plasma glucose | Number | 1997 | 232.9290791 | 365.9723856 | 114.0924746 |
| Deaths | Global | Both | 95+ years | Colon and rectum cancer | High fasting plasma glucose | Number | 1997 | 321.259811 | 492.5789559 | 159.2600575 |
| Deaths | Global | Male | 95+ years | Colon and rectum cancer | High fasting plasma glucose | Rate | 1997 | 23.28672222 | 36.20428868 | 11.8710285 |
| Deaths | Global | Female | 95+ years | Colon and rectum cancer | High fasting plasma glucose | Rate | 1997 | 21.69537178 | 34.08722946 | 10.62674812 |
| Deaths | Global | Both | 95+ years | Colon and rectum cancer | High fasting plasma glucose | Rate | 1997 | 22.11082082 | 33.90192194 | 10.96113014 |
| Deaths | Global | Male | 95+ years | Colon and rectum cancer | High fasting plasma glucose | Number | 1998 | 94.87855322 | 145.9020733 | 48.41321082 |
| Deaths | Global | Female | 95+ years | Colon and rectum cancer | High fasting plasma glucose | Number | 1998 | 251.7294911 | 392.6482442 | 123.324629 |
| Deaths | Global | Both | 95+ years | Colon and rectum cancer | High fasting plasma glucose | Number | 1998 | 346.6080443 | 530.3908515 | 171.2895049 |
| Deaths | Global | Male | 95+ years | Colon and rectum cancer | High fasting plasma glucose | Rate | 1998 | 23.22530072 | 35.71533727 | 11.85105951 |
| Deaths | Global | Female | 95+ years | Colon and rectum cancer | High fasting plasma glucose | Rate | 1998 | 21.98327508 | 34.28956346 | 10.7698118 |
| Deaths | Global | Both | 95+ years | Colon and rectum cancer | High fasting plasma glucose | Rate | 1998 | 22.3098595 | 34.13926933 | 11.02526283 |
| Deaths | Global | Male | 95+ years | Colon and rectum cancer | High fasting plasma glucose | Number | 1999 | 104.6338025 | 161.0229952 | 52.97638131 |
| Deaths | Global | Female | 95+ years | Colon and rectum cancer | High fasting plasma glucose | Number | 1999 | 271.6373147 | 421.6091735 | 131.9268315 |
| Deaths | Global | Both | 95+ years | Colon and rectum cancer | High fasting plasma glucose | Number | 1999 | 376.2711172 | 575.6682546 | 184.2463256 |
| Deaths | Global | Male | 95+ years | Colon and rectum cancer | High fasting plasma glucose | Rate | 1999 | 23.67731032 | 36.43747369 | 11.98788718 |
| Deaths | Global | Female | 95+ years | Colon and rectum cancer | High fasting plasma glucose | Rate | 1999 | 22.25406292 | 34.54060457 | 10.80819111 |
| Deaths | Global | Both | 95+ years | Colon and rectum cancer | High fasting plasma glucose | Rate | 1999 | 22.63237419 | 34.62593525 | 11.08225317 |
| Deaths | Global | Male | 95+ years | Colon and rectum cancer | High fasting plasma glucose | Number | 2000 | 113.4412553 | 173.1978804 | 57.23304183 |
| Deaths | Global | Female | 95+ years | Colon and rectum cancer | High fasting plasma glucose | Number | 2000 | 293.3807322 | 452.0924244 | 142.3147189 |
| Deaths | Global | Both | 95+ years | Colon and rectum cancer | High fasting plasma glucose | Number | 2000 | 406.8219876 | 620.1189127 | 199.02003 |
| Deaths | Global | Male | 95+ years | Colon and rectum cancer | High fasting plasma glucose | Rate | 2000 | 23.66377812 | 36.12897443 | 11.93877835 |
| Deaths | Global | Female | 95+ years | Colon and rectum cancer | High fasting plasma glucose | Rate | 2000 | 22.50432988 | 34.67861362 | 10.91652256 |
| Deaths | Global | Both | 95+ years | Colon and rectum cancer | High fasting plasma glucose | Rate | 2000 | 22.81605693 | 34.77852438 | 11.16176724 |
| Deaths | Global | Male | 95+ years | Colon and rectum cancer | High fasting plasma glucose | Number | 2001 | 122.7810467 | 187.6508997 | 61.90273533 |
| Deaths | Global | Female | 95+ years | Colon and rectum cancer | High fasting plasma glucose | Number | 2001 | 314.8511628 | 482.5784289 | 154.339933 |
| Deaths | Global | Both | 95+ years | Colon and rectum cancer | High fasting plasma glucose | Number | 2001 | 437.6322095 | 668.3883194 | 215.1352478 |
| Deaths | Global | Male | 95+ years | Colon and rectum cancer | High fasting plasma glucose | Rate | 2001 | 23.73956683 | 36.28207443 | 11.96881898 |
| Deaths | Global | Female | 95+ years | Colon and rectum cancer | High fasting plasma glucose | Rate | 2001 | 22.62552057 | 34.67857026 | 11.0910225 |
| Deaths | Global | Both | 95+ years | Colon and rectum cancer | High fasting plasma glucose | Rate | 2001 | 22.92738153 | 35.01660453 | 11.2708521 |
| Deaths | Global | Male | 95+ years | Colon and rectum cancer | High fasting plasma glucose | Number | 2002 | 133.8722602 | 201.6656803 | 67.60512867 |
| Deaths | Global | Female | 95+ years | Colon and rectum cancer | High fasting plasma glucose | Number | 2002 | 338.3941515 | 519.353413 | 166.5797031 |
| Deaths | Global | Both | 95+ years | Colon and rectum cancer | High fasting plasma glucose | Number | 2002 | 472.2664117 | 724.3489498 | 231.9908364 |
| Deaths | Global | Male | 95+ years | Colon and rectum cancer | High fasting plasma glucose | Rate | 2002 | 24.10724686 | 36.31524808 | 12.17409435 |
| Deaths | Global | Female | 95+ years | Colon and rectum cancer | High fasting plasma glucose | Rate | 2002 | 22.78809893 | 34.97423612 | 11.21779067 |
| Deaths | Global | Both | 95+ years | Colon and rectum cancer | High fasting plasma glucose | Rate | 2002 | 23.14714221 | 35.5024362 | 11.3705416 |
| Deaths | Global | Male | 95+ years | Colon and rectum cancer | High fasting plasma glucose | Number | 2003 | 145.7616222 | 221.2432802 | 73.4970459 |
| Deaths | Global | Female | 95+ years | Colon and rectum cancer | High fasting plasma glucose | Number | 2003 | 363.3314602 | 557.4094258 | 178.7896162 |
| Deaths | Global | Both | 95+ years | Colon and rectum cancer | High fasting plasma glucose | Number | 2003 | 509.0930824 | 782.3304548 | 250.6330107 |
| Deaths | Global | Male | 95+ years | Colon and rectum cancer | High fasting plasma glucose | Rate | 2003 | 24.74796069 | 37.56352268 | 12.47860703 |
| Deaths | Global | Female | 95+ years | Colon and rectum cancer | High fasting plasma glucose | Rate | 2003 | 23.10198506 | 35.44219435 | 11.36811836 |
| Deaths | Global | Both | 95+ years | Colon and rectum cancer | High fasting plasma glucose | Rate | 2003 | 23.55045073 | 36.19030678 | 11.59418694 |
| Deaths | Global | Male | 95+ years | Colon and rectum cancer | High fasting plasma glucose | Number | 2004 | 155.3733624 | 236.1045902 | 78.56712989 |
| Deaths | Global | Female | 95+ years | Colon and rectum cancer | High fasting plasma glucose | Number | 2004 | 387.8254079 | 592.5780372 | 188.6924888 |
| Deaths | Global | Both | 95+ years | Colon and rectum cancer | High fasting plasma glucose | Number | 2004 | 543.1987703 | 829.768577 | 266.3681442 |
| Deaths | Global | Male | 95+ years | Colon and rectum cancer | High fasting plasma glucose | Rate | 2004 | 25.03182103 | 38.03823097 | 12.65775744 |
| Deaths | Global | Female | 95+ years | Colon and rectum cancer | High fasting plasma glucose | Rate | 2004 | 23.29550632 | 35.59438121 | 11.33419053 |
| Deaths | Global | Both | 95+ years | Colon and rectum cancer | High fasting plasma glucose | Rate | 2004 | 23.76705791 | 36.305601 | 11.65464182 |
| Deaths | Global | Male | 95+ years | Colon and rectum cancer | High fasting plasma glucose | Number | 2005 | 166.0189223 | 252.0113734 | 83.95480445 |
| Deaths | Global | Female | 95+ years | Colon and rectum cancer | High fasting plasma glucose | Number | 2005 | 412.5998299 | 636.8014087 | 201.820133 |
| Deaths | Global | Both | 95+ years | Colon and rectum cancer | High fasting plasma glucose | Number | 2005 | 578.6187521 | 890.9929658 | 285.7291029 |
| Deaths | Global | Male | 95+ years | Colon and rectum cancer | High fasting plasma glucose | Rate | 2005 | 25.41096484 | 38.57302566 | 12.85017729 |
| Deaths | Global | Female | 95+ years | Colon and rectum cancer | High fasting plasma glucose | Rate | 2005 | 23.46394488 | 36.21395859 | 11.47721384 |
| Deaths | Global | Both | 95+ years | Colon and rectum cancer | High fasting plasma glucose | Rate | 2005 | 23.99138065 | 36.94341277 | 11.84724077 |
| Deaths | Global | Male | 95+ years | Colon and rectum cancer | High fasting plasma glucose | Number | 2006 | 175.3883497 | 266.9521358 | 88.66714948 |
| Deaths | Global | Female | 95+ years | Colon and rectum cancer | High fasting plasma glucose | Number | 2006 | 440.0400717 | 683.4162043 | 214.5237231 |
| Deaths | Global | Both | 95+ years | Colon and rectum cancer | High fasting plasma glucose | Number | 2006 | 615.4284214 | 938.2373743 | 303.0711514 |
| Deaths | Global | Male | 95+ years | Colon and rectum cancer | High fasting plasma glucose | Rate | 2006 | 25.50224904 | 38.8160323 | 12.89259937 |
| Deaths | Global | Female | 95+ years | Colon and rectum cancer | High fasting plasma glucose | Rate | 2006 | 23.69781372 | 36.80453429 | 11.55290974 |
| Deaths | Global | Both | 95+ years | Colon and rectum cancer | High fasting plasma glucose | Rate | 2006 | 24.18550065 | 36.87145382 | 11.9102844 |
| Deaths | Global | Male | 95+ years | Colon and rectum cancer | High fasting plasma glucose | Number | 2007 | 184.1421991 | 280.7051384 | 92.4465951 |
| Deaths | Global | Female | 95+ years | Colon and rectum cancer | High fasting plasma glucose | Number | 2007 | 474.1239094 | 726.7308624 | 232.228825 |
| Deaths | Global | Both | 95+ years | Colon and rectum cancer | High fasting plasma glucose | Number | 2007 | 658.2661084 | 994.4109959 | 324.6413904 |
| Deaths | Global | Male | 95+ years | Colon and rectum cancer | High fasting plasma glucose | Rate | 2007 | 25.40195564 | 38.72257152 | 12.7527765 |
| Deaths | Global | Female | 95+ years | Colon and rectum cancer | High fasting plasma glucose | Rate | 2007 | 24.24435442 | 37.16142606 | 11.87503484 |
| Deaths | Global | Both | 95+ years | Colon and rectum cancer | High fasting plasma glucose | Rate | 2007 | 24.55741348 | 37.09770515 | 12.11113979 |
| Deaths | Global | Male | 95+ years | Colon and rectum cancer | High fasting plasma glucose | Number | 2008 | 197.1791462 | 301.5595273 | 98.98419756 |
| Deaths | Global | Female | 95+ years | Colon and rectum cancer | High fasting plasma glucose | Number | 2008 | 508.6222635 | 779.7246872 | 247.7075257 |
| Deaths | Global | Both | 95+ years | Colon and rectum cancer | High fasting plasma glucose | Number | 2008 | 705.8014097 | 1066.943002 | 344.7110187 |
| Deaths | Global | Male | 95+ years | Colon and rectum cancer | High fasting plasma glucose | Rate | 2008 | 25.75203214 | 39.38434055 | 12.9275549 |
| Deaths | Global | Female | 95+ years | Colon and rectum cancer | High fasting plasma glucose | Rate | 2008 | 24.69746622 | 37.86154384 | 12.02807798 |
| Deaths | Global | Both | 95+ years | Colon and rectum cancer | High fasting plasma glucose | Rate | 2008 | 24.98328465 | 37.76663005 | 12.20175163 |
| Deaths | Global | Male | 95+ years | Colon and rectum cancer | High fasting plasma glucose | Number | 2009 | 209.4998447 | 320.7476066 | 105.2106722 |
| Deaths | Global | Female | 95+ years | Colon and rectum cancer | High fasting plasma glucose | Number | 2009 | 538.6109116 | 827.6877162 | 260.4695072 |
| Deaths | Global | Both | 95+ years | Colon and rectum cancer | High fasting plasma glucose | Number | 2009 | 748.1107563 | 1135.252958 | 361.9949331 |
| Deaths | Global | Male | 95+ years | Colon and rectum cancer | High fasting plasma glucose | Rate | 2009 | 25.83969112 | 39.56097961 | 12.9766744 |
| Deaths | Global | Female | 95+ years | Colon and rectum cancer | High fasting plasma glucose | Rate | 2009 | 24.84928689 | 38.18609886 | 12.01698921 |
| Deaths | Global | Both | 95+ years | Colon and rectum cancer | High fasting plasma glucose | Rate | 2009 | 25.11890162 | 38.11776149 | 12.15450391 |
| Deaths | Global | Male | 95+ years | Colon and rectum cancer | High fasting plasma glucose | Number | 2010 | 225.7080865 | 347.1491956 | 112.6693965 |
| Deaths | Global | Female | 95+ years | Colon and rectum cancer | High fasting plasma glucose | Number | 2010 | 574.1977708 | 889.1274978 | 277.6676397 |
| Deaths | Global | Both | 95+ years | Colon and rectum cancer | High fasting plasma glucose | Number | 2010 | 799.9058572 | 1212.959835 | 386.464246 |
| Deaths | Global | Male | 95+ years | Colon and rectum cancer | High fasting plasma glucose | Rate | 2010 | 26.37818027 | 40.57082847 | 13.16751073 |
| Deaths | Global | Female | 95+ years | Colon and rectum cancer | High fasting plasma glucose | Rate | 2010 | 25.27598748 | 39.13908526 | 12.22283357 |
| Deaths | Global | Both | 95+ years | Colon and rectum cancer | High fasting plasma glucose | Rate | 2010 | 25.5775518 | 38.78524294 | 12.35746579 |
| Deaths | Global | Male | 95+ years | Colon and rectum cancer | High fasting plasma glucose | Number | 2011 | 237.3049114 | 364.0902192 | 118.7803258 |
| Deaths | Global | Female | 95+ years | Colon and rectum cancer | High fasting plasma glucose | Number | 2011 | 607.254274 | 942.9866067 | 291.4937808 |
| Deaths | Global | Both | 95+ years | Colon and rectum cancer | High fasting plasma glucose | Number | 2011 | 844.5591855 | 1281.176847 | 404.751292 |
| Deaths | Global | Male | 95+ years | Colon and rectum cancer | High fasting plasma glucose | Rate | 2011 | 26.43700358 | 40.56154747 | 13.23274718 |
| Deaths | Global | Female | 95+ years | Colon and rectum cancer | High fasting plasma glucose | Rate | 2011 | 25.76309047 | 40.00671597 | 12.36678105 |
| Deaths | Global | Both | 95+ years | Colon and rectum cancer | High fasting plasma glucose | Rate | 2011 | 25.94895141 | 39.36396208 | 12.43592136 |
| Deaths | Global | Male | 95+ years | Colon and rectum cancer | High fasting plasma glucose | Number | 2012 | 248.4061837 | 381.5538887 | 123.9148697 |
| Deaths | Global | Female | 95+ years | Colon and rectum cancer | High fasting plasma glucose | Number | 2012 | 632.0402904 | 981.5258267 | 304.4850763 |
| Deaths | Global | Both | 95+ years | Colon and rectum cancer | High fasting plasma glucose | Number | 2012 | 880.4464742 | 1353.933617 | 422.7322855 |
| Deaths | Global | Male | 95+ years | Colon and rectum cancer | High fasting plasma glucose | Rate | 2012 | 26.41321762 | 40.57091391 | 13.17596193 |
| Deaths | Global | Female | 95+ years | Colon and rectum cancer | High fasting plasma glucose | Rate | 2012 | 25.95456806 | 40.30609957 | 12.50359947 |
| Deaths | Global | Both | 95+ years | Colon and rectum cancer | High fasting plasma glucose | Rate | 2012 | 26.08234893 | 40.10893344 | 12.52302246 |
| Deaths | Global | Male | 95+ years | Colon and rectum cancer | High fasting plasma glucose | Number | 2013 | 259.6273146 | 397.2861817 | 129.27758 |
| Deaths | Global | Female | 95+ years | Colon and rectum cancer | High fasting plasma glucose | Number | 2013 | 652.4458875 | 1019.550226 | 311.3844629 |
| Deaths | Global | Both | 95+ years | Colon and rectum cancer | High fasting plasma glucose | Number | 2013 | 912.0732021 | 1405.157279 | 438.0157089 |
| Deaths | Global | Male | 95+ years | Colon and rectum cancer | High fasting plasma glucose | Rate | 2013 | 26.4503833 | 40.47483141 | 13.17057702 |
| Deaths | Global | Female | 95+ years | Colon and rectum cancer | High fasting plasma glucose | Rate | 2013 | 25.92889015 | 40.5180051 | 12.37474814 |
| Deaths | Global | Both | 95+ years | Colon and rectum cancer | High fasting plasma glucose | Rate | 2013 | 26.07523097 | 40.17199552 | 12.52241679 |
| Deaths | Global | Male | 95+ years | Colon and rectum cancer | High fasting plasma glucose | Number | 2014 | 275.1558542 | 422.4737084 | 135.6696898 |
| Deaths | Global | Female | 95+ years | Colon and rectum cancer | High fasting plasma glucose | Number | 2014 | 681.9273526 | 1074.297033 | 325.8389348 |
| Deaths | Global | Both | 95+ years | Colon and rectum cancer | High fasting plasma glucose | Number | 2014 | 957.0832069 | 1477.230672 | 457.6583002 |
| Deaths | Global | Male | 95+ years | Colon and rectum cancer | High fasting plasma glucose | Rate | 2014 | 26.93899607 | 41.36207678 | 13.28267302 |
| Deaths | Global | Female | 95+ years | Colon and rectum cancer | High fasting plasma glucose | Rate | 2014 | 25.98111853 | 40.93022292 | 12.41431357 |
| Deaths | Global | Both | 95+ years | Colon and rectum cancer | High fasting plasma glucose | Rate | 2014 | 26.2494539 | 40.5152845 | 12.55197078 |
| Deaths | Global | Male | 95+ years | Colon and rectum cancer | High fasting plasma glucose | Number | 2015 | 301.0630613 | 463.550905 | 148.3592229 |
| Deaths | Global | Female | 95+ years | Colon and rectum cancer | High fasting plasma glucose | Number | 2015 | 745.8566548 | 1174.032259 | 352.9114163 |
| Deaths | Global | Both | 95+ years | Colon and rectum cancer | High fasting plasma glucose | Number | 2015 | 1046.919716 | 1626.315361 | 497.3923602 |
| Deaths | Global | Male | 95+ years | Colon and rectum cancer | High fasting plasma glucose | Rate | 2015 | 27.92166316 | 42.9913659 | 13.75936401 |
| Deaths | Global | Female | 95+ years | Colon and rectum cancer | High fasting plasma glucose | Rate | 2015 | 26.66973264 | 41.98008595 | 12.61911798 |
| Deaths | Global | Both | 95+ years | Colon and rectum cancer | High fasting plasma glucose | Rate | 2015 | 27.01810032 | 41.97069831 | 12.83632018 |
| Deaths | Global | Male | 95+ years | Colon and rectum cancer | High fasting plasma glucose | Number | 2016 | 328.3168679 | 506.3150288 | 161.9806329 |
| Deaths | Global | Female | 95+ years | Colon and rectum cancer | High fasting plasma glucose | Number | 2016 | 810.453019 | 1276.829686 | 387.8576606 |
| Deaths | Global | Both | 95+ years | Colon and rectum cancer | High fasting plasma glucose | Number | 2016 | 1138.769887 | 1771.851876 | 544.1454262 |
| Deaths | Global | Male | 95+ years | Colon and rectum cancer | High fasting plasma glucose | Rate | 2016 | 28.68905986 | 44.24293598 | 14.15422882 |
| Deaths | Global | Female | 95+ years | Colon and rectum cancer | High fasting plasma glucose | Rate | 2016 | 27.13166167 | 42.74462583 | 12.98437118 |
| Deaths | Global | Both | 95+ years | Colon and rectum cancer | High fasting plasma glucose | Rate | 2016 | 27.56304945 | 42.88631217 | 13.17062162 |
| Deaths | Global | Male | 95+ years | Colon and rectum cancer | High fasting plasma glucose | Number | 2017 | 359.239073 | 552.6991895 | 178.69354 |
| Deaths | Global | Female | 95+ years | Colon and rectum cancer | High fasting plasma glucose | Number | 2017 | 892.5858094 | 1417.467112 | 428.5413834 |
| Deaths | Global | Both | 95+ years | Colon and rectum cancer | High fasting plasma glucose | Number | 2017 | 1251.824882 | 1941.912322 | 599.5888494 |
| Deaths | Global | Male | 95+ years | Colon and rectum cancer | High fasting plasma glucose | Rate | 2017 | 29.51991368 | 45.41719871 | 14.68386451 |
| Deaths | Global | Female | 95+ years | Colon and rectum cancer | High fasting plasma glucose | Rate | 2017 | 28.06895472 | 44.57478459 | 13.47624907 |
| Deaths | Global | Both | 95+ years | Colon and rectum cancer | High fasting plasma glucose | Rate | 2017 | 28.47053808 | 44.16535371 | 13.6365856 |
| Deaths | Global | Male | 95+ years | Colon and rectum cancer | High fasting plasma glucose | Number | 2018 | 389.4520037 | 602.8465543 | 194.5623864 |
| Deaths | Global | Female | 95+ years | Colon and rectum cancer | High fasting plasma glucose | Number | 2018 | 965.7280518 | 1530.871461 | 456.0708378 |
| Deaths | Global | Both | 95+ years | Colon and rectum cancer | High fasting plasma glucose | Number | 2018 | 1355.180056 | 2092.346923 | 639.9118425 |
| Deaths | Global | Male | 95+ years | Colon and rectum cancer | High fasting plasma glucose | Rate | 2018 | 30.12432404 | 46.63050845 | 15.04950628 |
| Deaths | Global | Female | 95+ years | Colon and rectum cancer | High fasting plasma glucose | Rate | 2018 | 28.62771377 | 45.38063268 | 13.51960873 |
| Deaths | Global | Both | 95+ years | Colon and rectum cancer | High fasting plasma glucose | Rate | 2018 | 29.04236245 | 44.84031288 | 13.71371398 |
| Deaths | Global | Male | 95+ years | Colon and rectum cancer | High fasting plasma glucose | Number | 2019 | 420.4693171 | 647.4098355 | 210.2575904 |
| Deaths | Global | Female | 95+ years | Colon and rectum cancer | High fasting plasma glucose | Number | 2019 | 1024.984357 | 1627.097775 | 485.6217642 |
| Deaths | Global | Both | 95+ years | Colon and rectum cancer | High fasting plasma glucose | Number | 2019 | 1445.453674 | 2210.27098 | 688.8480233 |
| Deaths | Global | Male | 95+ years | Colon and rectum cancer | High fasting plasma glucose | Rate | 2019 | 30.49844306 | 46.95941226 | 15.25088487 |
| Deaths | Global | Female | 95+ years | Colon and rectum cancer | High fasting plasma glucose | Rate | 2019 | 28.68399676 | 45.53402886 | 13.59003482 |
| Deaths | Global | Both | 95+ years | Colon and rectum cancer | High fasting plasma glucose | Rate | 2019 | 29.18914395 | 44.63368072 | 13.91043135 |
| Deaths | Global | Male | 95+ years | Colon and rectum cancer | High fasting plasma glucose | Number | 2020 | 447.3170539 | 692.3373035 | 223.8075006 |
| Deaths | Global | Female | 95+ years | Colon and rectum cancer | High fasting plasma glucose | Number | 2020 | 1066.346059 | 1694.837212 | 504.8669634 |
| Deaths | Global | Both | 95+ years | Colon and rectum cancer | High fasting plasma glucose | Number | 2020 | 1513.663113 | 2328.894984 | 724.6579318 |
| Deaths | Global | Male | 95+ years | Colon and rectum cancer | High fasting plasma glucose | Rate | 2020 | 30.69337169 | 47.50582613 | 15.35690791 |
| Deaths | Global | Female | 95+ years | Colon and rectum cancer | High fasting plasma glucose | Rate | 2020 | 28.280191 | 44.94818513 | 13.38940022 |
| Deaths | Global | Both | 95+ years | Colon and rectum cancer | High fasting plasma glucose | Rate | 2020 | 28.95289414 | 44.54640493 | 13.86103963 |
| Deaths | Global | Male | 95+ years | Colon and rectum cancer | High fasting plasma glucose | Number | 2021 | 470.6198529 | 736.7348494 | 232.5951029 |
| Deaths | Global | Female | 95+ years | Colon and rectum cancer | High fasting plasma glucose | Number | 2021 | 1106.760721 | 1764.897671 | 529.4925037 |
| Deaths | Global | Both | 95+ years | Colon and rectum cancer | High fasting plasma glucose | Number | 2021 | 1577.380573 | 2422.903808 | 754.763071 |
| Deaths | Global | Male | 95+ years | Colon and rectum cancer | High fasting plasma glucose | Rate | 2021 | 31.12478951 | 48.72450018 | 15.38284791 |
| Deaths | Global | Female | 95+ years | Colon and rectum cancer | High fasting plasma glucose | Rate | 2021 | 28.10273602 | 44.81407085 | 13.44481041 |
| Deaths | Global | Both | 95+ years | Colon and rectum cancer | High fasting plasma glucose | Rate | 2021 | 28.94112366 | 44.45443282 | 13.84807937 |
| Deaths | Global | Male | 25-29 years | Colon and rectum cancer | High fasting plasma glucose | Number | 1990 | 29.20144245 | 52.92856379 | 13.5680984 |
| Deaths | Global | Female | 25-29 years | Colon and rectum cancer | High fasting plasma glucose | Number | 1990 | 20.20979831 | 36.48361185 | 8.47742489 |
| Deaths | Global | Both | 25-29 years | Colon and rectum cancer | High fasting plasma glucose | Number | 1990 | 49.41124076 | 83.75150918 | 23.20053471 |
| Deaths | Global | Male | 25-29 years | Colon and rectum cancer | High fasting plasma glucose | Rate | 1990 | 0.01312306 | 0.023785972 | 0.006097472 |
| Deaths | Global | Female | 25-29 years | Colon and rectum cancer | High fasting plasma glucose | Rate | 1990 | 0.009182116 | 0.016575957 | 0.003851632 |
| Deaths | Global | Both | 25-29 years | Colon and rectum cancer | High fasting plasma glucose | Rate | 1990 | 0.011163364 | 0.018921778 | 0.005241641 |
| Deaths | Global | Male | 25-29 years | Colon and rectum cancer | High fasting plasma glucose | Number | 1991 | 30.92151189 | 55.67045052 | 14.13708113 |
| Deaths | Global | Female | 25-29 years | Colon and rectum cancer | High fasting plasma glucose | Number | 1991 | 21.16183125 | 37.75391838 | 9.022600232 |
| Deaths | Global | Both | 25-29 years | Colon and rectum cancer | High fasting plasma glucose | Number | 1991 | 52.08334314 | 86.91969844 | 24.19151466 |
| Deaths | Global | Male | 25-29 years | Colon and rectum cancer | High fasting plasma glucose | Rate | 1991 | 0.013489171 | 0.024285624 | 0.006167147 |
| Deaths | Global | Female | 25-29 years | Colon and rectum cancer | High fasting plasma glucose | Rate | 1991 | 0.009342569 | 0.016667677 | 0.003983316 |
| Deaths | Global | Both | 25-29 years | Colon and rectum cancer | High fasting plasma glucose | Rate | 1991 | 0.011428254 | 0.019072132 | 0.005308161 |
| Deaths | Global | Male | 25-29 years | Colon and rectum cancer | High fasting plasma glucose | Number | 1992 | 32.06766695 | 57.81975654 | 14.59514901 |
| Deaths | Global | Female | 25-29 years | Colon and rectum cancer | High fasting plasma glucose | Number | 1992 | 21.75592814 | 38.91126021 | 9.298040121 |
| Deaths | Global | Both | 25-29 years | Colon and rectum cancer | High fasting plasma glucose | Number | 1992 | 53.82359509 | 92.28899587 | 25.14234718 |
| Deaths | Global | Male | 25-29 years | Colon and rectum cancer | High fasting plasma glucose | Rate | 1992 | 0.013644336 | 0.024601484 | 0.006210028 |
| Deaths | Global | Female | 25-29 years | Colon and rectum cancer | High fasting plasma glucose | Rate | 1992 | 0.009381826 | 0.016779733 | 0.004009601 |
| Deaths | Global | Both | 25-29 years | Colon and rectum cancer | High fasting plasma glucose | Rate | 1992 | 0.011527373 | 0.019765489 | 0.005384724 |
| Deaths | Global | Male | 25-29 years | Colon and rectum cancer | High fasting plasma glucose | Number | 1993 | 32.73394453 | 60.0196675 | 14.82280076 |
| Deaths | Global | Female | 25-29 years | Colon and rectum cancer | High fasting plasma glucose | Number | 1993 | 21.92819499 | 39.48998028 | 9.164763608 |
| Deaths | Global | Both | 25-29 years | Colon and rectum cancer | High fasting plasma glucose | Number | 1993 | 54.66213952 | 90.77158474 | 25.61874331 |
| Deaths | Global | Male | 25-29 years | Colon and rectum cancer | High fasting plasma glucose | Rate | 1993 | 0.013685119 | 0.025092493 | 0.006196986 |
| Deaths | Global | Female | 25-29 years | Colon and rectum cancer | High fasting plasma glucose | Rate | 1993 | 0.009308031 | 0.016762619 | 0.003890238 |
| Deaths | Global | Both | 25-29 years | Colon and rectum cancer | High fasting plasma glucose | Rate | 1993 | 0.011513216 | 0.01911877 | 0.005395949 |
| Deaths | Global | Male | 25-29 years | Colon and rectum cancer | High fasting plasma glucose | Number | 1994 | 32.86120933 | 57.93740562 | 14.92426149 |
| Deaths | Global | Female | 25-29 years | Colon and rectum cancer | High fasting plasma glucose | Number | 1994 | 22.13822095 | 39.25403975 | 9.563513444 |
| Deaths | Global | Both | 25-29 years | Colon and rectum cancer | High fasting plasma glucose | Number | 1994 | 54.99943028 | 91.72358348 | 25.47377052 |
| Deaths | Global | Male | 25-29 years | Colon and rectum cancer | High fasting plasma glucose | Rate | 1994 | 0.013570386 | 0.023925868 | 0.006163133 |
| Deaths | Global | Female | 25-29 years | Colon and rectum cancer | High fasting plasma glucose | Rate | 1994 | 0.009295508 | 0.016482184 | 0.004015576 |
| Deaths | Global | Both | 25-29 years | Colon and rectum cancer | High fasting plasma glucose | Rate | 1994 | 0.011450718 | 0.019096577 | 0.005303563 |
| Deaths | Global | Male | 25-29 years | Colon and rectum cancer | High fasting plasma glucose | Number | 1995 | 32.84373982 | 58.70720439 | 14.35289365 |
| Deaths | Global | Female | 25-29 years | Colon and rectum cancer | High fasting plasma glucose | Number | 1995 | 22.10267647 | 38.64068294 | 9.20667082 |
| Deaths | Global | Both | 25-29 years | Colon and rectum cancer | High fasting plasma glucose | Number | 1995 | 54.94641629 | 91.88987176 | 25.38197934 |
| Deaths | Global | Male | 25-29 years | Colon and rectum cancer | High fasting plasma glucose | Rate | 1995 | 0.013436093 | 0.024016615 | 0.005871646 |
| Deaths | Global | Female | 25-29 years | Colon and rectum cancer | High fasting plasma glucose | Rate | 1995 | 0.00920157 | 0.016086512 | 0.003832831 |
| Deaths | Global | Both | 25-29 years | Colon and rectum cancer | High fasting plasma glucose | Rate | 1995 | 0.011337349 | 0.018960063 | 0.005237181 |
| Deaths | Global | Male | 25-29 years | Colon and rectum cancer | High fasting plasma glucose | Number | 1996 | 33.01056725 | 58.07709086 | 14.90290506 |
| Deaths | Global | Female | 25-29 years | Colon and rectum cancer | High fasting plasma glucose | Number | 1996 | 21.98626913 | 37.97585348 | 9.502932358 |
| Deaths | Global | Both | 25-29 years | Colon and rectum cancer | High fasting plasma glucose | Number | 1996 | 54.99683638 | 90.6871991 | 25.58465174 |
| Deaths | Global | Male | 25-29 years | Colon and rectum cancer | High fasting plasma glucose | Rate | 1996 | 0.013379653 | 0.023539472 | 0.00604036 |
| Deaths | Global | Female | 25-29 years | Colon and rectum cancer | High fasting plasma glucose | Rate | 1996 | 0.009071181 | 0.015668226 | 0.003920757 |
| Deaths | Global | Both | 25-29 years | Colon and rectum cancer | High fasting plasma glucose | Rate | 1996 | 0.011244564 | 0.018541758 | 0.005230996 |
| Deaths | Global | Male | 25-29 years | Colon and rectum cancer | High fasting plasma glucose | Number | 1997 | 33.00378027 | 58.63396433 | 15.36938944 |
| Deaths | Global | Female | 25-29 years | Colon and rectum cancer | High fasting plasma glucose | Number | 1997 | 21.81993186 | 37.62883187 | 9.378784611 |
| Deaths | Global | Both | 25-29 years | Colon and rectum cancer | High fasting plasma glucose | Number | 1997 | 54.82371213 | 90.05411652 | 25.42538127 |
| Deaths | Global | Male | 25-29 years | Colon and rectum cancer | High fasting plasma glucose | Rate | 1997 | 0.013287875 | 0.023607017 | 0.006187974 |
| Deaths | Global | Female | 25-29 years | Colon and rectum cancer | High fasting plasma glucose | Rate | 1997 | 0.008929306 | 0.015398735 | 0.003838052 |
| Deaths | Global | Both | 25-29 years | Colon and rectum cancer | High fasting plasma glucose | Rate | 1997 | 0.011126335 | 0.018276257 | 0.005160017 |
| Deaths | Global | Male | 25-29 years | Colon and rectum cancer | High fasting plasma glucose | Number | 1998 | 32.6743424 | 57.13682409 | 15.19323856 |
| Deaths | Global | Female | 25-29 years | Colon and rectum cancer | High fasting plasma glucose | Number | 1998 | 21.98765882 | 36.96276105 | 9.746748332 |
| Deaths | Global | Both | 25-29 years | Colon and rectum cancer | High fasting plasma glucose | Number | 1998 | 54.66200121 | 90.58770617 | 25.76410105 |
| Deaths | Global | Male | 25-29 years | Colon and rectum cancer | High fasting plasma glucose | Rate | 1998 | 0.013096723 | 0.022901919 | 0.006089844 |
| Deaths | Global | Female | 25-29 years | Colon and rectum cancer | High fasting plasma glucose | Rate | 1998 | 0.008929497 | 0.015011096 | 0.003958291 |
| Deaths | Global | Both | 25-29 years | Colon and rectum cancer | High fasting plasma glucose | Rate | 1998 | 0.011026764 | 0.018273924 | 0.005197297 |
| Deaths | Global | Male | 25-29 years | Colon and rectum cancer | High fasting plasma glucose | Number | 1999 | 32.8975147 | 57.33819546 | 15.29491854 |
| Deaths | Global | Female | 25-29 years | Colon and rectum cancer | High fasting plasma glucose | Number | 1999 | 22.11405015 | 37.29458742 | 9.442363884 |
| Deaths | Global | Both | 25-29 years | Colon and rectum cancer | High fasting plasma glucose | Number | 1999 | 55.01156485 | 90.8756459 | 25.75894771 |
| Deaths | Global | Male | 25-29 years | Colon and rectum cancer | High fasting plasma glucose | Rate | 1999 | 0.01315998 | 0.022936975 | 0.00611842 |
| Deaths | Global | Female | 25-29 years | Colon and rectum cancer | High fasting plasma glucose | Rate | 1999 | 0.008924514 | 0.015050886 | 0.003810632 |
| Deaths | Global | Both | 25-29 years | Colon and rectum cancer | High fasting plasma glucose | Rate | 1999 | 0.01105157 | 0.018256499 | 0.005174854 |
| Deaths | Global | Male | 25-29 years | Colon and rectum cancer | High fasting plasma glucose | Number | 2000 | 33.29915718 | 58.25706843 | 15.74464137 |
| Deaths | Global | Female | 25-29 years | Colon and rectum cancer | High fasting plasma glucose | Number | 2000 | 21.84018422 | 37.61963722 | 9.454758239 |
| Deaths | Global | Both | 25-29 years | Colon and rectum cancer | High fasting plasma glucose | Number | 2000 | 55.13934139 | 89.53520371 | 25.92065248 |
| Deaths | Global | Male | 25-29 years | Colon and rectum cancer | High fasting plasma glucose | Rate | 2000 | 0.013324738 | 0.023311707 | 0.006300256 |
| Deaths | Global | Female | 25-29 years | Colon and rectum cancer | High fasting plasma glucose | Rate | 2000 | 0.008776725 | 0.015117877 | 0.003799502 |
| Deaths | Global | Both | 25-29 years | Colon and rectum cancer | High fasting plasma glucose | Rate | 2000 | 0.011055577 | 0.017952034 | 0.005197156 |
| Deaths | Global | Male | 25-29 years | Colon and rectum cancer | High fasting plasma glucose | Number | 2001 | 32.72046832 | 56.61233209 | 15.33884362 |
| Deaths | Global | Female | 25-29 years | Colon and rectum cancer | High fasting plasma glucose | Number | 2001 | 21.25326715 | 35.44188275 | 9.477225866 |
| Deaths | Global | Both | 25-29 years | Colon and rectum cancer | High fasting plasma glucose | Number | 2001 | 53.97373547 | 86.46472606 | 25.78601872 |
| Deaths | Global | Male | 25-29 years | Colon and rectum cancer | High fasting plasma glucose | Rate | 2001 | 0.01311805 | 0.0226966 | 0.006149536 |
| Deaths | Global | Female | 25-29 years | Colon and rectum cancer | High fasting plasma glucose | Rate | 2001 | 0.008526571 | 0.014218883 | 0.003802156 |
| Deaths | Global | Both | 25-29 years | Colon and rectum cancer | High fasting plasma glucose | Rate | 2001 | 0.0108231 | 0.017338366 | 0.005170749 |
| Deaths | Global | Male | 25-29 years | Colon and rectum cancer | High fasting plasma glucose | Number | 2002 | 32.01422531 | 53.9004318 | 15.46972141 |
| Deaths | Global | Female | 25-29 years | Colon and rectum cancer | High fasting plasma glucose | Number | 2002 | 20.70624601 | 33.80134978 | 9.412389624 |
| Deaths | Global | Both | 25-29 years | Colon and rectum cancer | High fasting plasma glucose | Number | 2002 | 52.72047133 | 84.75616029 | 25.64063465 |
| Deaths | Global | Male | 25-29 years | Colon and rectum cancer | High fasting plasma glucose | Rate | 2002 | 0.012855799 | 0.021644538 | 0.006212102 |
| Deaths | Global | Female | 25-29 years | Colon and rectum cancer | High fasting plasma glucose | Rate | 2002 | 0.008301326 | 0.013551274 | 0.003773514 |
| Deaths | Global | Both | 25-29 years | Colon and rectum cancer | High fasting plasma glucose | Rate | 2002 | 0.010576701 | 0.017003652 | 0.005143985 |
| Deaths | Global | Male | 25-29 years | Colon and rectum cancer | High fasting plasma glucose | Number | 2003 | 32.48092834 | 54.63612829 | 15.44326255 |
| Deaths | Global | Female | 25-29 years | Colon and rectum cancer | High fasting plasma glucose | Number | 2003 | 20.30621409 | 33.07418157 | 9.436863218 |
| Deaths | Global | Both | 25-29 years | Colon and rectum cancer | High fasting plasma glucose | Number | 2003 | 52.78714242 | 84.47794244 | 25.74219572 |
| Deaths | Global | Male | 25-29 years | Colon and rectum cancer | High fasting plasma glucose | Rate | 2003 | 0.013030153 | 0.021918004 | 0.006195269 |
| Deaths | Global | Female | 25-29 years | Colon and rectum cancer | High fasting plasma glucose | Rate | 2003 | 0.00812446 | 0.013232889 | 0.003775663 |
| Deaths | Global | Both | 25-29 years | Colon and rectum cancer | High fasting plasma glucose | Rate | 2003 | 0.010574044 | 0.016922178 | 0.005156542 |
| Deaths | Global | Male | 25-29 years | Colon and rectum cancer | High fasting plasma glucose | Number | 2004 | 32.59374324 | 55.27610875 | 15.52203536 |
| Deaths | Global | Female | 25-29 years | Colon and rectum cancer | High fasting plasma glucose | Number | 2004 | 20.00881172 | 31.63666644 | 9.48289397 |
| Deaths | Global | Both | 25-29 years | Colon and rectum cancer | High fasting plasma glucose | Number | 2004 | 52.60255495 | 84.11676037 | 25.3847061 |
| Deaths | Global | Male | 25-29 years | Colon and rectum cancer | High fasting plasma glucose | Rate | 2004 | 0.013002589 | 0.022051241 | 0.00619219 |
| Deaths | Global | Female | 25-29 years | Colon and rectum cancer | High fasting plasma glucose | Rate | 2004 | 0.00796078 | 0.012587081 | 0.003772899 |
| Deaths | Global | Both | 25-29 years | Colon and rectum cancer | High fasting plasma glucose | Rate | 2004 | 0.010478314 | 0.016755875 | 0.005056578 |
| Deaths | Global | Male | 25-29 years | Colon and rectum cancer | High fasting plasma glucose | Number | 2005 | 31.82539062 | 52.81916933 | 15.20800438 |
| Deaths | Global | Female | 25-29 years | Colon and rectum cancer | High fasting plasma glucose | Number | 2005 | 19.59834286 | 30.79261348 | 9.031606221 |
| Deaths | Global | Both | 25-29 years | Colon and rectum cancer | High fasting plasma glucose | Number | 2005 | 51.42373348 | 80.99778223 | 24.85446582 |
| Deaths | Global | Male | 25-29 years | Colon and rectum cancer | High fasting plasma glucose | Rate | 2005 | 0.012564208 | 0.020852251 | 0.006003902 |
| Deaths | Global | Female | 25-29 years | Colon and rectum cancer | High fasting plasma glucose | Rate | 2005 | 0.007721688 | 0.012132198 | 0.003558426 |
| Deaths | Global | Both | 25-29 years | Colon and rectum cancer | High fasting plasma glucose | Rate | 2005 | 0.010140527 | 0.015972396 | 0.004901188 |
| Deaths | Global | Male | 25-29 years | Colon and rectum cancer | High fasting plasma glucose | Number | 2006 | 30.26190145 | 49.28593363 | 14.53506731 |
| Deaths | Global | Female | 25-29 years | Colon and rectum cancer | High fasting plasma glucose | Number | 2006 | 19.20521766 | 30.67538309 | 8.920196708 |
| Deaths | Global | Both | 25-29 years | Colon and rectum cancer | High fasting plasma glucose | Number | 2006 | 49.46711911 | 78.05951399 | 24.35944784 |
| Deaths | Global | Male | 25-29 years | Colon and rectum cancer | High fasting plasma glucose | Rate | 2006 | 0.011787086 | 0.019196994 | 0.005661445 |
| Deaths | Global | Female | 25-29 years | Colon and rectum cancer | High fasting plasma glucose | Rate | 2006 | 0.007472782 | 0.011935842 | 0.003470863 |
| Deaths | Global | Both | 25-29 years | Colon and rectum cancer | High fasting plasma glucose | Rate | 2006 | 0.009628824 | 0.015194361 | 0.004741591 |
| Deaths | Global | Male | 25-29 years | Colon and rectum cancer | High fasting plasma glucose | Number | 2007 | 30.37628693 | 48.97590683 | 14.59707173 |
| Deaths | Global | Female | 25-29 years | Colon and rectum cancer | High fasting plasma glucose | Number | 2007 | 19.21993172 | 30.74363985 | 8.988225673 |
| Deaths | Global | Both | 25-29 years | Colon and rectum cancer | High fasting plasma glucose | Number | 2007 | 49.59621864 | 78.34840694 | 24.24111752 |
| Deaths | Global | Male | 25-29 years | Colon and rectum cancer | High fasting plasma glucose | Rate | 2007 | 0.011643979 | 0.018773671 | 0.005595417 |
| Deaths | Global | Female | 25-29 years | Colon and rectum cancer | High fasting plasma glucose | Rate | 2007 | 0.007367992 | 0.011785624 | 0.003445651 |
| Deaths | Global | Both | 25-29 years | Colon and rectum cancer | High fasting plasma glucose | Rate | 2007 | 0.00950606 | 0.015016965 | 0.004646272 |
| Deaths | Global | Male | 25-29 years | Colon and rectum cancer | High fasting plasma glucose | Number | 2008 | 31.72960749 | 50.96771596 | 15.39047083 |
| Deaths | Global | Female | 25-29 years | Colon and rectum cancer | High fasting plasma glucose | Number | 2008 | 20.51880539 | 32.24916485 | 9.651720856 |
| Deaths | Global | Both | 25-29 years | Colon and rectum cancer | High fasting plasma glucose | Number | 2008 | 52.24841288 | 82.08390487 | 25.32125688 |
| Deaths | Global | Male | 25-29 years | Colon and rectum cancer | High fasting plasma glucose | Rate | 2008 | 0.011933765 | 0.019169376 | 0.005788482 |
| Deaths | Global | Female | 25-29 years | Colon and rectum cancer | High fasting plasma glucose | Rate | 2008 | 0.007727421 | 0.012145097 | 0.003634856 |
| Deaths | Global | Both | 25-29 years | Colon and rectum cancer | High fasting plasma glucose | Rate | 2008 | 0.009831973 | 0.015446339 | 0.004764889 |
| Deaths | Global | Male | 25-29 years | Colon and rectum cancer | High fasting plasma glucose | Number | 2009 | 34.14372631 | 55.80376846 | 16.32084246 |
| Deaths | Global | Female | 25-29 years | Colon and rectum cancer | High fasting plasma glucose | Number | 2009 | 22.13337621 | 34.8702186 | 10.49661989 |
| Deaths | Global | Both | 25-29 years | Colon and rectum cancer | High fasting plasma glucose | Number | 2009 | 56.27710252 | 89.06967943 | 27.3957155 |
| Deaths | Global | Male | 25-29 years | Colon and rectum cancer | High fasting plasma glucose | Rate | 2009 | 0.012572398 | 0.020548055 | 0.006009658 |
| Deaths | Global | Female | 25-29 years | Colon and rectum cancer | High fasting plasma glucose | Rate | 2009 | 0.008169108 | 0.012870091 | 0.00387415 |
| Deaths | Global | Both | 25-29 years | Colon and rectum cancer | High fasting plasma glucose | Rate | 2009 | 0.010373337 | 0.016417864 | 0.005049745 |
| Deaths | Global | Male | 25-29 years | Colon and rectum cancer | High fasting plasma glucose | Number | 2010 | 36.2054361 | 58.02662554 | 17.2572073 |
| Deaths | Global | Female | 25-29 years | Colon and rectum cancer | High fasting plasma glucose | Number | 2010 | 22.99108445 | 35.99098739 | 10.9891457 |
| Deaths | Global | Both | 25-29 years | Colon and rectum cancer | High fasting plasma glucose | Number | 2010 | 59.19652055 | 93.85789863 | 28.43374778 |
| Deaths | Global | Male | 25-29 years | Colon and rectum cancer | High fasting plasma glucose | Rate | 2010 | 0.013042634 | 0.020903492 | 0.00621673 |
| Deaths | Global | Female | 25-29 years | Colon and rectum cancer | High fasting plasma glucose | Rate | 2010 | 0.008303953 | 0.012999277 | 0.003969076 |
| Deaths | Global | Both | 25-29 years | Colon and rectum cancer | High fasting plasma glucose | Rate | 2010 | 0.010676387 | 0.016927739 | 0.005128168 |
| Deaths | Global | Male | 25-29 years | Colon and rectum cancer | High fasting plasma glucose | Number | 2011 | 37.44111062 | 61.03602228 | 17.77273559 |
| Deaths | Global | Female | 25-29 years | Colon and rectum cancer | High fasting plasma glucose | Number | 2011 | 23.38071344 | 37.30365767 | 10.96068772 |
| Deaths | Global | Both | 25-29 years | Colon and rectum cancer | High fasting plasma glucose | Number | 2011 | 60.82182406 | 96.87934838 | 29.38046636 |
| Deaths | Global | Male | 25-29 years | Colon and rectum cancer | High fasting plasma glucose | Rate | 2011 | 0.013178299 | 0.021483095 | 0.006255541 |
| Deaths | Global | Female | 25-29 years | Colon and rectum cancer | High fasting plasma glucose | Rate | 2011 | 0.008248996 | 0.013161178 | 0.003867062 |
| Deaths | Global | Both | 25-29 years | Colon and rectum cancer | High fasting plasma glucose | Rate | 2011 | 0.010716578 | 0.017069779 | 0.005176728 |
| Deaths | Global | Male | 25-29 years | Colon and rectum cancer | High fasting plasma glucose | Number | 2012 | 39.10039974 | 63.58827488 | 18.66658097 |
| Deaths | Global | Female | 25-29 years | Colon and rectum cancer | High fasting plasma glucose | Number | 2012 | 23.66073186 | 37.32325568 | 11.33976938 |
| Deaths | Global | Both | 25-29 years | Colon and rectum cancer | High fasting plasma glucose | Number | 2012 | 62.7611316 | 99.689582 | 30.36855466 |
| Deaths | Global | Male | 25-29 years | Colon and rectum cancer | High fasting plasma glucose | Rate | 2012 | 0.013447526 | 0.021869469 | 0.006419866 |
| Deaths | Global | Female | 25-29 years | Colon and rectum cancer | High fasting plasma glucose | Rate | 2012 | 0.008158513 | 0.01286952 | 0.003910093 |
| Deaths | Global | Both | 25-29 years | Colon and rectum cancer | High fasting plasma glucose | Rate | 2012 | 0.010806434 | 0.017164906 | 0.005228966 |
| Deaths | Global | Male | 25-29 years | Colon and rectum cancer | High fasting plasma glucose | Number | 2013 | 40.84518296 | 66.87907285 | 19.52124983 |
| Deaths | Global | Female | 25-29 years | Colon and rectum cancer | High fasting plasma glucose | Number | 2013 | 24.42062233 | 39.243903 | 11.69943558 |
| Deaths | Global | Both | 25-29 years | Colon and rectum cancer | High fasting plasma glucose | Number | 2013 | 65.26580529 | 103.711401 | 31.78065628 |
| Deaths | Global | Male | 25-29 years | Colon and rectum cancer | High fasting plasma glucose | Rate | 2013 | 0.013765359 | 0.022539119 | 0.006578916 |
| Deaths | Global | Female | 25-29 years | Colon and rectum cancer | High fasting plasma glucose | Rate | 2013 | 0.008258276 | 0.013271036 | 0.003956376 |
| Deaths | Global | Both | 25-29 years | Colon and rectum cancer | High fasting plasma glucose | Rate | 2013 | 0.011016528 | 0.017505944 | 0.005364409 |
| Deaths | Global | Male | 25-29 years | Colon and rectum cancer | High fasting plasma glucose | Number | 2014 | 42.17307516 | 70.12681511 | 20.119184 |
| Deaths | Global | Female | 25-29 years | Colon and rectum cancer | High fasting plasma glucose | Number | 2014 | 24.42077652 | 39.38885599 | 11.55403198 |
| Deaths | Global | Both | 25-29 years | Colon and rectum cancer | High fasting plasma glucose | Number | 2014 | 66.59385168 | 107.2906673 | 32.73749632 |
| Deaths | Global | Male | 25-29 years | Colon and rectum cancer | High fasting plasma glucose | Rate | 2014 | 0.013992811 | 0.023267719 | 0.006675442 |
| Deaths | Global | Female | 25-29 years | Colon and rectum cancer | High fasting plasma glucose | Rate | 2014 | 0.008143536 | 0.013134904 | 0.003852894 |
| Deaths | Global | Both | 25-29 years | Colon and rectum cancer | High fasting plasma glucose | Rate | 2014 | 0.011075527 | 0.017843999 | 0.005444722 |
| Deaths | Global | Male | 25-29 years | Colon and rectum cancer | High fasting plasma glucose | Number | 2015 | 43.04733352 | 71.74455963 | 19.86955829 |
| Deaths | Global | Female | 25-29 years | Colon and rectum cancer | High fasting plasma glucose | Number | 2015 | 24.37912955 | 38.41240814 | 11.49220891 |
| Deaths | Global | Both | 25-29 years | Colon and rectum cancer | High fasting plasma glucose | Number | 2015 | 67.42646306 | 108.2272011 | 32.74629926 |
| Deaths | Global | Male | 25-29 years | Colon and rectum cancer | High fasting plasma glucose | Rate | 2015 | 0.014143898 | 0.023572836 | 0.006528465 |
| Deaths | Global | Female | 25-29 years | Colon and rectum cancer | High fasting plasma glucose | Rate | 2015 | 0.008070084 | 0.01271544 | 0.0038042 |
| Deaths | Global | Both | 25-29 years | Colon and rectum cancer | High fasting plasma glucose | Rate | 2015 | 0.011118309 | 0.01784616 | 0.005399712 |
| Deaths | Global | Male | 25-29 years | Colon and rectum cancer | High fasting plasma glucose | Number | 2016 | 43.9379151 | 72.05509576 | 20.62371118 |
| Deaths | Global | Female | 25-29 years | Colon and rectum cancer | High fasting plasma glucose | Number | 2016 | 24.72213189 | 39.36917997 | 12.14044577 |
| Deaths | Global | Both | 25-29 years | Colon and rectum cancer | High fasting plasma glucose | Number | 2016 | 68.66004699 | 108.7546533 | 33.44272951 |
| Deaths | Global | Male | 25-29 years | Colon and rectum cancer | High fasting plasma glucose | Rate | 2016 | 0.014400419 | 0.023615676 | 0.006759312 |
| Deaths | Global | Female | 25-29 years | Colon and rectum cancer | High fasting plasma glucose | Rate | 2016 | 0.00818493 | 0.013034232 | 0.004019423 |
| Deaths | Global | Both | 25-29 years | Colon and rectum cancer | High fasting plasma glucose | Rate | 2016 | 0.011308394 | 0.017912025 | 0.005508059 |
| Deaths | Global | Male | 25-29 years | Colon and rectum cancer | High fasting plasma glucose | Number | 2017 | 44.34531194 | 73.38994374 | 20.18058331 |
| Deaths | Global | Female | 25-29 years | Colon and rectum cancer | High fasting plasma glucose | Number | 2017 | 24.72361281 | 39.92344895 | 11.73876147 |
| Deaths | Global | Both | 25-29 years | Colon and rectum cancer | High fasting plasma glucose | Number | 2017 | 69.06892475 | 110.2877337 | 33.81333923 |
| Deaths | Global | Male | 25-29 years | Colon and rectum cancer | High fasting plasma glucose | Rate | 2017 | 0.014572582 | 0.024117114 | 0.006631664 |
| Deaths | Global | Female | 25-29 years | Colon and rectum cancer | High fasting plasma glucose | Rate | 2017 | 0.008229551 | 0.013289 | 0.003907388 |
| Deaths | Global | Both | 25-29 years | Colon and rectum cancer | High fasting plasma glucose | Rate | 2017 | 0.011421424 | 0.018237478 | 0.005591465 |
| Deaths | Global | Male | 25-29 years | Colon and rectum cancer | High fasting plasma glucose | Number | 2018 | 43.88588833 | 72.35330483 | 20.19476913 |
| Deaths | Global | Female | 25-29 years | Colon and rectum cancer | High fasting plasma glucose | Number | 2018 | 24.4186909 | 39.30284634 | 11.71911604 |
| Deaths | Global | Both | 25-29 years | Colon and rectum cancer | High fasting plasma glucose | Number | 2018 | 68.30457922 | 108.4176226 | 32.93108026 |
| Deaths | Global | Male | 25-29 years | Colon and rectum cancer | High fasting plasma glucose | Rate | 2018 | 0.014504113 | 0.023912481 | 0.006674291 |
| Deaths | Global | Female | 25-29 years | Colon and rectum cancer | High fasting plasma glucose | Rate | 2018 | 0.008196308 | 0.013192282 | 0.003933605 |
| Deaths | Global | Both | 25-29 years | Colon and rectum cancer | High fasting plasma glucose | Rate | 2018 | 0.011374646 | 0.018054603 | 0.005483957 |
| Deaths | Global | Male | 25-29 years | Colon and rectum cancer | High fasting plasma glucose | Number | 2019 | 42.89953244 | 70.80560842 | 19.7899333 |
| Deaths | Global | Female | 25-29 years | Colon and rectum cancer | High fasting plasma glucose | Number | 2019 | 24.17074615 | 38.82860327 | 11.52262808 |
| Deaths | Global | Both | 25-29 years | Colon and rectum cancer | High fasting plasma glucose | Number | 2019 | 67.07027859 | 106.266406 | 32.57583115 |
| Deaths | Global | Male | 25-29 years | Colon and rectum cancer | High fasting plasma glucose | Rate | 2019 | 0.014277488 | 0.023564971 | 0.006586331 |
| Deaths | Global | Female | 25-29 years | Colon and rectum cancer | High fasting plasma glucose | Rate | 2019 | 0.008189575 | 0.013155977 | 0.003904117 |
| Deaths | Global | Both | 25-29 years | Colon and rectum cancer | High fasting plasma glucose | Rate | 2019 | 0.011260768 | 0.017841604 | 0.005469321 |
| Deaths | Global | Male | 25-29 years | Colon and rectum cancer | High fasting plasma glucose | Number | 2020 | 41.85931578 | 68.18619248 | 18.92917663 |
| Deaths | Global | Female | 25-29 years | Colon and rectum cancer | High fasting plasma glucose | Number | 2020 | 23.62225605 | 38.59410507 | 11.40769153 |
| Deaths | Global | Both | 25-29 years | Colon and rectum cancer | High fasting plasma glucose | Number | 2020 | 65.48157183 | 103.1128157 | 31.90358357 |
| Deaths | Global | Male | 25-29 years | Colon and rectum cancer | High fasting plasma glucose | Rate | 2020 | 0.014024584 | 0.022845165 | 0.006342049 |
| Deaths | Global | Female | 25-29 years | Colon and rectum cancer | High fasting plasma glucose | Rate | 2020 | 0.008073753 | 0.013190919 | 0.003898987 |
| Deaths | Global | Both | 25-29 years | Colon and rectum cancer | High fasting plasma glucose | Rate | 2020 | 0.01107882 | 0.017445645 | 0.005397764 |
| Deaths | Global | Male | 25-29 years | Colon and rectum cancer | High fasting plasma glucose | Number | 2021 | 41.44549241 | 67.75308153 | 18.87588066 |
| Deaths | Global | Female | 25-29 years | Colon and rectum cancer | High fasting plasma glucose | Number | 2021 | 23.44542165 | 37.25560015 | 11.73031732 |
| Deaths | Global | Both | 25-29 years | Colon and rectum cancer | High fasting plasma glucose | Number | 2021 | 64.89091406 | 102.2543417 | 31.35392389 |
| Deaths | Global | Male | 25-29 years | Colon and rectum cancer | High fasting plasma glucose | Rate | 2021 | 0.013938007 | 0.022785178 | 0.006347908 |
| Deaths | Global | Female | 25-29 years | Colon and rectum cancer | High fasting plasma glucose | Rate | 2021 | 0.008057198 | 0.012803172 | 0.004031213 |
| Deaths | Global | Both | 25-29 years | Colon and rectum cancer | High fasting plasma glucose | Rate | 2021 | 0.011029432 | 0.017380049 | 0.005329189 |
| Deaths | Global | Male | 30-34 years | Colon and rectum cancer | High fasting plasma glucose | Number | 1990 | 63.67768522 | 103.8839855 | 27.75908335 |
| Deaths | Global | Female | 30-34 years | Colon and rectum cancer | High fasting plasma glucose | Number | 1990 | 42.07423899 | 69.49121929 | 18.94335382 |
| Deaths | Global | Both | 30-34 years | Colon and rectum cancer | High fasting plasma glucose | Number | 1990 | 105.7519242 | 167.6171817 | 47.36419174 |
| Deaths | Global | Male | 30-34 years | Colon and rectum cancer | High fasting plasma glucose | Rate | 1990 | 0.032602817 | 0.053188343 | 0.014212582 |
| Deaths | Global | Female | 30-34 years | Colon and rectum cancer | High fasting plasma glucose | Rate | 1990 | 0.022131635 | 0.036553348 | 0.009964468 |
| Deaths | Global | Both | 30-34 years | Colon and rectum cancer | High fasting plasma glucose | Rate | 1990 | 0.027437923 | 0.043489207 | 0.012288902 |
| Deaths | Global | Male | 30-34 years | Colon and rectum cancer | High fasting plasma glucose | Number | 1991 | 63.72369779 | 105.1312529 | 27.66473726 |
| Deaths | Global | Female | 30-34 years | Colon and rectum cancer | High fasting plasma glucose | Number | 1991 | 41.96672997 | 70.32408947 | 18.36417584 |
| Deaths | Global | Both | 30-34 years | Colon and rectum cancer | High fasting plasma glucose | Number | 1991 | 105.6904278 | 168.8849669 | 46.67831987 |
| Deaths | Global | Male | 30-34 years | Colon and rectum cancer | High fasting plasma glucose | Rate | 1991 | 0.032221391 | 0.053158798 | 0.013988459 |
| Deaths | Global | Female | 30-34 years | Colon and rectum cancer | High fasting plasma glucose | Rate | 1991 | 0.021767763 | 0.036476469 | 0.009525332 |
| Deaths | Global | Both | 30-34 years | Colon and rectum cancer | High fasting plasma glucose | Rate | 1991 | 0.02706116 | 0.043241599 | 0.011951598 |
| Deaths | Global | Male | 30-34 years | Colon and rectum cancer | High fasting plasma glucose | Number | 1992 | 64.15945378 | 102.6352445 | 27.79007921 |
| Deaths | Global | Female | 30-34 years | Colon and rectum cancer | High fasting plasma glucose | Number | 1992 | 42.54839699 | 69.00955867 | 19.05555849 |
| Deaths | Global | Both | 30-34 years | Colon and rectum cancer | High fasting plasma glucose | Number | 1992 | 106.7078508 | 168.1173527 | 47.74382539 |
| Deaths | Global | Male | 30-34 years | Colon and rectum cancer | High fasting plasma glucose | Rate | 1992 | 0.031789199 | 0.050852868 | 0.0137692 |
| Deaths | Global | Female | 30-34 years | Colon and rectum cancer | High fasting plasma glucose | Rate | 1992 | 0.021588609 | 0.035014724 | 0.00966859 |
| Deaths | Global | Both | 30-34 years | Colon and rectum cancer | High fasting plasma glucose | Rate | 1992 | 0.026749514 | 0.042143643 | 0.011968418 |
| Deaths | Global | Male | 30-34 years | Colon and rectum cancer | High fasting plasma glucose | Number | 1993 | 67.10577322 | 107.6155428 | 28.7566865 |
| Deaths | Global | Female | 30-34 years | Colon and rectum cancer | High fasting plasma glucose | Number | 1993 | 43.93379309 | 71.84876398 | 20.01776146 |
| Deaths | Global | Both | 30-34 years | Colon and rectum cancer | High fasting plasma glucose | Number | 1993 | 111.0395663 | 175.9452929 | 49.69048625 |
| Deaths | Global | Male | 30-34 years | Colon and rectum cancer | High fasting plasma glucose | Rate | 1993 | 0.032275265 | 0.051758887 | 0.013830847 |
| Deaths | Global | Female | 30-34 years | Colon and rectum cancer | High fasting plasma glucose | Rate | 1993 | 0.021597446 | 0.035320187 | 0.009840546 |
| Deaths | Global | Both | 30-34 years | Colon and rectum cancer | High fasting plasma glucose | Rate | 1993 | 0.026994708 | 0.042773868 | 0.0120802 |
| Deaths | Global | Male | 30-34 years | Colon and rectum cancer | High fasting plasma glucose | Number | 1994 | 70.30084157 | 114.0128762 | 30.28600324 |
| Deaths | Global | Female | 30-34 years | Colon and rectum cancer | High fasting plasma glucose | Number | 1994 | 46.18516153 | 75.08350554 | 21.21741612 |
| Deaths | Global | Both | 30-34 years | Colon and rectum cancer | High fasting plasma glucose | Number | 1994 | 116.4860031 | 184.3039639 | 52.9661656 |
| Deaths | Global | Male | 30-34 years | Colon and rectum cancer | High fasting plasma glucose | Rate | 1994 | 0.032700784 | 0.053033654 | 0.014087684 |
| Deaths | Global | Female | 30-34 years | Colon and rectum cancer | High fasting plasma glucose | Rate | 1994 | 0.021922971 | 0.035640311 | 0.010071391 |
| Deaths | Global | Both | 30-34 years | Colon and rectum cancer | High fasting plasma glucose | Rate | 1994 | 0.027366469 | 0.043299182 | 0.012443529 |
| Deaths | Global | Male | 30-34 years | Colon and rectum cancer | High fasting plasma glucose | Number | 1995 | 73.39047636 | 118.1939109 | 32.1770996 |
| Deaths | Global | Female | 30-34 years | Colon and rectum cancer | High fasting plasma glucose | Number | 1995 | 48.45756534 | 79.14571527 | 22.31548817 |
| Deaths | Global | Both | 30-34 years | Colon and rectum cancer | High fasting plasma glucose | Number | 1995 | 121.8480417 | 196.5426793 | 54.93199308 |
| Deaths | Global | Male | 30-34 years | Colon and rectum cancer | High fasting plasma glucose | Rate | 1995 | 0.033066539 | 0.053253008 | 0.014497594 |
| Deaths | Global | Female | 30-34 years | Colon and rectum cancer | High fasting plasma glucose | Rate | 1995 | 0.02224876 | 0.036338888 | 0.010245912 |
| Deaths | Global | Both | 30-34 years | Colon and rectum cancer | High fasting plasma glucose | Rate | 1995 | 0.027708681 | 0.04469451 | 0.012491732 |
| Deaths | Global | Male | 30-34 years | Colon and rectum cancer | High fasting plasma glucose | Number | 1996 | 76.67113141 | 125.2158918 | 33.26305713 |
| Deaths | Global | Female | 30-34 years | Colon and rectum cancer | High fasting plasma glucose | Number | 1996 | 50.09540127 | 81.57491197 | 23.46836001 |
| Deaths | Global | Both | 30-34 years | Colon and rectum cancer | High fasting plasma glucose | Number | 1996 | 126.7665327 | 201.0806209 | 57.17601873 |
| Deaths | Global | Male | 30-34 years | Colon and rectum cancer | High fasting plasma glucose | Rate | 1996 | 0.033635445 | 0.054931917 | 0.014592425 |
| Deaths | Global | Female | 30-34 years | Colon and rectum cancer | High fasting plasma glucose | Rate | 1996 | 0.02235748 | 0.036406725 | 0.010473883 |
| Deaths | Global | Both | 30-34 years | Colon and rectum cancer | High fasting plasma glucose | Rate | 1996 | 0.028044891 | 0.04448559 | 0.0126492 |
| Deaths | Global | Male | 30-34 years | Colon and rectum cancer | High fasting plasma glucose | Number | 1997 | 79.86838152 | 131.9754388 | 34.42666048 |
| Deaths | Global | Female | 30-34 years | Colon and rectum cancer | High fasting plasma glucose | Number | 1997 | 51.37902702 | 83.26009646 | 23.86968489 |
| Deaths | Global | Both | 30-34 years | Colon and rectum cancer | High fasting plasma glucose | Number | 1997 | 131.2474085 | 211.1758348 | 59.21140632 |
| Deaths | Global | Male | 30-34 years | Colon and rectum cancer | High fasting plasma glucose | Rate | 1997 | 0.034284598 | 0.056652266 | 0.014778116 |
| Deaths | Global | Female | 30-34 years | Colon and rectum cancer | High fasting plasma glucose | Rate | 1997 | 0.022405161 | 0.03630773 | 0.010408997 |
| Deaths | Global | Both | 30-34 years | Colon and rectum cancer | High fasting plasma glucose | Rate | 1997 | 0.028391639 | 0.045681878 | 0.012808701 |
| Deaths | Global | Male | 30-34 years | Colon and rectum cancer | High fasting plasma glucose | Number | 1998 | 81.26520378 | 134.2665249 | 35.34291476 |
| Deaths | Global | Female | 30-34 years | Colon and rectum cancer | High fasting plasma glucose | Number | 1998 | 52.08296724 | 85.26565512 | 24.55968536 |
| Deaths | Global | Both | 30-34 years | Colon and rectum cancer | High fasting plasma glucose | Number | 1998 | 133.348171 | 211.2067379 | 61.40169578 |
| Deaths | Global | Male | 30-34 years | Colon and rectum cancer | High fasting plasma glucose | Rate | 1998 | 0.034364447 | 0.056777005 | 0.014945385 |
| Deaths | Global | Female | 30-34 years | Colon and rectum cancer | High fasting plasma glucose | Rate | 1998 | 0.022357917 | 0.036602416 | 0.010542859 |
| Deaths | Global | Both | 30-34 years | Colon and rectum cancer | High fasting plasma glucose | Rate | 1998 | 0.028406319 | 0.044992039 | 0.013080016 |
| Deaths | Global | Male | 30-34 years | Colon and rectum cancer | High fasting plasma glucose | Number | 1999 | 83.29207045 | 137.9808539 | 36.52461907 |
| Deaths | Global | Female | 30-34 years | Colon and rectum cancer | High fasting plasma glucose | Number | 1999 | 53.17303569 | 87.58683789 | 24.81730909 |
| Deaths | Global | Both | 30-34 years | Colon and rectum cancer | High fasting plasma glucose | Number | 1999 | 136.4651061 | 218.5547584 | 64.14145031 |
| Deaths | Global | Male | 30-34 years | Colon and rectum cancer | High fasting plasma glucose | Rate | 1999 | 0.034837824 | 0.057712009 | 0.015276823 |
| Deaths | Global | Female | 30-34 years | Colon and rectum cancer | High fasting plasma glucose | Rate | 1999 | 0.022569727 | 0.037176945 | 0.010533908 |
| Deaths | Global | Both | 30-34 years | Colon and rectum cancer | High fasting plasma glucose | Rate | 1999 | 0.028748884 | 0.046042578 | 0.013512576 |
| Deaths | Global | Male | 30-34 years | Colon and rectum cancer | High fasting plasma glucose | Number | 2000 | 86.44277804 | 142.3832884 | 38.52683563 |
| Deaths | Global | Female | 30-34 years | Colon and rectum cancer | High fasting plasma glucose | Number | 2000 | 53.5753539 | 87.27202067 | 25.54410499 |
| Deaths | Global | Both | 30-34 years | Colon and rectum cancer | High fasting plasma glucose | Number | 2000 | 140.0181319 | 219.4134577 | 65.90213468 |
| Deaths | Global | Male | 30-34 years | Colon and rectum cancer | High fasting plasma glucose | Rate | 2000 | 0.035821934 | 0.059003712 | 0.015965542 |
| Deaths | Global | Female | 30-34 years | Colon and rectum cancer | High fasting plasma glucose | Rate | 2000 | 0.022530709 | 0.036701587 | 0.01074238 |
| Deaths | Global | Both | 30-34 years | Colon and rectum cancer | High fasting plasma glucose | Rate | 2000 | 0.029225207 | 0.045796953 | 0.013755387 |
| Deaths | Global | Male | 30-34 years | Colon and rectum cancer | High fasting plasma glucose | Number | 2001 | 87.3016923 | 142.9559784 | 39.37809583 |
| Deaths | Global | Female | 30-34 years | Colon and rectum cancer | High fasting plasma glucose | Number | 2001 | 52.59985452 | 84.68371773 | 25.43113358 |
| Deaths | Global | Both | 30-34 years | Colon and rectum cancer | High fasting plasma glucose | Number | 2001 | 139.9015468 | 222.291779 | 65.1960301 |
| Deaths | Global | Male | 30-34 years | Colon and rectum cancer | High fasting plasma glucose | Rate | 2001 | 0.03579869 | 0.058620133 | 0.016147273 |
| Deaths | Global | Female | 30-34 years | Colon and rectum cancer | High fasting plasma glucose | Rate | 2001 | 0.021900192 | 0.035258456 | 0.01058837 |
| Deaths | Global | Both | 30-34 years | Colon and rectum cancer | High fasting plasma glucose | Rate | 2001 | 0.028902396 | 0.045923473 | 0.013468911 |
| Deaths | Global | Male | 30-34 years | Colon and rectum cancer | High fasting plasma glucose | Number | 2002 | 88.08419929 | 140.882225 | 39.82006148 |
| Deaths | Global | Female | 30-34 years | Colon and rectum cancer | High fasting plasma glucose | Number | 2002 | 52.32021275 | 82.71217768 | 25.39917875 |
| Deaths | Global | Both | 30-34 years | Colon and rectum cancer | High fasting plasma glucose | Number | 2002 | 140.404412 | 223.8234727 | 66.121958 |
| Deaths | Global | Male | 30-34 years | Colon and rectum cancer | High fasting plasma glucose | Rate | 2002 | 0.035796631 | 0.057253276 | 0.016182517 |
| Deaths | Global | Female | 30-34 years | Colon and rectum cancer | High fasting plasma glucose | Rate | 2002 | 0.021583601 | 0.034121165 | 0.010477896 |
| Deaths | Global | Both | 30-34 years | Colon and rectum cancer | High fasting plasma glucose | Rate | 2002 | 0.02874338 | 0.045820804 | 0.013536388 |
| Deaths | Global | Male | 30-34 years | Colon and rectum cancer | High fasting plasma glucose | Number | 2003 | 91.42118755 | 147.9178639 | 40.76417521 |
| Deaths | Global | Female | 30-34 years | Colon and rectum cancer | High fasting plasma glucose | Number | 2003 | 53.29307377 | 83.20094896 | 25.81682991 |
| Deaths | Global | Both | 30-34 years | Colon and rectum cancer | High fasting plasma glucose | Number | 2003 | 144.7142613 | 225.0386474 | 67.51065034 |
| Deaths | Global | Male | 30-34 years | Colon and rectum cancer | High fasting plasma glucose | Rate | 2003 | 0.036887957 | 0.059684062 | 0.016448125 |
| Deaths | Global | Female | 30-34 years | Colon and rectum cancer | High fasting plasma glucose | Rate | 2003 | 0.021797131 | 0.034029601 | 0.010559211 |
| Deaths | Global | Both | 30-34 years | Colon and rectum cancer | High fasting plasma glucose | Rate | 2003 | 0.029393717 | 0.045708849 | 0.013712463 |
| Deaths | Global | Male | 30-34 years | Colon and rectum cancer | High fasting plasma glucose | Number | 2004 | 92.07232808 | 148.0539583 | 43.01523903 |
| Deaths | Global | Female | 30-34 years | Colon and rectum cancer | High fasting plasma glucose | Number | 2004 | 53.30435286 | 82.91065448 | 25.77819897 |
| Deaths | Global | Both | 30-34 years | Colon and rectum cancer | High fasting plasma glucose | Number | 2004 | 145.3766809 | 225.7840656 | 68.48679769 |
| Deaths | Global | Male | 30-34 years | Colon and rectum cancer | High fasting plasma glucose | Rate | 2004 | 0.036971054 | 0.059450119 | 0.017272494 |
| Deaths | Global | Female | 30-34 years | Colon and rectum cancer | High fasting plasma glucose | Rate | 2004 | 0.021648224 | 0.03367208 | 0.010469168 |
| Deaths | Global | Both | 30-34 years | Colon and rectum cancer | High fasting plasma glucose | Rate | 2004 | 0.029353096 | 0.045588201 | 0.013828212 |
| Deaths | Global | Male | 30-34 years | Colon and rectum cancer | High fasting plasma glucose | Number | 2005 | 88.86518121 | 142.3166998 | 39.99870338 |
| Deaths | Global | Female | 30-34 years | Colon and rectum cancer | High fasting plasma glucose | Number | 2005 | 52.07375687 | 81.29965562 | 24.86586908 |
| Deaths | Global | Both | 30-34 years | Colon and rectum cancer | High fasting plasma glucose | Number | 2005 | 140.9389381 | 219.0523981 | 66.62703779 |
| Deaths | Global | Male | 30-34 years | Colon and rectum cancer | High fasting plasma glucose | Rate | 2005 | 0.035596928 | 0.057008125 | 0.016022372 |
| Deaths | Global | Female | 30-34 years | Colon and rectum cancer | High fasting plasma glucose | Rate | 2005 | 0.021045541 | 0.032857151 | 0.010049509 |
| Deaths | Global | Both | 30-34 years | Colon and rectum cancer | High fasting plasma glucose | Rate | 2005 | 0.02835357 | 0.044068145 | 0.013403779 |
| Deaths | Global | Male | 30-34 years | Colon and rectum cancer | High fasting plasma glucose | Number | 2006 | 83.51053385 | 131.8600677 | 37.61120148 |
| Deaths | Global | Female | 30-34 years | Colon and rectum cancer | High fasting plasma glucose | Number | 2006 | 50.2666451 | 78.90873667 | 24.29364886 |
| Deaths | Global | Both | 30-34 years | Colon and rectum cancer | High fasting plasma glucose | Number | 2006 | 133.777179 | 207.4875631 | 64.76049095 |
| Deaths | Global | Male | 30-34 years | Colon and rectum cancer | High fasting plasma glucose | Rate | 2006 | 0.033428725 | 0.05278273 | 0.01505552 |
| Deaths | Global | Female | 30-34 years | Colon and rectum cancer | High fasting plasma glucose | Rate | 2006 | 0.020267927 | 0.031816655 | 0.0097954 |
| Deaths | Global | Both | 30-34 years | Colon and rectum cancer | High fasting plasma glucose | Rate | 2006 | 0.026872197 | 0.041678608 | 0.013008621 |
| Deaths | Global | Male | 30-34 years | Colon and rectum cancer | High fasting plasma glucose | Number | 2007 | 81.61923495 | 125.9281947 | 38.04575505 |
| Deaths | Global | Female | 30-34 years | Colon and rectum cancer | High fasting plasma glucose | Number | 2007 | 49.3405181 | 77.66291614 | 23.81183929 |
| Deaths | Global | Both | 30-34 years | Colon and rectum cancer | High fasting plasma glucose | Number | 2007 | 130.959753 | 201.4921546 | 63.68257371 |
| Deaths | Global | Male | 30-34 years | Colon and rectum cancer | High fasting plasma glucose | Rate | 2007 | 0.032651537 | 0.050377207 | 0.015220093 |
| Deaths | Global | Female | 30-34 years | Colon and rectum cancer | High fasting plasma glucose | Rate | 2007 | 0.019870308 | 0.031276243 | 0.009589453 |
| Deaths | Global | Both | 30-34 years | Colon and rectum cancer | High fasting plasma glucose | Rate | 2007 | 0.026282184 | 0.040437262 | 0.012780393 |
| Deaths | Global | Male | 30-34 years | Colon and rectum cancer | High fasting plasma glucose | Number | 2008 | 83.50136831 | 129.8964254 | 38.87717552 |
| Deaths | Global | Female | 30-34 years | Colon and rectum cancer | High fasting plasma glucose | Number | 2008 | 50.75475909 | 80.0459708 | 24.55565876 |
| Deaths | Global | Both | 30-34 years | Colon and rectum cancer | High fasting plasma glucose | Number | 2008 | 134.2561274 | 205.7813994 | 64.83338536 |
| Deaths | Global | Male | 30-34 years | Colon and rectum cancer | High fasting plasma glucose | Rate | 2008 | 0.033298878 | 0.051800411 | 0.015503534 |
| Deaths | Global | Female | 30-34 years | Colon and rectum cancer | High fasting plasma glucose | Rate | 2008 | 0.020386659 | 0.032152057 | 0.009863269 |
| Deaths | Global | Both | 30-34 years | Colon and rectum cancer | High fasting plasma glucose | Rate | 2008 | 0.026866058 | 0.041179014 | 0.01297384 |
| Deaths | Global | Male | 30-34 years | Colon and rectum cancer | High fasting plasma glucose | Number | 2009 | 89.22621389 | 140.036053 | 42.19266008 |
| Deaths | Global | Female | 30-34 years | Colon and rectum cancer | High fasting plasma glucose | Number | 2009 | 52.31616536 | 81.12393539 | 25.30702161 |
| Deaths | Global | Both | 30-34 years | Colon and rectum cancer | High fasting plasma glucose | Number | 2009 | 141.5423792 | 219.7578405 | 69.69575838 |
| Deaths | Global | Male | 30-34 years | Colon and rectum cancer | High fasting plasma glucose | Rate | 2009 | 0.035325465 | 0.055441539 | 0.016704456 |
| Deaths | Global | Female | 30-34 years | Colon and rectum cancer | High fasting plasma glucose | Rate | 2009 | 0.020883421 | 0.032382827 | 0.010101987 |
| Deaths | Global | Both | 30-34 years | Colon and rectum cancer | High fasting plasma glucose | Rate | 2009 | 0.028134125 | 0.043680872 | 0.013853301 |
| Deaths | Global | Male | 30-34 years | Colon and rectum cancer | High fasting plasma glucose | Number | 2010 | 92.29951928 | 143.3323403 | 43.85539837 |
| Deaths | Global | Female | 30-34 years | Colon and rectum cancer | High fasting plasma glucose | Number | 2010 | 53.36681437 | 83.93432951 | 25.44926732 |
| Deaths | Global | Both | 30-34 years | Colon and rectum cancer | High fasting plasma glucose | Number | 2010 | 145.6663337 | 222.7390376 | 70.05839727 |
| Deaths | Global | Male | 30-34 years | Colon and rectum cancer | High fasting plasma glucose | Rate | 2010 | 0.036159949 | 0.056152949 | 0.017181119 |
| Deaths | Global | Female | 30-34 years | Colon and rectum cancer | High fasting plasma glucose | Rate | 2010 | 0.021092981 | 0.033174647 | 0.010058703 |
| Deaths | Global | Both | 30-34 years | Colon and rectum cancer | High fasting plasma glucose | Rate | 2010 | 0.028659756 | 0.043823759 | 0.013783944 |
| Deaths | Global | Male | 30-34 years | Colon and rectum cancer | High fasting plasma glucose | Number | 2011 | 90.61050546 | 141.5522421 | 42.43500462 |
| Deaths | Global | Female | 30-34 years | Colon and rectum cancer | High fasting plasma glucose | Number | 2011 | 53.84078357 | 85.40642098 | 26.20125647 |
| Deaths | Global | Both | 30-34 years | Colon and rectum cancer | High fasting plasma glucose | Number | 2011 | 144.451289 | 222.0681251 | 70.48951121 |
| Deaths | Global | Male | 30-34 years | Colon and rectum cancer | High fasting plasma glucose | Rate | 2011 | 0.035064418 | 0.054777831 | 0.016421481 |
| Deaths | Global | Female | 30-34 years | Colon and rectum cancer | High fasting plasma glucose | Rate | 2011 | 0.021027184 | 0.033354947 | 0.010232738 |
| Deaths | Global | Both | 30-34 years | Colon and rectum cancer | High fasting plasma glucose | Rate | 2011 | 0.028077974 | 0.043164884 | 0.013701523 |
| Deaths | Global | Male | 30-34 years | Colon and rectum cancer | High fasting plasma glucose | Number | 2012 | 89.39938977 | 141.2449261 | 42.93203974 |
| Deaths | Global | Female | 30-34 years | Colon and rectum cancer | High fasting plasma glucose | Number | 2012 | 54.06243072 | 85.36398865 | 25.90612087 |
| Deaths | Global | Both | 30-34 years | Colon and rectum cancer | High fasting plasma glucose | Number | 2012 | 143.4618205 | 221.2752868 | 70.991259 |
| Deaths | Global | Male | 30-34 years | Colon and rectum cancer | High fasting plasma glucose | Rate | 2012 | 0.034095877 | 0.053869156 | 0.016373776 |
| Deaths | Global | Female | 30-34 years | Colon and rectum cancer | High fasting plasma glucose | Rate | 2012 | 0.020815256 | 0.032867062 | 0.009974441 |
| Deaths | Global | Both | 30-34 years | Colon and rectum cancer | High fasting plasma glucose | Rate | 2012 | 0.027487055 | 0.04239599 | 0.013601811 |
| Deaths | Global | Male | 30-34 years | Colon and rectum cancer | High fasting plasma glucose | Number | 2013 | 92.22907263 | 146.8027771 | 44.49211146 |
| Deaths | Global | Female | 30-34 years | Colon and rectum cancer | High fasting plasma glucose | Number | 2013 | 55.57492017 | 87.76518199 | 26.80928227 |
| Deaths | Global | Both | 30-34 years | Colon and rectum cancer | High fasting plasma glucose | Number | 2013 | 147.8039928 | 228.01272 | 74.33409705 |
| Deaths | Global | Male | 30-34 years | Colon and rectum cancer | High fasting plasma glucose | Rate | 2013 | 0.034576502 | 0.055036078 | 0.016680007 |
| Deaths | Global | Female | 30-34 years | Colon and rectum cancer | High fasting plasma glucose | Rate | 2013 | 0.02104221 | 0.033230339 | 0.01015074 |
| Deaths | Global | Both | 30-34 years | Colon and rectum cancer | High fasting plasma glucose | Rate | 2013 | 0.027842851 | 0.042952319 | 0.014002823 |
| Deaths | Global | Male | 30-34 years | Colon and rectum cancer | High fasting plasma glucose | Number | 2014 | 97.0883995 | 155.2388712 | 46.56422903 |
| Deaths | Global | Female | 30-34 years | Colon and rectum cancer | High fasting plasma glucose | Number | 2014 | 56.24018812 | 88.69652314 | 26.80529086 |
| Deaths | Global | Both | 30-34 years | Colon and rectum cancer | High fasting plasma glucose | Number | 2014 | 153.3285876 | 237.7136017 | 75.42700982 |
| Deaths | Global | Male | 30-34 years | Colon and rectum cancer | High fasting plasma glucose | Rate | 2014 | 0.035700448 | 0.057083002 | 0.017122168 |
| Deaths | Global | Female | 30-34 years | Colon and rectum cancer | High fasting plasma glucose | Rate | 2014 | 0.020896646 | 0.032956146 | 0.009959794 |
| Deaths | Global | Both | 30-34 years | Colon and rectum cancer | High fasting plasma glucose | Rate | 2014 | 0.028337095 | 0.043932531 | 0.013939881 |
| Deaths | Global | Male | 30-34 years | Colon and rectum cancer | High fasting plasma glucose | Number | 2015 | 101.4863412 | 163.6610066 | 48.30200408 |
| Deaths | Global | Female | 30-34 years | Colon and rectum cancer | High fasting plasma glucose | Number | 2015 | 57.41592673 | 89.98540893 | 27.40073147 |
| Deaths | Global | Both | 30-34 years | Colon and rectum cancer | High fasting plasma glucose | Number | 2015 | 158.9022679 | 248.4870725 | 77.79996355 |
| Deaths | Global | Male | 30-34 years | Colon and rectum cancer | High fasting plasma glucose | Rate | 2015 | 0.036525867 | 0.0589031 | 0.017384335 |
| Deaths | Global | Female | 30-34 years | Colon and rectum cancer | High fasting plasma glucose | Rate | 2015 | 0.020893608 | 0.032745616 | 0.009971104 |
| Deaths | Global | Both | 30-34 years | Colon and rectum cancer | High fasting plasma glucose | Rate | 2015 | 0.028752824 | 0.044962889 | 0.014077638 |
| Deaths | Global | Male | 30-34 years | Colon and rectum cancer | High fasting plasma glucose | Number | 2016 | 107.67296 | 173.1607537 | 51.60452806 |
| Deaths | Global | Female | 30-34 years | Colon and rectum cancer | High fasting plasma glucose | Number | 2016 | 60.08261012 | 93.43250918 | 28.91277367 |
| Deaths | Global | Both | 30-34 years | Colon and rectum cancer | High fasting plasma glucose | Number | 2016 | 167.7555701 | 261.8690162 | 81.87465672 |
| Deaths | Global | Male | 30-34 years | Colon and rectum cancer | High fasting plasma glucose | Rate | 2016 | 0.037843998 | 0.060861103 | 0.018137531 |
| Deaths | Global | Female | 30-34 years | Colon and rectum cancer | High fasting plasma glucose | Rate | 2016 | 0.021367583 | 0.033228031 | 0.010282444 |
| Deaths | Global | Both | 30-34 years | Colon and rectum cancer | High fasting plasma glucose | Rate | 2016 | 0.029654315 | 0.04629084 | 0.014473063 |
| Deaths | Global | Male | 30-34 years | Colon and rectum cancer | High fasting plasma glucose | Number | 2017 | 115.262146 | 187.925154 | 54.1711309 |
| Deaths | Global | Female | 30-34 years | Colon and rectum cancer | High fasting plasma glucose | Number | 2017 | 62.76944959 | 99.41819169 | 29.8461186 |
| Deaths | Global | Both | 30-34 years | Colon and rectum cancer | High fasting plasma glucose | Number | 2017 | 178.0315956 | 277.8634194 | 85.71514525 |
| Deaths | Global | Male | 30-34 years | Colon and rectum cancer | High fasting plasma glucose | Rate | 2017 | 0.039567733 | 0.064511833 | 0.018596121 |
| Deaths | Global | Female | 30-34 years | Colon and rectum cancer | High fasting plasma glucose | Rate | 2017 | 0.021831782 | 0.034578546 | 0.01038075 |
| Deaths | Global | Both | 30-34 years | Colon and rectum cancer | High fasting plasma glucose | Rate | 2017 | 0.030757813 | 0.048005361 | 0.014808666 |
| Deaths | Global | Male | 30-34 years | Colon and rectum cancer | High fasting plasma glucose | Number | 2018 | 122.359101 | 195.5245978 | 56.32593254 |
| Deaths | Global | Female | 30-34 years | Colon and rectum cancer | High fasting plasma glucose | Number | 2018 | 64.66717468 | 103.4126595 | 30.74479597 |
| Deaths | Global | Both | 30-34 years | Colon and rectum cancer | High fasting plasma glucose | Number | 2018 | 187.0262757 | 291.4156979 | 90.23223981 |
| Deaths | Global | Male | 30-34 years | Colon and rectum cancer | High fasting plasma glucose | Rate | 2018 | 0.041145574 | 0.065748863 | 0.018940666 |
| Deaths | Global | Female | 30-34 years | Colon and rectum cancer | High fasting plasma glucose | Rate | 2018 | 0.022073715 | 0.035299232 | 0.010494534 |
| Deaths | Global | Both | 30-34 years | Colon and rectum cancer | High fasting plasma glucose | Rate | 2018 | 0.031681056 | 0.049363957 | 0.015284765 |
| Deaths | Global | Male | 30-34 years | Colon and rectum cancer | High fasting plasma glucose | Number | 2019 | 127.4569418 | 208.7491586 | 58.42310832 |
| Deaths | Global | Female | 30-34 years | Colon and rectum cancer | High fasting plasma glucose | Number | 2019 | 65.93758489 | 105.742702 | 30.5953164 |
| Deaths | Global | Both | 30-34 years | Colon and rectum cancer | High fasting plasma glucose | Number | 2019 | 193.3945267 | 305.6127778 | 93.09405964 |
| Deaths | Global | Male | 30-34 years | Colon and rectum cancer | High fasting plasma glucose | Rate | 2019 | 0.04218741 | 0.069094599 | 0.019337665 |
| Deaths | Global | Female | 30-34 years | Colon and rectum cancer | High fasting plasma glucose | Rate | 2019 | 0.022205559 | 0.035610582 | 0.010303473 |
| Deaths | Global | Both | 30-34 years | Colon and rectum cancer | High fasting plasma glucose | Rate | 2019 | 0.032282858 | 0.051015166 | 0.015539955 |
| Deaths | Global | Male | 30-34 years | Colon and rectum cancer | High fasting plasma glucose | Number | 2020 | 129.0597847 | 214.0878362 | 60.05983592 |
| Deaths | Global | Female | 30-34 years | Colon and rectum cancer | High fasting plasma glucose | Number | 2020 | 66.99144529 | 109.1367939 | 32.58891273 |
| Deaths | Global | Both | 30-34 years | Colon and rectum cancer | High fasting plasma glucose | Number | 2020 | 196.05123 | 311.8446404 | 94.32442875 |
| Deaths | Global | Male | 30-34 years | Colon and rectum cancer | High fasting plasma glucose | Rate | 2020 | 0.042313741 | 0.07019117 | 0.019691311 |
| Deaths | Global | Female | 30-34 years | Colon and rectum cancer | High fasting plasma glucose | Rate | 2020 | 0.022403057 | 0.036497165 | 0.010898276 |
| Deaths | Global | Both | 30-34 years | Colon and rectum cancer | High fasting plasma glucose | Rate | 2020 | 0.032456936 | 0.051626921 | 0.015615724 |
| Deaths | Global | Male | 30-34 years | Colon and rectum cancer | High fasting plasma glucose | Number | 2021 | 130.6467789 | 220.4542725 | 60.23967632 |
| Deaths | Global | Female | 30-34 years | Colon and rectum cancer | High fasting plasma glucose | Number | 2021 | 67.86912358 | 108.7587021 | 33.87378767 |
| Deaths | Global | Both | 30-34 years | Colon and rectum cancer | High fasting plasma glucose | Number | 2021 | 198.5159025 | 318.3093517 | 97.68059564 |
| Deaths | Global | Male | 30-34 years | Colon and rectum cancer | High fasting plasma glucose | Rate | 2021 | 0.042758003 | 0.072150148 | 0.019715207 |
| Deaths | Global | Female | 30-34 years | Colon and rectum cancer | High fasting plasma glucose | Rate | 2021 | 0.022703953 | 0.036382559 | 0.011331646 |
| Deaths | Global | Both | 30-34 years | Colon and rectum cancer | High fasting plasma glucose | Rate | 2021 | 0.032840763 | 0.052658361 | 0.016159437 |
| Deaths | Global | Male | 35-39 years | Colon and rectum cancer | High fasting plasma glucose | Number | 1990 | 137.7863286 | 240.8341684 | 61.2330814 |
| Deaths | Global | Female | 35-39 years | Colon and rectum cancer | High fasting plasma glucose | Number | 1990 | 89.96228597 | 147.8206513 | 41.78995055 |
| Deaths | Global | Both | 35-39 years | Colon and rectum cancer | High fasting plasma glucose | Number | 1990 | 227.7486145 | 382.7882877 | 106.3708399 |
| Deaths | Global | Male | 35-39 years | Colon and rectum cancer | High fasting plasma glucose | Rate | 1990 | 0.077065514 | 0.134701383 | 0.034248383 |
| Deaths | Global | Female | 35-39 years | Colon and rectum cancer | High fasting plasma glucose | Rate | 1990 | 0.051865419 | 0.085222157 | 0.024092911 |
| Deaths | Global | Both | 35-39 years | Colon and rectum cancer | High fasting plasma glucose | Rate | 1990 | 0.064656406 | 0.108671198 | 0.030198015 |
| Deaths | Global | Male | 35-39 years | Colon and rectum cancer | High fasting plasma glucose | Number | 1991 | 142.5791443 | 249.1925265 | 62.80718983 |
| Deaths | Global | Female | 35-39 years | Colon and rectum cancer | High fasting plasma glucose | Number | 1991 | 92.01308545 | 149.941576 | 43.48474599 |
| Deaths | Global | Both | 35-39 years | Colon and rectum cancer | High fasting plasma glucose | Number | 1991 | 234.5922298 | 384.7326057 | 108.1813783 |
| Deaths | Global | Male | 35-39 years | Colon and rectum cancer | High fasting plasma glucose | Rate | 1991 | 0.077728563 | 0.135850002 | 0.034240019 |
| Deaths | Global | Female | 35-39 years | Colon and rectum cancer | High fasting plasma glucose | Rate | 1991 | 0.051791842 | 0.084398326 | 0.024476465 |
| Deaths | Global | Both | 35-39 years | Colon and rectum cancer | High fasting plasma glucose | Rate | 1991 | 0.064967525 | 0.106547114 | 0.029959545 |
| Deaths | Global | Male | 35-39 years | Colon and rectum cancer | High fasting plasma glucose | Number | 1992 | 145.3833534 | 254.3608732 | 66.44485693 |
| Deaths | Global | Female | 35-39 years | Colon and rectum cancer | High fasting plasma glucose | Number | 1992 | 93.34028469 | 151.5354231 | 43.04943571 |
| Deaths | Global | Both | 35-39 years | Colon and rectum cancer | High fasting plasma glucose | Number | 1992 | 238.7236381 | 387.9453708 | 109.8333253 |
| Deaths | Global | Male | 35-39 years | Colon and rectum cancer | High fasting plasma glucose | Rate | 1992 | 0.077628031 | 0.13581702 | 0.035478501 |
| Deaths | Global | Female | 35-39 years | Colon and rectum cancer | High fasting plasma glucose | Rate | 1992 | 0.051537111 | 0.083669103 | 0.02376941 |
| Deaths | Global | Both | 35-39 years | Colon and rectum cancer | High fasting plasma glucose | Rate | 1992 | 0.064801035 | 0.105306963 | 0.029814028 |
| Deaths | Global | Male | 35-39 years | Colon and rectum cancer | High fasting plasma glucose | Number | 1993 | 146.4371839 | 255.7149006 | 65.86503533 |
| Deaths | Global | Female | 35-39 years | Colon and rectum cancer | High fasting plasma glucose | Number | 1993 | 93.83723781 | 153.3263877 | 44.71261187 |
| Deaths | Global | Both | 35-39 years | Colon and rectum cancer | High fasting plasma glucose | Number | 1993 | 240.2744217 | 396.7005648 | 110.2852777 |
| Deaths | Global | Male | 35-39 years | Colon and rectum cancer | High fasting plasma glucose | Rate | 1993 | 0.077128244 | 0.134684652 | 0.034691015 |
| Deaths | Global | Female | 35-39 years | Colon and rectum cancer | High fasting plasma glucose | Rate | 1993 | 0.051151991 | 0.083580359 | 0.02437347 |
| Deaths | Global | Both | 35-39 years | Colon and rectum cancer | High fasting plasma glucose | Rate | 1993 | 0.064363275 | 0.106265774 | 0.029542561 |
| Deaths | Global | Male | 35-39 years | Colon and rectum cancer | High fasting plasma glucose | Number | 1994 | 145.2640434 | 238.3926826 | 67.24306699 |
| Deaths | Global | Female | 35-39 years | Colon and rectum cancer | High fasting plasma glucose | Number | 1994 | 93.09582194 | 149.6009933 | 44.58713954 |
| Deaths | Global | Both | 35-39 years | Colon and rectum cancer | High fasting plasma glucose | Number | 1994 | 238.3598654 | 381.7415694 | 113.3381599 |
| Deaths | Global | Male | 35-39 years | Colon and rectum cancer | High fasting plasma glucose | Rate | 1994 | 0.07588171 | 0.124529402 | 0.035125822 |
| Deaths | Global | Female | 35-39 years | Colon and rectum cancer | High fasting plasma glucose | Rate | 1994 | 0.050311509 | 0.080848437 | 0.0240961 |
| Deaths | Global | Both | 35-39 years | Colon and rectum cancer | High fasting plasma glucose | Rate | 1994 | 0.06331382 | 0.101399273 | 0.030105202 |
| Deaths | Global | Male | 35-39 years | Colon and rectum cancer | High fasting plasma glucose | Number | 1995 | 143.2372798 | 238.6318891 | 65.3440623 |
| Deaths | Global | Female | 35-39 years | Colon and rectum cancer | High fasting plasma glucose | Number | 1995 | 92.86586815 | 149.4328577 | 43.45877126 |
| Deaths | Global | Both | 35-39 years | Colon and rectum cancer | High fasting plasma glucose | Number | 1995 | 236.1031479 | 376.2294409 | 111.3208741 |
| Deaths | Global | Male | 35-39 years | Colon and rectum cancer | High fasting plasma glucose | Rate | 1995 | 0.07427064 | 0.12373415 | 0.033881859 |
| Deaths | Global | Female | 35-39 years | Colon and rectum cancer | High fasting plasma glucose | Rate | 1995 | 0.049755661 | 0.080063114 | 0.023284334 |
| Deaths | Global | Both | 35-39 years | Colon and rectum cancer | High fasting plasma glucose | Rate | 1995 | 0.062213879 | 0.099137572 | 0.02933338 |
| Deaths | Global | Male | 35-39 years | Colon and rectum cancer | High fasting plasma glucose | Number | 1996 | 141.992757 | 230.8285622 | 66.25946213 |
| Deaths | Global | Female | 35-39 years | Colon and rectum cancer | High fasting plasma glucose | Number | 1996 | 93.08683144 | 152.9093167 | 44.7082705 |
| Deaths | Global | Both | 35-39 years | Colon and rectum cancer | High fasting plasma glucose | Number | 1996 | 235.0795884 | 378.1617899 | 112.3146875 |
| Deaths | Global | Male | 35-39 years | Colon and rectum cancer | High fasting plasma glucose | Rate | 1996 | 0.07272951 | 0.118231722 | 0.033938479 |
| Deaths | Global | Female | 35-39 years | Colon and rectum cancer | High fasting plasma glucose | Rate | 1996 | 0.049161101 | 0.080754606 | 0.023611372 |
| Deaths | Global | Both | 35-39 years | Colon and rectum cancer | High fasting plasma glucose | Rate | 1996 | 0.061125582 | 0.098329931 | 0.029204155 |
| Deaths | Global | Male | 35-39 years | Colon and rectum cancer | High fasting plasma glucose | Number | 1997 | 144.6135107 | 234.5072335 | 67.5136666 |
| Deaths | Global | Female | 35-39 years | Colon and rectum cancer | High fasting plasma glucose | Number | 1997 | 94.87546508 | 153.6445365 | 44.61027822 |
| Deaths | Global | Both | 35-39 years | Colon and rectum cancer | High fasting plasma glucose | Number | 1997 | 239.4889757 | 379.7307773 | 114.1450859 |
| Deaths | Global | Male | 35-39 years | Colon and rectum cancer | High fasting plasma glucose | Rate | 1997 | 0.072567194 | 0.117675948 | 0.033878421 |
| Deaths | Global | Female | 35-39 years | Colon and rectum cancer | High fasting plasma glucose | Rate | 1997 | 0.04896938 | 0.07930267 | 0.023025317 |
| Deaths | Global | Both | 35-39 years | Colon and rectum cancer | High fasting plasma glucose | Rate | 1997 | 0.060934534 | 0.096617048 | 0.029042579 |
| Deaths | Global | Male | 35-39 years | Colon and rectum cancer | High fasting plasma glucose | Number | 1998 | 150.308465 | 245.5879496 | 72.34355903 |
| Deaths | Global | Female | 35-39 years | Colon and rectum cancer | High fasting plasma glucose | Number | 1998 | 98.28330048 | 155.2570221 | 47.564945 |
| Deaths | Global | Both | 35-39 years | Colon and rectum cancer | High fasting plasma glucose | Number | 1998 | 248.5917655 | 390.267787 | 120.0445328 |
| Deaths | Global | Male | 35-39 years | Colon and rectum cancer | High fasting plasma glucose | Rate | 1998 | 0.073183699 | 0.119574334 | 0.035223361 |
| Deaths | Global | Female | 35-39 years | Colon and rectum cancer | High fasting plasma glucose | Rate | 1998 | 0.049093621 | 0.077552639 | 0.023759228 |
| Deaths | Global | Both | 35-39 years | Colon and rectum cancer | High fasting plasma glucose | Rate | 1998 | 0.061292779 | 0.096224415 | 0.029598177 |
| Deaths | Global | Male | 35-39 years | Colon and rectum cancer | High fasting plasma glucose | Number | 1999 | 159.608517 | 264.8397287 | 75.48855582 |
| Deaths | Global | Female | 35-39 years | Colon and rectum cancer | High fasting plasma glucose | Number | 1999 | 104.0457422 | 165.4004097 | 48.84458639 |
| Deaths | Global | Both | 35-39 years | Colon and rectum cancer | High fasting plasma glucose | Number | 1999 | 263.6542591 | 415.2364924 | 126.6896729 |
| Deaths | Global | Male | 35-39 years | Colon and rectum cancer | High fasting plasma glucose | Rate | 1999 | 0.075116201 | 0.124640932 | 0.035527011 |
| Deaths | Global | Female | 35-39 years | Colon and rectum cancer | High fasting plasma glucose | Rate | 1999 | 0.05011985 | 0.079674992 | 0.023528914 |
| Deaths | Global | Both | 35-39 years | Colon and rectum cancer | High fasting plasma glucose | Rate | 1999 | 0.062763462 | 0.098847938 | 0.030158748 |
| Deaths | Global | Male | 35-39 years | Colon and rectum cancer | High fasting plasma glucose | Number | 2000 | 171.6187101 | 283.2940608 | 81.28449972 |
| Deaths | Global | Female | 35-39 years | Colon and rectum cancer | High fasting plasma glucose | Number | 2000 | 108.5615025 | 176.2609923 | 50.58005708 |
| Deaths | Global | Both | 35-39 years | Colon and rectum cancer | High fasting plasma glucose | Number | 2000 | 280.1802126 | 446.8835843 | 135.5248646 |
| Deaths | Global | Male | 35-39 years | Colon and rectum cancer | High fasting plasma glucose | Rate | 2000 | 0.078195509 | 0.129078719 | 0.037036072 |
| Deaths | Global | Female | 35-39 years | Colon and rectum cancer | High fasting plasma glucose | Rate | 2000 | 0.050521155 | 0.082026396 | 0.023538389 |
| Deaths | Global | Both | 35-39 years | Colon and rectum cancer | High fasting plasma glucose | Rate | 2000 | 0.064504574 | 0.102883907 | 0.031201253 |
| Deaths | Global | Male | 35-39 years | Colon and rectum cancer | High fasting plasma glucose | Number | 2001 | 181.1147546 | 307.5852561 | 88.07796906 |
| Deaths | Global | Female | 35-39 years | Colon and rectum cancer | High fasting plasma glucose | Number | 2001 | 112.7417851 | 179.949488 | 53.94606864 |
| Deaths | Global | Both | 35-39 years | Colon and rectum cancer | High fasting plasma glucose | Number | 2001 | 293.8565397 | 467.1772817 | 141.5300472 |
| Deaths | Global | Male | 35-39 years | Colon and rectum cancer | High fasting plasma glucose | Rate | 2001 | 0.080344724 | 0.136448588 | 0.039072466 |
| Deaths | Global | Female | 35-39 years | Colon and rectum cancer | High fasting plasma glucose | Rate | 2001 | 0.050961783 | 0.081341153 | 0.024384818 |
| Deaths | Global | Both | 35-39 years | Colon and rectum cancer | High fasting plasma glucose | Rate | 2001 | 0.065791205 | 0.104595788 | 0.031687001 |
| Deaths | Global | Male | 35-39 years | Colon and rectum cancer | High fasting plasma glucose | Number | 2002 | 188.7865454 | 317.5443974 | 90.32621581 |
| Deaths | Global | Female | 35-39 years | Colon and rectum cancer | High fasting plasma glucose | Number | 2002 | 115.3409701 | 188.92382 | 55.25002634 |
| Deaths | Global | Both | 35-39 years | Colon and rectum cancer | High fasting plasma glucose | Number | 2002 | 304.1275155 | 491.1822658 | 148.050642 |
| Deaths | Global | Male | 35-39 years | Colon and rectum cancer | High fasting plasma glucose | Rate | 2002 | 0.081927616 | 0.137804606 | 0.039198829 |
| Deaths | Global | Female | 35-39 years | Colon and rectum cancer | High fasting plasma glucose | Rate | 2002 | 0.05091489 | 0.08339652 | 0.024388984 |
| Deaths | Global | Both | 35-39 years | Colon and rectum cancer | High fasting plasma glucose | Rate | 2002 | 0.066553392 | 0.107487301 | 0.03239849 |
| Deaths | Global | Male | 35-39 years | Colon and rectum cancer | High fasting plasma glucose | Number | 2003 | 194.9110515 | 325.1590586 | 92.34311135 |
| Deaths | Global | Female | 35-39 years | Colon and rectum cancer | High fasting plasma glucose | Number | 2003 | 115.5177536 | 188.9264246 | 54.11985457 |
| Deaths | Global | Both | 35-39 years | Colon and rectum cancer | High fasting plasma glucose | Number | 2003 | 310.4288051 | 491.472355 | 151.1315537 |
| Deaths | Global | Male | 35-39 years | Colon and rectum cancer | High fasting plasma glucose | Rate | 2003 | 0.083272192 | 0.138918278 | 0.03945191 |
| Deaths | Global | Female | 35-39 years | Colon and rectum cancer | High fasting plasma glucose | Rate | 2003 | 0.050168599 | 0.0820495 | 0.023503896 |
| Deaths | Global | Both | 35-39 years | Colon and rectum cancer | High fasting plasma glucose | Rate | 2003 | 0.066856065 | 0.105846839 | 0.032548722 |
| Deaths | Global | Male | 35-39 years | Colon and rectum cancer | High fasting plasma glucose | Number | 2004 | 199.9742633 | 329.329 | 96.24314486 |
| Deaths | Global | Female | 35-39 years | Colon and rectum cancer | High fasting plasma glucose | Number | 2004 | 115.3043746 | 185.7636923 | 54.50606062 |
| Deaths | Global | Both | 35-39 years | Colon and rectum cancer | High fasting plasma glucose | Number | 2004 | 315.2786379 | 501.0694955 | 153.9260716 |
| Deaths | Global | Male | 35-39 years | Colon and rectum cancer | High fasting plasma glucose | Rate | 2004 | 0.084404531 | 0.139002187 | 0.040622015 |
| Deaths | Global | Female | 35-39 years | Colon and rectum cancer | High fasting plasma glucose | Rate | 2004 | 0.049480275 | 0.079716304 | 0.023390048 |
| Deaths | Global | Both | 35-39 years | Colon and rectum cancer | High fasting plasma glucose | Rate | 2004 | 0.067087042 | 0.10662083 | 0.032753392 |
| Deaths | Global | Male | 35-39 years | Colon and rectum cancer | High fasting plasma glucose | Number | 2005 | 202.1070404 | 334.4645693 | 94.92458701 |
| Deaths | Global | Female | 35-39 years | Colon and rectum cancer | High fasting plasma glucose | Number | 2005 | 115.3937872 | 184.9357245 | 53.35082354 |
| Deaths | Global | Both | 35-39 years | Colon and rectum cancer | High fasting plasma glucose | Number | 2005 | 317.5008276 | 500.8945136 | 153.5197017 |
| Deaths | Global | Male | 35-39 years | Colon and rectum cancer | High fasting plasma glucose | Rate | 2005 | 0.084394801 | 0.139663966 | 0.039638113 |
| Deaths | Global | Female | 35-39 years | Colon and rectum cancer | High fasting plasma glucose | Rate | 2005 | 0.049028941 | 0.078576177 | 0.022667896 |
| Deaths | Global | Both | 35-39 years | Colon and rectum cancer | High fasting plasma glucose | Rate | 2005 | 0.066865283 | 0.105487768 | 0.03233106 |
| Deaths | Global | Male | 35-39 years | Colon and rectum cancer | High fasting plasma glucose | Number | 2006 | 201.3666979 | 326.6277875 | 99.05140106 |
| Deaths | Global | Female | 35-39 years | Colon and rectum cancer | High fasting plasma glucose | Number | 2006 | 114.916197 | 184.1791491 | 56.28780239 |
| Deaths | Global | Both | 35-39 years | Colon and rectum cancer | High fasting plasma glucose | Number | 2006 | 316.2828949 | 500.8269001 | 157.6790337 |
| Deaths | Global | Male | 35-39 years | Colon and rectum cancer | High fasting plasma glucose | Rate | 2006 | 0.083076053 | 0.134753897 | 0.040864748 |
| Deaths | Global | Female | 35-39 years | Colon and rectum cancer | High fasting plasma glucose | Rate | 2006 | 0.048310469 | 0.077428434 | 0.023663245 |
| Deaths | Global | Both | 35-39 years | Colon and rectum cancer | High fasting plasma glucose | Rate | 2006 | 0.065856796 | 0.104282765 | 0.032832114 |
| Deaths | Global | Male | 35-39 years | Colon and rectum cancer | High fasting plasma glucose | Number | 2007 | 204.5341228 | 331.8462694 | 98.79041835 |
| Deaths | Global | Female | 35-39 years | Colon and rectum cancer | High fasting plasma glucose | Number | 2007 | 114.1712865 | 175.662751 | 55.50562984 |
| Deaths | Global | Both | 35-39 years | Colon and rectum cancer | High fasting plasma glucose | Number | 2007 | 318.7054092 | 501.7386295 | 158.6820246 |
| Deaths | Global | Male | 35-39 years | Colon and rectum cancer | High fasting plasma glucose | Rate | 2007 | 0.083501236 | 0.135476532 | 0.040331275 |
| Deaths | Global | Female | 35-39 years | Colon and rectum cancer | High fasting plasma glucose | Rate | 2007 | 0.047535008 | 0.073136868 | 0.023109668 |
| Deaths | Global | Both | 35-39 years | Colon and rectum cancer | High fasting plasma glucose | Rate | 2007 | 0.06569471 | 0.103423328 | 0.032709108 |
| Deaths | Global | Male | 35-39 years | Colon and rectum cancer | High fasting plasma glucose | Number | 2008 | 208.0667478 | 338.7192536 | 98.76144524 |
| Deaths | Global | Female | 35-39 years | Colon and rectum cancer | High fasting plasma glucose | Number | 2008 | 114.3783206 | 178.2543315 | 56.64247658 |
| Deaths | Global | Both | 35-39 years | Colon and rectum cancer | High fasting plasma glucose | Number | 2008 | 322.4450685 | 502.4497582 | 159.5849436 |
| Deaths | Global | Male | 35-39 years | Colon and rectum cancer | High fasting plasma glucose | Rate | 2008 | 0.084206865 | 0.137083348 | 0.039969826 |
| Deaths | Global | Female | 35-39 years | Colon and rectum cancer | High fasting plasma glucose | Rate | 2008 | 0.047193882 | 0.073549899 | 0.023371373 |
| Deaths | Global | Both | 35-39 years | Colon and rectum cancer | High fasting plasma glucose | Rate | 2008 | 0.065879281 | 0.102656335 | 0.032605062 |
| Deaths | Global | Male | 35-39 years | Colon and rectum cancer | High fasting plasma glucose | Number | 2009 | 207.7842051 | 334.1760753 | 99.89937942 |
| Deaths | Global | Female | 35-39 years | Colon and rectum cancer | High fasting plasma glucose | Number | 2009 | 115.5007137 | 179.6608939 | 56.62645817 |
| Deaths | Global | Both | 35-39 years | Colon and rectum cancer | High fasting plasma glucose | Number | 2009 | 323.2849188 | 509.3872824 | 161.8034056 |
| Deaths | Global | Male | 35-39 years | Colon and rectum cancer | High fasting plasma glucose | Rate | 2009 | 0.083574177 | 0.134411037 | 0.040181151 |
| Deaths | Global | Female | 35-39 years | Colon and rectum cancer | High fasting plasma glucose | Rate | 2009 | 0.047301659 | 0.07357754 | 0.023190553 |
| Deaths | Global | Both | 35-39 years | Colon and rectum cancer | High fasting plasma glucose | Rate | 2009 | 0.06560145 | 0.103365614 | 0.032833384 |
| Deaths | Global | Male | 35-39 years | Colon and rectum cancer | High fasting plasma glucose | Number | 2010 | 206.9358393 | 339.4367763 | 98.51484506 |
| Deaths | Global | Female | 35-39 years | Colon and rectum cancer | High fasting plasma glucose | Number | 2010 | 114.9951265 | 181.0221154 | 57.00109672 |
| Deaths | Global | Both | 35-39 years | Colon and rectum cancer | High fasting plasma glucose | Number | 2010 | 321.9309659 | 510.7010436 | 159.6167668 |
| Deaths | Global | Male | 35-39 years | Colon and rectum cancer | High fasting plasma glucose | Rate | 2010 | 0.082979568 | 0.136111353 | 0.039503642 |
| Deaths | Global | Female | 35-39 years | Colon and rectum cancer | High fasting plasma glucose | Rate | 2010 | 0.046865975 | 0.073775109 | 0.023230654 |
| Deaths | Global | Both | 35-39 years | Colon and rectum cancer | High fasting plasma glucose | Rate | 2010 | 0.065069178 | 0.103223674 | 0.032261984 |
| Deaths | Global | Male | 35-39 years | Colon and rectum cancer | High fasting plasma glucose | Number | 2011 | 198.9492984 | 318.3895838 | 96.73759132 |
| Deaths | Global | Female | 35-39 years | Colon and rectum cancer | High fasting plasma glucose | Number | 2011 | 114.0688222 | 177.1389267 | 56.67008087 |
| Deaths | Global | Both | 35-39 years | Colon and rectum cancer | High fasting plasma glucose | Number | 2011 | 313.0181206 | 491.4657331 | 155.4721192 |
| Deaths | Global | Male | 35-39 years | Colon and rectum cancer | High fasting plasma glucose | Rate | 2011 | 0.079734247 | 0.127603132 | 0.038770174 |
| Deaths | Global | Female | 35-39 years | Colon and rectum cancer | High fasting plasma glucose | Rate | 2011 | 0.04640098 | 0.072056673 | 0.023052288 |
| Deaths | Global | Both | 35-39 years | Colon and rectum cancer | High fasting plasma glucose | Rate | 2011 | 0.063191523 | 0.099216199 | 0.031386426 |
| Deaths | Global | Male | 35-39 years | Colon and rectum cancer | High fasting plasma glucose | Number | 2012 | 191.7482006 | 303.5528917 | 95.11648568 |
| Deaths | Global | Female | 35-39 years | Colon and rectum cancer | High fasting plasma glucose | Number | 2012 | 114.8317375 | 181.5068287 | 56.7083976 |
| Deaths | Global | Both | 35-39 years | Colon and rectum cancer | High fasting plasma glucose | Number | 2012 | 306.5799381 | 476.9737281 | 151.9487104 |
| Deaths | Global | Male | 35-39 years | Colon and rectum cancer | High fasting plasma glucose | Rate | 2012 | 0.076843232 | 0.121649044 | 0.038118001 |
| Deaths | Global | Female | 35-39 years | Colon and rectum cancer | High fasting plasma glucose | Rate | 2012 | 0.046675583 | 0.073776965 | 0.023050226 |
| Deaths | Global | Both | 35-39 years | Colon and rectum cancer | High fasting plasma glucose | Rate | 2012 | 0.061866267 | 0.096250864 | 0.030662474 |
| Deaths | Global | Male | 35-39 years | Colon and rectum cancer | High fasting plasma glucose | Number | 2013 | 192.1688465 | 300.2197348 | 93.22172723 |
| Deaths | Global | Female | 35-39 years | Colon and rectum cancer | High fasting plasma glucose | Number | 2013 | 116.3719629 | 179.5966408 | 58.16077492 |
| Deaths | Global | Both | 35-39 years | Colon and rectum cancer | High fasting plasma glucose | Number | 2013 | 308.5408095 | 475.8595649 | 153.3599948 |
| Deaths | Global | Male | 35-39 years | Colon and rectum cancer | High fasting plasma glucose | Rate | 2013 | 0.076859344 | 0.120075091 | 0.037284715 |
| Deaths | Global | Female | 35-39 years | Colon and rectum cancer | High fasting plasma glucose | Rate | 2013 | 0.0472082 | 0.07285633 | 0.023593875 |
| Deaths | Global | Both | 35-39 years | Colon and rectum cancer | High fasting plasma glucose | Rate | 2013 | 0.062138834 | 0.095836134 | 0.030886064 |
| Deaths | Global | Male | 35-39 years | Colon and rectum cancer | High fasting plasma glucose | Number | 2014 | 199.858258 | 318.5883684 | 98.49642119 |
| Deaths | Global | Female | 35-39 years | Colon and rectum cancer | High fasting plasma glucose | Number | 2014 | 117.5140353 | 179.1958379 | 58.04259368 |
| Deaths | Global | Both | 35-39 years | Colon and rectum cancer | High fasting plasma glucose | Number | 2014 | 317.3722934 | 490.2190408 | 155.7923608 |
| Deaths | Global | Male | 35-39 years | Colon and rectum cancer | High fasting plasma glucose | Rate | 2014 | 0.079473299 | 0.126686127 | 0.039166936 |
| Deaths | Global | Female | 35-39 years | Colon and rectum cancer | High fasting plasma glucose | Rate | 2014 | 0.04741302 | 0.072299583 | 0.023418263 |
| Deaths | Global | Both | 35-39 years | Colon and rectum cancer | High fasting plasma glucose | Rate | 2014 | 0.063559587 | 0.098175299 | 0.03120026 |
| Deaths | Global | Male | 35-39 years | Colon and rectum cancer | High fasting plasma glucose | Number | 2015 | 204.707663 | 322.4440319 | 97.11344767 |
| Deaths | Global | Female | 35-39 years | Colon and rectum cancer | High fasting plasma glucose | Number | 2015 | 119.6542939 | 181.3953122 | 58.9913033 |
| Deaths | Global | Both | 35-39 years | Colon and rectum cancer | High fasting plasma glucose | Number | 2015 | 324.3619569 | 504.8989483 | 159.2153668 |
| Deaths | Global | Male | 35-39 years | Colon and rectum cancer | High fasting plasma glucose | Rate | 2015 | 0.080587788 | 0.126937364 | 0.038230899 |
| Deaths | Global | Female | 35-39 years | Colon and rectum cancer | High fasting plasma glucose | Rate | 2015 | 0.047818024 | 0.072491885 | 0.02357498 |
| Deaths | Global | Both | 35-39 years | Colon and rectum cancer | High fasting plasma glucose | Rate | 2015 | 0.06432605 | 0.10012936 | 0.031574898 |
| Deaths | Global | Male | 35-39 years | Colon and rectum cancer | High fasting plasma glucose | Number | 2016 | 210.0439597 | 327.8743139 | 101.3549107 |
| Deaths | Global | Female | 35-39 years | Colon and rectum cancer | High fasting plasma glucose | Number | 2016 | 123.9518404 | 190.153637 | 61.92796768 |
| Deaths | Global | Both | 35-39 years | Colon and rectum cancer | High fasting plasma glucose | Number | 2016 | 333.9958002 | 519.5810248 | 169.5920254 |
| Deaths | Global | Male | 35-39 years | Colon and rectum cancer | High fasting plasma glucose | Rate | 2016 | 0.081666288 | 0.127479401 | 0.039407367 |
| Deaths | Global | Female | 35-39 years | Colon and rectum cancer | High fasting plasma glucose | Rate | 2016 | 0.048948437 | 0.075091449 | 0.024455282 |
| Deaths | Global | Both | 35-39 years | Colon and rectum cancer | High fasting plasma glucose | Rate | 2016 | 0.06543455 | 0.101793347 | 0.033225501 |
| Deaths | Global | Male | 35-39 years | Colon and rectum cancer | High fasting plasma glucose | Number | 2017 | 221.2123063 | 353.8272278 | 104.8678338 |
| Deaths | Global | Female | 35-39 years | Colon and rectum cancer | High fasting plasma glucose | Number | 2017 | 127.092615 | 192.8418446 | 62.85930556 |
| Deaths | Global | Both | 35-39 years | Colon and rectum cancer | High fasting plasma glucose | Number | 2017 | 348.3049212 | 538.0929637 | 173.8048766 |
| Deaths | Global | Male | 35-39 years | Colon and rectum cancer | High fasting plasma glucose | Rate | 2017 | 0.084766831 | 0.135583835 | 0.040184536 |
| Deaths | Global | Female | 35-39 years | Colon and rectum cancer | High fasting plasma glucose | Rate | 2017 | 0.049487649 | 0.075089253 | 0.024476318 |
| Deaths | Global | Both | 35-39 years | Colon and rectum cancer | High fasting plasma glucose | Rate | 2017 | 0.06726858 | 0.10392259 | 0.033567161 |
| Deaths | Global | Male | 35-39 years | Colon and rectum cancer | High fasting plasma glucose | Number | 2018 | 231.7449284 | 369.8544986 | 106.2625494 |
| Deaths | Global | Female | 35-39 years | Colon and rectum cancer | High fasting plasma glucose | Number | 2018 | 130.1909658 | 201.4091302 | 64.78311543 |
| Deaths | Global | Both | 35-39 years | Colon and rectum cancer | High fasting plasma glucose | Number | 2018 | 361.9358942 | 556.2570801 | 175.2416102 |
| Deaths | Global | Male | 35-39 years | Colon and rectum cancer | High fasting plasma glucose | Rate | 2018 | 0.087296283 | 0.139320947 | 0.040028171 |
| Deaths | Global | Female | 35-39 years | Colon and rectum cancer | High fasting plasma glucose | Rate | 2018 | 0.04986316 | 0.077139728 | 0.024811943 |
| Deaths | Global | Both | 35-39 years | Colon and rectum cancer | High fasting plasma glucose | Rate | 2018 | 0.068735155 | 0.105638643 | 0.03328009 |
| Deaths | Global | Male | 35-39 years | Colon and rectum cancer | High fasting plasma glucose | Number | 2019 | 238.3176243 | 374.9301099 | 110.3045025 |
| Deaths | Global | Female | 35-39 years | Colon and rectum cancer | High fasting plasma glucose | Number | 2019 | 133.4920106 | 204.676088 | 67.84822246 |
| Deaths | Global | Both | 35-39 years | Colon and rectum cancer | High fasting plasma glucose | Number | 2019 | 371.809635 | 574.4560251 | 184.3202976 |
| Deaths | Global | Male | 35-39 years | Colon and rectum cancer | High fasting plasma glucose | Rate | 2019 | 0.088045224 | 0.138516006 | 0.040751433 |
| Deaths | Global | Female | 35-39 years | Colon and rectum cancer | High fasting plasma glucose | Rate | 2019 | 0.050181962 | 0.076941291 | 0.025505323 |
| Deaths | Global | Both | 35-39 years | Colon and rectum cancer | High fasting plasma glucose | Rate | 2019 | 0.069277989 | 0.107036382 | 0.034343756 |
| Deaths | Global | Male | 35-39 years | Colon and rectum cancer | High fasting plasma glucose | Number | 2020 | 243.3094859 | 391.6270552 | 115.2192525 |
| Deaths | Global | Female | 35-39 years | Colon and rectum cancer | High fasting plasma glucose | Number | 2020 | 136.1634342 | 208.62529 | 68.96584685 |
| Deaths | Global | Both | 35-39 years | Colon and rectum cancer | High fasting plasma glucose | Number | 2020 | 379.4729201 | 594.2881405 | 186.1777718 |
| Deaths | Global | Male | 35-39 years | Colon and rectum cancer | High fasting plasma glucose | Rate | 2020 | 0.087989052 | 0.141625771 | 0.041667232 |
| Deaths | Global | Female | 35-39 years | Colon and rectum cancer | High fasting plasma glucose | Rate | 2020 | 0.050141919 | 0.076825856 | 0.025396539 |
| Deaths | Global | Both | 35-39 years | Colon and rectum cancer | High fasting plasma glucose | Rate | 2020 | 0.06923696 | 0.1084312 | 0.033969177 |
| Deaths | Global | Male | 35-39 years | Colon and rectum cancer | High fasting plasma glucose | Number | 2021 | 253.736833 | 403.5412022 | 119.2452196 |
| Deaths | Global | Female | 35-39 years | Colon and rectum cancer | High fasting plasma glucose | Number | 2021 | 140.1833644 | 211.6458121 | 69.29922276 |
| Deaths | Global | Both | 35-39 years | Colon and rectum cancer | High fasting plasma glucose | Number | 2021 | 393.9201973 | 617.2296 | 193.207036 |
| Deaths | Global | Male | 35-39 years | Colon and rectum cancer | High fasting plasma glucose | Rate | 2021 | 0.089639585 | 0.142562141 | 0.042126687 |
| Deaths | Global | Female | 35-39 years | Colon and rectum cancer | High fasting plasma glucose | Rate | 2021 | 0.050461478 | 0.076185648 | 0.024945479 |
| Deaths | Global | Both | 35-39 years | Colon and rectum cancer | High fasting plasma glucose | Rate | 2021 | 0.070234267 | 0.110049367 | 0.034447979 |
| Deaths | Global | Male | 40-44 years | Colon and rectum cancer | High fasting plasma glucose | Number | 1990 | 225.0714977 | 359.9429097 | 109.8639348 |
| Deaths | Global | Female | 40-44 years | Colon and rectum cancer | High fasting plasma glucose | Number | 1990 | 153.8213713 | 244.0963016 | 73.03855598 |
| Deaths | Global | Both | 40-44 years | Colon and rectum cancer | High fasting plasma glucose | Number | 1990 | 378.8928691 | 589.6456235 | 187.4020692 |
| Deaths | Global | Male | 40-44 years | Colon and rectum cancer | High fasting plasma glucose | Rate | 1990 | 0.153888541 | 0.246104415 | 0.075117466 |
| Deaths | Global | Female | 40-44 years | Colon and rectum cancer | High fasting plasma glucose | Rate | 1990 | 0.109695753 | 0.17407417 | 0.052086516 |
| Deaths | Global | Both | 40-44 years | Colon and rectum cancer | High fasting plasma glucose | Rate | 1990 | 0.132257298 | 0.205823184 | 0.065415038 |
| Deaths | Global | Male | 40-44 years | Colon and rectum cancer | High fasting plasma glucose | Number | 1991 | 238.4950794 | 399.1324489 | 118.4000008 |
| Deaths | Global | Female | 40-44 years | Colon and rectum cancer | High fasting plasma glucose | Number | 1991 | 162.5983864 | 252.4659915 | 77.35930877 |
| Deaths | Global | Both | 40-44 years | Colon and rectum cancer | High fasting plasma glucose | Number | 1991 | 401.0934658 | 626.8352218 | 198.3971422 |
| Deaths | Global | Male | 40-44 years | Colon and rectum cancer | High fasting plasma glucose | Rate | 1991 | 0.155553989 | 0.260326732 | 0.077224203 |
| Deaths | Global | Female | 40-44 years | Colon and rectum cancer | High fasting plasma glucose | Rate | 1991 | 0.110453482 | 0.171500766 | 0.052550368 |
| Deaths | Global | Both | 40-44 years | Colon and rectum cancer | High fasting plasma glucose | Rate | 1991 | 0.133462198 | 0.208576837 | 0.066015831 |
| Deaths | Global | Male | 40-44 years | Colon and rectum cancer | High fasting plasma glucose | Number | 1992 | 247.472577 | 395.9553196 | 122.9690221 |
| Deaths | Global | Female | 40-44 years | Colon and rectum cancer | High fasting plasma glucose | Number | 1992 | 168.3470978 | 259.4130003 | 79.87598272 |
| Deaths | Global | Both | 40-44 years | Colon and rectum cancer | High fasting plasma glucose | Number | 1992 | 415.8196748 | 649.427088 | 207.158299 |
| Deaths | Global | Male | 40-44 years | Colon and rectum cancer | High fasting plasma glucose | Rate | 1992 | 0.156233358 | 0.249972865 | 0.077632291 |
| Deaths | Global | Female | 40-44 years | Colon and rectum cancer | High fasting plasma glucose | Rate | 1992 | 0.110530837 | 0.170321535 | 0.052443786 |
| Deaths | Global | Both | 40-44 years | Colon and rectum cancer | High fasting plasma glucose | Rate | 1992 | 0.133830103 | 0.20901583 | 0.066673172 |
| Deaths | Global | Male | 40-44 years | Colon and rectum cancer | High fasting plasma glucose | Number | 1993 | 258.0470645 | 409.386497 | 126.8323317 |
| Deaths | Global | Female | 40-44 years | Colon and rectum cancer | High fasting plasma glucose | Number | 1993 | 176.042281 | 274.4609308 | 85.64812749 |
| Deaths | Global | Both | 40-44 years | Colon and rectum cancer | High fasting plasma glucose | Number | 1993 | 434.0893455 | 679.1259788 | 210.577321 |
| Deaths | Global | Male | 40-44 years | Colon and rectum cancer | High fasting plasma glucose | Rate | 1993 | 0.157436997 | 0.249770642 | 0.077381626 |
| Deaths | Global | Female | 40-44 years | Colon and rectum cancer | High fasting plasma glucose | Rate | 1993 | 0.111446865 | 0.173752636 | 0.054221152 |
| Deaths | Global | Both | 40-44 years | Colon and rectum cancer | High fasting plasma glucose | Rate | 1993 | 0.134866606 | 0.210996692 | 0.065423971 |
| Deaths | Global | Male | 40-44 years | Colon and rectum cancer | High fasting plasma glucose | Number | 1994 | 264.7215633 | 420.1566982 | 133.7348655 |
| Deaths | Global | Female | 40-44 years | Colon and rectum cancer | High fasting plasma glucose | Number | 1994 | 180.5340388 | 278.1659435 | 89.80783699 |
| Deaths | Global | Both | 40-44 years | Colon and rectum cancer | High fasting plasma glucose | Number | 1994 | 445.2556021 | 677.7225477 | 221.7629512 |
| Deaths | Global | Male | 40-44 years | Colon and rectum cancer | High fasting plasma glucose | Rate | 1994 | 0.157294368 | 0.249652054 | 0.079463648 |
| Deaths | Global | Female | 40-44 years | Colon and rectum cancer | High fasting plasma glucose | Rate | 1994 | 0.111082841 | 0.171155885 | 0.055258885 |
| Deaths | Global | Both | 40-44 years | Colon and rectum cancer | High fasting plasma glucose | Rate | 1994 | 0.134591949 | 0.204862102 | 0.067034548 |
| Deaths | Global | Male | 40-44 years | Colon and rectum cancer | High fasting plasma glucose | Number | 1995 | 275.9831441 | 444.5701627 | 136.7322128 |
| Deaths | Global | Female | 40-44 years | Colon and rectum cancer | High fasting plasma glucose | Number | 1995 | 187.9681339 | 290.1272784 | 91.82931407 |
| Deaths | Global | Both | 40-44 years | Colon and rectum cancer | High fasting plasma glucose | Number | 1995 | 463.951278 | 732.3309348 | 229.2230953 |
| Deaths | Global | Male | 40-44 years | Colon and rectum cancer | High fasting plasma glucose | Rate | 1995 | 0.157985615 | 0.254492682 | 0.078271892 |
| Deaths | Global | Female | 40-44 years | Colon and rectum cancer | High fasting plasma glucose | Rate | 1995 | 0.111428283 | 0.171988644 | 0.054436795 |
| Deaths | Global | Both | 40-44 years | Colon and rectum cancer | High fasting plasma glucose | Rate | 1995 | 0.135113639 | 0.213272173 | 0.066755213 |
| Deaths | Global | Male | 40-44 years | Colon and rectum cancer | High fasting plasma glucose | Number | 1996 | 280.7652566 | 449.060659 | 139.1138216 |
| Deaths | Global | Female | 40-44 years | Colon and rectum cancer | High fasting plasma glucose | Number | 1996 | 191.1960128 | 297.7745059 | 95.71428066 |
| Deaths | Global | Both | 40-44 years | Colon and rectum cancer | High fasting plasma glucose | Number | 1996 | 471.9612694 | 737.4095771 | 235.2099149 |
| Deaths | Global | Male | 40-44 years | Colon and rectum cancer | High fasting plasma glucose | Rate | 1996 | 0.157096825 | 0.251263296 | 0.077838476 |
| Deaths | Global | Female | 40-44 years | Colon and rectum cancer | High fasting plasma glucose | Rate | 1996 | 0.110909974 | 0.172734579 | 0.055522436 |
| Deaths | Global | Both | 40-44 years | Colon and rectum cancer | High fasting plasma glucose | Rate | 1996 | 0.134419917 | 0.210022602 | 0.066990449 |
| Deaths | Global | Male | 40-44 years | Colon and rectum cancer | High fasting plasma glucose | Number | 1997 | 286.4520143 | 457.0786877 | 140.1992 |
| Deaths | Global | Female | 40-44 years | Colon and rectum cancer | High fasting plasma glucose | Number | 1997 | 192.8158267 | 296.648205 | 94.28892015 |
| Deaths | Global | Both | 40-44 years | Colon and rectum cancer | High fasting plasma glucose | Number | 1997 | 479.267841 | 739.9284154 | 238.8773117 |
| Deaths | Global | Male | 40-44 years | Colon and rectum cancer | High fasting plasma glucose | Rate | 1997 | 0.15732435 | 0.25103544 | 0.076999801 |
| Deaths | Global | Female | 40-44 years | Colon and rectum cancer | High fasting plasma glucose | Rate | 1997 | 0.109905492 | 0.1690902 | 0.053744914 |
| Deaths | Global | Both | 40-44 years | Colon and rectum cancer | High fasting plasma glucose | Rate | 1997 | 0.134055235 | 0.206964184 | 0.066815988 |
| Deaths | Global | Male | 40-44 years | Colon and rectum cancer | High fasting plasma glucose | Number | 1998 | 289.3086087 | 465.8916429 | 144.7156236 |
| Deaths | Global | Female | 40-44 years | Colon and rectum cancer | High fasting plasma glucose | Number | 1998 | 194.6073584 | 298.9005406 | 97.6233307 |
| Deaths | Global | Both | 40-44 years | Colon and rectum cancer | High fasting plasma glucose | Number | 1998 | 483.9159671 | 738.6015104 | 240.0620364 |
| Deaths | Global | Male | 40-44 years | Colon and rectum cancer | High fasting plasma glucose | Rate | 1998 | 0.156943656 | 0.252736129 | 0.078505093 |
| Deaths | Global | Female | 40-44 years | Colon and rectum cancer | High fasting plasma glucose | Rate | 1998 | 0.109631753 | 0.168385155 | 0.054995951 |
| Deaths | Global | Both | 40-44 years | Colon and rectum cancer | High fasting plasma glucose | Rate | 1998 | 0.13373416 | 0.204118605 | 0.066343119 |
| Deaths | Global | Male | 40-44 years | Colon and rectum cancer | High fasting plasma glucose | Number | 1999 | 291.4562845 | 455.206799 | 146.4039844 |
| Deaths | Global | Female | 40-44 years | Colon and rectum cancer | High fasting plasma glucose | Number | 1999 | 197.0295806 | 303.5601154 | 97.97224781 |
| Deaths | Global | Both | 40-44 years | Colon and rectum cancer | High fasting plasma glucose | Number | 1999 | 488.485865 | 747.9965921 | 244.0619421 |
| Deaths | Global | Male | 40-44 years | Colon and rectum cancer | High fasting plasma glucose | Rate | 1999 | 0.156876972 | 0.245016039 | 0.078802259 |
| Deaths | Global | Female | 40-44 years | Colon and rectum cancer | High fasting plasma glucose | Rate | 1999 | 0.110076781 | 0.169593419 | 0.054735282 |
| Deaths | Global | Both | 40-44 years | Colon and rectum cancer | High fasting plasma glucose | Rate | 1999 | 0.133912681 | 0.205054508 | 0.066906724 |
| Deaths | Global | Male | 40-44 years | Colon and rectum cancer | High fasting plasma glucose | Number | 2000 | 291.926808 | 451.9890277 | 143.2173923 |
| Deaths | Global | Female | 40-44 years | Colon and rectum cancer | High fasting plasma glucose | Number | 2000 | 194.3973033 | 296.9192548 | 95.6919096 |
| Deaths | Global | Both | 40-44 years | Colon and rectum cancer | High fasting plasma glucose | Number | 2000 | 486.3241113 | 750.9076722 | 243.1733999 |
| Deaths | Global | Male | 40-44 years | Colon and rectum cancer | High fasting plasma glucose | Rate | 2000 | 0.155926157 | 0.241419802 | 0.076496358 |
| Deaths | Global | Female | 40-44 years | Colon and rectum cancer | High fasting plasma glucose | Rate | 2000 | 0.107632977 | 0.164396844 | 0.052982242 |
| Deaths | Global | Both | 40-44 years | Colon and rectum cancer | High fasting plasma glucose | Rate | 2000 | 0.132213478 | 0.20414393 | 0.066109823 |
| Deaths | Global | Male | 40-44 years | Colon and rectum cancer | High fasting plasma glucose | Number | 2001 | 293.772786 | 460.517559 | 143.3408084 |
| Deaths | Global | Female | 40-44 years | Colon and rectum cancer | High fasting plasma glucose | Number | 2001 | 194.5714844 | 293.4316756 | 97.91069139 |
| Deaths | Global | Both | 40-44 years | Colon and rectum cancer | High fasting plasma glucose | Number | 2001 | 488.3442704 | 752.7946625 | 247.8147432 |
| Deaths | Global | Male | 40-44 years | Colon and rectum cancer | High fasting plasma glucose | Rate | 2001 | 0.154904476 | 0.242827908 | 0.075582674 |
| Deaths | Global | Female | 40-44 years | Colon and rectum cancer | High fasting plasma glucose | Rate | 2001 | 0.106101718 | 0.160011139 | 0.05339165 |
| Deaths | Global | Both | 40-44 years | Colon and rectum cancer | High fasting plasma glucose | Rate | 2001 | 0.13091296 | 0.201805536 | 0.066432973 |
| Deaths | Global | Male | 40-44 years | Colon and rectum cancer | High fasting plasma glucose | Number | 2002 | 301.6973184 | 465.5476414 | 148.7748275 |
| Deaths | Global | Female | 40-44 years | Colon and rectum cancer | High fasting plasma glucose | Number | 2002 | 197.9142377 | 300.5565316 | 99.4833308 |
| Deaths | Global | Both | 40-44 years | Colon and rectum cancer | High fasting plasma glucose | Number | 2002 | 499.6115561 | 760.9144506 | 250.0500148 |
| Deaths | Global | Male | 40-44 years | Colon and rectum cancer | High fasting plasma glucose | Rate | 2002 | 0.155740052 | 0.240321705 | 0.076799487 |
| Deaths | Global | Female | 40-44 years | Colon and rectum cancer | High fasting plasma glucose | Rate | 2002 | 0.105364705 | 0.160008954 | 0.052962495 |
| Deaths | Global | Both | 40-44 years | Colon and rectum cancer | High fasting plasma glucose | Rate | 2002 | 0.130940614 | 0.199424141 | 0.065534318 |
| Deaths | Global | Male | 40-44 years | Colon and rectum cancer | High fasting plasma glucose | Number | 2003 | 311.5618423 | 478.4814972 | 152.5329247 |
| Deaths | Global | Female | 40-44 years | Colon and rectum cancer | High fasting plasma glucose | Number | 2003 | 202.6841088 | 304.1227936 | 103.6141295 |
| Deaths | Global | Both | 40-44 years | Colon and rectum cancer | High fasting plasma glucose | Number | 2003 | 514.2459511 | 768.7076207 | 260.0746043 |
| Deaths | Global | Male | 40-44 years | Colon and rectum cancer | High fasting plasma glucose | Rate | 2003 | 0.155943419 | 0.239490305 | 0.076346017 |
| Deaths | Global | Female | 40-44 years | Colon and rectum cancer | High fasting plasma glucose | Rate | 2003 | 0.104303699 | 0.156505276 | 0.053321087 |
| Deaths | Global | Both | 40-44 years | Colon and rectum cancer | High fasting plasma glucose | Rate | 2003 | 0.130481951 | 0.195047662 | 0.065989906 |
| Deaths | Global | Male | 40-44 years | Colon and rectum cancer | High fasting plasma glucose | Number | 2004 | 329.8838733 | 514.0545492 | 162.6908767 |
| Deaths | Global | Female | 40-44 years | Colon and rectum cancer | High fasting plasma glucose | Number | 2004 | 209.5612966 | 314.0288387 | 107.8939615 |
| Deaths | Global | Both | 40-44 years | Colon and rectum cancer | High fasting plasma glucose | Number | 2004 | 539.4451698 | 820.1153933 | 272.1570811 |
| Deaths | Global | Male | 40-44 years | Colon and rectum cancer | High fasting plasma glucose | Rate | 2004 | 0.159453862 | 0.248475266 | 0.078638851 |
| Deaths | Global | Female | 40-44 years | Colon and rectum cancer | High fasting plasma glucose | Rate | 2004 | 0.103862992 | 0.155639306 | 0.05347452 |
| Deaths | Global | Both | 40-44 years | Colon and rectum cancer | High fasting plasma glucose | Rate | 2004 | 0.132006442 | 0.200688635 | 0.066598961 |
| Deaths | Global | Male | 40-44 years | Colon and rectum cancer | High fasting plasma glucose | Number | 2005 | 351.2506879 | 545.7155507 | 172.2015972 |
| Deaths | Global | Female | 40-44 years | Colon and rectum cancer | High fasting plasma glucose | Number | 2005 | 218.9890211 | 327.9932112 | 111.4171777 |
| Deaths | Global | Both | 40-44 years | Colon and rectum cancer | High fasting plasma glucose | Number | 2005 | 570.239709 | 859.9336467 | 288.8117368 |
| Deaths | Global | Male | 40-44 years | Colon and rectum cancer | High fasting plasma glucose | Rate | 2005 | 0.164202644 | 0.255111063 | 0.080500789 |
| Deaths | Global | Female | 40-44 years | Colon and rectum cancer | High fasting plasma glucose | Rate | 2005 | 0.104712125 | 0.156833735 | 0.053275408 |
| Deaths | Global | Both | 40-44 years | Colon and rectum cancer | High fasting plasma glucose | Rate | 2005 | 0.134793376 | 0.203271286 | 0.068269376 |
| Deaths | Global | Male | 40-44 years | Colon and rectum cancer | High fasting plasma glucose | Number | 2006 | 374.545378 | 578.6070662 | 185.6089239 |
| Deaths | Global | Female | 40-44 years | Colon and rectum cancer | High fasting plasma glucose | Number | 2006 | 227.8877395 | 346.7717255 | 117.4421259 |
| Deaths | Global | Both | 40-44 years | Colon and rectum cancer | High fasting plasma glucose | Number | 2006 | 602.4331175 | 912.3529419 | 310.5463982 |
| Deaths | Global | Male | 40-44 years | Colon and rectum cancer | High fasting plasma glucose | Rate | 2006 | 0.170311015 | 0.263100715 | 0.08439897 |
| Deaths | Global | Female | 40-44 years | Colon and rectum cancer | High fasting plasma glucose | Rate | 2006 | 0.105715471 | 0.160864891 | 0.054480551 |
| Deaths | Global | Both | 40-44 years | Colon and rectum cancer | High fasting plasma glucose | Rate | 2006 | 0.138335965 | 0.209502467 | 0.071310382 |
| Deaths | Global | Male | 40-44 years | Colon and rectum cancer | High fasting plasma glucose | Number | 2007 | 397.2579395 | 609.3965067 | 195.8684205 |
| Deaths | Global | Female | 40-44 years | Colon and rectum cancer | High fasting plasma glucose | Number | 2007 | 236.8076147 | 357.8828778 | 119.2496449 |
| Deaths | Global | Both | 40-44 years | Colon and rectum cancer | High fasting plasma glucose | Number | 2007 | 634.0655542 | 945.7407368 | 328.4330879 |
| Deaths | Global | Male | 40-44 years | Colon and rectum cancer | High fasting plasma glucose | Rate | 2007 | 0.176526089 | 0.270792277 | 0.087036363 |
| Deaths | Global | Female | 40-44 years | Colon and rectum cancer | High fasting plasma glucose | Rate | 2007 | 0.107147903 | 0.161930603 | 0.053956666 |
| Deaths | Global | Both | 40-44 years | Colon and rectum cancer | High fasting plasma glucose | Rate | 2007 | 0.142150563 | 0.212024731 | 0.073631107 |
| Deaths | Global | Male | 40-44 years | Colon and rectum cancer | High fasting plasma glucose | Number | 2008 | 403.4811706 | 625.9671035 | 197.0387683 |
| Deaths | Global | Female | 40-44 years | Colon and rectum cancer | High fasting plasma glucose | Number | 2008 | 240.3666487 | 363.4186148 | 121.1615833 |
| Deaths | Global | Both | 40-44 years | Colon and rectum cancer | High fasting plasma glucose | Number | 2008 | 643.8478192 | 979.2644595 | 326.1783346 |
| Deaths | Global | Male | 40-44 years | Colon and rectum cancer | High fasting plasma glucose | Rate | 2008 | 0.17630056 | 0.273515493 | 0.086095828 |
| Deaths | Global | Female | 40-44 years | Colon and rectum cancer | High fasting plasma glucose | Rate | 2008 | 0.106878636 | 0.161593491 | 0.053874299 |
| Deaths | Global | Both | 40-44 years | Colon and rectum cancer | High fasting plasma glucose | Rate | 2008 | 0.141892757 | 0.215812696 | 0.071883979 |
| Deaths | Global | Male | 40-44 years | Colon and rectum cancer | High fasting plasma glucose | Number | 2009 | 400.3887746 | 613.0944356 | 197.7865517 |
| Deaths | Global | Female | 40-44 years | Colon and rectum cancer | High fasting plasma glucose | Number | 2009 | 238.2826282 | 360.0917277 | 121.9236934 |
| Deaths | Global | Both | 40-44 years | Colon and rectum cancer | High fasting plasma glucose | Number | 2009 | 638.6714027 | 968.8800745 | 328.297692 |
| Deaths | Global | Male | 40-44 years | Colon and rectum cancer | High fasting plasma glucose | Rate | 2009 | 0.172642294 | 0.264358135 | 0.08528292 |
| Deaths | Global | Female | 40-44 years | Colon and rectum cancer | High fasting plasma glucose | Rate | 2009 | 0.104594083 | 0.158062149 | 0.053518366 |
| Deaths | Global | Both | 40-44 years | Colon and rectum cancer | High fasting plasma glucose | Rate | 2009 | 0.138921739 | 0.210747662 | 0.071410253 |
| Deaths | Global | Male | 40-44 years | Colon and rectum cancer | High fasting plasma glucose | Number | 2010 | 409.4895961 | 626.1181666 | 208.0360203 |
| Deaths | Global | Female | 40-44 years | Colon and rectum cancer | High fasting plasma glucose | Number | 2010 | 238.5891502 | 352.2917507 | 120.6936402 |
| Deaths | Global | Both | 40-44 years | Colon and rectum cancer | High fasting plasma glucose | Number | 2010 | 648.0787463 | 969.8220376 | 328.1323563 |
| Deaths | Global | Male | 40-44 years | Colon and rectum cancer | High fasting plasma glucose | Rate | 2010 | 0.174548342 | 0.26688807 | 0.088677082 |
| Deaths | Global | Female | 40-44 years | Colon and rectum cancer | High fasting plasma glucose | Rate | 2010 | 0.103634064 | 0.153022154 | 0.052424733 |
| Deaths | Global | Both | 40-44 years | Colon and rectum cancer | High fasting plasma glucose | Rate | 2010 | 0.139425072 | 0.208643637 | 0.070593084 |
| Deaths | Global | Male | 40-44 years | Colon and rectum cancer | High fasting plasma glucose | Number | 2011 | 410.7205476 | 633.203097 | 206.0585389 |
| Deaths | Global | Female | 40-44 years | Colon and rectum cancer | High fasting plasma glucose | Number | 2011 | 241.3350625 | 362.2766646 | 123.5590411 |
| Deaths | Global | Both | 40-44 years | Colon and rectum cancer | High fasting plasma glucose | Number | 2011 | 652.0556101 | 979.7694097 | 332.5498465 |
| Deaths | Global | Male | 40-44 years | Colon and rectum cancer | High fasting plasma glucose | Rate | 2011 | 0.172901448 | 0.266560154 | 0.086744673 |
| Deaths | Global | Female | 40-44 years | Colon and rectum cancer | High fasting plasma glucose | Rate | 2011 | 0.103684246 | 0.155644117 | 0.053084396 |
| Deaths | Global | Both | 40-44 years | Colon and rectum cancer | High fasting plasma glucose | Rate | 2011 | 0.138645067 | 0.20832609 | 0.0707093 |
| Deaths | Global | Male | 40-44 years | Colon and rectum cancer | High fasting plasma glucose | Number | 2012 | 407.1181529 | 626.8254398 | 210.7779602 |
| Deaths | Global | Female | 40-44 years | Colon and rectum cancer | High fasting plasma glucose | Number | 2012 | 245.06737 | 366.7277776 | 126.4631497 |
| Deaths | Global | Both | 40-44 years | Colon and rectum cancer | High fasting plasma glucose | Number | 2012 | 652.1855229 | 991.5476395 | 338.6509374 |
| Deaths | Global | Male | 40-44 years | Colon and rectum cancer | High fasting plasma glucose | Rate | 2012 | 0.169557383 | 0.261061514 | 0.087785227 |
| Deaths | Global | Female | 40-44 years | Colon and rectum cancer | High fasting plasma glucose | Rate | 2012 | 0.104249258 | 0.156002403 | 0.053796185 |
| Deaths | Global | Both | 40-44 years | Colon and rectum cancer | High fasting plasma glucose | Rate | 2012 | 0.137248847 | 0.208665733 | 0.071267222 |
| Deaths | Global | Male | 40-44 years | Colon and rectum cancer | High fasting plasma glucose | Number | 2013 | 413.0466875 | 629.4383429 | 209.1570427 |
| Deaths | Global | Female | 40-44 years | Colon and rectum cancer | High fasting plasma glucose | Number | 2013 | 251.6135521 | 374.7630973 | 129.3871228 |
| Deaths | Global | Both | 40-44 years | Colon and rectum cancer | High fasting plasma glucose | Number | 2013 | 664.6602396 | 990.1164984 | 342.1464002 |
| Deaths | Global | Male | 40-44 years | Colon and rectum cancer | High fasting plasma glucose | Rate | 2013 | 0.17055024 | 0.259900064 | 0.086362595 |
| Deaths | Global | Female | 40-44 years | Colon and rectum cancer | High fasting plasma glucose | Rate | 2013 | 0.106069654 | 0.157984305 | 0.05454415 |
| Deaths | Global | Both | 40-44 years | Colon and rectum cancer | High fasting plasma glucose | Rate | 2013 | 0.138644143 | 0.206532369 | 0.071369689 |
| Deaths | Global | Male | 40-44 years | Colon and rectum cancer | High fasting plasma glucose | Number | 2014 | 425.6681305 | 645.1589324 | 208.0094094 |
| Deaths | Global | Female | 40-44 years | Colon and rectum cancer | High fasting plasma glucose | Number | 2014 | 258.0527626 | 393.325874 | 130.2652981 |
| Deaths | Global | Both | 40-44 years | Colon and rectum cancer | High fasting plasma glucose | Number | 2014 | 683.7208931 | 1032.495179 | 348.9186575 |
| Deaths | Global | Male | 40-44 years | Colon and rectum cancer | High fasting plasma glucose | Rate | 2014 | 0.174728876 | 0.264825781 | 0.085384006 |
| Deaths | Global | Female | 40-44 years | Colon and rectum cancer | High fasting plasma glucose | Rate | 2014 | 0.107995364 | 0.16460731 | 0.05451617 |
| Deaths | Global | Both | 40-44 years | Colon and rectum cancer | High fasting plasma glucose | Rate | 2014 | 0.141684912 | 0.213960098 | 0.072305103 |
| Deaths | Global | Male | 40-44 years | Colon and rectum cancer | High fasting plasma glucose | Number | 2015 | 429.9094543 | 654.7464677 | 212.9247424 |
| Deaths | Global | Female | 40-44 years | Colon and rectum cancer | High fasting plasma glucose | Number | 2015 | 262.7930117 | 400.043321 | 132.3429075 |
| Deaths | Global | Both | 40-44 years | Colon and rectum cancer | High fasting plasma glucose | Number | 2015 | 692.702466 | 1033.310731 | 355.8690513 |
| Deaths | Global | Male | 40-44 years | Colon and rectum cancer | High fasting plasma glucose | Rate | 2015 | 0.17592496 | 0.26793141 | 0.087131782 |
| Deaths | Global | Female | 40-44 years | Colon and rectum cancer | High fasting plasma glucose | Rate | 2015 | 0.109455969 | 0.166622122 | 0.05512217 |
| Deaths | Global | Both | 40-44 years | Colon and rectum cancer | High fasting plasma glucose | Rate | 2015 | 0.142984129 | 0.213290759 | 0.073456684 |
| Deaths | Global | Male | 40-44 years | Colon and rectum cancer | High fasting plasma glucose | Number | 2016 | 434.8702076 | 665.2104879 | 221.2338082 |
| Deaths | Global | Female | 40-44 years | Colon and rectum cancer | High fasting plasma glucose | Number | 2016 | 267.654357 | 410.0320308 | 133.0027622 |
| Deaths | Global | Both | 40-44 years | Colon and rectum cancer | High fasting plasma glucose | Number | 2016 | 702.5245646 | 1046.049567 | 357.8172018 |
| Deaths | Global | Male | 40-44 years | Colon and rectum cancer | High fasting plasma glucose | Rate | 2016 | 0.177815018 | 0.271999353 | 0.09046077 |
| Deaths | Global | Female | 40-44 years | Colon and rectum cancer | High fasting plasma glucose | Rate | 2016 | 0.111264651 | 0.170451441 | 0.055289613 |
| Deaths | Global | Both | 40-44 years | Colon and rectum cancer | High fasting plasma glucose | Rate | 2016 | 0.144814665 | 0.215627075 | 0.073758528 |
| Deaths | Global | Male | 40-44 years | Colon and rectum cancer | High fasting plasma glucose | Number | 2017 | 443.7310052 | 676.4572655 | 217.7436953 |
| Deaths | Global | Female | 40-44 years | Colon and rectum cancer | High fasting plasma glucose | Number | 2017 | 272.0747106 | 413.3673808 | 133.7598701 |
| Deaths | Global | Both | 40-44 years | Colon and rectum cancer | High fasting plasma glucose | Number | 2017 | 715.8057159 | 1080.32815 | 354.5213206 |
| Deaths | Global | Male | 40-44 years | Colon and rectum cancer | High fasting plasma glucose | Rate | 2017 | 0.181402122 | 0.27654318 | 0.089016021 |
| Deaths | Global | Female | 40-44 years | Colon and rectum cancer | High fasting plasma glucose | Rate | 2017 | 0.113006165 | 0.171692041 | 0.055557129 |
| Deaths | Global | Both | 40-44 years | Colon and rectum cancer | High fasting plasma glucose | Rate | 2017 | 0.147475464 | 0.222577009 | 0.073041043 |
| Deaths | Global | Male | 40-44 years | Colon and rectum cancer | High fasting plasma glucose | Number | 2018 | 449.6160494 | 681.791622 | 220.5015659 |
| Deaths | Global | Female | 40-44 years | Colon and rectum cancer | High fasting plasma glucose | Number | 2018 | 275.268574 | 416.6930221 | 135.8252946 |
| Deaths | Global | Both | 40-44 years | Colon and rectum cancer | High fasting plasma glucose | Number | 2018 | 724.8846234 | 1097.747949 | 363.0089469 |
| Deaths | Global | Male | 40-44 years | Colon and rectum cancer | High fasting plasma glucose | Rate | 2018 | 0.183430249 | 0.278151119 | 0.089958215 |
| Deaths | Global | Female | 40-44 years | Colon and rectum cancer | High fasting plasma glucose | Rate | 2018 | 0.114092821 | 0.17271017 | 0.056296622 |
| Deaths | Global | Both | 40-44 years | Colon and rectum cancer | High fasting plasma glucose | Rate | 2018 | 0.149035838 | 0.225696312 | 0.074634419 |
| Deaths | Global | Male | 40-44 years | Colon and rectum cancer | High fasting plasma glucose | Number | 2019 | 451.8776792 | 688.0833865 | 217.1885305 |
| Deaths | Global | Female | 40-44 years | Colon and rectum cancer | High fasting plasma glucose | Number | 2019 | 275.5597667 | 421.7623586 | 139.3103081 |
| Deaths | Global | Both | 40-44 years | Colon and rectum cancer | High fasting plasma glucose | Number | 2019 | 727.4374459 | 1108.353293 | 362.1239186 |
| Deaths | Global | Male | 40-44 years | Colon and rectum cancer | High fasting plasma glucose | Rate | 2019 | 0.183250077 | 0.279038642 | 0.088076524 |
| Deaths | Global | Female | 40-44 years | Colon and rectum cancer | High fasting plasma glucose | Rate | 2019 | 0.113559825 | 0.173810786 | 0.05741061 |
| Deaths | Global | Both | 40-44 years | Colon and rectum cancer | High fasting plasma glucose | Rate | 2019 | 0.148685187 | 0.226542802 | 0.074016622 |
| Deaths | Global | Male | 40-44 years | Colon and rectum cancer | High fasting plasma glucose | Number | 2020 | 452.4545329 | 696.1610337 | 224.241566 |
| Deaths | Global | Female | 40-44 years | Colon and rectum cancer | High fasting plasma glucose | Number | 2020 | 277.2505252 | 418.6551752 | 136.8748734 |
| Deaths | Global | Both | 40-44 years | Colon and rectum cancer | High fasting plasma glucose | Number | 2020 | 729.7050581 | 1110.551122 | 360.6192594 |
| Deaths | Global | Male | 40-44 years | Colon and rectum cancer | High fasting plasma glucose | Rate | 2020 | 0.181627049 | 0.279457194 | 0.090016413 |
| Deaths | Global | Female | 40-44 years | Colon and rectum cancer | High fasting plasma glucose | Rate | 2020 | 0.113126403 | 0.170823677 | 0.055848991 |
| Deaths | Global | Both | 40-44 years | Colon and rectum cancer | High fasting plasma glucose | Rate | 2020 | 0.147656137 | 0.224720504 | 0.072971464 |
| Deaths | Global | Male | 40-44 years | Colon and rectum cancer | High fasting plasma glucose | Number | 2021 | 456.1107796 | 702.4869225 | 221.0008197 |
| Deaths | Global | Female | 40-44 years | Colon and rectum cancer | High fasting plasma glucose | Number | 2021 | 282.8298914 | 430.9511292 | 139.3927129 |
| Deaths | Global | Both | 40-44 years | Colon and rectum cancer | High fasting plasma glucose | Number | 2021 | 738.940671 | 1119.140036 | 364.4069878 |
| Deaths | Global | Male | 40-44 years | Colon and rectum cancer | High fasting plasma glucose | Rate | 2021 | 0.180880825 | 0.278586737 | 0.087642766 |
| Deaths | Global | Female | 40-44 years | Colon and rectum cancer | High fasting plasma glucose | Rate | 2021 | 0.114003003 | 0.173707676 | 0.056186381 |
| Deaths | Global | Both | 40-44 years | Colon and rectum cancer | High fasting plasma glucose | Rate | 2021 | 0.147714042 | 0.223715793 | 0.072844859 |
| Deaths | Global | Male | 45-49 years | Colon and rectum cancer | High fasting plasma glucose | Number | 1990 | 398.9181731 | 610.7710635 | 192.2092659 |
| Deaths | Global | Female | 45-49 years | Colon and rectum cancer | High fasting plasma glucose | Number | 1990 | 279.5309219 | 441.8961725 | 135.4008354 |
| Deaths | Global | Both | 45-49 years | Colon and rectum cancer | High fasting plasma glucose | Number | 1990 | 678.449095 | 1042.718466 | 333.3010679 |
| Deaths | Global | Male | 45-49 years | Colon and rectum cancer | High fasting plasma glucose | Rate | 1990 | 0.336938871 | 0.515876503 | 0.162346008 |
| Deaths | Global | Female | 45-49 years | Colon and rectum cancer | High fasting plasma glucose | Rate | 1990 | 0.24563205 | 0.388307176 | 0.1189807 |
| Deaths | Global | Both | 45-49 years | Colon and rectum cancer | High fasting plasma glucose | Rate | 1990 | 0.292188745 | 0.449069211 | 0.143543298 |
| Deaths | Global | Male | 45-49 years | Colon and rectum cancer | High fasting plasma glucose | Number | 1991 | 404.9617779 | 626.0101376 | 194.2227992 |
| Deaths | Global | Female | 45-49 years | Colon and rectum cancer | High fasting plasma glucose | Number | 1991 | 283.9293282 | 449.1848765 | 137.0582018 |
| Deaths | Global | Both | 45-49 years | Colon and rectum cancer | High fasting plasma glucose | Number | 1991 | 688.8911061 | 1056.432608 | 336.2603591 |
| Deaths | Global | Male | 45-49 years | Colon and rectum cancer | High fasting plasma glucose | Rate | 1991 | 0.337353397 | 0.521497727 | 0.161797297 |
| Deaths | Global | Female | 45-49 years | Colon and rectum cancer | High fasting plasma glucose | Rate | 1991 | 0.24615134 | 0.389418945 | 0.118822033 |
| Deaths | Global | Both | 45-49 years | Colon and rectum cancer | High fasting plasma glucose | Rate | 1991 | 0.292661597 | 0.448804248 | 0.142853483 |
| Deaths | Global | Male | 45-49 years | Colon and rectum cancer | High fasting plasma glucose | Number | 1992 | 418.4540236 | 642.3207886 | 201.1284302 |
| Deaths | Global | Female | 45-49 years | Colon and rectum cancer | High fasting plasma glucose | Number | 1992 | 291.5090885 | 453.6834161 | 143.0603904 |
| Deaths | Global | Both | 45-49 years | Colon and rectum cancer | High fasting plasma glucose | Number | 1992 | 709.9631121 | 1082.611208 | 351.2930742 |
| Deaths | Global | Male | 45-49 years | Colon and rectum cancer | High fasting plasma glucose | Rate | 1992 | 0.336787076 | 0.516963222 | 0.161875504 |
| Deaths | Global | Female | 45-49 years | Colon and rectum cancer | High fasting plasma glucose | Rate | 1992 | 0.244262128 | 0.380151704 | 0.119873571 |
| Deaths | Global | Both | 45-49 years | Colon and rectum cancer | High fasting plasma glucose | Rate | 1992 | 0.291456361 | 0.444437067 | 0.144213973 |
| Deaths | Global | Male | 45-49 years | Colon and rectum cancer | High fasting plasma glucose | Number | 1993 | 436.5652593 | 666.7960149 | 210.7139164 |
| Deaths | Global | Female | 45-49 years | Colon and rectum cancer | High fasting plasma glucose | Number | 1993 | 303.2599248 | 469.3287901 | 146.9392038 |
| Deaths | Global | Both | 45-49 years | Colon and rectum cancer | High fasting plasma glucose | Number | 1993 | 739.8251841 | 1131.213065 | 361.2214625 |
| Deaths | Global | Male | 45-49 years | Colon and rectum cancer | High fasting plasma glucose | Rate | 1993 | 0.338891086 | 0.517611561 | 0.163570203 |
| Deaths | Global | Female | 45-49 years | Colon and rectum cancer | High fasting plasma glucose | Rate | 1993 | 0.245094543 | 0.37931133 | 0.118756202 |
| Deaths | Global | Both | 45-49 years | Colon and rectum cancer | High fasting plasma glucose | Rate | 1993 | 0.292937988 | 0.447910245 | 0.143027692 |
| Deaths | Global | Male | 45-49 years | Colon and rectum cancer | High fasting plasma glucose | Number | 1994 | 463.5915814 | 705.8120113 | 229.5881435 |
| Deaths | Global | Female | 45-49 years | Colon and rectum cancer | High fasting plasma glucose | Number | 1994 | 319.5224455 | 494.4296783 | 154.8902036 |
| Deaths | Global | Both | 45-49 years | Colon and rectum cancer | High fasting plasma glucose | Number | 1994 | 783.1140268 | 1188.2359 | 388.5739157 |
| Deaths | Global | Male | 45-49 years | Colon and rectum cancer | High fasting plasma glucose | Rate | 1994 | 0.342521925 | 0.521485072 | 0.169629855 |
| Deaths | Global | Female | 45-49 years | Colon and rectum cancer | High fasting plasma glucose | Rate | 1994 | 0.246005578 | 0.380669528 | 0.119252511 |
| Deaths | Global | Both | 45-49 years | Colon and rectum cancer | High fasting plasma glucose | Rate | 1994 | 0.295257608 | 0.448000773 | 0.146504086 |
| Deaths | Global | Male | 45-49 years | Colon and rectum cancer | High fasting plasma glucose | Number | 1995 | 488.026042 | 748.3883061 | 239.4859513 |
| Deaths | Global | Female | 45-49 years | Colon and rectum cancer | High fasting plasma glucose | Number | 1995 | 335.2025766 | 522.2083317 | 160.4701167 |
| Deaths | Global | Both | 45-49 years | Colon and rectum cancer | High fasting plasma glucose | Number | 1995 | 823.2286187 | 1261.341728 | 408.7549481 |
| Deaths | Global | Male | 45-49 years | Colon and rectum cancer | High fasting plasma glucose | Rate | 1995 | 0.347332064 | 0.532633984 | 0.17044408 |
| Deaths | Global | Female | 45-49 years | Colon and rectum cancer | High fasting plasma glucose | Rate | 1995 | 0.248141478 | 0.386576823 | 0.118791724 |
| Deaths | Global | Both | 45-49 years | Colon and rectum cancer | High fasting plasma glucose | Rate | 1995 | 0.298712469 | 0.457683921 | 0.148318702 |
| Deaths | Global | Male | 45-49 years | Colon and rectum cancer | High fasting plasma glucose | Number | 1996 | 513.6701902 | 777.9685314 | 252.0641575 |
| Deaths | Global | Female | 45-49 years | Colon and rectum cancer | High fasting plasma glucose | Number | 1996 | 352.3760533 | 547.9418474 | 171.3337846 |
| Deaths | Global | Both | 45-49 years | Colon and rectum cancer | High fasting plasma glucose | Number | 1996 | 866.0462435 | 1309.862763 | 431.4924419 |
| Deaths | Global | Male | 45-49 years | Colon and rectum cancer | High fasting plasma glucose | Rate | 1996 | 0.349433392 | 0.529227096 | 0.17147118 |
| Deaths | Global | Female | 45-49 years | Colon and rectum cancer | High fasting plasma glucose | Rate | 1996 | 0.248671653 | 0.386682363 | 0.12091019 |
| Deaths | Global | Both | 45-49 years | Colon and rectum cancer | High fasting plasma glucose | Rate | 1996 | 0.29997698 | 0.453704036 | 0.1494583 |
| Deaths | Global | Male | 45-49 years | Colon and rectum cancer | High fasting plasma glucose | Number | 1997 | 531.9518881 | 817.0886525 | 258.6742055 |
| Deaths | Global | Female | 45-49 years | Colon and rectum cancer | High fasting plasma glucose | Number | 1997 | 365.2046626 | 569.43926 | 180.0294086 |
| Deaths | Global | Both | 45-49 years | Colon and rectum cancer | High fasting plasma glucose | Number | 1997 | 897.1565507 | 1361.580149 | 435.8070031 |
| Deaths | Global | Male | 45-49 years | Colon and rectum cancer | High fasting plasma glucose | Rate | 1997 | 0.350891926 | 0.53897696 | 0.17062951 |
| Deaths | Global | Female | 45-49 years | Colon and rectum cancer | High fasting plasma glucose | Rate | 1997 | 0.249331598 | 0.388766123 | 0.12290922 |
| Deaths | Global | Both | 45-49 years | Colon and rectum cancer | High fasting plasma glucose | Rate | 1997 | 0.30098511 | 0.456793578 | 0.146207948 |
| Deaths | Global | Male | 45-49 years | Colon and rectum cancer | High fasting plasma glucose | Number | 1998 | 551.5333774 | 840.9344822 | 273.2333953 |
| Deaths | Global | Female | 45-49 years | Colon and rectum cancer | High fasting plasma glucose | Number | 1998 | 378.5359326 | 588.0545787 | 185.7556109 |
| Deaths | Global | Both | 45-49 years | Colon and rectum cancer | High fasting plasma glucose | Number | 1998 | 930.0693099 | 1401.701381 | 459.5854589 |
| Deaths | Global | Male | 45-49 years | Colon and rectum cancer | High fasting plasma glucose | Rate | 1998 | 0.352017016 | 0.536727711 | 0.17439163 |
| Deaths | Global | Female | 45-49 years | Colon and rectum cancer | High fasting plasma glucose | Rate | 1998 | 0.249330533 | 0.387334329 | 0.122351781 |
| Deaths | Global | Both | 45-49 years | Colon and rectum cancer | High fasting plasma glucose | Rate | 1998 | 0.30148214 | 0.454361764 | 0.148974712 |
| Deaths | Global | Male | 45-49 years | Colon and rectum cancer | High fasting plasma glucose | Number | 1999 | 570.9605975 | 866.4042893 | 280.5898764 |
| Deaths | Global | Female | 45-49 years | Colon and rectum cancer | High fasting plasma glucose | Number | 1999 | 392.3384034 | 613.063362 | 190.9047898 |
| Deaths | Global | Both | 45-49 years | Colon and rectum cancer | High fasting plasma glucose | Number | 1999 | 963.2990009 | 1440.374111 | 487.6514593 |
| Deaths | Global | Male | 45-49 years | Colon and rectum cancer | High fasting plasma glucose | Rate | 1999 | 0.355208858 | 0.539011763 | 0.174561975 |
| Deaths | Global | Female | 45-49 years | Colon and rectum cancer | High fasting plasma glucose | Rate | 1999 | 0.251256256 | 0.392610063 | 0.122256762 |
| Deaths | Global | Both | 45-49 years | Colon and rectum cancer | High fasting plasma glucose | Rate | 1999 | 0.303985196 | 0.454534268 | 0.153886617 |
| Deaths | Global | Male | 45-49 years | Colon and rectum cancer | High fasting plasma glucose | Number | 2000 | 604.140976 | 899.3453706 | 298.6017182 |
| Deaths | Global | Female | 45-49 years | Colon and rectum cancer | High fasting plasma glucose | Number | 2000 | 405.7320225 | 623.8791901 | 200.7295547 |
| Deaths | Global | Both | 45-49 years | Colon and rectum cancer | High fasting plasma glucose | Number | 2000 | 1009.872998 | 1519.048186 | 500.6203416 |
| Deaths | Global | Male | 45-49 years | Colon and rectum cancer | High fasting plasma glucose | Rate | 2000 | 0.362143861 | 0.539100008 | 0.178992625 |
| Deaths | Global | Female | 45-49 years | Colon and rectum cancer | High fasting plasma glucose | Rate | 2000 | 0.250232002 | 0.384772534 | 0.123798358 |
| Deaths | Global | Both | 45-49 years | Colon and rectum cancer | High fasting plasma glucose | Rate | 2000 | 0.306984176 | 0.461764753 | 0.15218005 |
| Deaths | Global | Male | 45-49 years | Colon and rectum cancer | High fasting plasma glucose | Number | 2001 | 622.4344311 | 949.6690725 | 313.9624113 |
| Deaths | Global | Female | 45-49 years | Colon and rectum cancer | High fasting plasma glucose | Number | 2001 | 413.835387 | 637.8283656 | 205.3631495 |
| Deaths | Global | Both | 45-49 years | Colon and rectum cancer | High fasting plasma glucose | Number | 2001 | 1036.269818 | 1566.615488 | 520.2782911 |
| Deaths | Global | Male | 45-49 years | Colon and rectum cancer | High fasting plasma glucose | Rate | 2001 | 0.364483822 | 0.556105183 | 0.183849437 |
| Deaths | Global | Female | 45-49 years | Colon and rectum cancer | High fasting plasma glucose | Rate | 2001 | 0.249452823 | 0.384471921 | 0.123789359 |
| Deaths | Global | Both | 45-49 years | Colon and rectum cancer | High fasting plasma glucose | Rate | 2001 | 0.307801021 | 0.465328469 | 0.154537155 |
| Deaths | Global | Male | 45-49 years | Colon and rectum cancer | High fasting plasma glucose | Number | 2002 | 636.3068067 | 958.6587226 | 316.1619118 |
| Deaths | Global | Female | 45-49 years | Colon and rectum cancer | High fasting plasma glucose | Number | 2002 | 422.0547314 | 645.1253359 | 210.6713475 |
| Deaths | Global | Both | 45-49 years | Colon and rectum cancer | High fasting plasma glucose | Number | 2002 | 1058.361538 | 1579.136626 | 528.9935983 |
| Deaths | Global | Male | 45-49 years | Colon and rectum cancer | High fasting plasma glucose | Rate | 2002 | 0.365448259 | 0.550583709 | 0.181580362 |
| Deaths | Global | Female | 45-49 years | Colon and rectum cancer | High fasting plasma glucose | Rate | 2002 | 0.249615989 | 0.381546721 | 0.124597435 |
| Deaths | Global | Both | 45-49 years | Colon and rectum cancer | High fasting plasma glucose | Rate | 2002 | 0.308381833 | 0.46012353 | 0.154136379 |
| Deaths | Global | Male | 45-49 years | Colon and rectum cancer | High fasting plasma glucose | Number | 2003 | 648.0192191 | 961.9869677 | 324.704899 |
| Deaths | Global | Female | 45-49 years | Colon and rectum cancer | High fasting plasma glucose | Number | 2003 | 428.2963938 | 655.7177373 | 214.8103988 |
| Deaths | Global | Both | 45-49 years | Colon and rectum cancer | High fasting plasma glucose | Number | 2003 | 1076.315613 | 1598.457432 | 539.5889842 |
| Deaths | Global | Male | 45-49 years | Colon and rectum cancer | High fasting plasma glucose | Rate | 2003 | 0.36724706 | 0.545179641 | 0.184017566 |
| Deaths | Global | Female | 45-49 years | Colon and rectum cancer | High fasting plasma glucose | Rate | 2003 | 0.249918969 | 0.382623583 | 0.125345892 |
| Deaths | Global | Both | 45-49 years | Colon and rectum cancer | High fasting plasma glucose | Rate | 2003 | 0.309439649 | 0.459554893 | 0.155131286 |
| Deaths | Global | Male | 45-49 years | Colon and rectum cancer | High fasting plasma glucose | Number | 2004 | 649.3380413 | 973.9782202 | 330.5185527 |
| Deaths | Global | Female | 45-49 years | Colon and rectum cancer | High fasting plasma glucose | Number | 2004 | 430.0850181 | 650.9510479 | 216.7876713 |
| Deaths | Global | Both | 45-49 years | Colon and rectum cancer | High fasting plasma glucose | Number | 2004 | 1079.423059 | 1612.258752 | 553.0371157 |
| Deaths | Global | Male | 45-49 years | Colon and rectum cancer | High fasting plasma glucose | Rate | 2004 | 0.364690591 | 0.547019688 | 0.185630594 |
| Deaths | Global | Female | 45-49 years | Colon and rectum cancer | High fasting plasma glucose | Rate | 2004 | 0.248433633 | 0.376014339 | 0.125224889 |
| Deaths | Global | Both | 45-49 years | Colon and rectum cancer | High fasting plasma glucose | Rate | 2004 | 0.307378676 | 0.459110036 | 0.157483958 |
| Deaths | Global | Male | 45-49 years | Colon and rectum cancer | High fasting plasma glucose | Number | 2005 | 644.0211173 | 974.8682166 | 326.9136006 |
| Deaths | Global | Female | 45-49 years | Colon and rectum cancer | High fasting plasma glucose | Number | 2005 | 430.8662647 | 649.3927646 | 214.2743367 |
| Deaths | Global | Both | 45-49 years | Colon and rectum cancer | High fasting plasma glucose | Number | 2005 | 1074.887382 | 1595.338042 | 549.8647649 |
| Deaths | Global | Male | 45-49 years | Colon and rectum cancer | High fasting plasma glucose | Rate | 2005 | 0.358505288 | 0.542676943 | 0.181982006 |
| Deaths | Global | Female | 45-49 years | Colon and rectum cancer | High fasting plasma glucose | Rate | 2005 | 0.246242852 | 0.371132157 | 0.122459167 |
| Deaths | Global | Both | 45-49 years | Colon and rectum cancer | High fasting plasma glucose | Rate | 2005 | 0.303112394 | 0.449876649 | 0.155058872 |
| Deaths | Global | Male | 45-49 years | Colon and rectum cancer | High fasting plasma glucose | Number | 2006 | 634.4077451 | 948.254201 | 331.4678914 |
| Deaths | Global | Female | 45-49 years | Colon and rectum cancer | High fasting plasma glucose | Number | 2006 | 424.4207242 | 646.9067713 | 213.5861385 |
| Deaths | Global | Both | 45-49 years | Colon and rectum cancer | High fasting plasma glucose | Number | 2006 | 1058.828469 | 1573.598409 | 543.879142 |
| Deaths | Global | Male | 45-49 years | Colon and rectum cancer | High fasting plasma glucose | Rate | 2006 | 0.348222788 | 0.520491316 | 0.181940833 |
| Deaths | Global | Female | 45-49 years | Colon and rectum cancer | High fasting plasma glucose | Rate | 2006 | 0.238535082 | 0.363577815 | 0.120040762 |
| Deaths | Global | Both | 45-49 years | Colon and rectum cancer | High fasting plasma glucose | Rate | 2006 | 0.294027175 | 0.436974172 | 0.151030362 |
| Deaths | Global | Male | 45-49 years | Colon and rectum cancer | High fasting plasma glucose | Number | 2007 | 646.881548 | 955.1984691 | 332.7838754 |
| Deaths | Global | Female | 45-49 years | Colon and rectum cancer | High fasting plasma glucose | Number | 2007 | 430.9524613 | 647.2068855 | 218.7476757 |
| Deaths | Global | Both | 45-49 years | Colon and rectum cancer | High fasting plasma glucose | Number | 2007 | 1077.834009 | 1593.346954 | 550.7626762 |
| Deaths | Global | Male | 45-49 years | Colon and rectum cancer | High fasting plasma glucose | Rate | 2007 | 0.347122632 | 0.512568349 | 0.178574911 |
| Deaths | Global | Female | 45-49 years | Colon and rectum cancer | High fasting plasma glucose | Rate | 2007 | 0.236109664 | 0.354590852 | 0.119847187 |
| Deaths | Global | Both | 45-49 years | Colon and rectum cancer | High fasting plasma glucose | Rate | 2007 | 0.292192942 | 0.431944743 | 0.149307746 |
| Deaths | Global | Male | 45-49 years | Colon and rectum cancer | High fasting plasma glucose | Number | 2008 | 693.7194572 | 1050.538559 | 353.1257702 |
| Deaths | Global | Female | 45-49 years | Colon and rectum cancer | High fasting plasma glucose | Number | 2008 | 453.120013 | 682.8162892 | 230.0467112 |
| Deaths | Global | Both | 45-49 years | Colon and rectum cancer | High fasting plasma glucose | Number | 2008 | 1146.83947 | 1721.502275 | 597.281781 |
| Deaths | Global | Male | 45-49 years | Colon and rectum cancer | High fasting plasma glucose | Rate | 2008 | 0.360324387 | 0.545659572 | 0.183416834 |
| Deaths | Global | Female | 45-49 years | Colon and rectum cancer | High fasting plasma glucose | Rate | 2008 | 0.239583627 | 0.361033719 | 0.121635381 |
| Deaths | Global | Both | 45-49 years | Colon and rectum cancer | High fasting plasma glucose | Rate | 2008 | 0.300491544 | 0.451063022 | 0.156498036 |
| Deaths | Global | Male | 45-49 years | Colon and rectum cancer | High fasting plasma glucose | Number | 2009 | 755.3591661 | 1161.368648 | 381.8612497 |
| Deaths | Global | Female | 45-49 years | Colon and rectum cancer | High fasting plasma glucose | Number | 2009 | 478.9195758 | 720.025048 | 249.3491384 |
| Deaths | Global | Both | 45-49 years | Colon and rectum cancer | High fasting plasma glucose | Number | 2009 | 1234.278742 | 1865.523526 | 633.8218841 |
| Deaths | Global | Male | 45-49 years | Colon and rectum cancer | High fasting plasma glucose | Rate | 2009 | 0.378244413 | 0.581552754 | 0.191216167 |
| Deaths | Global | Female | 45-49 years | Colon and rectum cancer | High fasting plasma glucose | Rate | 2009 | 0.243503232 | 0.366091585 | 0.126779786 |
| Deaths | Global | Both | 45-49 years | Colon and rectum cancer | High fasting plasma glucose | Rate | 2009 | 0.311387524 | 0.470639842 | 0.159902476 |
| Deaths | Global | Male | 45-49 years | Colon and rectum cancer | High fasting plasma glucose | Number | 2010 | 810.5538485 | 1217.777102 | 413.8220022 |
| Deaths | Global | Female | 45-49 years | Colon and rectum cancer | High fasting plasma glucose | Number | 2010 | 501.0199674 | 757.0634767 | 256.6530368 |
| Deaths | Global | Both | 45-49 years | Colon and rectum cancer | High fasting plasma glucose | Number | 2010 | 1311.573816 | 1937.557882 | 667.2013528 |
| Deaths | Global | Male | 45-49 years | Colon and rectum cancer | High fasting plasma glucose | Rate | 2010 | 0.391971328 | 0.588898207 | 0.200117932 |
| Deaths | Global | Female | 45-49 years | Colon and rectum cancer | High fasting plasma glucose | Rate | 2010 | 0.245460315 | 0.370901464 | 0.12573977 |
| Deaths | Global | Both | 45-49 years | Colon and rectum cancer | High fasting plasma glucose | Rate | 2010 | 0.31919265 | 0.471535973 | 0.162374214 |
| Deaths | Global | Male | 45-49 years | Colon and rectum cancer | High fasting plasma glucose | Number | 2011 | 855.0562443 | 1308.608454 | 426.4682301 |
| Deaths | Global | Female | 45-49 years | Colon and rectum cancer | High fasting plasma glucose | Number | 2011 | 513.4724164 | 774.3911647 | 263.9131833 |
| Deaths | Global | Both | 45-49 years | Colon and rectum cancer | High fasting plasma glucose | Number | 2011 | 1368.528661 | 2067.663192 | 689.7396446 |
| Deaths | Global | Male | 45-49 years | Colon and rectum cancer | High fasting plasma glucose | Rate | 2011 | 0.401787367 | 0.614909661 | 0.200395645 |
| Deaths | Global | Female | 45-49 years | Colon and rectum cancer | High fasting plasma glucose | Rate | 2011 | 0.243851692 | 0.367763856 | 0.125334242 |
| Deaths | Global | Both | 45-49 years | Colon and rectum cancer | High fasting plasma glucose | Rate | 2011 | 0.323238377 | 0.488369819 | 0.162912425 |
| Deaths | Global | Male | 45-49 years | Colon and rectum cancer | High fasting plasma glucose | Number | 2012 | 899.7089515 | 1357.871429 | 469.303694 |
| Deaths | Global | Female | 45-49 years | Colon and rectum cancer | High fasting plasma glucose | Number | 2012 | 521.7861531 | 791.6398544 | 268.4441305 |
| Deaths | Global | Both | 45-49 years | Colon and rectum cancer | High fasting plasma glucose | Number | 2012 | 1421.495105 | 2150.933512 | 727.9465905 |
| Deaths | Global | Male | 45-49 years | Colon and rectum cancer | High fasting plasma glucose | Rate | 2012 | 0.412752549 | 0.622940222 | 0.215298843 |
| Deaths | Global | Female | 45-49 years | Colon and rectum cancer | High fasting plasma glucose | Rate | 2012 | 0.241516934 | 0.366422968 | 0.124253591 |
| Deaths | Global | Both | 45-49 years | Colon and rectum cancer | High fasting plasma glucose | Rate | 2012 | 0.327515945 | 0.495580336 | 0.167720673 |
| Deaths | Global | Male | 45-49 years | Colon and rectum cancer | High fasting plasma glucose | Number | 2013 | 916.9899729 | 1376.172138 | 461.1501025 |
| Deaths | Global | Female | 45-49 years | Colon and rectum cancer | High fasting plasma glucose | Number | 2013 | 533.9733268 | 813.5382099 | 271.6139509 |
| Deaths | Global | Both | 45-49 years | Colon and rectum cancer | High fasting plasma glucose | Number | 2013 | 1450.9633 | 2199.8421 | 727.6270491 |
| Deaths | Global | Male | 45-49 years | Colon and rectum cancer | High fasting plasma glucose | Rate | 2013 | 0.413370391 | 0.62036536 | 0.207882096 |
| Deaths | Global | Female | 45-49 years | Colon and rectum cancer | High fasting plasma glucose | Rate | 2013 | 0.242768398 | 0.369871224 | 0.123487973 |
| Deaths | Global | Both | 45-49 years | Colon and rectum cancer | High fasting plasma glucose | Rate | 2013 | 0.328432541 | 0.497944869 | 0.164701892 |
| Deaths | Global | Male | 45-49 years | Colon and rectum cancer | High fasting plasma glucose | Number | 2014 | 903.8650928 | 1385.944549 | 450.1158312 |
| Deaths | Global | Female | 45-49 years | Colon and rectum cancer | High fasting plasma glucose | Number | 2014 | 538.6750892 | 822.9013131 | 273.4391381 |
| Deaths | Global | Both | 45-49 years | Colon and rectum cancer | High fasting plasma glucose | Number | 2014 | 1442.540182 | 2156.094072 | 721.7390252 |
| Deaths | Global | Male | 45-49 years | Colon and rectum cancer | High fasting plasma glucose | Rate | 2014 | 0.401873757 | 0.616214464 | 0.200129136 |
| Deaths | Global | Female | 45-49 years | Colon and rectum cancer | High fasting plasma glucose | Rate | 2014 | 0.241714788 | 0.369253045 | 0.122697865 |
| Deaths | Global | Both | 45-49 years | Colon and rectum cancer | High fasting plasma glucose | Rate | 2014 | 0.322162155 | 0.481519975 | 0.161185805 |
| Deaths | Global | Male | 45-49 years | Colon and rectum cancer | High fasting plasma glucose | Number | 2015 | 912.3045753 | 1417.775459 | 454.0977866 |
| Deaths | Global | Female | 45-49 years | Colon and rectum cancer | High fasting plasma glucose | Number | 2015 | 545.5850709 | 829.6475407 | 281.1836123 |
| Deaths | Global | Both | 45-49 years | Colon and rectum cancer | High fasting plasma glucose | Number | 2015 | 1457.889646 | 2195.257057 | 731.4140453 |
| Deaths | Global | Male | 45-49 years | Colon and rectum cancer | High fasting plasma glucose | Rate | 2015 | 0.400714644 | 0.622734341 | 0.199454917 |
| Deaths | Global | Female | 45-49 years | Colon and rectum cancer | High fasting plasma glucose | Rate | 2015 | 0.242194147 | 0.368294128 | 0.12482201 |
| Deaths | Global | Both | 45-49 years | Colon and rectum cancer | High fasting plasma glucose | Rate | 2015 | 0.321874679 | 0.484671568 | 0.161482497 |
| Deaths | Global | Male | 45-49 years | Colon and rectum cancer | High fasting plasma glucose | Number | 2016 | 933.2334438 | 1394.691455 | 463.8745667 |
| Deaths | Global | Female | 45-49 years | Colon and rectum cancer | High fasting plasma glucose | Number | 2016 | 560.9973696 | 857.6115766 | 285.6909305 |
| Deaths | Global | Both | 45-49 years | Colon and rectum cancer | High fasting plasma glucose | Number | 2016 | 1494.230813 | 2224.769856 | 775.6969778 |
| Deaths | Global | Male | 45-49 years | Colon and rectum cancer | High fasting plasma glucose | Rate | 2016 | 0.404430293 | 0.604409837 | 0.201026793 |
| Deaths | Global | Female | 45-49 years | Colon and rectum cancer | High fasting plasma glucose | Rate | 2016 | 0.246204745 | 0.376379731 | 0.125381092 |
| Deaths | Global | Both | 45-49 years | Colon and rectum cancer | High fasting plasma glucose | Rate | 2016 | 0.325816842 | 0.485110789 | 0.169140629 |
| Deaths | Global | Male | 45-49 years | Colon and rectum cancer | High fasting plasma glucose | Number | 2017 | 939.1712978 | 1447.147994 | 464.974168 |
| Deaths | Global | Female | 45-49 years | Colon and rectum cancer | High fasting plasma glucose | Number | 2017 | 578.0562939 | 866.4712202 | 293.4339281 |
| Deaths | Global | Both | 45-49 years | Colon and rectum cancer | High fasting plasma glucose | Number | 2017 | 1517.227592 | 2285.508861 | 761.4292948 |
| Deaths | Global | Male | 45-49 years | Colon and rectum cancer | High fasting plasma glucose | Rate | 2017 | 0.402347458 | 0.619968176 | 0.199198139 |
| Deaths | Global | Female | 45-49 years | Colon and rectum cancer | High fasting plasma glucose | Rate | 2017 | 0.251101689 | 0.376386157 | 0.127464671 |
| Deaths | Global | Both | 45-49 years | Colon and rectum cancer | High fasting plasma glucose | Rate | 2017 | 0.327248956 | 0.4929586 | 0.16423175 |
| Deaths | Global | Male | 45-49 years | Colon and rectum cancer | High fasting plasma glucose | Number | 2018 | 945.0707355 | 1412.78703 | 462.384089 |
| Deaths | Global | Female | 45-49 years | Colon and rectum cancer | High fasting plasma glucose | Number | 2018 | 595.3266442 | 889.9393413 | 301.0290968 |
| Deaths | Global | Both | 45-49 years | Colon and rectum cancer | High fasting plasma glucose | Number | 2018 | 1540.39738 | 2324.870675 | 802.9331555 |
| Deaths | Global | Male | 45-49 years | Colon and rectum cancer | High fasting plasma glucose | Rate | 2018 | 0.401142926 | 0.599668894 | 0.196262671 |
| Deaths | Global | Female | 45-49 years | Colon and rectum cancer | High fasting plasma glucose | Rate | 2018 | 0.256201002 | 0.382988655 | 0.129548974 |
| Deaths | Global | Both | 45-49 years | Colon and rectum cancer | High fasting plasma glucose | Rate | 2018 | 0.32917179 | 0.496808065 | 0.171581014 |
| Deaths | Global | Male | 45-49 years | Colon and rectum cancer | High fasting plasma glucose | Number | 2019 | 959.0720691 | 1476.874499 | 483.8746163 |
| Deaths | Global | Female | 45-49 years | Colon and rectum cancer | High fasting plasma glucose | Number | 2019 | 605.0348677 | 911.9017999 | 308.7651575 |
| Deaths | Global | Both | 45-49 years | Colon and rectum cancer | High fasting plasma glucose | Number | 2019 | 1564.106937 | 2352.493004 | 810.4037144 |
| Deaths | Global | Male | 45-49 years | Colon and rectum cancer | High fasting plasma glucose | Rate | 2019 | 0.404483601 | 0.622864053 | 0.204071575 |
| Deaths | Global | Female | 45-49 years | Colon and rectum cancer | High fasting plasma glucose | Rate | 2019 | 0.25842937 | 0.389501862 | 0.131883284 |
| Deaths | Global | Both | 45-49 years | Colon and rectum cancer | High fasting plasma glucose | Rate | 2019 | 0.331919886 | 0.499223673 | 0.171976162 |
| Deaths | Global | Male | 45-49 years | Colon and rectum cancer | High fasting plasma glucose | Number | 2020 | 954.5956768 | 1486.787392 | 481.2200459 |
| Deaths | Global | Female | 45-49 years | Colon and rectum cancer | High fasting plasma glucose | Number | 2020 | 608.411995 | 938.1456115 | 309.6195504 |
| Deaths | Global | Both | 45-49 years | Colon and rectum cancer | High fasting plasma glucose | Number | 2020 | 1563.007672 | 2365.619904 | 813.3707785 |
| Deaths | Global | Male | 45-49 years | Colon and rectum cancer | High fasting plasma glucose | Rate | 2020 | 0.401355808 | 0.625113615 | 0.20232698 |
| Deaths | Global | Female | 45-49 years | Colon and rectum cancer | High fasting plasma glucose | Rate | 2020 | 0.258631649 | 0.398799084 | 0.131617087 |
| Deaths | Global | Both | 45-49 years | Colon and rectum cancer | High fasting plasma glucose | Rate | 2020 | 0.330385934 | 0.500040758 | 0.171928948 |
| Deaths | Global | Male | 45-49 years | Colon and rectum cancer | High fasting plasma glucose | Number | 2021 | 944.3286819 | 1426.12181 | 470.5719463 |
| Deaths | Global | Female | 45-49 years | Colon and rectum cancer | High fasting plasma glucose | Number | 2021 | 612.1971791 | 931.2470386 | 312.3844334 |
| Deaths | Global | Both | 45-49 years | Colon and rectum cancer | High fasting plasma glucose | Number | 2021 | 1556.525861 | 2357.075689 | 797.0341772 |
| Deaths | Global | Male | 45-49 years | Colon and rectum cancer | High fasting plasma glucose | Rate | 2021 | 0.397006872 | 0.599558363 | 0.197833975 |
| Deaths | Global | Female | 45-49 years | Colon and rectum cancer | High fasting plasma glucose | Rate | 2021 | 0.259799052 | 0.395194728 | 0.132567059 |
| Deaths | Global | Both | 45-49 years | Colon and rectum cancer | High fasting plasma glucose | Rate | 2021 | 0.328724531 | 0.497793593 | 0.168326587 |
| Deaths | Global | Male | 50-54 years | Colon and rectum cancer | High fasting plasma glucose | Number | 1990 | 789.2200602 | 1230.630002 | 385.7576205 |
| Deaths | Global | Female | 50-54 years | Colon and rectum cancer | High fasting plasma glucose | Number | 1990 | 577.7547958 | 893.0790315 | 282.5830798 |
| Deaths | Global | Both | 50-54 years | Colon and rectum cancer | High fasting plasma glucose | Number | 1990 | 1366.974856 | 2091.490696 | 665.8205335 |
| Deaths | Global | Male | 50-54 years | Colon and rectum cancer | High fasting plasma glucose | Rate | 1990 | 0.733103485 | 1.143127485 | 0.358328773 |
| Deaths | Global | Female | 50-54 years | Colon and rectum cancer | High fasting plasma glucose | Rate | 1990 | 0.55067897 | 0.851225892 | 0.269340143 |
| Deaths | Global | Both | 50-54 years | Colon and rectum cancer | High fasting plasma glucose | Rate | 1990 | 0.643066012 | 0.983900015 | 0.313221968 |
| Deaths | Global | Male | 50-54 years | Colon and rectum cancer | High fasting plasma glucose | Number | 1991 | 796.4572003 | 1274.995551 | 384.0954861 |
| Deaths | Global | Female | 50-54 years | Colon and rectum cancer | High fasting plasma glucose | Number | 1991 | 582.9961748 | 896.1502349 | 284.7266612 |
| Deaths | Global | Both | 50-54 years | Colon and rectum cancer | High fasting plasma glucose | Number | 1991 | 1379.453375 | 2134.881503 | 680.6620519 |
| Deaths | Global | Male | 50-54 years | Colon and rectum cancer | High fasting plasma glucose | Rate | 1991 | 0.731919963 | 1.171682164 | 0.352972079 |
| Deaths | Global | Female | 50-54 years | Colon and rectum cancer | High fasting plasma glucose | Rate | 1991 | 0.549667472 | 0.844919153 | 0.268449419 |
| Deaths | Global | Both | 50-54 years | Colon and rectum cancer | High fasting plasma glucose | Rate | 1991 | 0.641961669 | 0.993518242 | 0.316762389 |
| Deaths | Global | Male | 50-54 years | Colon and rectum cancer | High fasting plasma glucose | Number | 1992 | 803.8953498 | 1239.932757 | 390.1084462 |
| Deaths | Global | Female | 50-54 years | Colon and rectum cancer | High fasting plasma glucose | Number | 1992 | 585.5718308 | 885.4019869 | 291.709705 |
| Deaths | Global | Both | 50-54 years | Colon and rectum cancer | High fasting plasma glucose | Number | 1992 | 1389.467181 | 2121.081168 | 688.9455712 |
| Deaths | Global | Male | 50-54 years | Colon and rectum cancer | High fasting plasma glucose | Rate | 1992 | 0.733961521 | 1.132066423 | 0.35617147 |
| Deaths | Global | Female | 50-54 years | Colon and rectum cancer | High fasting plasma glucose | Rate | 1992 | 0.5484001 | 0.829197226 | 0.273192157 |
| Deaths | Global | Both | 50-54 years | Colon and rectum cancer | High fasting plasma glucose | Rate | 1992 | 0.642360396 | 0.98059066 | 0.318504356 |
| Deaths | Global | Male | 50-54 years | Colon and rectum cancer | High fasting plasma glucose | Number | 1993 | 818.0951863 | 1247.696099 | 398.7908972 |
| Deaths | Global | Female | 50-54 years | Colon and rectum cancer | High fasting plasma glucose | Number | 1993 | 590.820842 | 896.0876105 | 299.3000795 |
| Deaths | Global | Both | 50-54 years | Colon and rectum cancer | High fasting plasma glucose | Number | 1993 | 1408.916028 | 2131.705984 | 704.9145276 |
| Deaths | Global | Male | 50-54 years | Colon and rectum cancer | High fasting plasma glucose | Rate | 1993 | 0.743230338 | 1.133517968 | 0.362297075 |
| Deaths | Global | Female | 50-54 years | Colon and rectum cancer | High fasting plasma glucose | Rate | 1993 | 0.551302657 | 0.836151072 | 0.27928082 |
| Deaths | Global | Both | 50-54 years | Colon and rectum cancer | High fasting plasma glucose | Rate | 1993 | 0.64854964 | 0.981262986 | 0.324484961 |
| Deaths | Global | Male | 50-54 years | Colon and rectum cancer | High fasting plasma glucose | Number | 1994 | 828.6640928 | 1249.948388 | 405.6316364 |
| Deaths | Global | Female | 50-54 years | Colon and rectum cancer | High fasting plasma glucose | Number | 1994 | 592.4524868 | 896.6285435 | 296.5230569 |
| Deaths | Global | Both | 50-54 years | Colon and rectum cancer | High fasting plasma glucose | Number | 1994 | 1421.11658 | 2138.095578 | 714.5668599 |
| Deaths | Global | Male | 50-54 years | Colon and rectum cancer | High fasting plasma glucose | Rate | 1994 | 0.749936483 | 1.131196472 | 0.367094418 |
| Deaths | Global | Female | 50-54 years | Colon and rectum cancer | High fasting plasma glucose | Rate | 1994 | 0.550344842 | 0.832902055 | 0.275448139 |
| Deaths | Global | Both | 50-54 years | Colon and rectum cancer | High fasting plasma glucose | Rate | 1994 | 0.651442961 | 0.980107709 | 0.327559018 |
| Deaths | Global | Male | 50-54 years | Colon and rectum cancer | High fasting plasma glucose | Number | 1995 | 833.416632 | 1270.435436 | 410.4496082 |
| Deaths | Global | Female | 50-54 years | Colon and rectum cancer | High fasting plasma glucose | Number | 1995 | 591.2879783 | 905.1244411 | 298.1213849 |
| Deaths | Global | Both | 50-54 years | Colon and rectum cancer | High fasting plasma glucose | Number | 1995 | 1424.70461 | 2186.731233 | 720.9798503 |
| Deaths | Global | Male | 50-54 years | Colon and rectum cancer | High fasting plasma glucose | Rate | 1995 | 0.751759288 | 1.145959419 | 0.370234158 |
| Deaths | Global | Female | 50-54 years | Colon and rectum cancer | High fasting plasma glucose | Rate | 1995 | 0.547969252 | 0.838813541 | 0.276280524 |
| Deaths | Global | Both | 50-54 years | Colon and rectum cancer | High fasting plasma glucose | Rate | 1995 | 0.651241476 | 0.999568658 | 0.329564443 |
| Deaths | Global | Male | 50-54 years | Colon and rectum cancer | High fasting plasma glucose | Number | 1996 | 842.6302096 | 1296.780104 | 408.3683938 |
| Deaths | Global | Female | 50-54 years | Colon and rectum cancer | High fasting plasma glucose | Number | 1996 | 594.7857353 | 905.3341204 | 299.4800588 |
| Deaths | Global | Both | 50-54 years | Colon and rectum cancer | High fasting plasma glucose | Number | 1996 | 1437.415945 | 2165.932923 | 723.6996466 |
| Deaths | Global | Male | 50-54 years | Colon and rectum cancer | High fasting plasma glucose | Rate | 1996 | 0.750299982 | 1.154686929 | 0.363621901 |
| Deaths | Global | Female | 50-54 years | Colon and rectum cancer | High fasting plasma glucose | Rate | 1996 | 0.544063627 | 0.828129082 | 0.273941013 |
| Deaths | Global | Both | 50-54 years | Colon and rectum cancer | High fasting plasma glucose | Rate | 1996 | 0.648569695 | 0.977280418 | 0.326537117 |
| Deaths | Global | Male | 50-54 years | Colon and rectum cancer | High fasting plasma glucose | Number | 1997 | 880.5653339 | 1321.040921 | 428.9930556 |
| Deaths | Global | Female | 50-54 years | Colon and rectum cancer | High fasting plasma glucose | Number | 1997 | 614.5250613 | 925.2704052 | 311.7819955 |
| Deaths | Global | Both | 50-54 years | Colon and rectum cancer | High fasting plasma glucose | Number | 1997 | 1495.090395 | 2254.093104 | 763.7916144 |
| Deaths | Global | Male | 50-54 years | Colon and rectum cancer | High fasting plasma glucose | Rate | 1997 | 0.757059558 | 1.135755199 | 0.368823619 |
| Deaths | Global | Female | 50-54 years | Colon and rectum cancer | High fasting plasma glucose | Rate | 1997 | 0.542559279 | 0.816913866 | 0.275269839 |
| Deaths | Global | Both | 50-54 years | Colon and rectum cancer | High fasting plasma glucose | Rate | 1997 | 0.651234144 | 0.981841899 | 0.332693715 |
| Deaths | Global | Male | 50-54 years | Colon and rectum cancer | High fasting plasma glucose | Number | 1998 | 916.8341411 | 1379.595831 | 451.4320896 |
| Deaths | Global | Female | 50-54 years | Colon and rectum cancer | High fasting plasma glucose | Number | 1998 | 637.8869752 | 976.3343617 | 321.5215546 |
| Deaths | Global | Both | 50-54 years | Colon and rectum cancer | High fasting plasma glucose | Number | 1998 | 1554.721116 | 2354.275608 | 790.7596947 |
| Deaths | Global | Male | 50-54 years | Colon and rectum cancer | High fasting plasma glucose | Rate | 1998 | 0.759718005 | 1.143177097 | 0.374071024 |
| Deaths | Global | Female | 50-54 years | Colon and rectum cancer | High fasting plasma glucose | Rate | 1998 | 0.542296147 | 0.830025355 | 0.273339803 |
| Deaths | Global | Both | 50-54 years | Colon and rectum cancer | High fasting plasma glucose | Rate | 1998 | 0.652400152 | 0.987913362 | 0.331822691 |
| Deaths | Global | Male | 50-54 years | Colon and rectum cancer | High fasting plasma glucose | Number | 1999 | 982.6678713 | 1496.710023 | 483.8882782 |
| Deaths | Global | Female | 50-54 years | Colon and rectum cancer | High fasting plasma glucose | Number | 1999 | 678.1573884 | 1039.5178 | 343.0992552 |
| Deaths | Global | Both | 50-54 years | Colon and rectum cancer | High fasting plasma glucose | Number | 1999 | 1660.82526 | 2515.213507 | 835.4302709 |
| Deaths | Global | Male | 50-54 years | Colon and rectum cancer | High fasting plasma glucose | Rate | 1999 | 0.773817493 | 1.178608186 | 0.381045545 |
| Deaths | Global | Female | 50-54 years | Colon and rectum cancer | High fasting plasma glucose | Rate | 1999 | 0.547822633 | 0.839733355 | 0.277159168 |
| Deaths | Global | Both | 50-54 years | Colon and rectum cancer | High fasting plasma glucose | Rate | 1999 | 0.662261116 | 1.002952053 | 0.333131363 |
| Deaths | Global | Male | 50-54 years | Colon and rectum cancer | High fasting plasma glucose | Number | 2000 | 1046.36161 | 1593.854122 | 509.9839785 |
| Deaths | Global | Female | 50-54 years | Colon and rectum cancer | High fasting plasma glucose | Number | 2000 | 711.9215454 | 1078.442795 | 363.8122049 |
| Deaths | Global | Both | 50-54 years | Colon and rectum cancer | High fasting plasma glucose | Number | 2000 | 1758.283155 | 2671.281768 | 902.4405876 |
| Deaths | Global | Male | 50-54 years | Colon and rectum cancer | High fasting plasma glucose | Rate | 2000 | 0.7925408 | 1.207225503 | 0.386274789 |
| Deaths | Global | Female | 50-54 years | Colon and rectum cancer | High fasting plasma glucose | Rate | 2000 | 0.551473814 | 0.835391154 | 0.28181884 |
| Deaths | Global | Both | 50-54 years | Colon and rectum cancer | High fasting plasma glucose | Rate | 2000 | 0.673360657 | 1.02300704 | 0.345603031 |
| Deaths | Global | Male | 50-54 years | Colon and rectum cancer | High fasting plasma glucose | Number | 2001 | 1115.48416 | 1694.695353 | 549.8586364 |
| Deaths | Global | Female | 50-54 years | Colon and rectum cancer | High fasting plasma glucose | Number | 2001 | 754.7537167 | 1139.743432 | 384.7693511 |
| Deaths | Global | Both | 50-54 years | Colon and rectum cancer | High fasting plasma glucose | Number | 2001 | 1870.237877 | 2840.661414 | 958.6312147 |
| Deaths | Global | Male | 50-54 years | Colon and rectum cancer | High fasting plasma glucose | Rate | 2001 | 0.806534309 | 1.225324387 | 0.397567147 |
| Deaths | Global | Female | 50-54 years | Colon and rectum cancer | High fasting plasma glucose | Rate | 2001 | 0.556087081 | 0.839739618 | 0.283490178 |
| Deaths | Global | Both | 50-54 years | Colon and rectum cancer | High fasting plasma glucose | Rate | 2001 | 0.682489689 | 1.036617935 | 0.349824976 |
| Deaths | Global | Male | 50-54 years | Colon and rectum cancer | High fasting plasma glucose | Number | 2002 | 1163.489791 | 1747.679251 | 574.9071813 |
| Deaths | Global | Female | 50-54 years | Colon and rectum cancer | High fasting plasma glucose | Number | 2002 | 779.4299257 | 1204.817069 | 397.8502867 |
| Deaths | Global | Both | 50-54 years | Colon and rectum cancer | High fasting plasma glucose | Number | 2002 | 1942.919717 | 2952.763887 | 1006.035548 |
| Deaths | Global | Male | 50-54 years | Colon and rectum cancer | High fasting plasma glucose | Rate | 2002 | 0.814760378 | 1.22385243 | 0.402591923 |
| Deaths | Global | Female | 50-54 years | Colon and rectum cancer | High fasting plasma glucose | Rate | 2002 | 0.554450399 | 0.857051137 | 0.283012292 |
| Deaths | Global | Both | 50-54 years | Colon and rectum cancer | High fasting plasma glucose | Rate | 2002 | 0.685627066 | 1.041985843 | 0.355014772 |
| Deaths | Global | Male | 50-54 years | Colon and rectum cancer | High fasting plasma glucose | Number | 2003 | 1206.355236 | 1803.86935 | 593.2639112 |
| Deaths | Global | Female | 50-54 years | Colon and rectum cancer | High fasting plasma glucose | Number | 2003 | 807.2764096 | 1253.9705 | 412.9374417 |
| Deaths | Global | Both | 50-54 years | Colon and rectum cancer | High fasting plasma glucose | Number | 2003 | 2013.631646 | 3047.394395 | 1043.328907 |
| Deaths | Global | Male | 50-54 years | Colon and rectum cancer | High fasting plasma glucose | Rate | 2003 | 0.816303545 | 1.220622998 | 0.401443471 |
| Deaths | Global | Female | 50-54 years | Colon and rectum cancer | High fasting plasma glucose | Rate | 2003 | 0.552780341 | 0.858652912 | 0.282757798 |
| Deaths | Global | Both | 50-54 years | Colon and rectum cancer | High fasting plasma glucose | Rate | 2003 | 0.685323754 | 1.037156806 | 0.355088819 |
| Deaths | Global | Male | 50-54 years | Colon and rectum cancer | High fasting plasma glucose | Number | 2004 | 1241.402367 | 1850.785119 | 618.4242474 |
| Deaths | Global | Female | 50-54 years | Colon and rectum cancer | High fasting plasma glucose | Number | 2004 | 815.6215172 | 1254.07929 | 420.7647882 |
| Deaths | Global | Both | 50-54 years | Colon and rectum cancer | High fasting plasma glucose | Number | 2004 | 2057.023884 | 3104.39779 | 1052.457604 |
| Deaths | Global | Male | 50-54 years | Colon and rectum cancer | High fasting plasma glucose | Rate | 2004 | 0.817685959 | 1.219073723 | 0.407343209 |
| Deaths | Global | Female | 50-54 years | Colon and rectum cancer | High fasting plasma glucose | Rate | 2004 | 0.541769161 | 0.833010741 | 0.279489176 |
| Deaths | Global | Both | 50-54 years | Colon and rectum cancer | High fasting plasma glucose | Rate | 2004 | 0.680307547 | 1.026699428 | 0.348073183 |
| Deaths | Global | Male | 50-54 years | Colon and rectum cancer | High fasting plasma glucose | Number | 2005 | 1301.234364 | 1934.728703 | 646.8205306 |
| Deaths | Global | Female | 50-54 years | Colon and rectum cancer | High fasting plasma glucose | Number | 2005 | 845.4736281 | 1273.519632 | 435.584117 |
| Deaths | Global | Both | 50-54 years | Colon and rectum cancer | High fasting plasma glucose | Number | 2005 | 2146.707993 | 3239.139935 | 1100.611526 |
| Deaths | Global | Male | 50-54 years | Colon and rectum cancer | High fasting plasma glucose | Rate | 2005 | 0.824469486 | 1.225855098 | 0.409829163 |
| Deaths | Global | Female | 50-54 years | Colon and rectum cancer | High fasting plasma glucose | Rate | 2005 | 0.539511309 | 0.81265485 | 0.27795374 |
| Deaths | Global | Both | 50-54 years | Colon and rectum cancer | High fasting plasma glucose | Rate | 2005 | 0.682495857 | 1.029809175 | 0.349913826 |
| Deaths | Global | Male | 50-54 years | Colon and rectum cancer | High fasting plasma glucose | Number | 2006 | 1340.270163 | 2006.930817 | 671.9827289 |
| Deaths | Global | Female | 50-54 years | Colon and rectum cancer | High fasting plasma glucose | Number | 2006 | 857.3834331 | 1295.095641 | 446.2656702 |
| Deaths | Global | Both | 50-54 years | Colon and rectum cancer | High fasting plasma glucose | Number | 2006 | 2197.653596 | 3278.044492 | 1143.094488 |
| Deaths | Global | Male | 50-54 years | Colon and rectum cancer | High fasting plasma glucose | Rate | 2006 | 0.828185902 | 1.240131919 | 0.415234658 |
| Deaths | Global | Female | 50-54 years | Colon and rectum cancer | High fasting plasma glucose | Rate | 2006 | 0.533473213 | 0.805822467 | 0.27767131 |
| Deaths | Global | Both | 50-54 years | Colon and rectum cancer | High fasting plasma glucose | Rate | 2006 | 0.681338853 | 1.016292595 | 0.354393745 |
| Deaths | Global | Male | 50-54 years | Colon and rectum cancer | High fasting plasma glucose | Number | 2007 | 1378.643526 | 2071.895506 | 686.2560095 |
| Deaths | Global | Female | 50-54 years | Colon and rectum cancer | High fasting plasma glucose | Number | 2007 | 871.7726829 | 1317.754721 | 450.9878861 |
| Deaths | Global | Both | 50-54 years | Colon and rectum cancer | High fasting plasma glucose | Number | 2007 | 2250.416209 | 3367.675894 | 1178.576271 |
| Deaths | Global | Male | 50-54 years | Colon and rectum cancer | High fasting plasma glucose | Rate | 2007 | 0.834268521 | 1.253781102 | 0.415279059 |
| Deaths | Global | Female | 50-54 years | Colon and rectum cancer | High fasting plasma glucose | Rate | 2007 | 0.531119039 | 0.802829263 | 0.274759989 |
| Deaths | Global | Both | 50-54 years | Colon and rectum cancer | High fasting plasma glucose | Rate | 2007 | 0.683205911 | 1.022395798 | 0.357805046 |
| Deaths | Global | Male | 50-54 years | Colon and rectum cancer | High fasting plasma glucose | Number | 2008 | 1402.381138 | 2124.559498 | 692.3988033 |
| Deaths | Global | Female | 50-54 years | Colon and rectum cancer | High fasting plasma glucose | Number | 2008 | 878.2461899 | 1324.889819 | 455.9637495 |
| Deaths | Global | Both | 50-54 years | Colon and rectum cancer | High fasting plasma glucose | Number | 2008 | 2280.627328 | 3462.124721 | 1180.533241 |
| Deaths | Global | Male | 50-54 years | Colon and rectum cancer | High fasting plasma glucose | Rate | 2008 | 0.836291118 | 1.266952464 | 0.412902708 |
| Deaths | Global | Female | 50-54 years | Colon and rectum cancer | High fasting plasma glucose | Rate | 2008 | 0.526966835 | 0.794962737 | 0.273588177 |
| Deaths | Global | Both | 50-54 years | Colon and rectum cancer | High fasting plasma glucose | Rate | 2008 | 0.682105394 | 1.035475598 | 0.353081839 |
| Deaths | Global | Male | 50-54 years | Colon and rectum cancer | High fasting plasma glucose | Number | 2009 | 1390.513113 | 2118.534657 | 703.5115902 |
| Deaths | Global | Female | 50-54 years | Colon and rectum cancer | High fasting plasma glucose | Number | 2009 | 876.1915429 | 1318.122607 | 461.2956503 |
| Deaths | Global | Both | 50-54 years | Colon and rectum cancer | High fasting plasma glucose | Number | 2009 | 2266.704656 | 3430.926879 | 1174.370765 |
| Deaths | Global | Male | 50-54 years | Colon and rectum cancer | High fasting plasma glucose | Rate | 2009 | 0.8207697 | 1.250494539 | 0.415257498 |
| Deaths | Global | Female | 50-54 years | Colon and rectum cancer | High fasting plasma glucose | Rate | 2009 | 0.519656967 | 0.78176011 | 0.273588008 |
| Deaths | Global | Both | 50-54 years | Colon and rectum cancer | High fasting plasma glucose | Rate | 2009 | 0.67057239 | 1.014991005 | 0.347420917 |
| Deaths | Global | Male | 50-54 years | Colon and rectum cancer | High fasting plasma glucose | Number | 2010 | 1395.777474 | 2126.986655 | 696.5393576 |
| Deaths | Global | Female | 50-54 years | Colon and rectum cancer | High fasting plasma glucose | Number | 2010 | 885.6048114 | 1342.049667 | 458.6975801 |
| Deaths | Global | Both | 50-54 years | Colon and rectum cancer | High fasting plasma glucose | Number | 2010 | 2281.382285 | 3427.172599 | 1172.508261 |
| Deaths | Global | Male | 50-54 years | Colon and rectum cancer | High fasting plasma glucose | Rate | 2010 | 0.815569071 | 1.242823132 | 0.406996078 |
| Deaths | Global | Female | 50-54 years | Colon and rectum cancer | High fasting plasma glucose | Rate | 2010 | 0.519045693 | 0.786564267 | 0.268838877 |
| Deaths | Global | Both | 50-54 years | Colon and rectum cancer | High fasting plasma glucose | Rate | 2010 | 0.667532877 | 1.002791334 | 0.343076133 |
| Deaths | Global | Male | 50-54 years | Colon and rectum cancer | High fasting plasma glucose | Number | 2011 | 1417.012943 | 2145.776975 | 716.1782281 |
| Deaths | Global | Female | 50-54 years | Colon and rectum cancer | High fasting plasma glucose | Number | 2011 | 898.1415156 | 1356.391481 | 465.482482 |
| Deaths | Global | Both | 50-54 years | Colon and rectum cancer | High fasting plasma glucose | Number | 2011 | 2315.154459 | 3492.811917 | 1188.894862 |
| Deaths | Global | Male | 50-54 years | Colon and rectum cancer | High fasting plasma glucose | Rate | 2011 | 0.815369939 | 1.234711404 | 0.41209941 |
| Deaths | Global | Female | 50-54 years | Colon and rectum cancer | High fasting plasma glucose | Rate | 2011 | 0.517184177 | 0.781062004 | 0.268042586 |
| Deaths | Global | Both | 50-54 years | Colon and rectum cancer | High fasting plasma glucose | Rate | 2011 | 0.666331914 | 1.005277224 | 0.342179584 |
| Deaths | Global | Male | 50-54 years | Colon and rectum cancer | High fasting plasma glucose | Number | 2012 | 1451.645542 | 2189.869263 | 735.6392132 |
| Deaths | Global | Female | 50-54 years | Colon and rectum cancer | High fasting plasma glucose | Number | 2012 | 919.0221592 | 1380.906673 | 468.2161425 |
| Deaths | Global | Both | 50-54 years | Colon and rectum cancer | High fasting plasma glucose | Number | 2012 | 2370.667701 | 3592.736524 | 1238.805983 |
| Deaths | Global | Male | 50-54 years | Colon and rectum cancer | High fasting plasma glucose | Rate | 2012 | 0.815469756 | 1.230170935 | 0.413249318 |
| Deaths | Global | Female | 50-54 years | Colon and rectum cancer | High fasting plasma glucose | Rate | 2012 | 0.515485616 | 0.774559699 | 0.262625535 |
| Deaths | Global | Both | 50-54 years | Colon and rectum cancer | High fasting plasma glucose | Rate | 2012 | 0.665364283 | 1.008356658 | 0.347689916 |
| Deaths | Global | Male | 50-54 years | Colon and rectum cancer | High fasting plasma glucose | Number | 2013 | 1498.533835 | 2228.504223 | 754.7068873 |
| Deaths | Global | Female | 50-54 years | Colon and rectum cancer | High fasting plasma glucose | Number | 2013 | 954.6384872 | 1440.158707 | 497.3055239 |
| Deaths | Global | Both | 50-54 years | Colon and rectum cancer | High fasting plasma glucose | Number | 2013 | 2453.172322 | 3648.499244 | 1268.696778 |
| Deaths | Global | Male | 50-54 years | Colon and rectum cancer | High fasting plasma glucose | Rate | 2013 | 0.813613616 | 1.209943572 | 0.409760384 |
| Deaths | Global | Female | 50-54 years | Colon and rectum cancer | High fasting plasma glucose | Rate | 2013 | 0.516393671 | 0.779026669 | 0.269008036 |
| Deaths | Global | Both | 50-54 years | Colon and rectum cancer | High fasting plasma glucose | Rate | 2013 | 0.664728237 | 0.98862214 | 0.343774698 |
| Deaths | Global | Male | 50-54 years | Colon and rectum cancer | High fasting plasma glucose | Number | 2014 | 1556.251557 | 2353.373337 | 801.0457008 |
| Deaths | Global | Female | 50-54 years | Colon and rectum cancer | High fasting plasma glucose | Number | 2014 | 1003.786757 | 1525.773333 | 518.4785501 |
| Deaths | Global | Both | 50-54 years | Colon and rectum cancer | High fasting plasma glucose | Number | 2014 | 2560.038314 | 3870.561592 | 1331.083735 |
| Deaths | Global | Male | 50-54 years | Colon and rectum cancer | High fasting plasma glucose | Rate | 2014 | 0.813470184 | 1.230134699 | 0.418715593 |
| Deaths | Global | Female | 50-54 years | Colon and rectum cancer | High fasting plasma glucose | Rate | 2014 | 0.521881521 | 0.79326899 | 0.269563602 |
| Deaths | Global | Both | 50-54 years | Colon and rectum cancer | High fasting plasma glucose | Rate | 2014 | 0.667284526 | 1.008877814 | 0.346952456 |
| Deaths | Global | Male | 50-54 years | Colon and rectum cancer | High fasting plasma glucose | Number | 2015 | 1630.32927 | 2478.276351 | 815.5308825 |
| Deaths | Global | Female | 50-54 years | Colon and rectum cancer | High fasting plasma glucose | Number | 2015 | 1059.587452 | 1583.1639 | 546.6485844 |
| Deaths | Global | Both | 50-54 years | Colon and rectum cancer | High fasting plasma glucose | Number | 2015 | 2689.916722 | 4029.331358 | 1405.477475 |
| Deaths | Global | Male | 50-54 years | Colon and rectum cancer | High fasting plasma glucose | Rate | 2015 | 0.821907805 | 1.249388521 | 0.411138541 |
| Deaths | Global | Female | 50-54 years | Colon and rectum cancer | High fasting plasma glucose | Rate | 2015 | 0.530637415 | 0.792842534 | 0.273759557 |
| Deaths | Global | Both | 50-54 years | Colon and rectum cancer | High fasting plasma glucose | Rate | 2015 | 0.6757886 | 1.012290148 | 0.353098535 |
| Deaths | Global | Male | 50-54 years | Colon and rectum cancer | High fasting plasma glucose | Number | 2016 | 1697.172144 | 2619.745703 | 852.2816686 |
| Deaths | Global | Female | 50-54 years | Colon and rectum cancer | High fasting plasma glucose | Number | 2016 | 1097.413425 | 1664.442817 | 581.4824197 |
| Deaths | Global | Both | 50-54 years | Colon and rectum cancer | High fasting plasma glucose | Number | 2016 | 2794.585569 | 4228.250203 | 1451.347944 |
| Deaths | Global | Male | 50-54 years | Colon and rectum cancer | High fasting plasma glucose | Rate | 2016 | 0.830443957 | 1.281868782 | 0.417030272 |
| Deaths | Global | Female | 50-54 years | Colon and rectum cancer | High fasting plasma glucose | Rate | 2016 | 0.532631455 | 0.807840126 | 0.282223472 |
| Deaths | Global | Both | 50-54 years | Colon and rectum cancer | High fasting plasma glucose | Rate | 2016 | 0.680932906 | 1.030261779 | 0.353637614 |
| Deaths | Global | Male | 50-54 years | Colon and rectum cancer | High fasting plasma glucose | Number | 2017 | 1765.041596 | 2731.273075 | 874.8462245 |
| Deaths | Global | Female | 50-54 years | Colon and rectum cancer | High fasting plasma glucose | Number | 2017 | 1128.433056 | 1734.413755 | 581.5604156 |
| Deaths | Global | Both | 50-54 years | Colon and rectum cancer | High fasting plasma glucose | Number | 2017 | 2893.474651 | 4396.71228 | 1469.763052 |
| Deaths | Global | Male | 50-54 years | Colon and rectum cancer | High fasting plasma glucose | Rate | 2017 | 0.842366513 | 1.303500711 | 0.417520565 |
| Deaths | Global | Female | 50-54 years | Colon and rectum cancer | High fasting plasma glucose | Rate | 2017 | 0.533722474 | 0.820337188 | 0.275064491 |
| Deaths | Global | Both | 50-54 years | Colon and rectum cancer | High fasting plasma glucose | Rate | 2017 | 0.687350438 | 1.044447412 | 0.349145024 |
| Deaths | Global | Male | 50-54 years | Colon and rectum cancer | High fasting plasma glucose | Number | 2018 | 1821.918204 | 2761.801466 | 895.0634166 |
| Deaths | Global | Female | 50-54 years | Colon and rectum cancer | High fasting plasma glucose | Number | 2018 | 1153.696321 | 1755.499707 | 593.892637 |
| Deaths | Global | Both | 50-54 years | Colon and rectum cancer | High fasting plasma glucose | Number | 2018 | 2975.614525 | 4480.754741 | 1530.861126 |
| Deaths | Global | Male | 50-54 years | Colon and rectum cancer | High fasting plasma glucose | Rate | 2018 | 0.853721493 | 1.294135635 | 0.419412285 |
| Deaths | Global | Female | 50-54 years | Colon and rectum cancer | High fasting plasma glucose | Rate | 2018 | 0.535926704 | 0.815482511 | 0.275881025 |
| Deaths | Global | Both | 50-54 years | Colon and rectum cancer | High fasting plasma glucose | Rate | 2018 | 0.694133798 | 1.045244026 | 0.35711025 |
| Deaths | Global | Male | 50-54 years | Colon and rectum cancer | High fasting plasma glucose | Number | 2019 | 1864.017178 | 2856.202307 | 875.4885624 |
| Deaths | Global | Female | 50-54 years | Colon and rectum cancer | High fasting plasma glucose | Number | 2019 | 1166.810934 | 1764.33116 | 604.1060538 |
| Deaths | Global | Both | 50-54 years | Colon and rectum cancer | High fasting plasma glucose | Number | 2019 | 3030.828112 | 4674.743192 | 1537.418532 |
| Deaths | Global | Male | 50-54 years | Colon and rectum cancer | High fasting plasma glucose | Rate | 2019 | 0.860951833 | 1.319222076 | 0.404370459 |
| Deaths | Global | Female | 50-54 years | Colon and rectum cancer | High fasting plasma glucose | Rate | 2019 | 0.534886077 | 0.808799562 | 0.276932541 |
| Deaths | Global | Both | 50-54 years | Colon and rectum cancer | High fasting plasma glucose | Rate | 2019 | 0.697305533 | 1.075522653 | 0.353715357 |
| Deaths | Global | Male | 50-54 years | Colon and rectum cancer | High fasting plasma glucose | Number | 2020 | 1876.656029 | 2907.149834 | 928.1576111 |
| Deaths | Global | Female | 50-54 years | Colon and rectum cancer | High fasting plasma glucose | Number | 2020 | 1175.736815 | 1789.637685 | 602.4978981 |
| Deaths | Global | Both | 50-54 years | Colon and rectum cancer | High fasting plasma glucose | Number | 2020 | 3052.392845 | 4642.405388 | 1555.843855 |
| Deaths | Global | Male | 50-54 years | Colon and rectum cancer | High fasting plasma glucose | Rate | 2020 | 0.856276156 | 1.326467422 | 0.423497551 |
| Deaths | Global | Female | 50-54 years | Colon and rectum cancer | High fasting plasma glucose | Rate | 2020 | 0.533240987 | 0.811668184 | 0.273255519 |
| Deaths | Global | Both | 50-54 years | Colon and rectum cancer | High fasting plasma glucose | Rate | 2020 | 0.694272161 | 1.055923331 | 0.353879442 |
| Deaths | Global | Male | 50-54 years | Colon and rectum cancer | High fasting plasma glucose | Number | 2021 | 1882.152522 | 2868.513589 | 912.1278598 |
| Deaths | Global | Female | 50-54 years | Colon and rectum cancer | High fasting plasma glucose | Number | 2021 | 1195.386417 | 1838.777184 | 623.3319571 |
| Deaths | Global | Both | 50-54 years | Colon and rectum cancer | High fasting plasma glucose | Number | 2021 | 3077.538939 | 4658.154695 | 1558.689635 |
| Deaths | Global | Male | 50-54 years | Colon and rectum cancer | High fasting plasma glucose | Rate | 2021 | 0.847891249 | 1.292237234 | 0.410904654 |
| Deaths | Global | Female | 50-54 years | Colon and rectum cancer | High fasting plasma glucose | Rate | 2021 | 0.536185814 | 0.824776179 | 0.279593066 |
| Deaths | Global | Both | 50-54 years | Colon and rectum cancer | High fasting plasma glucose | Rate | 2021 | 0.691701498 | 1.046957535 | 0.350327966 |
| Deaths | Global | Male | 55-59 years | Colon and rectum cancer | High fasting plasma glucose | Number | 1990 | 1343.571964 | 2080.952847 | 681.3100775 |
| Deaths | Global | Female | 55-59 years | Colon and rectum cancer | High fasting plasma glucose | Number | 1990 | 969.0964958 | 1476.195819 | 477.0027401 |
| Deaths | Global | Both | 55-59 years | Colon and rectum cancer | High fasting plasma glucose | Number | 1990 | 2312.66846 | 3589.10258 | 1162.340667 |
| Deaths | Global | Male | 55-59 years | Colon and rectum cancer | High fasting plasma glucose | Rate | 1990 | 1.446442448 | 2.240280842 | 0.733474531 |
| Deaths | Global | Female | 55-59 years | Colon and rectum cancer | High fasting plasma glucose | Rate | 1990 | 1.049806409 | 1.599138825 | 0.516729279 |
| Deaths | Global | Both | 55-59 years | Colon and rectum cancer | High fasting plasma glucose | Rate | 1990 | 1.248741346 | 1.937960787 | 0.627613891 |
| Deaths | Global | Male | 55-59 years | Colon and rectum cancer | High fasting plasma glucose | Number | 1991 | 1358.300949 | 2118.064505 | 679.0378548 |
| Deaths | Global | Female | 55-59 years | Colon and rectum cancer | High fasting plasma glucose | Number | 1991 | 982.4290773 | 1496.231964 | 485.6689132 |
| Deaths | Global | Both | 55-59 years | Colon and rectum cancer | High fasting plasma glucose | Number | 1991 | 2340.730027 | 3598.609368 | 1184.294241 |
| Deaths | Global | Male | 55-59 years | Colon and rectum cancer | High fasting plasma glucose | Rate | 1991 | 1.442663383 | 2.249614936 | 0.721212077 |
| Deaths | Global | Female | 55-59 years | Colon and rectum cancer | High fasting plasma glucose | Rate | 1991 | 1.048609257 | 1.597023871 | 0.518385429 |
| Deaths | Global | Both | 55-59 years | Colon and rectum cancer | High fasting plasma glucose | Rate | 1991 | 1.246122536 | 1.915773361 | 0.6304767 |
| Deaths | Global | Male | 55-59 years | Colon and rectum cancer | High fasting plasma glucose | Number | 1992 | 1380.398446 | 2186.824202 | 701.9643982 |
| Deaths | Global | Female | 55-59 years | Colon and rectum cancer | High fasting plasma glucose | Number | 1992 | 998.0770675 | 1506.22889 | 497.7585686 |
| Deaths | Global | Both | 55-59 years | Colon and rectum cancer | High fasting plasma glucose | Number | 1992 | 2378.475514 | 3710.364384 | 1201.906036 |
| Deaths | Global | Male | 55-59 years | Colon and rectum cancer | High fasting plasma glucose | Rate | 1992 | 1.443007652 | 2.286009568 | 0.733802621 |
| Deaths | Global | Female | 55-59 years | Colon and rectum cancer | High fasting plasma glucose | Rate | 1992 | 1.045734151 | 1.578149664 | 0.521525993 |
| Deaths | Global | Both | 55-59 years | Colon and rectum cancer | High fasting plasma glucose | Rate | 1992 | 1.244597999 | 1.941542833 | 0.628928 |
| Deaths | Global | Male | 55-59 years | Colon and rectum cancer | High fasting plasma glucose | Number | 1993 | 1416.342841 | 2176.787379 | 717.3210903 |
| Deaths | Global | Female | 55-59 years | Colon and rectum cancer | High fasting plasma glucose | Number | 1993 | 1025.088377 | 1557.463839 | 516.521552 |
| Deaths | Global | Both | 55-59 years | Colon and rectum cancer | High fasting plasma glucose | Number | 1993 | 2441.431218 | 3761.934338 | 1234.751243 |
| Deaths | Global | Male | 55-59 years | Colon and rectum cancer | High fasting plasma glucose | Rate | 1993 | 1.458170799 | 2.241073064 | 0.738505281 |
| Deaths | Global | Female | 55-59 years | Colon and rectum cancer | High fasting plasma glucose | Rate | 1993 | 1.05554543 | 1.603738638 | 0.531868253 |
| Deaths | Global | Both | 55-59 years | Colon and rectum cancer | High fasting plasma glucose | Rate | 1993 | 1.25687564 | 1.936685168 | 0.635663518 |
| Deaths | Global | Male | 55-59 years | Colon and rectum cancer | High fasting plasma glucose | Number | 1994 | 1441.053812 | 2202.833657 | 731.4432135 |
| Deaths | Global | Female | 55-59 years | Colon and rectum cancer | High fasting plasma glucose | Number | 1994 | 1046.506269 | 1580.902718 | 529.696574 |
| Deaths | Global | Both | 55-59 years | Colon and rectum cancer | High fasting plasma glucose | Number | 1994 | 2487.560081 | 3794.839275 | 1273.110195 |
| Deaths | Global | Male | 55-59 years | Colon and rectum cancer | High fasting plasma glucose | Rate | 1994 | 1.464212171 | 2.238234148 | 0.743197823 |
| Deaths | Global | Female | 55-59 years | Colon and rectum cancer | High fasting plasma glucose | Rate | 1994 | 1.061522602 | 1.603587113 | 0.537297197 |
| Deaths | Global | Both | 55-59 years | Colon and rectum cancer | High fasting plasma glucose | Rate | 1994 | 1.262696683 | 1.926277481 | 0.64623646 |
| Deaths | Global | Male | 55-59 years | Colon and rectum cancer | High fasting plasma glucose | Number | 1995 | 1458.134448 | 2253.017066 | 754.6891534 |
| Deaths | Global | Female | 55-59 years | Colon and rectum cancer | High fasting plasma glucose | Number | 1995 | 1053.134426 | 1588.706388 | 530.7957417 |
| Deaths | Global | Both | 55-59 years | Colon and rectum cancer | High fasting plasma glucose | Number | 1995 | 2511.268875 | 3868.886428 | 1283.447025 |
| Deaths | Global | Male | 55-59 years | Colon and rectum cancer | High fasting plasma glucose | Rate | 1995 | 1.464673125 | 2.263120214 | 0.758073387 |
| Deaths | Global | Female | 55-59 years | Colon and rectum cancer | High fasting plasma glucose | Rate | 1995 | 1.053216279 | 1.588829867 | 0.530836997 |
| Deaths | Global | Both | 55-59 years | Colon and rectum cancer | High fasting plasma glucose | Rate | 1995 | 1.258492457 | 1.938846308 | 0.643184175 |
| Deaths | Global | Male | 55-59 years | Colon and rectum cancer | High fasting plasma glucose | Number | 1996 | 1469.797663 | 2271.698284 | 755.8868289 |
| Deaths | Global | Female | 55-59 years | Colon and rectum cancer | High fasting plasma glucose | Number | 1996 | 1057.317642 | 1599.021036 | 537.0739915 |
| Deaths | Global | Both | 55-59 years | Colon and rectum cancer | High fasting plasma glucose | Number | 1996 | 2527.115305 | 3890.718144 | 1301.32741 |
| Deaths | Global | Male | 55-59 years | Colon and rectum cancer | High fasting plasma glucose | Rate | 1996 | 1.464074477 | 2.26285261 | 0.752943512 |
| Deaths | Global | Female | 55-59 years | Colon and rectum cancer | High fasting plasma glucose | Rate | 1996 | 1.04731606 | 1.583895269 | 0.531993598 |
| Deaths | Global | Both | 55-59 years | Colon and rectum cancer | High fasting plasma glucose | Rate | 1996 | 1.255111501 | 1.932355472 | 0.646314394 |
| Deaths | Global | Male | 55-59 years | Colon and rectum cancer | High fasting plasma glucose | Number | 1997 | 1486.120459 | 2249.121808 | 753.671823 |
| Deaths | Global | Female | 55-59 years | Colon and rectum cancer | High fasting plasma glucose | Number | 1997 | 1065.086539 | 1612.449315 | 540.5507966 |
| Deaths | Global | Both | 55-59 years | Colon and rectum cancer | High fasting plasma glucose | Number | 1997 | 2551.206999 | 3912.982534 | 1281.129969 |
| Deaths | Global | Male | 55-59 years | Colon and rectum cancer | High fasting plasma glucose | Rate | 1997 | 1.47286406 | 2.229059333 | 0.746948967 |
| Deaths | Global | Female | 55-59 years | Colon and rectum cancer | High fasting plasma glucose | Rate | 1997 | 1.049234011 | 1.588449951 | 0.53250535 |
| Deaths | Global | Both | 55-59 years | Colon and rectum cancer | High fasting plasma glucose | Rate | 1997 | 1.260409832 | 1.93318757 | 0.632935238 |
| Deaths | Global | Male | 55-59 years | Colon and rectum cancer | High fasting plasma glucose | Number | 1998 | 1515.965602 | 2309.433579 | 771.9177234 |
| Deaths | Global | Female | 55-59 years | Colon and rectum cancer | High fasting plasma glucose | Number | 1998 | 1071.87336 | 1605.692016 | 549.0456966 |
| Deaths | Global | Both | 55-59 years | Colon and rectum cancer | High fasting plasma glucose | Number | 1998 | 2587.838961 | 3954.813862 | 1318.456832 |
| Deaths | Global | Male | 55-59 years | Colon and rectum cancer | High fasting plasma glucose | Rate | 1998 | 1.495113574 | 2.277667441 | 0.761300036 |
| Deaths | Global | Female | 55-59 years | Colon and rectum cancer | High fasting plasma glucose | Rate | 1998 | 1.052902833 | 1.577273712 | 0.539328424 |
| Deaths | Global | Both | 55-59 years | Colon and rectum cancer | High fasting plasma glucose | Rate | 1998 | 1.27356527 | 1.94630101 | 0.648858317 |
| Deaths | Global | Male | 55-59 years | Colon and rectum cancer | High fasting plasma glucose | Number | 1999 | 1554.202772 | 2352.712594 | 779.8674205 |
| Deaths | Global | Female | 55-59 years | Colon and rectum cancer | High fasting plasma glucose | Number | 1999 | 1088.267093 | 1636.150872 | 550.0922997 |
| Deaths | Global | Both | 55-59 years | Colon and rectum cancer | High fasting plasma glucose | Number | 1999 | 2642.469865 | 4015.216668 | 1339.434427 |
| Deaths | Global | Male | 55-59 years | Colon and rectum cancer | High fasting plasma glucose | Rate | 1999 | 1.525246604 | 2.308879484 | 0.765337803 |
| Deaths | Global | Female | 55-59 years | Colon and rectum cancer | High fasting plasma glucose | Rate | 1999 | 1.064147525 | 1.599888403 | 0.53790045 |
| Deaths | Global | Both | 55-59 years | Colon and rectum cancer | High fasting plasma glucose | Rate | 1999 | 1.29428139 | 1.966652591 | 0.656054805 |
| Deaths | Global | Male | 55-59 years | Colon and rectum cancer | High fasting plasma glucose | Number | 2000 | 1577.098072 | 2396.227228 | 786.0307659 |
| Deaths | Global | Female | 55-59 years | Colon and rectum cancer | High fasting plasma glucose | Number | 2000 | 1089.320076 | 1644.100686 | 552.8837877 |
| Deaths | Global | Both | 55-59 years | Colon and rectum cancer | High fasting plasma glucose | Number | 2000 | 2666.418147 | 4105.905848 | 1354.005331 |
| Deaths | Global | Male | 55-59 years | Colon and rectum cancer | High fasting plasma glucose | Rate | 2000 | 1.539568844 | 2.339205689 | 0.767326078 |
| Deaths | Global | Female | 55-59 years | Colon and rectum cancer | High fasting plasma glucose | Rate | 2000 | 1.061612844 | 1.602282418 | 0.538820998 |
| Deaths | Global | Both | 55-59 years | Colon and rectum cancer | High fasting plasma glucose | Rate | 2000 | 1.300390064 | 2.002416302 | 0.660337194 |
| Deaths | Global | Male | 55-59 years | Colon and rectum cancer | High fasting plasma glucose | Number | 2001 | 1605.802557 | 2456.087325 | 802.6829052 |
| Deaths | Global | Female | 55-59 years | Colon and rectum cancer | High fasting plasma glucose | Number | 2001 | 1098.242402 | 1656.49598 | 556.1298297 |
| Deaths | Global | Both | 55-59 years | Colon and rectum cancer | High fasting plasma glucose | Number | 2001 | 2704.044959 | 4170.870474 | 1381.432149 |
| Deaths | Global | Male | 55-59 years | Colon and rectum cancer | High fasting plasma glucose | Rate | 2001 | 1.543243815 | 2.36040325 | 0.771412042 |
| Deaths | Global | Female | 55-59 years | Colon and rectum cancer | High fasting plasma glucose | Rate | 2001 | 1.054390965 | 1.59035418 | 0.533924265 |
| Deaths | Global | Both | 55-59 years | Colon and rectum cancer | High fasting plasma glucose | Rate | 2001 | 1.298693872 | 2.003178204 | 0.663471759 |
| Deaths | Global | Male | 55-59 years | Colon and rectum cancer | High fasting plasma glucose | Number | 2002 | 1669.270616 | 2552.351373 | 838.0301626 |
| Deaths | Global | Female | 55-59 years | Colon and rectum cancer | High fasting plasma glucose | Number | 2002 | 1129.007989 | 1720.037539 | 579.7625747 |
| Deaths | Global | Both | 55-59 years | Colon and rectum cancer | High fasting plasma glucose | Number | 2002 | 2798.278605 | 4297.312055 | 1415.216818 |
| Deaths | Global | Male | 55-59 years | Colon and rectum cancer | High fasting plasma glucose | Rate | 2002 | 1.543373272 | 2.359851575 | 0.774825449 |
| Deaths | Global | Female | 55-59 years | Colon and rectum cancer | High fasting plasma glucose | Rate | 2002 | 1.042675835 | 1.58851097 | 0.535429716 |
| Deaths | Global | Both | 55-59 years | Colon and rectum cancer | High fasting plasma glucose | Rate | 2002 | 1.292882766 | 1.985478032 | 0.653869644 |
| Deaths | Global | Male | 55-59 years | Colon and rectum cancer | High fasting plasma glucose | Number | 2003 | 1729.704353 | 2642.64356 | 878.1592064 |
| Deaths | Global | Female | 55-59 years | Colon and rectum cancer | High fasting plasma glucose | Number | 2003 | 1170.21184 | 1763.920744 | 593.511308 |
| Deaths | Global | Both | 55-59 years | Colon and rectum cancer | High fasting plasma glucose | Number | 2003 | 2899.916193 | 4441.222165 | 1488.304274 |
| Deaths | Global | Male | 55-59 years | Colon and rectum cancer | High fasting plasma glucose | Rate | 2003 | 1.536200116 | 2.347007647 | 0.779918413 |
| Deaths | Global | Female | 55-59 years | Colon and rectum cancer | High fasting plasma glucose | Rate | 2003 | 1.036876428 | 1.562937392 | 0.525885882 |
| Deaths | Global | Both | 55-59 years | Colon and rectum cancer | High fasting plasma glucose | Rate | 2003 | 1.286246986 | 1.969887489 | 0.660131797 |
| Deaths | Global | Male | 55-59 years | Colon and rectum cancer | High fasting plasma glucose | Number | 2004 | 1835.784904 | 2809.212189 | 923.6752904 |
| Deaths | Global | Female | 55-59 years | Colon and rectum cancer | High fasting plasma glucose | Number | 2004 | 1227.301307 | 1872.630848 | 629.4013695 |
| Deaths | Global | Both | 55-59 years | Colon and rectum cancer | High fasting plasma glucose | Number | 2004 | 3063.08621 | 4702.786624 | 1560.733343 |
| Deaths | Global | Male | 55-59 years | Colon and rectum cancer | High fasting plasma glucose | Rate | 2004 | 1.544069099 | 2.362813707 | 0.776898465 |
| Deaths | Global | Female | 55-59 years | Colon and rectum cancer | High fasting plasma glucose | Rate | 2004 | 1.029437094 | 1.570727291 | 0.527929949 |
| Deaths | Global | Both | 55-59 years | Colon and rectum cancer | High fasting plasma glucose | Rate | 2004 | 1.286398691 | 1.975020663 | 0.655458316 |
| Deaths | Global | Male | 55-59 years | Colon and rectum cancer | High fasting plasma glucose | Number | 2005 | 1955.613753 | 2980.571267 | 996.2295833 |
| Deaths | Global | Female | 55-59 years | Colon and rectum cancer | High fasting plasma glucose | Number | 2005 | 1288.668966 | 1941.518948 | 654.277747 |
| Deaths | Global | Both | 55-59 years | Colon and rectum cancer | High fasting plasma glucose | Number | 2005 | 3244.282719 | 4923.399723 | 1643.931485 |
| Deaths | Global | Male | 55-59 years | Colon and rectum cancer | High fasting plasma glucose | Rate | 2005 | 1.578011412 | 2.405063611 | 0.803871239 |
| Deaths | Global | Female | 55-59 years | Colon and rectum cancer | High fasting plasma glucose | Rate | 2005 | 1.033222523 | 1.556661299 | 0.524583522 |
| Deaths | Global | Both | 55-59 years | Colon and rectum cancer | High fasting plasma glucose | Rate | 2005 | 1.304746852 | 1.980034062 | 0.661136718 |
| Deaths | Global | Male | 55-59 years | Colon and rectum cancer | High fasting plasma glucose | Number | 2006 | 2066.063226 | 3142.234785 | 1054.147619 |
| Deaths | Global | Female | 55-59 years | Colon and rectum cancer | High fasting plasma glucose | Number | 2006 | 1355.619599 | 2055.048125 | 685.1348499 |
| Deaths | Global | Both | 55-59 years | Colon and rectum cancer | High fasting plasma glucose | Number | 2006 | 3421.682825 | 5224.941029 | 1757.356863 |
| Deaths | Global | Male | 55-59 years | Colon and rectum cancer | High fasting plasma glucose | Rate | 2006 | 1.588040508 | 2.415219468 | 0.810250674 |
| Deaths | Global | Female | 55-59 years | Colon and rectum cancer | High fasting plasma glucose | Rate | 2006 | 1.031086361 | 1.563072778 | 0.521114625 |
| Deaths | Global | Both | 55-59 years | Colon and rectum cancer | High fasting plasma glucose | Rate | 2006 | 1.308101227 | 1.997482561 | 0.67183336 |
| Deaths | Global | Male | 55-59 years | Colon and rectum cancer | High fasting plasma glucose | Number | 2007 | 2145.160809 | 3281.38633 | 1075.444615 |
| Deaths | Global | Female | 55-59 years | Colon and rectum cancer | High fasting plasma glucose | Number | 2007 | 1400.948669 | 2124.003948 | 719.1960517 |
| Deaths | Global | Both | 55-59 years | Colon and rectum cancer | High fasting plasma glucose | Number | 2007 | 3546.109479 | 5323.820668 | 1815.294525 |
| Deaths | Global | Male | 55-59 years | Colon and rectum cancer | High fasting plasma glucose | Rate | 2007 | 1.594483755 | 2.439032624 | 0.799370826 |
| Deaths | Global | Female | 55-59 years | Colon and rectum cancer | High fasting plasma glucose | Rate | 2007 | 1.02691778 | 1.556928863 | 0.527182208 |
| Deaths | Global | Both | 55-59 years | Colon and rectum cancer | High fasting plasma glucose | Rate | 2007 | 1.308725207 | 1.964806318 | 0.669951596 |
| Deaths | Global | Male | 55-59 years | Colon and rectum cancer | High fasting plasma glucose | Number | 2008 | 2233.627958 | 3380.311928 | 1140.317908 |
| Deaths | Global | Female | 55-59 years | Colon and rectum cancer | High fasting plasma glucose | Number | 2008 | 1444.508537 | 2196.535896 | 739.7781237 |
| Deaths | Global | Both | 55-59 years | Colon and rectum cancer | High fasting plasma glucose | Number | 2008 | 3678.136495 | 5540.718608 | 1870.656868 |
| Deaths | Global | Male | 55-59 years | Colon and rectum cancer | High fasting plasma glucose | Rate | 2008 | 1.601899645 | 2.424271445 | 0.817806226 |
| Deaths | Global | Female | 55-59 years | Colon and rectum cancer | High fasting plasma glucose | Rate | 2008 | 1.017582497 | 1.547347367 | 0.521135909 |
| Deaths | Global | Both | 55-59 years | Colon and rectum cancer | High fasting plasma glucose | Rate | 2008 | 1.307125957 | 1.969045228 | 0.664788855 |
| Deaths | Global | Male | 55-59 years | Colon and rectum cancer | High fasting plasma glucose | Number | 2009 | 2275.187477 | 3472.679923 | 1157.571226 |
| Deaths | Global | Female | 55-59 years | Colon and rectum cancer | High fasting plasma glucose | Number | 2009 | 1455.303397 | 2190.566756 | 745.4700444 |
| Deaths | Global | Both | 55-59 years | Colon and rectum cancer | High fasting plasma glucose | Number | 2009 | 3730.490874 | 5601.442572 | 1899.368957 |
| Deaths | Global | Male | 55-59 years | Colon and rectum cancer | High fasting plasma glucose | Rate | 2009 | 1.586230725 | 2.421106677 | 0.807043403 |
| Deaths | Global | Female | 55-59 years | Colon and rectum cancer | High fasting plasma glucose | Rate | 2009 | 0.992993902 | 1.494684499 | 0.508654903 |
| Deaths | Global | Both | 55-59 years | Colon and rectum cancer | High fasting plasma glucose | Rate | 2009 | 1.286417369 | 1.931593792 | 0.654975792 |
| Deaths | Global | Male | 55-59 years | Colon and rectum cancer | High fasting plasma glucose | Number | 2010 | 2382.957861 | 3619.230214 | 1206.849644 |
| Deaths | Global | Female | 55-59 years | Colon and rectum cancer | High fasting plasma glucose | Number | 2010 | 1502.638119 | 2259.244013 | 753.9773177 |
| Deaths | Global | Both | 55-59 years | Colon and rectum cancer | High fasting plasma glucose | Number | 2010 | 3885.59598 | 5860.682994 | 1964.105105 |
| Deaths | Global | Male | 55-59 years | Colon and rectum cancer | High fasting plasma glucose | Rate | 2010 | 1.595576241 | 2.423357053 | 0.808080012 |
| Deaths | Global | Female | 55-59 years | Colon and rectum cancer | High fasting plasma glucose | Rate | 2010 | 0.983530928 | 1.478756816 | 0.49350539 |
| Deaths | Global | Both | 55-59 years | Colon and rectum cancer | High fasting plasma glucose | Rate | 2010 | 1.286077173 | 1.939802968 | 0.650090939 |
| Deaths | Global | Male | 55-59 years | Colon and rectum cancer | High fasting plasma glucose | Number | 2011 | 2475.271205 | 3743.568584 | 1250.034754 |
| Deaths | Global | Female | 55-59 years | Colon and rectum cancer | High fasting plasma glucose | Number | 2011 | 1540.948174 | 2335.286055 | 773.5847136 |
| Deaths | Global | Both | 55-59 years | Colon and rectum cancer | High fasting plasma glucose | Number | 2011 | 4016.219379 | 6053.939736 | 2063.114665 |
| Deaths | Global | Male | 55-59 years | Colon and rectum cancer | High fasting plasma glucose | Rate | 2011 | 1.614598409 | 2.44189803 | 0.815387066 |
| Deaths | Global | Female | 55-59 years | Colon and rectum cancer | High fasting plasma glucose | Rate | 2011 | 0.982625249 | 1.489155237 | 0.493296196 |
| Deaths | Global | Both | 55-59 years | Colon and rectum cancer | High fasting plasma glucose | Rate | 2011 | 1.295031589 | 1.952095355 | 0.665252171 |
| Deaths | Global | Male | 55-59 years | Colon and rectum cancer | High fasting plasma glucose | Number | 2012 | 2557.552898 | 3852.184379 | 1300.426586 |
| Deaths | Global | Female | 55-59 years | Colon and rectum cancer | High fasting plasma glucose | Number | 2012 | 1582.564914 | 2384.953672 | 786.5450264 |
| Deaths | Global | Both | 55-59 years | Colon and rectum cancer | High fasting plasma glucose | Number | 2012 | 4140.117812 | 6188.938962 | 2112.61589 |
| Deaths | Global | Male | 55-59 years | Colon and rectum cancer | High fasting plasma glucose | Rate | 2012 | 1.632303589 | 2.45857452 | 0.829969532 |
| Deaths | Global | Female | 55-59 years | Colon and rectum cancer | High fasting plasma glucose | Rate | 2012 | 0.987596028 | 1.488324904 | 0.490841631 |
| Deaths | Global | Both | 55-59 years | Colon and rectum cancer | High fasting plasma glucose | Rate | 2012 | 1.306328349 | 1.952791389 | 0.666592148 |
| Deaths | Global | Male | 55-59 years | Colon and rectum cancer | High fasting plasma glucose | Number | 2013 | 2596.074991 | 3917.380797 | 1344.798917 |
| Deaths | Global | Female | 55-59 years | Colon and rectum cancer | High fasting plasma glucose | Number | 2013 | 1603.770373 | 2384.728834 | 810.8939717 |
| Deaths | Global | Both | 55-59 years | Colon and rectum cancer | High fasting plasma glucose | Number | 2013 | 4199.845364 | 6250.824548 | 2149.142512 |
| Deaths | Global | Male | 55-59 years | Colon and rectum cancer | High fasting plasma glucose | Rate | 2013 | 1.631607937 | 2.462035813 | 0.845193068 |
| Deaths | Global | Female | 55-59 years | Colon and rectum cancer | High fasting plasma glucose | Rate | 2013 | 0.985456526 | 1.465326104 | 0.498263822 |
| Deaths | Global | Both | 55-59 years | Colon and rectum cancer | High fasting plasma glucose | Rate | 2013 | 1.304886006 | 1.942122334 | 0.667735535 |
| Deaths | Global | Male | 55-59 years | Colon and rectum cancer | High fasting plasma glucose | Number | 2014 | 2611.903574 | 3899.983968 | 1347.54569 |
| Deaths | Global | Female | 55-59 years | Colon and rectum cancer | High fasting plasma glucose | Number | 2014 | 1640.239936 | 2484.667314 | 820.340537 |
| Deaths | Global | Both | 55-59 years | Colon and rectum cancer | High fasting plasma glucose | Number | 2014 | 4252.14351 | 6355.190904 | 2177.547877 |
| Deaths | Global | Male | 55-59 years | Colon and rectum cancer | High fasting plasma glucose | Rate | 2014 | 1.623742689 | 2.42450392 | 0.837729036 |
| Deaths | Global | Female | 55-59 years | Colon and rectum cancer | High fasting plasma glucose | Rate | 2014 | 0.99621125 | 1.509080151 | 0.498239589 |
| Deaths | Global | Both | 55-59 years | Colon and rectum cancer | High fasting plasma glucose | Rate | 2014 | 1.306322867 | 1.952410868 | 0.668975678 |
| Deaths | Global | Male | 55-59 years | Colon and rectum cancer | High fasting plasma glucose | Number | 2015 | 2655.1246 | 3979.412821 | 1353.449011 |
| Deaths | Global | Female | 55-59 years | Colon and rectum cancer | High fasting plasma glucose | Number | 2015 | 1683.817436 | 2498.144228 | 845.6563039 |
| Deaths | Global | Both | 55-59 years | Colon and rectum cancer | High fasting plasma glucose | Number | 2015 | 4338.942036 | 6414.033817 | 2233.636722 |
| Deaths | Global | Male | 55-59 years | Colon and rectum cancer | High fasting plasma glucose | Rate | 2015 | 1.632892324 | 2.447324939 | 0.832366399 |
| Deaths | Global | Female | 55-59 years | Colon and rectum cancer | High fasting plasma glucose | Rate | 2015 | 1.01066118 | 1.499436541 | 0.507579966 |
| Deaths | Global | Both | 55-59 years | Colon and rectum cancer | High fasting plasma glucose | Rate | 2015 | 1.317993767 | 1.948322085 | 0.678487809 |
| Deaths | Global | Male | 55-59 years | Colon and rectum cancer | High fasting plasma glucose | Number | 2016 | 2704.597272 | 4061.208218 | 1407.653728 |
| Deaths | Global | Female | 55-59 years | Colon and rectum cancer | High fasting plasma glucose | Number | 2016 | 1729.248767 | 2606.938484 | 876.6848904 |
| Deaths | Global | Both | 55-59 years | Colon and rectum cancer | High fasting plasma glucose | Number | 2016 | 4433.846039 | 6606.592648 | 2281.397879 |
| Deaths | Global | Male | 55-59 years | Colon and rectum cancer | High fasting plasma glucose | Rate | 2016 | 1.636769118 | 2.457763402 | 0.851884373 |
| Deaths | Global | Female | 55-59 years | Colon and rectum cancer | High fasting plasma glucose | Rate | 2016 | 1.019754484 | 1.537336477 | 0.516989438 |
| Deaths | Global | Both | 55-59 years | Colon and rectum cancer | High fasting plasma glucose | Rate | 2016 | 1.324267408 | 1.973206838 | 0.681390565 |
| Deaths | Global | Male | 55-59 years | Colon and rectum cancer | High fasting plasma glucose | Number | 2017 | 2755.113079 | 4150.852682 | 1401.987329 |
| Deaths | Global | Female | 55-59 years | Colon and rectum cancer | High fasting plasma glucose | Number | 2017 | 1776.246731 | 2664.446839 | 889.0789008 |
| Deaths | Global | Both | 55-59 years | Colon and rectum cancer | High fasting plasma glucose | Number | 2017 | 4531.35981 | 6796.083213 | 2300.529381 |
| Deaths | Global | Male | 55-59 years | Colon and rectum cancer | High fasting plasma glucose | Rate | 2017 | 1.62648835 | 2.450466944 | 0.827666956 |
| Deaths | Global | Female | 55-59 years | Colon and rectum cancer | High fasting plasma glucose | Rate | 2017 | 1.020366259 | 1.530593473 | 0.510732037 |
| Deaths | Global | Both | 55-59 years | Colon and rectum cancer | High fasting plasma glucose | Rate | 2017 | 1.319289909 | 1.978656381 | 0.66979126 |
| Deaths | Global | Male | 55-59 years | Colon and rectum cancer | High fasting plasma glucose | Number | 2018 | 2860.225645 | 4302.460405 | 1460.120388 |
| Deaths | Global | Female | 55-59 years | Colon and rectum cancer | High fasting plasma glucose | Number | 2018 | 1856.766947 | 2795.282712 | 932.4193157 |
| Deaths | Global | Both | 55-59 years | Colon and rectum cancer | High fasting plasma glucose | Number | 2018 | 4716.992592 | 7144.752067 | 2394.891144 |
| Deaths | Global | Male | 55-59 years | Colon and rectum cancer | High fasting plasma glucose | Rate | 2018 | 1.630435916 | 2.452563833 | 0.832323396 |
| Deaths | Global | Female | 55-59 years | Colon and rectum cancer | High fasting plasma glucose | Rate | 2018 | 1.028699821 | 1.548663299 | 0.516585878 |
| Deaths | Global | Both | 55-59 years | Colon and rectum cancer | High fasting plasma glucose | Rate | 2018 | 1.325282598 | 2.007384026 | 0.672866767 |
| Deaths | Global | Male | 55-59 years | Colon and rectum cancer | High fasting plasma glucose | Number | 2019 | 2989.728184 | 4528.94488 | 1513.883813 |
| Deaths | Global | Female | 55-59 years | Colon and rectum cancer | High fasting plasma glucose | Number | 2019 | 1931.793935 | 2918.333207 | 997.6165378 |
| Deaths | Global | Both | 55-59 years | Colon and rectum cancer | High fasting plasma glucose | Number | 2019 | 4921.52212 | 7385.786015 | 2588.12661 |
| Deaths | Global | Male | 55-59 years | Colon and rectum cancer | High fasting plasma glucose | Rate | 2019 | 1.639091611 | 2.482953333 | 0.829973197 |
| Deaths | Global | Female | 55-59 years | Colon and rectum cancer | High fasting plasma glucose | Rate | 2019 | 1.028695031 | 1.55403473 | 0.531238429 |
| Deaths | Global | Both | 55-59 years | Colon and rectum cancer | High fasting plasma glucose | Rate | 2019 | 1.329450291 | 1.995121657 | 0.699130389 |
| Deaths | Global | Male | 55-59 years | Colon and rectum cancer | High fasting plasma glucose | Number | 2020 | 3068.91347 | 4729.689345 | 1547.429275 |
| Deaths | Global | Female | 55-59 years | Colon and rectum cancer | High fasting plasma glucose | Number | 2020 | 1985.049982 | 2999.027731 | 1003.62332 |
| Deaths | Global | Both | 55-59 years | Colon and rectum cancer | High fasting plasma glucose | Number | 2020 | 5053.963453 | 7721.134915 | 2581.20629 |
| Deaths | Global | Male | 55-59 years | Colon and rectum cancer | High fasting plasma glucose | Rate | 2020 | 1.622187677 | 2.500052167 | 0.817950954 |
| Deaths | Global | Female | 55-59 years | Colon and rectum cancer | High fasting plasma glucose | Rate | 2020 | 1.01836888 | 1.538558998 | 0.514878096 |
| Deaths | Global | Both | 55-59 years | Colon and rectum cancer | High fasting plasma glucose | Rate | 2020 | 1.315765981 | 2.010146442 | 0.671999997 |
| Deaths | Global | Male | 55-59 years | Colon and rectum cancer | High fasting plasma glucose | Number | 2021 | 3132.766648 | 4691.768119 | 1605.841022 |
| Deaths | Global | Female | 55-59 years | Colon and rectum cancer | High fasting plasma glucose | Number | 2021 | 2041.580259 | 3072.715654 | 1018.95096 |
| Deaths | Global | Both | 55-59 years | Colon and rectum cancer | High fasting plasma glucose | Number | 2021 | 5174.346907 | 7835.948369 | 2664.239262 |
| Deaths | Global | Male | 55-59 years | Colon and rectum cancer | High fasting plasma glucose | Rate | 2021 | 1.608820137 | 2.409439284 | 0.824673416 |
| Deaths | Global | Female | 55-59 years | Colon and rectum cancer | High fasting plasma glucose | Rate | 2021 | 1.015693765 | 1.528687455 | 0.506931889 |
| Deaths | Global | Both | 55-59 years | Colon and rectum cancer | High fasting plasma glucose | Rate | 2021 | 1.307551362 | 1.980134913 | 0.67325012 |
| Deaths | Global | Male | 60-64 years | Colon and rectum cancer | High fasting plasma glucose | Number | 1990 | 2107.522069 | 3219.223651 | 1047.144016 |
| Deaths | Global | Female | 60-64 years | Colon and rectum cancer | High fasting plasma glucose | Number | 1990 | 1500.44658 | 2278.158245 | 758.6251643 |
| Deaths | Global | Both | 60-64 years | Colon and rectum cancer | High fasting plasma glucose | Number | 1990 | 3607.96865 | 5546.122394 | 1812.229758 |
| Deaths | Global | Male | 60-64 years | Colon and rectum cancer | High fasting plasma glucose | Rate | 1990 | 2.68316547 | 4.098514491 | 1.33315836 |
| Deaths | Global | Female | 60-64 years | Colon and rectum cancer | High fasting plasma glucose | Rate | 1990 | 1.828405298 | 2.776104568 | 0.924440955 |
| Deaths | Global | Both | 60-64 years | Colon and rectum cancer | High fasting plasma glucose | Rate | 1990 | 2.24642662 | 3.453177728 | 1.128347157 |
| Deaths | Global | Male | 60-64 years | Colon and rectum cancer | High fasting plasma glucose | Number | 1991 | 2169.69412 | 3304.60905 | 1085.439296 |
| Deaths | Global | Female | 60-64 years | Colon and rectum cancer | High fasting plasma glucose | Number | 1991 | 1526.710918 | 2305.481575 | 764.2020523 |
| Deaths | Global | Both | 60-64 years | Colon and rectum cancer | High fasting plasma glucose | Number | 1991 | 3696.405038 | 5598.716707 | 1866.267606 |
| Deaths | Global | Male | 60-64 years | Colon and rectum cancer | High fasting plasma glucose | Rate | 1991 | 2.696557388 | 4.107061851 | 1.349014742 |
| Deaths | Global | Female | 60-64 years | Colon and rectum cancer | High fasting plasma glucose | Rate | 1991 | 1.82942246 | 2.762605366 | 0.915725683 |
| Deaths | Global | Both | 60-64 years | Colon and rectum cancer | High fasting plasma glucose | Rate | 1991 | 2.255077093 | 3.415626174 | 1.138559569 |
| Deaths | Global | Male | 60-64 years | Colon and rectum cancer | High fasting plasma glucose | Number | 1992 | 2226.631211 | 3376.194297 | 1118.826715 |
| Deaths | Global | Female | 60-64 years | Colon and rectum cancer | High fasting plasma glucose | Number | 1992 | 1548.909968 | 2346.717839 | 779.7023672 |
| Deaths | Global | Both | 60-64 years | Colon and rectum cancer | High fasting plasma glucose | Number | 1992 | 3775.541179 | 5702.387024 | 1912.370679 |
| Deaths | Global | Male | 60-64 years | Colon and rectum cancer | High fasting plasma glucose | Rate | 1992 | 2.721597174 | 4.126700827 | 1.367534781 |
| Deaths | Global | Female | 60-64 years | Colon and rectum cancer | High fasting plasma glucose | Rate | 1992 | 1.833363362 | 2.777686628 | 0.922892733 |
| Deaths | Global | Both | 60-64 years | Colon and rectum cancer | High fasting plasma glucose | Rate | 1992 | 2.270346502 | 3.429016881 | 1.149966025 |
| Deaths | Global | Male | 60-64 years | Colon and rectum cancer | High fasting plasma glucose | Number | 1993 | 2274.724579 | 3405.721823 | 1130.325792 |
| Deaths | Global | Female | 60-64 years | Colon and rectum cancer | High fasting plasma glucose | Number | 1993 | 1577.20213 | 2398.232526 | 793.5919355 |
| Deaths | Global | Both | 60-64 years | Colon and rectum cancer | High fasting plasma glucose | Number | 1993 | 3851.926709 | 5800.733258 | 1945.464102 |
| Deaths | Global | Male | 60-64 years | Colon and rectum cancer | High fasting plasma glucose | Rate | 1993 | 2.74747457 | 4.113523979 | 1.365238412 |
| Deaths | Global | Female | 60-64 years | Colon and rectum cancer | High fasting plasma glucose | Rate | 1993 | 1.845489568 | 2.806180022 | 0.928584618 |
| Deaths | Global | Both | 60-64 years | Colon and rectum cancer | High fasting plasma glucose | Rate | 1993 | 2.289327436 | 3.447567619 | 1.156253657 |
| Deaths | Global | Male | 60-64 years | Colon and rectum cancer | High fasting plasma glucose | Number | 1994 | 2302.679419 | 3452.660614 | 1161.944479 |
| Deaths | Global | Female | 60-64 years | Colon and rectum cancer | High fasting plasma glucose | Number | 1994 | 1585.122257 | 2386.73383 | 801.1250144 |
| Deaths | Global | Both | 60-64 years | Colon and rectum cancer | High fasting plasma glucose | Number | 1994 | 3887.801677 | 5836.713729 | 1975.61717 |
| Deaths | Global | Male | 60-64 years | Colon and rectum cancer | High fasting plasma glucose | Rate | 1994 | 2.753991943 | 4.129363138 | 1.389679218 |
| Deaths | Global | Female | 60-64 years | Colon and rectum cancer | High fasting plasma glucose | Rate | 1994 | 1.836712781 | 2.765556101 | 0.928279536 |
| Deaths | Global | Both | 60-64 years | Colon and rectum cancer | High fasting plasma glucose | Rate | 1994 | 2.288092208 | 3.435087567 | 1.162712152 |
| Deaths | Global | Male | 60-64 years | Colon and rectum cancer | High fasting plasma glucose | Number | 1995 | 2328.608703 | 3521.322415 | 1155.719576 |
| Deaths | Global | Female | 60-64 years | Colon and rectum cancer | High fasting plasma glucose | Number | 1995 | 1586.290281 | 2399.546009 | 790.3941558 |
| Deaths | Global | Both | 60-64 years | Colon and rectum cancer | High fasting plasma glucose | Number | 1995 | 3914.898983 | 5912.602965 | 1975.173177 |
| Deaths | Global | Male | 60-64 years | Colon and rectum cancer | High fasting plasma glucose | Rate | 1995 | 2.757422315 | 4.169774422 | 1.368545494 |
| Deaths | Global | Female | 60-64 years | Colon and rectum cancer | High fasting plasma glucose | Rate | 1995 | 1.816183417 | 2.747300241 | 0.904942038 |
| Deaths | Global | Both | 60-64 years | Colon and rectum cancer | High fasting plasma glucose | Rate | 1995 | 2.278876927 | 3.441747675 | 1.149755485 |
| Deaths | Global | Male | 60-64 years | Colon and rectum cancer | High fasting plasma glucose | Number | 1996 | 2358.831876 | 3583.019372 | 1181.984788 |
| Deaths | Global | Female | 60-64 years | Colon and rectum cancer | High fasting plasma glucose | Number | 1996 | 1599.104484 | 2412.70633 | 810.351163 |
| Deaths | Global | Both | 60-64 years | Colon and rectum cancer | High fasting plasma glucose | Number | 1996 | 3957.936361 | 6012.582046 | 2026.521861 |
| Deaths | Global | Male | 60-64 years | Colon and rectum cancer | High fasting plasma glucose | Rate | 1996 | 2.756939852 | 4.187737581 | 1.381472329 |
| Deaths | Global | Female | 60-64 years | Colon and rectum cancer | High fasting plasma glucose | Rate | 1996 | 1.802002736 | 2.718836356 | 0.913170482 |
| Deaths | Global | Both | 60-64 years | Colon and rectum cancer | High fasting plasma glucose | Rate | 1996 | 2.270758488 | 3.449555645 | 1.162661876 |
| Deaths | Global | Male | 60-64 years | Colon and rectum cancer | High fasting plasma glucose | Number | 1997 | 2409.963881 | 3620.723054 | 1223.613985 |
| Deaths | Global | Female | 60-64 years | Colon and rectum cancer | High fasting plasma glucose | Number | 1997 | 1631.667859 | 2465.503614 | 822.9585817 |
| Deaths | Global | Both | 60-64 years | Colon and rectum cancer | High fasting plasma glucose | Number | 1997 | 4041.63174 | 6087.537917 | 2063.480037 |
| Deaths | Global | Male | 60-64 years | Colon and rectum cancer | High fasting plasma glucose | Rate | 1997 | 2.771227122 | 4.163483946 | 1.407038624 |
| Deaths | Global | Female | 60-64 years | Colon and rectum cancer | High fasting plasma glucose | Rate | 1997 | 1.802071679 | 2.722989372 | 0.908904558 |
| Deaths | Global | Both | 60-64 years | Colon and rectum cancer | High fasting plasma glucose | Rate | 1997 | 2.276875765 | 3.429448411 | 1.162472979 |
| Deaths | Global | Male | 60-64 years | Colon and rectum cancer | High fasting plasma glucose | Number | 1998 | 2482.270899 | 3725.969686 | 1260.47012 |
| Deaths | Global | Female | 60-64 years | Colon and rectum cancer | High fasting plasma glucose | Number | 1998 | 1673.65039 | 2510.265254 | 837.3577186 |
| Deaths | Global | Both | 60-64 years | Colon and rectum cancer | High fasting plasma glucose | Number | 1998 | 4155.921289 | 6189.501052 | 2098.608304 |
| Deaths | Global | Male | 60-64 years | Colon and rectum cancer | High fasting plasma glucose | Rate | 1998 | 2.808295104 | 4.215342665 | 1.42602166 |
| Deaths | Global | Female | 60-64 years | Colon and rectum cancer | High fasting plasma glucose | Rate | 1998 | 1.813471829 | 2.719979841 | 0.907312926 |
| Deaths | Global | Both | 60-64 years | Colon and rectum cancer | High fasting plasma glucose | Rate | 1998 | 2.300149074 | 3.42566043 | 1.161502254 |
| Deaths | Global | Male | 60-64 years | Colon and rectum cancer | High fasting plasma glucose | Number | 1999 | 2556.85377 | 3783.1675 | 1269.350783 |
| Deaths | Global | Female | 60-64 years | Colon and rectum cancer | High fasting plasma glucose | Number | 1999 | 1727.150759 | 2578.842789 | 882.1393144 |
| Deaths | Global | Both | 60-64 years | Colon and rectum cancer | High fasting plasma glucose | Number | 1999 | 4284.004529 | 6356.54193 | 2173.558167 |
| Deaths | Global | Male | 60-64 years | Colon and rectum cancer | High fasting plasma glucose | Rate | 1999 | 2.849832383 | 4.216664003 | 1.4148001 |
| Deaths | Global | Female | 60-64 years | Colon and rectum cancer | High fasting plasma glucose | Rate | 1999 | 1.840168434 | 2.747591705 | 0.9398629 |
| Deaths | Global | Both | 60-64 years | Colon and rectum cancer | High fasting plasma glucose | Rate | 1999 | 2.333618716 | 3.462588594 | 1.183998753 |
| Deaths | Global | Male | 60-64 years | Colon and rectum cancer | High fasting plasma glucose | Number | 2000 | 2621.342942 | 3966.840105 | 1308.016408 |
| Deaths | Global | Female | 60-64 years | Colon and rectum cancer | High fasting plasma glucose | Number | 2000 | 1749.33366 | 2647.729495 | 895.131622 |
| Deaths | Global | Both | 60-64 years | Colon and rectum cancer | High fasting plasma glucose | Number | 2000 | 4370.676602 | 6598.824777 | 2210.561723 |
| Deaths | Global | Male | 60-64 years | Colon and rectum cancer | High fasting plasma glucose | Rate | 2000 | 2.881732368 | 4.360883632 | 1.437947382 |
| Deaths | Global | Female | 60-64 years | Colon and rectum cancer | High fasting plasma glucose | Rate | 2000 | 1.834318571 | 2.776359648 | 0.938618284 |
| Deaths | Global | Both | 60-64 years | Colon and rectum cancer | High fasting plasma glucose | Rate | 2000 | 2.34565081 | 3.54145138 | 1.186362289 |
| Deaths | Global | Male | 60-64 years | Colon and rectum cancer | High fasting plasma glucose | Number | 2001 | 2664.945585 | 4008.458728 | 1344.355053 |
| Deaths | Global | Female | 60-64 years | Colon and rectum cancer | High fasting plasma glucose | Number | 2001 | 1761.174175 | 2638.816579 | 894.3651299 |
| Deaths | Global | Both | 60-64 years | Colon and rectum cancer | High fasting plasma glucose | Number | 2001 | 4426.11976 | 6590.352867 | 2266.584645 |
| Deaths | Global | Male | 60-64 years | Colon and rectum cancer | High fasting plasma glucose | Rate | 2001 | 2.897330037 | 4.35799813 | 1.461583417 |
| Deaths | Global | Female | 60-64 years | Colon and rectum cancer | High fasting plasma glucose | Rate | 2001 | 1.826036383 | 2.736001441 | 0.927303665 |
| Deaths | Global | Both | 60-64 years | Colon and rectum cancer | High fasting plasma glucose | Rate | 2001 | 2.348980312 | 3.497557674 | 1.202896215 |
| Deaths | Global | Male | 60-64 years | Colon and rectum cancer | High fasting plasma glucose | Number | 2002 | 2703.771948 | 4017.958028 | 1372.440828 |
| Deaths | Global | Female | 60-64 years | Colon and rectum cancer | High fasting plasma glucose | Number | 2002 | 1770.259077 | 2673.869328 | 901.9094782 |
| Deaths | Global | Both | 60-64 years | Colon and rectum cancer | High fasting plasma glucose | Number | 2002 | 4474.031024 | 6690.264063 | 2287.761788 |
| Deaths | Global | Male | 60-64 years | Colon and rectum cancer | High fasting plasma glucose | Rate | 2002 | 2.915881806 | 4.333165274 | 1.480108278 |
| Deaths | Global | Female | 60-64 years | Colon and rectum cancer | High fasting plasma glucose | Rate | 2002 | 1.822711653 | 2.753095773 | 0.928632954 |
| Deaths | Global | Both | 60-64 years | Colon and rectum cancer | High fasting plasma glucose | Rate | 2002 | 2.3566387 | 3.524011147 | 1.205049302 |
| Deaths | Global | Male | 60-64 years | Colon and rectum cancer | High fasting plasma glucose | Number | 2003 | 2738.89193 | 4048.59481 | 1394.080833 |
| Deaths | Global | Female | 60-64 years | Colon and rectum cancer | High fasting plasma glucose | Number | 2003 | 1783.716469 | 2674.900946 | 907.012633 |
| Deaths | Global | Both | 60-64 years | Colon and rectum cancer | High fasting plasma glucose | Number | 2003 | 4522.608399 | 6718.354527 | 2302.310091 |
| Deaths | Global | Male | 60-64 years | Colon and rectum cancer | High fasting plasma glucose | Rate | 2003 | 2.927985828 | 4.328110976 | 1.490328581 |
| Deaths | Global | Female | 60-64 years | Colon and rectum cancer | High fasting plasma glucose | Rate | 2003 | 1.827898745 | 2.741157672 | 0.929479142 |
| Deaths | Global | Both | 60-64 years | Colon and rectum cancer | High fasting plasma glucose | Rate | 2003 | 2.366312433 | 3.515167452 | 1.204611258 |
| Deaths | Global | Male | 60-64 years | Colon and rectum cancer | High fasting plasma glucose | Number | 2004 | 2762.228796 | 4098.20765 | 1388.439731 |
| Deaths | Global | Female | 60-64 years | Colon and rectum cancer | High fasting plasma glucose | Number | 2004 | 1773.436362 | 2673.814334 | 911.3625619 |
| Deaths | Global | Both | 60-64 years | Colon and rectum cancer | High fasting plasma glucose | Number | 2004 | 4535.665158 | 6773.055949 | 2290.213453 |
| Deaths | Global | Male | 60-64 years | Colon and rectum cancer | High fasting plasma glucose | Rate | 2004 | 2.925109318 | 4.339866922 | 1.470311945 |
| Deaths | Global | Female | 60-64 years | Colon and rectum cancer | High fasting plasma glucose | Rate | 2004 | 1.80476384 | 2.721046849 | 0.92746164 |
| Deaths | Global | Both | 60-64 years | Colon and rectum cancer | High fasting plasma glucose | Rate | 2004 | 2.35379528 | 3.514895074 | 1.188512253 |
| Deaths | Global | Male | 60-64 years | Colon and rectum cancer | High fasting plasma glucose | Number | 2005 | 2784.44041 | 4121.087727 | 1423.350687 |
| Deaths | Global | Female | 60-64 years | Colon and rectum cancer | High fasting plasma glucose | Number | 2005 | 1765.818176 | 2649.601203 | 905.6726499 |
| Deaths | Global | Both | 60-64 years | Colon and rectum cancer | High fasting plasma glucose | Number | 2005 | 4550.258586 | 6740.89519 | 2337.663786 |
| Deaths | Global | Male | 60-64 years | Colon and rectum cancer | High fasting plasma glucose | Rate | 2005 | 2.919063608 | 4.320335664 | 1.492167395 |
| Deaths | Global | Female | 60-64 years | Colon and rectum cancer | High fasting plasma glucose | Rate | 2005 | 1.785919141 | 2.6797626 | 0.915982259 |
| Deaths | Global | Both | 60-64 years | Colon and rectum cancer | High fasting plasma glucose | Rate | 2005 | 2.342323376 | 3.469991008 | 1.203352387 |
| Deaths | Global | Male | 60-64 years | Colon and rectum cancer | High fasting plasma glucose | Number | 2006 | 2807.180802 | 4151.307891 | 1456.949529 |
| Deaths | Global | Female | 60-64 years | Colon and rectum cancer | High fasting plasma glucose | Number | 2006 | 1767.616016 | 2651.704136 | 908.6657253 |
| Deaths | Global | Both | 60-64 years | Colon and rectum cancer | High fasting plasma glucose | Number | 2006 | 4574.796818 | 6794.793112 | 2355.527655 |
| Deaths | Global | Male | 60-64 years | Colon and rectum cancer | High fasting plasma glucose | Rate | 2006 | 2.884833463 | 4.266141998 | 1.497251888 |
| Deaths | Global | Female | 60-64 years | Colon and rectum cancer | High fasting plasma glucose | Rate | 2006 | 1.756430318 | 2.634923816 | 0.902915574 |
| Deaths | Global | Both | 60-64 years | Colon and rectum cancer | High fasting plasma glucose | Rate | 2006 | 2.31114441 | 3.432665698 | 1.18999046 |
| Deaths | Global | Male | 60-64 years | Colon and rectum cancer | High fasting plasma glucose | Number | 2007 | 2947.447069 | 4340.411974 | 1495.877943 |
| Deaths | Global | Female | 60-64 years | Colon and rectum cancer | High fasting plasma glucose | Number | 2007 | 1835.494893 | 2712.360255 | 943.800067 |
| Deaths | Global | Both | 60-64 years | Colon and rectum cancer | High fasting plasma glucose | Number | 2007 | 4782.941962 | 7075.851479 | 2436.545045 |
| Deaths | Global | Male | 60-64 years | Colon and rectum cancer | High fasting plasma glucose | Rate | 2007 | 2.90206306 | 4.27357946 | 1.472844811 |
| Deaths | Global | Female | 60-64 years | Colon and rectum cancer | High fasting plasma glucose | Rate | 2007 | 1.748830896 | 2.584294532 | 0.899237979 |
| Deaths | Global | Both | 60-64 years | Colon and rectum cancer | High fasting plasma glucose | Rate | 2007 | 2.315977163 | 3.426240703 | 1.179814166 |
| Deaths | Global | Male | 60-64 years | Colon and rectum cancer | High fasting plasma glucose | Number | 2008 | 3126.086223 | 4665.091132 | 1601.168474 |
| Deaths | Global | Female | 60-64 years | Colon and rectum cancer | High fasting plasma glucose | Number | 2008 | 1920.76069 | 2837.368082 | 990.9574349 |
| Deaths | Global | Both | 60-64 years | Colon and rectum cancer | High fasting plasma glucose | Number | 2008 | 5046.846912 | 7506.586403 | 2588.086567 |
| Deaths | Global | Male | 60-64 years | Colon and rectum cancer | High fasting plasma glucose | Rate | 2008 | 2.948435784 | 4.399981527 | 1.510176652 |
| Deaths | Global | Female | 60-64 years | Colon and rectum cancer | High fasting plasma glucose | Rate | 2008 | 1.75125581 | 2.586973675 | 0.903506603 |
| Deaths | Global | Both | 60-64 years | Colon and rectum cancer | High fasting plasma glucose | Rate | 2008 | 2.339706313 | 3.480035733 | 1.199830822 |
| Deaths | Global | Male | 60-64 years | Colon and rectum cancer | High fasting plasma glucose | Number | 2009 | 3352.128023 | 5039.692614 | 1731.992915 |
| Deaths | Global | Female | 60-64 years | Colon and rectum cancer | High fasting plasma glucose | Number | 2009 | 2029.166956 | 3024.223055 | 1047.063352 |
| Deaths | Global | Both | 60-64 years | Colon and rectum cancer | High fasting plasma glucose | Number | 2009 | 5381.29498 | 8045.631892 | 2756.705041 |
| Deaths | Global | Male | 60-64 years | Colon and rectum cancer | High fasting plasma glucose | Rate | 2009 | 2.988567913 | 4.493105137 | 1.544147006 |
| Deaths | Global | Female | 60-64 years | Colon and rectum cancer | High fasting plasma glucose | Rate | 2009 | 1.747825467 | 2.604918268 | 0.901889313 |
| Deaths | Global | Both | 60-64 years | Colon and rectum cancer | High fasting plasma glucose | Rate | 2009 | 2.357511277 | 3.524740418 | 1.207695034 |
| Deaths | Global | Male | 60-64 years | Colon and rectum cancer | High fasting plasma glucose | Number | 2010 | 3574.164768 | 5369.468431 | 1821.306132 |
| Deaths | Global | Female | 60-64 years | Colon and rectum cancer | High fasting plasma glucose | Number | 2010 | 2150.29844 | 3202.286285 | 1101.074844 |
| Deaths | Global | Both | 60-64 years | Colon and rectum cancer | High fasting plasma glucose | Number | 2010 | 5724.463208 | 8614.171402 | 2916.75221 |
| Deaths | Global | Male | 60-64 years | Colon and rectum cancer | High fasting plasma glucose | Rate | 2010 | 3.054703195 | 4.589081209 | 1.556601338 |
| Deaths | Global | Female | 60-64 years | Colon and rectum cancer | High fasting plasma glucose | Rate | 2010 | 1.768170989 | 2.633211093 | 0.905403901 |
| Deaths | Global | Both | 60-64 years | Colon and rectum cancer | High fasting plasma glucose | Rate | 2010 | 2.399019776 | 3.610044609 | 1.222358495 |
| Deaths | Global | Male | 60-64 years | Colon and rectum cancer | High fasting plasma glucose | Number | 2011 | 3805.908706 | 5634.615398 | 1947.288901 |
| Deaths | Global | Female | 60-64 years | Colon and rectum cancer | High fasting plasma glucose | Number | 2011 | 2290.230861 | 3398.90088 | 1170.291389 |
| Deaths | Global | Both | 60-64 years | Colon and rectum cancer | High fasting plasma glucose | Number | 2011 | 6096.139568 | 9119.811512 | 3128.374557 |
| Deaths | Global | Male | 60-64 years | Colon and rectum cancer | High fasting plasma glucose | Rate | 2011 | 3.095634968 | 4.583061183 | 1.583878143 |
| Deaths | Global | Female | 60-64 years | Colon and rectum cancer | High fasting plasma glucose | Rate | 2011 | 1.784500314 | 2.648352962 | 0.91186674 |
| Deaths | Global | Both | 60-64 years | Colon and rectum cancer | High fasting plasma glucose | Rate | 2011 | 2.425990694 | 3.629276793 | 1.244953053 |
| Deaths | Global | Male | 60-64 years | Colon and rectum cancer | High fasting plasma glucose | Number | 2012 | 3957.442416 | 5916.782676 | 2077.703785 |
| Deaths | Global | Female | 60-64 years | Colon and rectum cancer | High fasting plasma glucose | Number | 2012 | 2366.377589 | 3494.918648 | 1212.443411 |
| Deaths | Global | Both | 60-64 years | Colon and rectum cancer | High fasting plasma glucose | Number | 2012 | 6323.820004 | 9500.352122 | 3279.81281 |
| Deaths | Global | Male | 60-64 years | Colon and rectum cancer | High fasting plasma glucose | Rate | 2012 | 3.110914942 | 4.6511372 | 1.633266911 |
| Deaths | Global | Female | 60-64 years | Colon and rectum cancer | High fasting plasma glucose | Rate | 2012 | 1.776025773 | 2.623024163 | 0.909969211 |
| Deaths | Global | Both | 60-64 years | Colon and rectum cancer | High fasting plasma glucose | Rate | 2012 | 2.428021408 | 3.647646252 | 1.259279314 |
| Deaths | Global | Male | 60-64 years | Colon and rectum cancer | High fasting plasma glucose | Number | 2013 | 4101.620951 | 6127.029923 | 2170.265282 |
| Deaths | Global | Female | 60-64 years | Colon and rectum cancer | High fasting plasma glucose | Number | 2013 | 2445.890457 | 3626.424048 | 1253.098584 |
| Deaths | Global | Both | 60-64 years | Colon and rectum cancer | High fasting plasma glucose | Number | 2013 | 6547.511408 | 9766.673399 | 3433.720995 |
| Deaths | Global | Male | 60-64 years | Colon and rectum cancer | High fasting plasma glucose | Rate | 2013 | 3.109472546 | 4.64495173 | 1.645295944 |
| Deaths | Global | Female | 60-64 years | Colon and rectum cancer | High fasting plasma glucose | Rate | 2013 | 1.76387693 | 2.615229843 | 0.903683841 |
| Deaths | Global | Both | 60-64 years | Colon and rectum cancer | High fasting plasma glucose | Rate | 2013 | 2.419869751 | 3.609627544 | 1.269055837 |
| Deaths | Global | Male | 60-64 years | Colon and rectum cancer | High fasting plasma glucose | Number | 2014 | 4179.311263 | 6323.690717 | 2165.956143 |
| Deaths | Global | Female | 60-64 years | Colon and rectum cancer | High fasting plasma glucose | Number | 2014 | 2515.375153 | 3762.370338 | 1278.116925 |
| Deaths | Global | Both | 60-64 years | Colon and rectum cancer | High fasting plasma glucose | Number | 2014 | 6694.686416 | 10102.39305 | 3452.57029 |
| Deaths | Global | Male | 60-64 years | Colon and rectum cancer | High fasting plasma glucose | Rate | 2014 | 3.078646407 | 4.658281349 | 1.595529186 |
| Deaths | Global | Female | 60-64 years | Colon and rectum cancer | High fasting plasma glucose | Rate | 2014 | 1.757154587 | 2.628262543 | 0.892848534 |
| Deaths | Global | Both | 60-64 years | Colon and rectum cancer | High fasting plasma glucose | Rate | 2014 | 2.400371808 | 3.622200945 | 1.237914949 |
| Deaths | Global | Male | 60-64 years | Colon and rectum cancer | High fasting plasma glucose | Number | 2015 | 4366.509191 | 6554.367533 | 2241.339691 |
| Deaths | Global | Female | 60-64 years | Colon and rectum cancer | High fasting plasma glucose | Number | 2015 | 2635.49059 | 3913.245484 | 1342.029043 |
| Deaths | Global | Both | 60-64 years | Colon and rectum cancer | High fasting plasma glucose | Number | 2015 | 7001.999781 | 10487.76843 | 3589.001003 |
| Deaths | Global | Male | 60-64 years | Colon and rectum cancer | High fasting plasma glucose | Rate | 2015 | 3.088626262 | 4.636195828 | 1.585399304 |
| Deaths | Global | Female | 60-64 years | Colon and rectum cancer | High fasting plasma glucose | Rate | 2015 | 1.766871019 | 2.623496386 | 0.899715686 |
| Deaths | Global | Both | 60-64 years | Colon and rectum cancer | High fasting plasma glucose | Rate | 2015 | 2.410034198 | 3.609808821 | 1.235306402 |
| Deaths | Global | Male | 60-64 years | Colon and rectum cancer | High fasting plasma glucose | Number | 2016 | 4506.584746 | 6765.110246 | 2351.860734 |
| Deaths | Global | Female | 60-64 years | Colon and rectum cancer | High fasting plasma glucose | Number | 2016 | 2725.128487 | 4057.136067 | 1384.810144 |
| Deaths | Global | Both | 60-64 years | Colon and rectum cancer | High fasting plasma glucose | Number | 2016 | 7231.713233 | 10794.54866 | 3748.784844 |
| Deaths | Global | Male | 60-64 years | Colon and rectum cancer | High fasting plasma glucose | Rate | 2016 | 3.105802016 | 4.662309538 | 1.620831344 |
| Deaths | Global | Female | 60-64 years | Colon and rectum cancer | High fasting plasma glucose | Rate | 2016 | 1.781070734 | 2.651635088 | 0.90507469 |
| Deaths | Global | Both | 60-64 years | Colon and rectum cancer | High fasting plasma glucose | Rate | 2016 | 2.425876794 | 3.621029243 | 1.257529145 |
| Deaths | Global | Male | 60-64 years | Colon and rectum cancer | High fasting plasma glucose | Number | 2017 | 4595.32998 | 6833.68035 | 2350.407208 |
| Deaths | Global | Female | 60-64 years | Colon and rectum cancer | High fasting plasma glucose | Number | 2017 | 2800.554862 | 4169.025216 | 1410.357023 |
| Deaths | Global | Both | 60-64 years | Colon and rectum cancer | High fasting plasma glucose | Number | 2017 | 7395.884842 | 11042.8158 | 3769.639131 |
| Deaths | Global | Male | 60-64 years | Colon and rectum cancer | High fasting plasma glucose | Rate | 2017 | 3.099552291 | 4.609320697 | 1.585350797 |
| Deaths | Global | Female | 60-64 years | Colon and rectum cancer | High fasting plasma glucose | Rate | 2017 | 1.792606084 | 2.668549747 | 0.902754885 |
| Deaths | Global | Both | 60-64 years | Colon and rectum cancer | High fasting plasma glucose | Rate | 2017 | 2.428973798 | 3.626707394 | 1.2380337 |
| Deaths | Global | Male | 60-64 years | Colon and rectum cancer | High fasting plasma glucose | Number | 2018 | 4698.013257 | 7101.653905 | 2392.589638 |
| Deaths | Global | Female | 60-64 years | Colon and rectum cancer | High fasting plasma glucose | Number | 2018 | 2874.136017 | 4328.023428 | 1444.031452 |
| Deaths | Global | Both | 60-64 years | Colon and rectum cancer | High fasting plasma glucose | Number | 2018 | 7572.149274 | 11547.70038 | 3869.460781 |
| Deaths | Global | Male | 60-64 years | Colon and rectum cancer | High fasting plasma glucose | Rate | 2018 | 3.121777406 | 4.718969806 | 1.589849127 |
| Deaths | Global | Female | 60-64 years | Colon and rectum cancer | High fasting plasma glucose | Rate | 2018 | 1.813016589 | 2.73013463 | 0.910900862 |
| Deaths | Global | Both | 60-64 years | Colon and rectum cancer | High fasting plasma glucose | Rate | 2018 | 2.45037947 | 3.736884591 | 1.252173843 |
| Deaths | Global | Male | 60-64 years | Colon and rectum cancer | High fasting plasma glucose | Number | 2019 | 4769.818614 | 7233.676264 | 2450.108672 |
| Deaths | Global | Female | 60-64 years | Colon and rectum cancer | High fasting plasma glucose | Number | 2019 | 2922.257178 | 4401.697597 | 1496.534652 |
| Deaths | Global | Both | 60-64 years | Colon and rectum cancer | High fasting plasma glucose | Number | 2019 | 7692.075792 | 11586.06042 | 3978.807528 |
| Deaths | Global | Male | 60-64 years | Colon and rectum cancer | High fasting plasma glucose | Rate | 2019 | 3.136669519 | 4.756921319 | 1.611210365 |
| Deaths | Global | Female | 60-64 years | Colon and rectum cancer | High fasting plasma glucose | Rate | 2019 | 1.823651978 | 2.746905572 | 0.933921353 |
| Deaths | Global | Both | 60-64 years | Colon and rectum cancer | High fasting plasma glucose | Rate | 2019 | 2.46297447 | 3.709814074 | 1.273999585 |
| Deaths | Global | Male | 60-64 years | Colon and rectum cancer | High fasting plasma glucose | Number | 2020 | 4745.214777 | 7243.092894 | 2434.222708 |
| Deaths | Global | Female | 60-64 years | Colon and rectum cancer | High fasting plasma glucose | Number | 2020 | 2922.837201 | 4395.962711 | 1458.039764 |
| Deaths | Global | Both | 60-64 years | Colon and rectum cancer | High fasting plasma glucose | Number | 2020 | 7668.051978 | 11705.84485 | 3930.705622 |
| Deaths | Global | Male | 60-64 years | Colon and rectum cancer | High fasting plasma glucose | Rate | 2020 | 3.091406195 | 4.7187205 | 1.585844164 |
| Deaths | Global | Female | 60-64 years | Colon and rectum cancer | High fasting plasma glucose | Rate | 2020 | 1.804836196 | 2.714483248 | 0.900331685 |
| Deaths | Global | Both | 60-64 years | Colon and rectum cancer | High fasting plasma glucose | Rate | 2020 | 2.430893537 | 3.710937624 | 1.246095738 |
| Deaths | Global | Male | 60-64 years | Colon and rectum cancer | High fasting plasma glucose | Number | 2021 | 4735.815743 | 7154.073595 | 2414.312052 |
| Deaths | Global | Female | 60-64 years | Colon and rectum cancer | High fasting plasma glucose | Number | 2021 | 2951.694445 | 4410.648982 | 1486.831366 |
| Deaths | Global | Both | 60-64 years | Colon and rectum cancer | High fasting plasma glucose | Number | 2021 | 7687.510188 | 11685.54239 | 3966.892664 |
| Deaths | Global | Male | 60-64 years | Colon and rectum cancer | High fasting plasma glucose | Rate | 2021 | 3.04481774 | 4.599598332 | 1.552243703 |
| Deaths | Global | Female | 60-64 years | Colon and rectum cancer | High fasting plasma glucose | Rate | 2021 | 1.79422388 | 2.681067393 | 0.903788788 |
| Deaths | Global | Both | 60-64 years | Colon and rectum cancer | High fasting plasma glucose | Rate | 2021 | 2.401987737 | 3.651185994 | 1.239468605 |
| Deaths | Global | Male | 65-69 years | Colon and rectum cancer | High fasting plasma glucose | Number | 1990 | 2648.436799 | 3932.937259 | 1305.172004 |
| Deaths | Global | Female | 65-69 years | Colon and rectum cancer | High fasting plasma glucose | Number | 1990 | 2030.60342 | 3096.143849 | 1003.245447 |
| Deaths | Global | Both | 65-69 years | Colon and rectum cancer | High fasting plasma glucose | Number | 1990 | 4679.040218 | 7007.029833 | 2328.903291 |
| Deaths | Global | Male | 65-69 years | Colon and rectum cancer | High fasting plasma glucose | Rate | 1990 | 4.619537079 | 6.860027586 | 2.276546855 |
| Deaths | Global | Female | 65-69 years | Colon and rectum cancer | High fasting plasma glucose | Rate | 1990 | 3.063755752 | 4.671433347 | 1.513687497 |
| Deaths | Global | Both | 65-69 years | Colon and rectum cancer | High fasting plasma glucose | Rate | 1990 | 3.785341607 | 5.668684245 | 1.884081802 |
| Deaths | Global | Male | 65-69 years | Colon and rectum cancer | High fasting plasma glucose | Number | 1991 | 2752.144599 | 4123.19126 | 1386.813982 |
| Deaths | Global | Female | 65-69 years | Colon and rectum cancer | High fasting plasma glucose | Number | 1991 | 2087.120129 | 3170.179631 | 1035.484931 |
| Deaths | Global | Both | 65-69 years | Colon and rectum cancer | High fasting plasma glucose | Number | 1991 | 4839.264728 | 7224.321761 | 2432.304483 |
| Deaths | Global | Male | 65-69 years | Colon and rectum cancer | High fasting plasma glucose | Rate | 1991 | 4.639662082 | 6.951020725 | 2.337939746 |
| Deaths | Global | Female | 65-69 years | Colon and rectum cancer | High fasting plasma glucose | Rate | 1991 | 3.064260521 | 4.654382924 | 1.52027454 |
| Deaths | Global | Both | 65-69 years | Colon and rectum cancer | High fasting plasma glucose | Rate | 1991 | 3.79760197 | 5.669270042 | 1.908745401 |
| Deaths | Global | Male | 65-69 years | Colon and rectum cancer | High fasting plasma glucose | Number | 1992 | 2882.535141 | 4295.835302 | 1450.671109 |
| Deaths | Global | Female | 65-69 years | Colon and rectum cancer | High fasting plasma glucose | Number | 1992 | 2143.214909 | 3265.825147 | 1048.370179 |
| Deaths | Global | Both | 65-69 years | Colon and rectum cancer | High fasting plasma glucose | Number | 1992 | 5025.750049 | 7586.860139 | 2516.165429 |
| Deaths | Global | Male | 65-69 years | Colon and rectum cancer | High fasting plasma glucose | Rate | 1992 | 4.693745429 | 6.995077711 | 2.362184867 |
| Deaths | Global | Female | 65-69 years | Colon and rectum cancer | High fasting plasma glucose | Rate | 1992 | 3.067260541 | 4.67388341 | 1.50037426 |
| Deaths | Global | Both | 65-69 years | Colon and rectum cancer | High fasting plasma glucose | Rate | 1992 | 3.828087913 | 5.77887227 | 1.916550241 |
| Deaths | Global | Male | 65-69 years | Colon and rectum cancer | High fasting plasma glucose | Number | 1993 | 3024.216153 | 4525.682418 | 1519.987242 |
| Deaths | Global | Female | 65-69 years | Colon and rectum cancer | High fasting plasma glucose | Number | 1993 | 2212.47394 | 3346.393782 | 1094.678345 |
| Deaths | Global | Both | 65-69 years | Colon and rectum cancer | High fasting plasma glucose | Number | 1993 | 5236.690093 | 7867.638477 | 2614.665587 |
| Deaths | Global | Male | 65-69 years | Colon and rectum cancer | High fasting plasma glucose | Rate | 1993 | 4.753941888 | 7.114184348 | 2.389356664 |
| Deaths | Global | Female | 65-69 years | Colon and rectum cancer | High fasting plasma glucose | Rate | 1993 | 3.093202464 | 4.678506402 | 1.530441418 |
| Deaths | Global | Both | 65-69 years | Colon and rectum cancer | High fasting plasma glucose | Rate | 1993 | 3.874957135 | 5.821761707 | 1.934755903 |
| Deaths | Global | Male | 65-69 years | Colon and rectum cancer | High fasting plasma glucose | Number | 1994 | 3153.676745 | 4752.707381 | 1601.050373 |
| Deaths | Global | Female | 65-69 years | Colon and rectum cancer | High fasting plasma glucose | Number | 1994 | 2267.255515 | 3425.907352 | 1112.371081 |
| Deaths | Global | Both | 65-69 years | Colon and rectum cancer | High fasting plasma glucose | Number | 1994 | 5420.93226 | 8195.825506 | 2717.360002 |
| Deaths | Global | Male | 65-69 years | Colon and rectum cancer | High fasting plasma glucose | Rate | 1994 | 4.799096142 | 7.232415211 | 2.436392595 |
| Deaths | Global | Female | 65-69 years | Colon and rectum cancer | High fasting plasma glucose | Rate | 1994 | 3.100290163 | 4.684653666 | 1.52107828 |
| Deaths | Global | Both | 65-69 years | Colon and rectum cancer | High fasting plasma glucose | Rate | 1994 | 3.904321785 | 5.902885064 | 1.957126071 |
| Deaths | Global | Male | 65-69 years | Colon and rectum cancer | High fasting plasma glucose | Number | 1995 | 3267.677234 | 4864.534264 | 1663.846343 |
| Deaths | Global | Female | 65-69 years | Colon and rectum cancer | High fasting plasma glucose | Number | 1995 | 2302.46235 | 3467.024315 | 1135.112677 |
| Deaths | Global | Both | 65-69 years | Colon and rectum cancer | High fasting plasma glucose | Number | 1995 | 5570.139584 | 8342.976111 | 2798.764503 |
| Deaths | Global | Male | 65-69 years | Colon and rectum cancer | High fasting plasma glucose | Rate | 1995 | 4.838072473 | 7.202354343 | 2.463465212 |
| Deaths | Global | Female | 65-69 years | Colon and rectum cancer | High fasting plasma glucose | Rate | 1995 | 3.091900502 | 4.655752231 | 1.524305253 |
| Deaths | Global | Both | 65-69 years | Colon and rectum cancer | High fasting plasma glucose | Rate | 1995 | 3.922400541 | 5.874986348 | 1.970843861 |
| Deaths | Global | Male | 65-69 years | Colon and rectum cancer | High fasting plasma glucose | Number | 1996 | 3370.794432 | 5132.779003 | 1727.788508 |
| Deaths | Global | Female | 65-69 years | Colon and rectum cancer | High fasting plasma glucose | Number | 1996 | 2331.979437 | 3549.716378 | 1165.377772 |
| Deaths | Global | Both | 65-69 years | Colon and rectum cancer | High fasting plasma glucose | Number | 1996 | 5702.77387 | 8612.458775 | 2889.819723 |
| Deaths | Global | Male | 65-69 years | Colon and rectum cancer | High fasting plasma glucose | Rate | 1996 | 4.876195827 | 7.425085112 | 2.499421214 |
| Deaths | Global | Female | 65-69 years | Colon and rectum cancer | High fasting plasma glucose | Rate | 1996 | 3.079075711 | 4.686939047 | 1.538729859 |
| Deaths | Global | Both | 65-69 years | Colon and rectum cancer | High fasting plasma glucose | Rate | 1996 | 3.93664277 | 5.945207428 | 1.994851659 |
| Deaths | Global | Male | 65-69 years | Colon and rectum cancer | High fasting plasma glucose | Number | 1997 | 3465.629373 | 5213.252876 | 1759.861923 |
| Deaths | Global | Female | 65-69 years | Colon and rectum cancer | High fasting plasma glucose | Number | 1997 | 2375.044626 | 3604.553251 | 1178.6099 |
| Deaths | Global | Both | 65-69 years | Colon and rectum cancer | High fasting plasma glucose | Number | 1997 | 5840.673999 | 8893.243826 | 2933.397786 |
| Deaths | Global | Male | 65-69 years | Colon and rectum cancer | High fasting plasma glucose | Rate | 1997 | 4.931755563 | 7.418706995 | 2.504367286 |
| Deaths | Global | Female | 65-69 years | Colon and rectum cancer | High fasting plasma glucose | Rate | 1997 | 3.096405727 | 4.699347207 | 1.536583526 |
| Deaths | Global | Both | 65-69 years | Colon and rectum cancer | High fasting plasma glucose | Rate | 1997 | 3.973923616 | 6.050855033 | 1.995848208 |
| Deaths | Global | Male | 65-69 years | Colon and rectum cancer | High fasting plasma glucose | Number | 1998 | 3567.417685 | 5310.271308 | 1823.129294 |
| Deaths | Global | Female | 65-69 years | Colon and rectum cancer | High fasting plasma glucose | Number | 1998 | 2435.696387 | 3680.755625 | 1205.636123 |
| Deaths | Global | Both | 65-69 years | Colon and rectum cancer | High fasting plasma glucose | Number | 1998 | 6003.114073 | 9035.413244 | 3023.434798 |
| Deaths | Global | Male | 65-69 years | Colon and rectum cancer | High fasting plasma glucose | Rate | 1998 | 5.013814958 | 7.463302607 | 2.562310817 |
| Deaths | Global | Female | 65-69 years | Colon and rectum cancer | High fasting plasma glucose | Rate | 1998 | 3.13705578 | 4.74063014 | 1.55279935 |
| Deaths | Global | Both | 65-69 years | Colon and rectum cancer | High fasting plasma glucose | Rate | 1998 | 4.034499645 | 6.072410267 | 2.031953162 |
| Deaths | Global | Male | 65-69 years | Colon and rectum cancer | High fasting plasma glucose | Number | 1999 | 3653.802373 | 5470.490146 | 1871.256324 |
| Deaths | Global | Female | 65-69 years | Colon and rectum cancer | High fasting plasma glucose | Number | 1999 | 2495.708723 | 3741.973314 | 1239.687325 |
| Deaths | Global | Both | 65-69 years | Colon and rectum cancer | High fasting plasma glucose | Number | 1999 | 6149.511096 | 9219.838381 | 3119.656673 |
| Deaths | Global | Male | 65-69 years | Colon and rectum cancer | High fasting plasma glucose | Rate | 1999 | 5.07507126 | 7.598420626 | 2.599144184 |
| Deaths | Global | Female | 65-69 years | Colon and rectum cancer | High fasting plasma glucose | Rate | 1999 | 3.178892606 | 4.76631395 | 1.579043594 |
| Deaths | Global | Both | 65-69 years | Colon and rectum cancer | High fasting plasma glucose | Rate | 1999 | 4.085949604 | 6.125982114 | 2.072808675 |
| Deaths | Global | Male | 65-69 years | Colon and rectum cancer | High fasting plasma glucose | Number | 2000 | 3752.794714 | 5633.771139 | 1897.308567 |
| Deaths | Global | Female | 65-69 years | Colon and rectum cancer | High fasting plasma glucose | Number | 2000 | 2529.392822 | 3813.715512 | 1265.785422 |
| Deaths | Global | Both | 65-69 years | Colon and rectum cancer | High fasting plasma glucose | Number | 2000 | 6282.187535 | 9419.321547 | 3174.890859 |
| Deaths | Global | Male | 65-69 years | Colon and rectum cancer | High fasting plasma glucose | Rate | 2000 | 5.145577693 | 7.724645047 | 2.601460881 |
| Deaths | Global | Female | 65-69 years | Colon and rectum cancer | High fasting plasma glucose | Rate | 2000 | 3.176681909 | 4.789671643 | 1.589708651 |
| Deaths | Global | Both | 65-69 years | Colon and rectum cancer | High fasting plasma glucose | Rate | 2000 | 4.117950655 | 6.174330377 | 2.081129195 |
| Deaths | Global | Male | 65-69 years | Colon and rectum cancer | High fasting plasma glucose | Number | 2001 | 3821.014973 | 5720.90979 | 1953.544494 |
| Deaths | Global | Female | 65-69 years | Colon and rectum cancer | High fasting plasma glucose | Number | 2001 | 2559.77356 | 3805.904371 | 1275.890444 |
| Deaths | Global | Both | 65-69 years | Colon and rectum cancer | High fasting plasma glucose | Number | 2001 | 6380.788532 | 9506.933544 | 3241.71473 |
| Deaths | Global | Male | 65-69 years | Colon and rectum cancer | High fasting plasma glucose | Rate | 2001 | 5.153430937 | 7.715833021 | 2.634759797 |
| Deaths | Global | Female | 65-69 years | Colon and rectum cancer | High fasting plasma glucose | Rate | 2001 | 3.156249645 | 4.692752713 | 1.573197264 |
| Deaths | Global | Both | 65-69 years | Colon and rectum cancer | High fasting plasma glucose | Rate | 2001 | 4.11009302 | 6.123754298 | 2.088103847 |
| Deaths | Global | Male | 65-69 years | Colon and rectum cancer | High fasting plasma glucose | Number | 2002 | 3901.97581 | 5844.534258 | 1998.794662 |
| Deaths | Global | Female | 65-69 years | Colon and rectum cancer | High fasting plasma glucose | Number | 2002 | 2593.5902 | 3893.846556 | 1283.704375 |
| Deaths | Global | Both | 65-69 years | Colon and rectum cancer | High fasting plasma glucose | Number | 2002 | 6495.56601 | 9780.040951 | 3296.379656 |
| Deaths | Global | Male | 65-69 years | Colon and rectum cancer | High fasting plasma glucose | Rate | 2002 | 5.159784392 | 7.72852988 | 2.643109543 |
| Deaths | Global | Female | 65-69 years | Colon and rectum cancer | High fasting plasma glucose | Rate | 2002 | 3.126709383 | 4.694236801 | 1.547573135 |
| Deaths | Global | Both | 65-69 years | Colon and rectum cancer | High fasting plasma glucose | Rate | 2002 | 4.096278856 | 6.167557207 | 2.078785785 |
| Deaths | Global | Male | 65-69 years | Colon and rectum cancer | High fasting plasma glucose | Number | 2003 | 4010.62158 | 5975.40282 | 2048.594889 |
| Deaths | Global | Female | 65-69 years | Colon and rectum cancer | High fasting plasma glucose | Number | 2003 | 2652.914841 | 4000.728291 | 1328.386819 |
| Deaths | Global | Both | 65-69 years | Colon and rectum cancer | High fasting plasma glucose | Number | 2003 | 6663.536422 | 9851.719974 | 3370.375371 |
| Deaths | Global | Male | 65-69 years | Colon and rectum cancer | High fasting plasma glucose | Rate | 2003 | 5.200516825 | 7.748221137 | 2.656384298 |
| Deaths | Global | Female | 65-69 years | Colon and rectum cancer | High fasting plasma glucose | Rate | 2003 | 3.130558179 | 4.721038339 | 1.567555866 |
| Deaths | Global | Both | 65-69 years | Colon and rectum cancer | High fasting plasma glucose | Rate | 2003 | 4.116795306 | 6.086485011 | 2.082249519 |
| Deaths | Global | Male | 65-69 years | Colon and rectum cancer | High fasting plasma glucose | Number | 2004 | 4065.36032 | 6069.266172 | 2067.274577 |
| Deaths | Global | Female | 65-69 years | Colon and rectum cancer | High fasting plasma glucose | Number | 2004 | 2681.228295 | 4031.945941 | 1336.980319 |
| Deaths | Global | Both | 65-69 years | Colon and rectum cancer | High fasting plasma glucose | Number | 2004 | 6746.588615 | 10137.39439 | 3401.361814 |
| Deaths | Global | Male | 65-69 years | Colon and rectum cancer | High fasting plasma glucose | Rate | 2004 | 5.176917677 | 7.72873469 | 2.632512116 |
| Deaths | Global | Female | 65-69 years | Colon and rectum cancer | High fasting plasma glucose | Rate | 2004 | 3.104537481 | 4.668504848 | 1.548061208 |
| Deaths | Global | Both | 65-69 years | Colon and rectum cancer | High fasting plasma glucose | Rate | 2004 | 4.091484679 | 6.147846889 | 2.062763946 |
| Deaths | Global | Male | 65-69 years | Colon and rectum cancer | High fasting plasma glucose | Number | 2005 | 4119.628129 | 6105.793576 | 2116.598378 |
| Deaths | Global | Female | 65-69 years | Colon and rectum cancer | High fasting plasma glucose | Number | 2005 | 2718.925122 | 4053.844045 | 1344.583306 |
| Deaths | Global | Both | 65-69 years | Colon and rectum cancer | High fasting plasma glucose | Number | 2005 | 6838.553251 | 10211.74928 | 3452.017523 |
| Deaths | Global | Male | 65-69 years | Colon and rectum cancer | High fasting plasma glucose | Rate | 2005 | 5.156952471 | 7.643235331 | 2.649558866 |
| Deaths | Global | Female | 65-69 years | Colon and rectum cancer | High fasting plasma glucose | Rate | 2005 | 3.092402461 | 4.610688688 | 1.52927813 |
| Deaths | Global | Both | 65-69 years | Colon and rectum cancer | High fasting plasma glucose | Rate | 2005 | 4.075232589 | 6.085388522 | 2.05712726 |
| Deaths | Global | Male | 65-69 years | Colon and rectum cancer | High fasting plasma glucose | Number | 2006 | 4108.49596 | 6104.4873 | 2096.868287 |
| Deaths | Global | Female | 65-69 years | Colon and rectum cancer | High fasting plasma glucose | Number | 2006 | 2698.277464 | 4036.378734 | 1346.100224 |
| Deaths | Global | Both | 65-69 years | Colon and rectum cancer | High fasting plasma glucose | Number | 2006 | 6806.773424 | 10163.5852 | 3443.698662 |
| Deaths | Global | Male | 65-69 years | Colon and rectum cancer | High fasting plasma glucose | Rate | 2006 | 5.064214468 | 7.524513399 | 2.584641878 |
| Deaths | Global | Female | 65-69 years | Colon and rectum cancer | High fasting plasma glucose | Rate | 2006 | 3.028060868 | 4.529704842 | 1.51062056 |
| Deaths | Global | Both | 65-69 years | Colon and rectum cancer | High fasting plasma glucose | Rate | 2006 | 3.998407993 | 5.970253124 | 2.022883883 |
| Deaths | Global | Male | 65-69 years | Colon and rectum cancer | High fasting plasma glucose | Number | 2007 | 4147.503288 | 6179.393721 | 2095.686162 |
| Deaths | Global | Female | 65-69 years | Colon and rectum cancer | High fasting plasma glucose | Number | 2007 | 2693.793743 | 4018.421876 | 1345.517846 |
| Deaths | Global | Both | 65-69 years | Colon and rectum cancer | High fasting plasma glucose | Number | 2007 | 6841.297031 | 10171.27075 | 3425.064734 |
| Deaths | Global | Male | 65-69 years | Colon and rectum cancer | High fasting plasma glucose | Rate | 2007 | 5.046154097 | 7.518299752 | 2.549764178 |
| Deaths | Global | Female | 65-69 years | Colon and rectum cancer | High fasting plasma glucose | Rate | 2007 | 2.995230778 | 4.468085544 | 1.49608205 |
| Deaths | Global | Both | 65-69 years | Colon and rectum cancer | High fasting plasma glucose | Rate | 2007 | 3.974552671 | 5.909150141 | 1.989842003 |
| Deaths | Global | Male | 65-69 years | Colon and rectum cancer | High fasting plasma glucose | Number | 2008 | 4254.950927 | 6379.780143 | 2175.53922 |
| Deaths | Global | Female | 65-69 years | Colon and rectum cancer | High fasting plasma glucose | Number | 2008 | 2705.315539 | 4061.804767 | 1343.143795 |
| Deaths | Global | Both | 65-69 years | Colon and rectum cancer | High fasting plasma glucose | Number | 2008 | 6960.266465 | 10460.96491 | 3499.731945 |
| Deaths | Global | Male | 65-69 years | Colon and rectum cancer | High fasting plasma glucose | Rate | 2008 | 5.10406523 | 7.652923515 | 2.609687932 |
| Deaths | Global | Female | 65-69 years | Colon and rectum cancer | High fasting plasma glucose | Rate | 2008 | 2.986200376 | 4.483529832 | 1.482598406 |
| Deaths | Global | Both | 65-69 years | Colon and rectum cancer | High fasting plasma glucose | Rate | 2008 | 4.001122014 | 6.013504974 | 2.011827363 |
| Deaths | Global | Male | 65-69 years | Colon and rectum cancer | High fasting plasma glucose | Number | 2009 | 4352.861857 | 6529.03023 | 2201.941795 |
| Deaths | Global | Female | 65-69 years | Colon and rectum cancer | High fasting plasma glucose | Number | 2009 | 2723.821614 | 4061.311298 | 1367.249755 |
| Deaths | Global | Both | 65-69 years | Colon and rectum cancer | High fasting plasma glucose | Number | 2009 | 7076.683472 | 10598.56265 | 3584.306186 |
| Deaths | Global | Male | 65-69 years | Colon and rectum cancer | High fasting plasma glucose | Rate | 2009 | 5.146327708 | 7.719181147 | 2.603324995 |
| Deaths | Global | Female | 65-69 years | Colon and rectum cancer | High fasting plasma glucose | Rate | 2009 | 2.977929684 | 4.440195131 | 1.494801866 |
| Deaths | Global | Both | 65-69 years | Colon and rectum cancer | High fasting plasma glucose | Rate | 2009 | 4.019727012 | 6.020239388 | 2.035972423 |
| Deaths | Global | Male | 65-69 years | Colon and rectum cancer | High fasting plasma glucose | Number | 2010 | 4468.629257 | 6705.141499 | 2271.846938 |
| Deaths | Global | Female | 65-69 years | Colon and rectum cancer | High fasting plasma glucose | Number | 2010 | 2732.820085 | 4099.556856 | 1359.898211 |
| Deaths | Global | Both | 65-69 years | Colon and rectum cancer | High fasting plasma glucose | Number | 2010 | 7201.449342 | 10825.477 | 3623.671107 |
| Deaths | Global | Male | 65-69 years | Colon and rectum cancer | High fasting plasma glucose | Rate | 2010 | 5.209121287 | 7.816243709 | 2.648312394 |
| Deaths | Global | Female | 65-69 years | Colon and rectum cancer | High fasting plasma glucose | Rate | 2010 | 2.962232362 | 4.443702698 | 1.4740577 |
| Deaths | Global | Both | 65-69 years | Colon and rectum cancer | High fasting plasma glucose | Rate | 2010 | 4.044846157 | 6.08035785 | 2.035311429 |
| Deaths | Global | Male | 65-69 years | Colon and rectum cancer | High fasting plasma glucose | Number | 2011 | 4569.190501 | 6764.58947 | 2305.393373 |
| Deaths | Global | Female | 65-69 years | Colon and rectum cancer | High fasting plasma glucose | Number | 2011 | 2770.973616 | 4149.641187 | 1386.635838 |
| Deaths | Global | Both | 65-69 years | Colon and rectum cancer | High fasting plasma glucose | Number | 2011 | 7340.164117 | 10979.38822 | 3714.310666 |
| Deaths | Global | Male | 65-69 years | Colon and rectum cancer | High fasting plasma glucose | Rate | 2011 | 5.206685225 | 7.708386865 | 2.627042494 |
| Deaths | Global | Female | 65-69 years | Colon and rectum cancer | High fasting plasma glucose | Rate | 2011 | 2.944762066 | 4.409896177 | 1.473602128 |
| Deaths | Global | Both | 65-69 years | Colon and rectum cancer | High fasting plasma glucose | Rate | 2011 | 4.03628148 | 6.037453743 | 2.042461601 |
| Deaths | Global | Male | 65-69 years | Colon and rectum cancer | High fasting plasma glucose | Number | 2012 | 4787.85342 | 7183.264394 | 2429.334247 |
| Deaths | Global | Female | 65-69 years | Colon and rectum cancer | High fasting plasma glucose | Number | 2012 | 2871.916152 | 4348.466143 | 1429.149743 |
| Deaths | Global | Both | 65-69 years | Colon and rectum cancer | High fasting plasma glucose | Number | 2012 | 7659.769571 | 11562.55603 | 3884.921978 |
| Deaths | Global | Male | 65-69 years | Colon and rectum cancer | High fasting plasma glucose | Rate | 2012 | 5.212259536 | 7.820005138 | 2.644675909 |
| Deaths | Global | Female | 65-69 years | Colon and rectum cancer | High fasting plasma glucose | Rate | 2012 | 2.918996728 | 4.419752449 | 1.45257842 |
| Deaths | Global | Both | 65-69 years | Colon and rectum cancer | High fasting plasma glucose | Rate | 2012 | 4.026273568 | 6.077730312 | 2.042066479 |
| Deaths | Global | Male | 65-69 years | Colon and rectum cancer | High fasting plasma glucose | Number | 2013 | 4994.319036 | 7490.607646 | 2540.723558 |
| Deaths | Global | Female | 65-69 years | Colon and rectum cancer | High fasting plasma glucose | Number | 2013 | 2988.292885 | 4530.644319 | 1508.697235 |
| Deaths | Global | Both | 65-69 years | Colon and rectum cancer | High fasting plasma glucose | Number | 2013 | 7982.611921 | 11973.39791 | 4066.055602 |
| Deaths | Global | Male | 65-69 years | Colon and rectum cancer | High fasting plasma glucose | Rate | 2013 | 5.19628885 | 7.793527148 | 2.643470191 |
| Deaths | Global | Female | 65-69 years | Colon and rectum cancer | High fasting plasma glucose | Rate | 2013 | 2.901301 | 4.398753201 | 1.464777706 |
| Deaths | Global | Both | 65-69 years | Colon and rectum cancer | High fasting plasma glucose | Rate | 2013 | 4.009115127 | 6.013411547 | 2.04209915 |
| Deaths | Global | Male | 65-69 years | Colon and rectum cancer | High fasting plasma glucose | Number | 2014 | 5276.271427 | 7904.823213 | 2656.359415 |
| Deaths | Global | Female | 65-69 years | Colon and rectum cancer | High fasting plasma glucose | Number | 2014 | 3150.120544 | 4771.309755 | 1585.300775 |
| Deaths | Global | Both | 65-69 years | Colon and rectum cancer | High fasting plasma glucose | Number | 2014 | 8426.391972 | 12698.47996 | 4248.014206 |
| Deaths | Global | Male | 65-69 years | Colon and rectum cancer | High fasting plasma glucose | Rate | 2014 | 5.176995107 | 7.75608906 | 2.606378364 |
| Deaths | Global | Female | 65-69 years | Colon and rectum cancer | High fasting plasma glucose | Rate | 2014 | 2.884371898 | 4.368795283 | 1.451562549 |
| Deaths | Global | Both | 65-69 years | Colon and rectum cancer | High fasting plasma glucose | Rate | 2014 | 3.99107205 | 6.014501655 | 2.012027309 |
| Deaths | Global | Male | 65-69 years | Colon and rectum cancer | High fasting plasma glucose | Number | 2015 | 5607.081039 | 8410.485612 | 2835.222567 |
| Deaths | Global | Female | 65-69 years | Colon and rectum cancer | High fasting plasma glucose | Number | 2015 | 3353.013707 | 5069.881385 | 1677.354632 |
| Deaths | Global | Both | 65-69 years | Colon and rectum cancer | High fasting plasma glucose | Number | 2015 | 8960.094747 | 13436.72339 | 4482.410222 |
| Deaths | Global | Male | 65-69 years | Colon and rectum cancer | High fasting plasma glucose | Rate | 2015 | 5.266885018 | 7.900199828 | 2.663202325 |
| Deaths | Global | Female | 65-69 years | Colon and rectum cancer | High fasting plasma glucose | Rate | 2015 | 2.928066461 | 4.42734535 | 1.464773565 |
| Deaths | Global | Both | 65-69 years | Colon and rectum cancer | High fasting plasma glucose | Rate | 2015 | 4.054854398 | 6.080734465 | 2.028496496 |
| Deaths | Global | Male | 65-69 years | Colon and rectum cancer | High fasting plasma glucose | Number | 2016 | 5984.786334 | 8995.366492 | 3034.702123 |
| Deaths | Global | Female | 65-69 years | Colon and rectum cancer | High fasting plasma glucose | Number | 2016 | 3587.394411 | 5346.360342 | 1798.491748 |
| Deaths | Global | Both | 65-69 years | Colon and rectum cancer | High fasting plasma glucose | Number | 2016 | 9572.180745 | 14370.78325 | 4901.462859 |
| Deaths | Global | Male | 65-69 years | Colon and rectum cancer | High fasting plasma glucose | Rate | 2016 | 5.341688515 | 8.028765472 | 2.708606886 |
| Deaths | Global | Female | 65-69 years | Colon and rectum cancer | High fasting plasma glucose | Rate | 2016 | 2.965336015 | 4.419295192 | 1.48663117 |
| Deaths | Global | Both | 65-69 years | Colon and rectum cancer | High fasting plasma glucose | Rate | 2016 | 4.107934241 | 6.167270987 | 2.103479619 |
| Deaths | Global | Male | 65-69 years | Colon and rectum cancer | High fasting plasma glucose | Number | 2017 | 6191.805096 | 9308.74616 | 3134.091057 |
| Deaths | Global | Female | 65-69 years | Colon and rectum cancer | High fasting plasma glucose | Number | 2017 | 3753.884565 | 5697.697125 | 1892.559872 |
| Deaths | Global | Both | 65-69 years | Colon and rectum cancer | High fasting plasma glucose | Number | 2017 | 9945.689662 | 15066.93864 | 4997.098025 |
| Deaths | Global | Male | 65-69 years | Colon and rectum cancer | High fasting plasma glucose | Rate | 2017 | 5.335247852 | 8.020999883 | 2.700529542 |
| Deaths | Global | Female | 65-69 years | Colon and rectum cancer | High fasting plasma glucose | Rate | 2017 | 2.987035257 | 4.533762799 | 1.505944832 |
| Deaths | Global | Both | 65-69 years | Colon and rectum cancer | High fasting plasma glucose | Rate | 2017 | 4.114425922 | 6.233032099 | 2.067246249 |
| Deaths | Global | Male | 65-69 years | Colon and rectum cancer | High fasting plasma glucose | Number | 2018 | 6452.393005 | 9705.011945 | 3272.711168 |
| Deaths | Global | Female | 65-69 years | Colon and rectum cancer | High fasting plasma glucose | Number | 2018 | 3928.331935 | 5930.780501 | 1950.863995 |
| Deaths | Global | Both | 65-69 years | Colon and rectum cancer | High fasting plasma glucose | Number | 2018 | 10380.72494 | 15667.2262 | 5264.143214 |
| Deaths | Global | Male | 65-69 years | Colon and rectum cancer | High fasting plasma glucose | Rate | 2018 | 5.358364211 | 8.059488724 | 2.717810025 |
| Deaths | Global | Female | 65-69 years | Colon and rectum cancer | High fasting plasma glucose | Rate | 2018 | 3.002721225 | 4.533344123 | 1.491192908 |
| Deaths | Global | Both | 65-69 years | Colon and rectum cancer | High fasting plasma glucose | Rate | 2018 | 4.131747833 | 6.235887018 | 2.095240212 |
| Deaths | Global | Male | 65-69 years | Colon and rectum cancer | High fasting plasma glucose | Number | 2019 | 6633.132205 | 10190.82577 | 3384.904993 |
| Deaths | Global | Female | 65-69 years | Colon and rectum cancer | High fasting plasma glucose | Number | 2019 | 4063.403806 | 6162.769221 | 2039.604587 |
| Deaths | Global | Both | 65-69 years | Colon and rectum cancer | High fasting plasma glucose | Number | 2019 | 10696.53601 | 16216.64247 | 5503.590576 |
| Deaths | Global | Male | 65-69 years | Colon and rectum cancer | High fasting plasma glucose | Rate | 2019 | 5.350576941 | 8.220369459 | 2.730413633 |
| Deaths | Global | Female | 65-69 years | Colon and rectum cancer | High fasting plasma glucose | Rate | 2019 | 3.008407753 | 4.562707422 | 1.510054759 |
| Deaths | Global | Both | 65-69 years | Colon and rectum cancer | High fasting plasma glucose | Rate | 2019 | 4.129320251 | 6.260317368 | 2.124621279 |
| Deaths | Global | Male | 65-69 years | Colon and rectum cancer | High fasting plasma glucose | Number | 2020 | 6810.148126 | 10338.0284 | 3431.865397 |
| Deaths | Global | Female | 65-69 years | Colon and rectum cancer | High fasting plasma glucose | Number | 2020 | 4203.231055 | 6252.19042 | 2121.011522 |
| Deaths | Global | Both | 65-69 years | Colon and rectum cancer | High fasting plasma glucose | Number | 2020 | 11013.37918 | 16752.6001 | 5560.372016 |
| Deaths | Global | Male | 65-69 years | Colon and rectum cancer | High fasting plasma glucose | Rate | 2020 | 5.282482546 | 8.018981901 | 2.662022723 |
| Deaths | Global | Female | 65-69 years | Colon and rectum cancer | High fasting plasma glucose | Rate | 2020 | 2.98869037 | 4.445594605 | 1.508136628 |
| Deaths | Global | Both | 65-69 years | Colon and rectum cancer | High fasting plasma glucose | Rate | 2020 | 4.085727616 | 6.214855563 | 2.062778837 |
| Deaths | Global | Male | 65-69 years | Colon and rectum cancer | High fasting plasma glucose | Number | 2021 | 6863.368373 | 10485.0153 | 3471.019378 |
| Deaths | Global | Female | 65-69 years | Colon and rectum cancer | High fasting plasma glucose | Number | 2021 | 4292.623326 | 6512.411962 | 2187.508318 |
| Deaths | Global | Both | 65-69 years | Colon and rectum cancer | High fasting plasma glucose | Number | 2021 | 11155.9917 | 16841.77761 | 5663.000428 |
| Deaths | Global | Male | 65-69 years | Colon and rectum cancer | High fasting plasma glucose | Rate | 2021 | 5.206098669 | 7.953241213 | 2.632886417 |
| Deaths | Global | Female | 65-69 years | Colon and rectum cancer | High fasting plasma glucose | Rate | 2021 | 2.980803736 | 4.522228118 | 1.519008884 |
| Deaths | Global | Both | 65-69 years | Colon and rectum cancer | High fasting plasma glucose | Rate | 2021 | 4.044338892 | 6.105585056 | 2.05298583 |
| Deaths | Global | Male | 70-74 years | Colon and rectum cancer | High fasting plasma glucose | Number | 1990 | 2647.819959 | 3980.130281 | 1319.294355 |
| Deaths | Global | Female | 70-74 years | Colon and rectum cancer | High fasting plasma glucose | Number | 1990 | 2235.06578 | 3406.818845 | 1124.669879 |
| Deaths | Global | Both | 70-74 years | Colon and rectum cancer | High fasting plasma glucose | Number | 1990 | 4882.88574 | 7372.369373 | 2417.712892 |
| Deaths | Global | Male | 70-74 years | Colon and rectum cancer | High fasting plasma glucose | Rate | 1990 | 7.038608859 | 10.58024363 | 3.507034874 |
| Deaths | Global | Female | 70-74 years | Colon and rectum cancer | High fasting plasma glucose | Rate | 1990 | 4.751148506 | 7.241980263 | 2.390745571 |
| Deaths | Global | Both | 70-74 years | Colon and rectum cancer | High fasting plasma glucose | Rate | 1990 | 5.767563373 | 8.708089813 | 2.855752329 |
| Deaths | Global | Male | 70-74 years | Colon and rectum cancer | High fasting plasma glucose | Number | 1991 | 2774.494258 | 4224.243964 | 1351.837974 |
| Deaths | Global | Female | 70-74 years | Colon and rectum cancer | High fasting plasma glucose | Number | 1991 | 2322.649844 | 3488.400681 | 1155.753609 |
| Deaths | Global | Both | 70-74 years | Colon and rectum cancer | High fasting plasma glucose | Number | 1991 | 5097.144102 | 7701.54491 | 2517.51827 |
| Deaths | Global | Male | 70-74 years | Colon and rectum cancer | High fasting plasma glucose | Rate | 1991 | 7.110342798 | 10.82569285 | 3.464426489 |
| Deaths | Global | Female | 70-74 years | Colon and rectum cancer | High fasting plasma glucose | Rate | 1991 | 4.768442772 | 7.161750643 | 2.372783378 |
| Deaths | Global | Both | 70-74 years | Colon and rectum cancer | High fasting plasma glucose | Rate | 1991 | 5.810081023 | 8.778759053 | 2.869643242 |
| Deaths | Global | Male | 70-74 years | Colon and rectum cancer | High fasting plasma glucose | Number | 1992 | 2963.136907 | 4493.635196 | 1486.082404 |
| Deaths | Global | Female | 70-74 years | Colon and rectum cancer | High fasting plasma glucose | Number | 1992 | 2455.491666 | 3724.014855 | 1210.851812 |
| Deaths | Global | Both | 70-74 years | Colon and rectum cancer | High fasting plasma glucose | Number | 1992 | 5418.628573 | 8188.504161 | 2701.770028 |
| Deaths | Global | Male | 70-74 years | Colon and rectum cancer | High fasting plasma glucose | Rate | 1992 | 7.28818788 | 11.05263057 | 3.655196537 |
| Deaths | Global | Female | 70-74 years | Colon and rectum cancer | High fasting plasma glucose | Rate | 1992 | 4.838582713 | 7.338226453 | 2.386001437 |
| Deaths | Global | Both | 70-74 years | Colon and rectum cancer | High fasting plasma glucose | Rate | 1992 | 5.928162275 | 8.958499517 | 2.955827464 |
| Deaths | Global | Male | 70-74 years | Colon and rectum cancer | High fasting plasma glucose | Number | 1993 | 3180.047617 | 4814.62857 | 1575.786446 |
| Deaths | Global | Female | 70-74 years | Colon and rectum cancer | High fasting plasma glucose | Number | 1993 | 2628.898558 | 3956.080544 | 1315.395715 |
| Deaths | Global | Both | 70-74 years | Colon and rectum cancer | High fasting plasma glucose | Number | 1993 | 5808.946175 | 8730.573759 | 2914.174107 |
| Deaths | Global | Male | 70-74 years | Colon and rectum cancer | High fasting plasma glucose | Rate | 1993 | 7.505456164 | 11.36334673 | 3.719125472 |
| Deaths | Global | Female | 70-74 years | Colon and rectum cancer | High fasting plasma glucose | Rate | 1993 | 4.958295931 | 7.461458719 | 2.480933014 |
| Deaths | Global | Both | 70-74 years | Colon and rectum cancer | High fasting plasma glucose | Rate | 1993 | 6.089679541 | 9.152502847 | 3.055009618 |
| Deaths | Global | Male | 70-74 years | Colon and rectum cancer | High fasting plasma glucose | Number | 1994 | 3382.743208 | 5139.558123 | 1688.242947 |
| Deaths | Global | Female | 70-74 years | Colon and rectum cancer | High fasting plasma glucose | Number | 1994 | 2784.456294 | 4114.827632 | 1394.246968 |
| Deaths | Global | Both | 70-74 years | Colon and rectum cancer | High fasting plasma glucose | Number | 1994 | 6167.199502 | 9261.387474 | 3082.537427 |
| Deaths | Global | Male | 70-74 years | Colon and rectum cancer | High fasting plasma glucose | Rate | 1994 | 7.668551324 | 11.6511845 | 3.827183113 |
| Deaths | Global | Female | 70-74 years | Colon and rectum cancer | High fasting plasma glucose | Rate | 1994 | 5.053851903 | 7.468506331 | 2.530590158 |
| Deaths | Global | Both | 70-74 years | Colon and rectum cancer | High fasting plasma glucose | Rate | 1994 | 6.216457614 | 9.335359212 | 3.107157999 |
| Deaths | Global | Male | 70-74 years | Colon and rectum cancer | High fasting plasma glucose | Number | 1995 | 3553.494902 | 5375.418669 | 1771.462468 |
| Deaths | Global | Female | 70-74 years | Colon and rectum cancer | High fasting plasma glucose | Number | 1995 | 2883.745355 | 4330.28616 | 1453.618192 |
| Deaths | Global | Both | 70-74 years | Colon and rectum cancer | High fasting plasma glucose | Number | 1995 | 6437.240257 | 9700.27707 | 3229.240103 |
| Deaths | Global | Male | 70-74 years | Colon and rectum cancer | High fasting plasma glucose | Rate | 1995 | 7.764976525 | 11.74618254 | 3.870939696 |
| Deaths | Global | Female | 70-74 years | Colon and rectum cancer | High fasting plasma glucose | Rate | 1995 | 5.065386192 | 7.606278995 | 2.553324449 |
| Deaths | Global | Both | 70-74 years | Colon and rectum cancer | High fasting plasma glucose | Rate | 1995 | 6.268399263 | 9.445850584 | 3.144541057 |
| Deaths | Global | Male | 70-74 years | Colon and rectum cancer | High fasting plasma glucose | Number | 1996 | 3704.320163 | 5622.466535 | 1832.944865 |
| Deaths | Global | Female | 70-74 years | Colon and rectum cancer | High fasting plasma glucose | Number | 1996 | 2957.113249 | 4483.33996 | 1489.985362 |
| Deaths | Global | Both | 70-74 years | Colon and rectum cancer | High fasting plasma glucose | Number | 1996 | 6661.433413 | 10084.25784 | 3323.841564 |
| Deaths | Global | Male | 70-74 years | Colon and rectum cancer | High fasting plasma glucose | Rate | 1996 | 7.813810152 | 11.85990523 | 3.866372928 |
| Deaths | Global | Female | 70-74 years | Colon and rectum cancer | High fasting plasma glucose | Rate | 1996 | 5.049464065 | 7.655595882 | 2.544247348 |
| Deaths | Global | Both | 70-74 years | Colon and rectum cancer | High fasting plasma glucose | Rate | 1996 | 6.286134764 | 9.516120603 | 3.136579578 |
| Deaths | Global | Male | 70-74 years | Colon and rectum cancer | High fasting plasma glucose | Number | 1997 | 3892.878617 | 5849.096095 | 1899.763761 |
| Deaths | Global | Female | 70-74 years | Colon and rectum cancer | High fasting plasma glucose | Number | 1997 | 3057.780724 | 4639.898745 | 1531.068658 |
| Deaths | Global | Both | 70-74 years | Colon and rectum cancer | High fasting plasma glucose | Number | 1997 | 6950.659342 | 10474.12446 | 3435.903575 |
| Deaths | Global | Male | 70-74 years | Colon and rectum cancer | High fasting plasma glucose | Rate | 1997 | 7.915016916 | 11.89240639 | 3.862607541 |
| Deaths | Global | Female | 70-74 years | Colon and rectum cancer | High fasting plasma glucose | Rate | 1997 | 5.082702778 | 7.712530219 | 2.544972194 |
| Deaths | Global | Both | 70-74 years | Colon and rectum cancer | High fasting plasma glucose | Rate | 1997 | 6.356691437 | 9.579059189 | 3.142288776 |
| Deaths | Global | Male | 70-74 years | Colon and rectum cancer | High fasting plasma glucose | Number | 1998 | 4138.697379 | 6242.771508 | 2072.566305 |
| Deaths | Global | Female | 70-74 years | Colon and rectum cancer | High fasting plasma glucose | Number | 1998 | 3187.530659 | 4778.774967 | 1606.419904 |
| Deaths | Global | Both | 70-74 years | Colon and rectum cancer | High fasting plasma glucose | Number | 1998 | 7326.228037 | 11049.08623 | 3681.353146 |
| Deaths | Global | Male | 70-74 years | Colon and rectum cancer | High fasting plasma glucose | Rate | 1998 | 8.101601047 | 12.22037747 | 4.057099084 |
| Deaths | Global | Female | 70-74 years | Colon and rectum cancer | High fasting plasma glucose | Rate | 1998 | 5.167439136 | 7.747071772 | 2.604234428 |
| Deaths | Global | Both | 70-74 years | Colon and rectum cancer | High fasting plasma glucose | Rate | 1998 | 6.496619408 | 9.797907965 | 3.264483466 |
| Deaths | Global | Male | 70-74 years | Colon and rectum cancer | High fasting plasma glucose | Number | 1999 | 4375.042116 | 6596.06833 | 2159.135125 |
| Deaths | Global | Female | 70-74 years | Colon and rectum cancer | High fasting plasma glucose | Number | 1999 | 3320.045321 | 5026.410579 | 1677.411648 |
| Deaths | Global | Both | 70-74 years | Colon and rectum cancer | High fasting plasma glucose | Number | 1999 | 7695.087438 | 11622.27087 | 3816.384847 |
| Deaths | Global | Male | 70-74 years | Colon and rectum cancer | High fasting plasma glucose | Rate | 1999 | 8.263888086 | 12.45911903 | 4.07832669 |
| Deaths | Global | Female | 70-74 years | Colon and rectum cancer | High fasting plasma glucose | Rate | 1999 | 5.253903262 | 7.954191097 | 2.654469344 |
| Deaths | Global | Both | 70-74 years | Colon and rectum cancer | High fasting plasma glucose | Rate | 1999 | 6.626060795 | 10.00766709 | 3.286200218 |
| Deaths | Global | Male | 70-74 years | Colon and rectum cancer | High fasting plasma glucose | Number | 2000 | 4596.383204 | 6971.969105 | 2248.592174 |
| Deaths | Global | Female | 70-74 years | Colon and rectum cancer | High fasting plasma glucose | Number | 2000 | 3390.761543 | 5088.356469 | 1692.260981 |
| Deaths | Global | Both | 70-74 years | Colon and rectum cancer | High fasting plasma glucose | Number | 2000 | 7987.144747 | 12122.83212 | 3932.462725 |
| Deaths | Global | Male | 70-74 years | Colon and rectum cancer | High fasting plasma glucose | Rate | 2000 | 8.415709218 | 12.76526827 | 4.117040953 |
| Deaths | Global | Female | 70-74 years | Colon and rectum cancer | High fasting plasma glucose | Rate | 2000 | 5.256892616 | 7.888771656 | 2.623609518 |
| Deaths | Global | Both | 70-74 years | Colon and rectum cancer | High fasting plasma glucose | Rate | 2000 | 6.705239798 | 10.17716581 | 3.301318106 |
| Deaths | Global | Male | 70-74 years | Colon and rectum cancer | High fasting plasma glucose | Number | 2001 | 4746.378226 | 7159.976902 | 2340.312009 |
| Deaths | Global | Female | 70-74 years | Colon and rectum cancer | High fasting plasma glucose | Number | 2001 | 3451.177946 | 5252.229745 | 1759.228992 |
| Deaths | Global | Both | 70-74 years | Colon and rectum cancer | High fasting plasma glucose | Number | 2001 | 8197.556172 | 12372.19115 | 4095.797062 |
| Deaths | Global | Male | 70-74 years | Colon and rectum cancer | High fasting plasma glucose | Rate | 2001 | 8.459308888 | 12.76098392 | 4.171058697 |
| Deaths | Global | Female | 70-74 years | Colon and rectum cancer | High fasting plasma glucose | Rate | 2001 | 5.247588625 | 7.986125751 | 2.674944669 |
| Deaths | Global | Both | 70-74 years | Colon and rectum cancer | High fasting plasma glucose | Rate | 2001 | 6.726184743 | 10.15151852 | 3.360646408 |
| Deaths | Global | Male | 70-74 years | Colon and rectum cancer | High fasting plasma glucose | Number | 2002 | 4861.01927 | 7300.939725 | 2398.527508 |
| Deaths | Global | Female | 70-74 years | Colon and rectum cancer | High fasting plasma glucose | Number | 2002 | 3495.242758 | 5289.770995 | 1770.348911 |
| Deaths | Global | Both | 70-74 years | Colon and rectum cancer | High fasting plasma glucose | Number | 2002 | 8356.262028 | 12599.6214 | 4161.074483 |
| Deaths | Global | Male | 70-74 years | Colon and rectum cancer | High fasting plasma glucose | Rate | 2002 | 8.491637192 | 12.7538954 | 4.189949528 |
| Deaths | Global | Female | 70-74 years | Colon and rectum cancer | High fasting plasma glucose | Rate | 2002 | 5.235664192 | 7.92375995 | 2.651876576 |
| Deaths | Global | Both | 70-74 years | Colon and rectum cancer | High fasting plasma glucose | Rate | 2002 | 6.738751015 | 10.16072871 | 3.355620587 |
| Deaths | Global | Male | 70-74 years | Colon and rectum cancer | High fasting plasma glucose | Number | 2003 | 4954.945466 | 7393.818829 | 2464.032651 |
| Deaths | Global | Female | 70-74 years | Colon and rectum cancer | High fasting plasma glucose | Number | 2003 | 3533.294765 | 5323.383644 | 1803.888271 |
| Deaths | Global | Both | 70-74 years | Colon and rectum cancer | High fasting plasma glucose | Number | 2003 | 8488.240231 | 12772.22675 | 4272.236414 |
| Deaths | Global | Male | 70-74 years | Colon and rectum cancer | High fasting plasma glucose | Rate | 2003 | 8.51654926 | 12.70847938 | 4.235173847 |
| Deaths | Global | Female | 70-74 years | Colon and rectum cancer | High fasting plasma glucose | Rate | 2003 | 5.216772973 | 7.859769921 | 2.663371218 |
| Deaths | Global | Both | 70-74 years | Colon and rectum cancer | High fasting plasma glucose | Rate | 2003 | 6.741529452 | 10.14395687 | 3.39309525 |
| Deaths | Global | Male | 70-74 years | Colon and rectum cancer | High fasting plasma glucose | Number | 2004 | 5005.107886 | 7519.149076 | 2440.363154 |
| Deaths | Global | Female | 70-74 years | Colon and rectum cancer | High fasting plasma glucose | Number | 2004 | 3535.785713 | 5403.863504 | 1796.791662 |
| Deaths | Global | Both | 70-74 years | Colon and rectum cancer | High fasting plasma glucose | Number | 2004 | 8540.893598 | 12955.49596 | 4257.734777 |
| Deaths | Global | Male | 70-74 years | Colon and rectum cancer | High fasting plasma glucose | Rate | 2004 | 8.466457286 | 12.71911734 | 4.128028982 |
| Deaths | Global | Female | 70-74 years | Colon and rectum cancer | High fasting plasma glucose | Rate | 2004 | 5.14885139 | 7.869167527 | 2.616508464 |
| Deaths | Global | Both | 70-74 years | Colon and rectum cancer | High fasting plasma glucose | Rate | 2004 | 6.683629385 | 10.13825222 | 3.33186697 |
| Deaths | Global | Male | 70-74 years | Colon and rectum cancer | High fasting plasma glucose | Number | 2005 | 5075.013973 | 7608.115563 | 2506.595208 |
| Deaths | Global | Female | 70-74 years | Colon and rectum cancer | High fasting plasma glucose | Number | 2005 | 3567.02103 | 5401.096268 | 1817.871269 |
| Deaths | Global | Both | 70-74 years | Colon and rectum cancer | High fasting plasma glucose | Number | 2005 | 8642.035003 | 13037.65124 | 4294.393437 |
| Deaths | Global | Male | 70-74 years | Colon and rectum cancer | High fasting plasma glucose | Rate | 2005 | 8.435583941 | 12.64605335 | 4.166411047 |
| Deaths | Global | Female | 70-74 years | Colon and rectum cancer | High fasting plasma glucose | Rate | 2005 | 5.106312195 | 7.731853417 | 2.602344688 |
| Deaths | Global | Both | 70-74 years | Colon and rectum cancer | High fasting plasma glucose | Rate | 2005 | 6.646844611 | 10.02764301 | 3.30294495 |
| Deaths | Global | Male | 70-74 years | Colon and rectum cancer | High fasting plasma glucose | Number | 2006 | 5085.612172 | 7706.563639 | 2537.116897 |
| Deaths | Global | Female | 70-74 years | Colon and rectum cancer | High fasting plasma glucose | Number | 2006 | 3566.330151 | 5313.15724 | 1799.786142 |
| Deaths | Global | Both | 70-74 years | Colon and rectum cancer | High fasting plasma glucose | Number | 2006 | 8651.942322 | 12985.18007 | 4372.493575 |
| Deaths | Global | Male | 70-74 years | Colon and rectum cancer | High fasting plasma glucose | Rate | 2006 | 8.271874468 | 12.5349171 | 4.126683626 |
| Deaths | Global | Female | 70-74 years | Colon and rectum cancer | High fasting plasma glucose | Rate | 2006 | 4.996608279 | 7.444001068 | 2.521591092 |
| Deaths | Global | Both | 70-74 years | Colon and rectum cancer | High fasting plasma glucose | Rate | 2006 | 6.512280939 | 9.773890935 | 3.291157696 |
| Deaths | Global | Male | 70-74 years | Colon and rectum cancer | High fasting plasma glucose | Number | 2007 | 5190.520006 | 7868.559976 | 2553.210258 |
| Deaths | Global | Female | 70-74 years | Colon and rectum cancer | High fasting plasma glucose | Number | 2007 | 3623.752817 | 5429.566496 | 1847.196949 |
| Deaths | Global | Both | 70-74 years | Colon and rectum cancer | High fasting plasma glucose | Number | 2007 | 8814.272823 | 13254.34006 | 4428.464827 |
| Deaths | Global | Male | 70-74 years | Colon and rectum cancer | High fasting plasma glucose | Rate | 2007 | 8.235754793 | 12.48497847 | 4.051157417 |
| Deaths | Global | Female | 70-74 years | Colon and rectum cancer | High fasting plasma glucose | Rate | 2007 | 4.947619687 | 7.413151902 | 2.522034049 |
| Deaths | Global | Both | 70-74 years | Colon and rectum cancer | High fasting plasma glucose | Rate | 2007 | 6.468404639 | 9.726773434 | 3.2498543 |
| Deaths | Global | Male | 70-74 years | Colon and rectum cancer | High fasting plasma glucose | Number | 2008 | 5353.3154 | 8069.678649 | 2645.809126 |
| Deaths | Global | Female | 70-74 years | Colon and rectum cancer | High fasting plasma glucose | Number | 2008 | 3698.624079 | 5551.741974 | 1884.262962 |
| Deaths | Global | Both | 70-74 years | Colon and rectum cancer | High fasting plasma glucose | Number | 2008 | 9051.939479 | 13657.13642 | 4572.923176 |
| Deaths | Global | Male | 70-74 years | Colon and rectum cancer | High fasting plasma glucose | Rate | 2008 | 8.293497348 | 12.5017589 | 4.09895725 |
| Deaths | Global | Female | 70-74 years | Colon and rectum cancer | High fasting plasma glucose | Rate | 2008 | 4.926506084 | 7.394828464 | 2.509807093 |
| Deaths | Global | Both | 70-74 years | Colon and rectum cancer | High fasting plasma glucose | Rate | 2008 | 6.483066329 | 9.781342605 | 3.275161564 |
| Deaths | Global | Male | 70-74 years | Colon and rectum cancer | High fasting plasma glucose | Number | 2009 | 5459.81394 | 8239.079706 | 2739.63216 |
| Deaths | Global | Female | 70-74 years | Colon and rectum cancer | High fasting plasma glucose | Number | 2009 | 3747.502184 | 5606.85331 | 1886.899882 |
| Deaths | Global | Both | 70-74 years | Colon and rectum cancer | High fasting plasma glucose | Number | 2009 | 9207.316124 | 13876.39864 | 4701.817661 |
| Deaths | Global | Male | 70-74 years | Colon and rectum cancer | High fasting plasma glucose | Rate | 2009 | 8.275032313 | 12.48735791 | 4.1522559 |
| Deaths | Global | Female | 70-74 years | Colon and rectum cancer | High fasting plasma glucose | Rate | 2009 | 4.882784975 | 7.305415116 | 2.458524622 |
| Deaths | Global | Both | 70-74 years | Colon and rectum cancer | High fasting plasma glucose | Rate | 2009 | 6.450923824 | 9.722224083 | 3.294235493 |
| Deaths | Global | Male | 70-74 years | Colon and rectum cancer | High fasting plasma glucose | Number | 2010 | 5602.700493 | 8465.875053 | 2752.673344 |
| Deaths | Global | Female | 70-74 years | Colon and rectum cancer | High fasting plasma glucose | Number | 2010 | 3806.007388 | 5701.134303 | 1938.048875 |
| Deaths | Global | Both | 70-74 years | Colon and rectum cancer | High fasting plasma glucose | Number | 2010 | 9408.707882 | 14221.79527 | 4708.594881 |
| Deaths | Global | Male | 70-74 years | Colon and rectum cancer | High fasting plasma glucose | Rate | 2010 | 8.316893398 | 12.56711481 | 4.086188578 |
| Deaths | Global | Female | 70-74 years | Colon and rectum cancer | High fasting plasma glucose | Rate | 2010 | 4.856980296 | 7.275418609 | 2.47321254 |
| Deaths | Global | Both | 70-74 years | Colon and rectum cancer | High fasting plasma glucose | Rate | 2010 | 6.456397382 | 9.759210608 | 3.231108888 |
| Deaths | Global | Male | 70-74 years | Colon and rectum cancer | High fasting plasma glucose | Number | 2011 | 5703.945061 | 8626.463852 | 2799.196447 |
| Deaths | Global | Female | 70-74 years | Colon and rectum cancer | High fasting plasma glucose | Number | 2011 | 3840.436879 | 5768.292514 | 1954.296941 |
| Deaths | Global | Both | 70-74 years | Colon and rectum cancer | High fasting plasma glucose | Number | 2011 | 9544.381939 | 14426.42106 | 4825.560023 |
| Deaths | Global | Male | 70-74 years | Colon and rectum cancer | High fasting plasma glucose | Rate | 2011 | 8.307346265 | 12.56376446 | 4.076808928 |
| Deaths | Global | Female | 70-74 years | Colon and rectum cancer | High fasting plasma glucose | Rate | 2011 | 4.82229792 | 7.243036631 | 2.453940104 |
| Deaths | Global | Both | 70-74 years | Colon and rectum cancer | High fasting plasma glucose | Rate | 2011 | 6.435834845 | 9.727823547 | 3.2539045 |
| Deaths | Global | Male | 70-74 years | Colon and rectum cancer | High fasting plasma glucose | Number | 2012 | 5779.000975 | 8746.110006 | 2840.200915 |
| Deaths | Global | Female | 70-74 years | Colon and rectum cancer | High fasting plasma glucose | Number | 2012 | 3863.31183 | 5795.988161 | 1958.186468 |
| Deaths | Global | Both | 70-74 years | Colon and rectum cancer | High fasting plasma glucose | Number | 2012 | 9642.312805 | 14585.79398 | 4814.723769 |
| Deaths | Global | Male | 70-74 years | Colon and rectum cancer | High fasting plasma glucose | Rate | 2012 | 8.280697273 | 12.53225075 | 4.069707563 |
| Deaths | Global | Female | 70-74 years | Colon and rectum cancer | High fasting plasma glucose | Rate | 2012 | 4.795872528 | 7.195075525 | 2.430870999 |
| Deaths | Global | Both | 70-74 years | Colon and rectum cancer | High fasting plasma glucose | Rate | 2012 | 6.413510812 | 9.70162961 | 3.202476789 |
| Deaths | Global | Male | 70-74 years | Colon and rectum cancer | High fasting plasma glucose | Number | 2013 | 5866.71555 | 8852.195903 | 2957.234555 |
| Deaths | Global | Female | 70-74 years | Colon and rectum cancer | High fasting plasma glucose | Number | 2013 | 3875.318762 | 5869.126563 | 1950.696666 |
| Deaths | Global | Both | 70-74 years | Colon and rectum cancer | High fasting plasma glucose | Number | 2013 | 9742.034312 | 14642.86884 | 4945.312771 |
| Deaths | Global | Male | 70-74 years | Colon and rectum cancer | High fasting plasma glucose | Rate | 2013 | 8.257064624 | 12.45895647 | 4.162137506 |
| Deaths | Global | Female | 70-74 years | Colon and rectum cancer | High fasting plasma glucose | Rate | 2013 | 4.765649399 | 7.217522272 | 2.39885722 |
| Deaths | Global | Both | 70-74 years | Colon and rectum cancer | High fasting plasma glucose | Rate | 2013 | 6.393727916 | 9.610161108 | 3.245624405 |
| Deaths | Global | Male | 70-74 years | Colon and rectum cancer | High fasting plasma glucose | Number | 2014 | 5949.291551 | 8947.117044 | 2951.687576 |
| Deaths | Global | Female | 70-74 years | Colon and rectum cancer | High fasting plasma glucose | Number | 2014 | 3884.713554 | 5888.074366 | 1940.267105 |
| Deaths | Global | Both | 70-74 years | Colon and rectum cancer | High fasting plasma glucose | Number | 2014 | 9834.005105 | 14815.60932 | 4897.982544 |
| Deaths | Global | Male | 70-74 years | Colon and rectum cancer | High fasting plasma glucose | Rate | 2014 | 8.220360783 | 12.36256946 | 4.07845818 |
| Deaths | Global | Female | 70-74 years | Colon and rectum cancer | High fasting plasma glucose | Rate | 2014 | 4.720537097 | 7.15493513 | 2.357729269 |
| Deaths | Global | Both | 70-74 years | Colon and rectum cancer | High fasting plasma glucose | Rate | 2014 | 6.358198902 | 9.579066706 | 3.16680202 |
| Deaths | Global | Male | 70-74 years | Colon and rectum cancer | High fasting plasma glucose | Number | 2015 | 6048.192618 | 9195.889303 | 3017.919081 |
| Deaths | Global | Female | 70-74 years | Colon and rectum cancer | High fasting plasma glucose | Number | 2015 | 3917.921305 | 5832.453802 | 1975.497523 |
| Deaths | Global | Both | 70-74 years | Colon and rectum cancer | High fasting plasma glucose | Number | 2015 | 9966.113923 | 14921.48962 | 4993.416604 |
| Deaths | Global | Male | 70-74 years | Colon and rectum cancer | High fasting plasma glucose | Rate | 2015 | 8.208559788 | 12.48058915 | 4.095896208 |
| Deaths | Global | Female | 70-74 years | Colon and rectum cancer | High fasting plasma glucose | Rate | 2015 | 4.710161377 | 7.011830124 | 2.374961467 |
| Deaths | Global | Both | 70-74 years | Colon and rectum cancer | High fasting plasma glucose | Rate | 2015 | 6.353438954 | 9.51251151 | 3.183323792 |
| Deaths | Global | Male | 70-74 years | Colon and rectum cancer | High fasting plasma glucose | Number | 2016 | 6184.59491 | 9443.238107 | 3158.608453 |
| Deaths | Global | Female | 70-74 years | Colon and rectum cancer | High fasting plasma glucose | Number | 2016 | 4002.240987 | 5996.651462 | 2041.384378 |
| Deaths | Global | Both | 70-74 years | Colon and rectum cancer | High fasting plasma glucose | Number | 2016 | 10186.8359 | 15348.68253 | 5151.854067 |
| Deaths | Global | Male | 70-74 years | Colon and rectum cancer | High fasting plasma glucose | Rate | 2016 | 8.176943864 | 12.48534933 | 4.176144822 |
| Deaths | Global | Female | 70-74 years | Colon and rectum cancer | High fasting plasma glucose | Rate | 2016 | 4.708820384 | 7.055335931 | 2.401782501 |
| Deaths | Global | Both | 70-74 years | Colon and rectum cancer | High fasting plasma glucose | Rate | 2016 | 6.341836777 | 9.555355592 | 3.207297921 |
| Deaths | Global | Male | 70-74 years | Colon and rectum cancer | High fasting plasma glucose | Number | 2017 | 6483.548282 | 9857.127902 | 3251.827778 |
| Deaths | Global | Female | 70-74 years | Colon and rectum cancer | High fasting plasma glucose | Number | 2017 | 4180.345768 | 6304.856667 | 2131.943194 |
| Deaths | Global | Both | 70-74 years | Colon and rectum cancer | High fasting plasma glucose | Number | 2017 | 10663.89405 | 16201.99363 | 5405.002692 |
| Deaths | Global | Male | 70-74 years | Colon and rectum cancer | High fasting plasma glucose | Rate | 2017 | 8.162329488 | 12.40942802 | 4.093821562 |
| Deaths | Global | Female | 70-74 years | Colon and rectum cancer | High fasting plasma glucose | Rate | 2017 | 4.693154342 | 7.078281817 | 2.3934715 |
| Deaths | Global | Both | 70-74 years | Colon and rectum cancer | High fasting plasma glucose | Rate | 2017 | 6.328501292 | 9.615093427 | 3.207605623 |
| Deaths | Global | Male | 70-74 years | Colon and rectum cancer | High fasting plasma glucose | Number | 2018 | 6817.000804 | 10276.27361 | 3304.706427 |
| Deaths | Global | Female | 70-74 years | Colon and rectum cancer | High fasting plasma glucose | Number | 2018 | 4374.098053 | 6583.502424 | 2171.973893 |
| Deaths | Global | Both | 70-74 years | Colon and rectum cancer | High fasting plasma glucose | Number | 2018 | 11191.09886 | 16938.13725 | 5512.135328 |
| Deaths | Global | Male | 70-74 years | Colon and rectum cancer | High fasting plasma glucose | Rate | 2018 | 8.187678527 | 12.34249888 | 3.969175687 |
| Deaths | Global | Female | 70-74 years | Colon and rectum cancer | High fasting plasma glucose | Rate | 2018 | 4.683353789 | 7.048966587 | 2.325535925 |
| Deaths | Global | Both | 70-74 years | Colon and rectum cancer | High fasting plasma glucose | Rate | 2018 | 6.334967946 | 9.588205581 | 3.120265584 |
| Deaths | Global | Male | 70-74 years | Colon and rectum cancer | High fasting plasma glucose | Number | 2019 | 7219.869663 | 11019.47984 | 3572.064198 |
| Deaths | Global | Female | 70-74 years | Colon and rectum cancer | High fasting plasma glucose | Number | 2019 | 4657.341983 | 7106.82739 | 2382.202184 |
| Deaths | Global | Both | 70-74 years | Colon and rectum cancer | High fasting plasma glucose | Number | 2019 | 11877.21165 | 18097.87036 | 5935.859383 |
| Deaths | Global | Male | 70-74 years | Colon and rectum cancer | High fasting plasma glucose | Rate | 2019 | 8.167037779 | 12.46511535 | 4.040680042 |
| Deaths | Global | Female | 70-74 years | Colon and rectum cancer | High fasting plasma glucose | Rate | 2019 | 4.696464957 | 7.166526725 | 2.402213348 |
| Deaths | Global | Both | 70-74 years | Colon and rectum cancer | High fasting plasma glucose | Rate | 2019 | 6.332165086 | 9.648620081 | 3.164618318 |
| Deaths | Global | Male | 70-74 years | Colon and rectum cancer | High fasting plasma glucose | Number | 2020 | 7559.70862 | 11654.91502 | 3692.838219 |
| Deaths | Global | Female | 70-74 years | Colon and rectum cancer | High fasting plasma glucose | Number | 2020 | 4905.340361 | 7362.363971 | 2454.540403 |
| Deaths | Global | Both | 70-74 years | Colon and rectum cancer | High fasting plasma glucose | Number | 2020 | 12465.04898 | 19055.00515 | 6137.396078 |
| Deaths | Global | Male | 70-74 years | Colon and rectum cancer | High fasting plasma glucose | Rate | 2020 | 8.204716689 | 12.64933353 | 4.007917882 |
| Deaths | Global | Female | 70-74 years | Colon and rectum cancer | High fasting plasma glucose | Rate | 2020 | 4.720865306 | 7.085487669 | 2.362232542 |
| Deaths | Global | Both | 70-74 years | Colon and rectum cancer | High fasting plasma glucose | Rate | 2020 | 6.358219388 | 9.719649186 | 3.130586233 |
| Deaths | Global | Male | 70-74 years | Colon and rectum cancer | High fasting plasma glucose | Number | 2021 | 7908.024224 | 12254.13042 | 3891.460805 |
| Deaths | Global | Female | 70-74 years | Colon and rectum cancer | High fasting plasma glucose | Number | 2021 | 5199.588032 | 7959.417622 | 2684.916337 |
| Deaths | Global | Both | 70-74 years | Colon and rectum cancer | High fasting plasma glucose | Number | 2021 | 13107.61226 | 20243.37823 | 6554.086305 |
| Deaths | Global | Male | 70-74 years | Colon and rectum cancer | High fasting plasma glucose | Rate | 2021 | 8.204075483 | 12.71288606 | 4.037144712 |
| Deaths | Global | Female | 70-74 years | Colon and rectum cancer | High fasting plasma glucose | Rate | 2021 | 4.75074661 | 7.272340819 | 2.453147654 |
| Deaths | Global | Both | 70-74 years | Colon and rectum cancer | High fasting plasma glucose | Rate | 2021 | 6.367888604 | 9.834558349 | 3.184080416 |
| Deaths | Global | Male | 75-79 years | Colon and rectum cancer | High fasting plasma glucose | Number | 1990 | 2780.311701 | 4193.122255 | 1362.848067 |
| Deaths | Global | Female | 75-79 years | Colon and rectum cancer | High fasting plasma glucose | Number | 1990 | 2804.772861 | 4168.698779 | 1382.259646 |
| Deaths | Global | Both | 75-79 years | Colon and rectum cancer | High fasting plasma glucose | Number | 1990 | 5585.084562 | 8325.781601 | 2761.320694 |
| Deaths | Global | Male | 75-79 years | Colon and rectum cancer | High fasting plasma glucose | Rate | 1990 | 11.01862763 | 16.61772409 | 5.401090593 |
| Deaths | Global | Female | 75-79 years | Colon and rectum cancer | High fasting plasma glucose | Rate | 1990 | 7.721821768 | 11.47684699 | 3.805499823 |
| Deaths | Global | Both | 75-79 years | Colon and rectum cancer | High fasting plasma glucose | Rate | 1990 | 9.073248142 | 13.52564703 | 4.485903047 |
| Deaths | Global | Male | 75-79 years | Colon and rectum cancer | High fasting plasma glucose | Number | 1991 | 2802.236095 | 4236.543765 | 1388.271085 |
| Deaths | Global | Female | 75-79 years | Colon and rectum cancer | High fasting plasma glucose | Number | 1991 | 2815.943583 | 4185.796781 | 1399.10946 |
| Deaths | Global | Both | 75-79 years | Colon and rectum cancer | High fasting plasma glucose | Number | 1991 | 5618.179678 | 8416.426843 | 2781.033094 |
| Deaths | Global | Male | 75-79 years | Colon and rectum cancer | High fasting plasma glucose | Rate | 1991 | 11.01684249 | 16.65574697 | 5.45791409 |
| Deaths | Global | Female | 75-79 years | Colon and rectum cancer | High fasting plasma glucose | Rate | 1991 | 7.749848622 | 11.51986553 | 3.850534004 |
| Deaths | Global | Both | 75-79 years | Colon and rectum cancer | High fasting plasma glucose | Rate | 1991 | 9.095115667 | 13.6251206 | 4.502137545 |
| Deaths | Global | Male | 75-79 years | Colon and rectum cancer | High fasting plasma glucose | Number | 1992 | 2817.199839 | 4263.115449 | 1410.52398 |
| Deaths | Global | Female | 75-79 years | Colon and rectum cancer | High fasting plasma glucose | Number | 1992 | 2805.872311 | 4213.80537 | 1397.125102 |
| Deaths | Global | Both | 75-79 years | Colon and rectum cancer | High fasting plasma glucose | Number | 1992 | 5623.07215 | 8403.005139 | 2804.807282 |
| Deaths | Global | Male | 75-79 years | Colon and rectum cancer | High fasting plasma glucose | Rate | 1992 | 11.02133724 | 16.67799082 | 5.518195853 |
| Deaths | Global | Female | 75-79 years | Colon and rectum cancer | High fasting plasma glucose | Rate | 1992 | 7.753060031 | 11.64339727 | 3.860473175 |
| Deaths | Global | Both | 75-79 years | Colon and rectum cancer | High fasting plasma glucose | Rate | 1992 | 9.105918452 | 13.60770011 | 4.54206272 |
| Deaths | Global | Male | 75-79 years | Colon and rectum cancer | High fasting plasma glucose | Number | 1993 | 2816.808888 | 4215.255265 | 1400.931196 |
| Deaths | Global | Female | 75-79 years | Colon and rectum cancer | High fasting plasma glucose | Number | 1993 | 2764.490333 | 4124.604942 | 1382.498903 |
| Deaths | Global | Both | 75-79 years | Colon and rectum cancer | High fasting plasma glucose | Number | 1993 | 5581.299221 | 8305.142624 | 2778.667275 |
| Deaths | Global | Male | 75-79 years | Colon and rectum cancer | High fasting plasma glucose | Rate | 1993 | 10.9595488 | 16.40057868 | 5.450697728 |
| Deaths | Global | Female | 75-79 years | Colon and rectum cancer | High fasting plasma glucose | Rate | 1993 | 7.687633343 | 11.46990825 | 3.844522273 |
| Deaths | Global | Both | 75-79 years | Colon and rectum cancer | High fasting plasma glucose | Rate | 1993 | 9.051426489 | 13.46879731 | 4.506280989 |
| Deaths | Global | Male | 75-79 years | Colon and rectum cancer | High fasting plasma glucose | Number | 1994 | 2819.344259 | 4241.876443 | 1408.750923 |
| Deaths | Global | Female | 75-79 years | Colon and rectum cancer | High fasting plasma glucose | Number | 1994 | 2745.582375 | 4088.728304 | 1345.144892 |
| Deaths | Global | Both | 75-79 years | Colon and rectum cancer | High fasting plasma glucose | Number | 1994 | 5564.926634 | 8310.544705 | 2775.163447 |
| Deaths | Global | Male | 75-79 years | Colon and rectum cancer | High fasting plasma glucose | Rate | 1994 | 10.83084763 | 16.29567488 | 5.411884891 |
| Deaths | Global | Female | 75-79 years | Colon and rectum cancer | High fasting plasma glucose | Rate | 1994 | 7.589691848 | 11.30258854 | 3.718422478 |
| Deaths | Global | Both | 75-79 years | Colon and rectum cancer | High fasting plasma glucose | Rate | 1994 | 8.945987657 | 13.35975032 | 4.461258805 |
| Deaths | Global | Male | 75-79 years | Colon and rectum cancer | High fasting plasma glucose | Number | 1995 | 2921.264301 | 4365.040837 | 1453.843052 |
| Deaths | Global | Female | 75-79 years | Colon and rectum cancer | High fasting plasma glucose | Number | 1995 | 2796.796783 | 4189.643366 | 1406.110969 |
| Deaths | Global | Both | 75-79 years | Colon and rectum cancer | High fasting plasma glucose | Number | 1995 | 5718.061084 | 8551.6103 | 2848.174567 |
| Deaths | Global | Male | 75-79 years | Colon and rectum cancer | High fasting plasma glucose | Rate | 1995 | 10.90010566 | 16.28726518 | 5.424720684 |
| Deaths | Global | Female | 75-79 years | Colon and rectum cancer | High fasting plasma glucose | Rate | 1995 | 7.557709182 | 11.32156127 | 3.799696083 |
| Deaths | Global | Both | 75-79 years | Colon and rectum cancer | High fasting plasma glucose | Rate | 1995 | 8.961605902 | 13.40247336 | 4.463788972 |
| Deaths | Global | Male | 75-79 years | Colon and rectum cancer | High fasting plasma glucose | Number | 1996 | 3081.622865 | 4645.721241 | 1535.879494 |
| Deaths | Global | Female | 75-79 years | Colon and rectum cancer | High fasting plasma glucose | Number | 1996 | 2911.041292 | 4358.223789 | 1454.721657 |
| Deaths | Global | Both | 75-79 years | Colon and rectum cancer | High fasting plasma glucose | Number | 1996 | 5992.664158 | 8985.217236 | 2988.806302 |
| Deaths | Global | Male | 75-79 years | Colon and rectum cancer | High fasting plasma glucose | Rate | 1996 | 11.04800728 | 16.65549755 | 5.506322016 |
| Deaths | Global | Female | 75-79 years | Colon and rectum cancer | High fasting plasma glucose | Rate | 1996 | 7.573845566 | 11.33907444 | 3.784844001 |
| Deaths | Global | Both | 75-79 years | Colon and rectum cancer | High fasting plasma glucose | Rate | 1996 | 9.034830093 | 13.54654774 | 4.506068821 |
| Deaths | Global | Male | 75-79 years | Colon and rectum cancer | High fasting plasma glucose | Number | 1997 | 3311.512897 | 4949.149028 | 1627.712378 |
| Deaths | Global | Female | 75-79 years | Colon and rectum cancer | High fasting plasma glucose | Number | 1997 | 3091.369089 | 4651.201795 | 1566.360865 |
| Deaths | Global | Both | 75-79 years | Colon and rectum cancer | High fasting plasma glucose | Number | 1997 | 6402.881986 | 9604.302414 | 3170.849873 |
| Deaths | Global | Male | 75-79 years | Colon and rectum cancer | High fasting plasma glucose | Rate | 1997 | 11.35265126 | 16.96685615 | 5.58018391 |
| Deaths | Global | Female | 75-79 years | Colon and rectum cancer | High fasting plasma glucose | Rate | 1997 | 7.693248923 | 11.57508281 | 3.898080005 |
| Deaths | Global | Both | 75-79 years | Colon and rectum cancer | High fasting plasma glucose | Rate | 1997 | 9.232388004 | 13.84855235 | 4.57208432 |
| Deaths | Global | Male | 75-79 years | Colon and rectum cancer | High fasting plasma glucose | Number | 1998 | 3603.215042 | 5406.791163 | 1791.642157 |
| Deaths | Global | Female | 75-79 years | Colon and rectum cancer | High fasting plasma glucose | Number | 1998 | 3344.335671 | 5003.309673 | 1692.432431 |
| Deaths | Global | Both | 75-79 years | Colon and rectum cancer | High fasting plasma glucose | Number | 1998 | 6947.550714 | 10381.89845 | 3484.075127 |
| Deaths | Global | Male | 75-79 years | Colon and rectum cancer | High fasting plasma glucose | Rate | 1998 | 11.80940478 | 17.72055918 | 5.872041277 |
| Deaths | Global | Female | 75-79 years | Colon and rectum cancer | High fasting plasma glucose | Rate | 1998 | 7.93862429 | 11.87661754 | 4.017415274 |
| Deaths | Global | Both | 75-79 years | Colon and rectum cancer | High fasting plasma glucose | Rate | 1998 | 9.564517679 | 14.2924975 | 4.796438272 |
| Deaths | Global | Male | 75-79 years | Colon and rectum cancer | High fasting plasma glucose | Number | 1999 | 3908.57423 | 5854.1501 | 1943.323715 |
| Deaths | Global | Female | 75-79 years | Colon and rectum cancer | High fasting plasma glucose | Number | 1999 | 3592.453175 | 5490.381488 | 1840.024339 |
| Deaths | Global | Both | 75-79 years | Colon and rectum cancer | High fasting plasma glucose | Number | 1999 | 7501.027405 | 11236.98954 | 3786.275808 |
| Deaths | Global | Male | 75-79 years | Colon and rectum cancer | High fasting plasma glucose | Rate | 1999 | 12.25376677 | 18.35333954 | 6.092512042 |
| Deaths | Global | Female | 75-79 years | Colon and rectum cancer | High fasting plasma glucose | Rate | 1999 | 8.177960528 | 12.49845743 | 4.188682686 |
| Deaths | Global | Both | 75-79 years | Colon and rectum cancer | High fasting plasma glucose | Rate | 1999 | 9.892500494 | 14.81955984 | 4.993414006 |
| Deaths | Global | Male | 75-79 years | Colon and rectum cancer | High fasting plasma glucose | Number | 2000 | 4151.209611 | 6241.349336 | 2067.205315 |
| Deaths | Global | Female | 75-79 years | Colon and rectum cancer | High fasting plasma glucose | Number | 2000 | 3749.07706 | 5664.369993 | 1909.163259 |
| Deaths | Global | Both | 75-79 years | Colon and rectum cancer | High fasting plasma glucose | Number | 2000 | 7900.286671 | 11912.90672 | 3991.427406 |
| Deaths | Global | Male | 75-79 years | Colon and rectum cancer | High fasting plasma glucose | Rate | 2000 | 12.48590269 | 18.7725718 | 6.217687575 |
| Deaths | Global | Female | 75-79 years | Colon and rectum cancer | High fasting plasma glucose | Rate | 2000 | 8.23369754 | 12.44005086 | 4.192891365 |
| Deaths | Global | Both | 75-79 years | Colon and rectum cancer | High fasting plasma glucose | Rate | 2000 | 10.02822519 | 15.12164257 | 5.06651651 |
| Deaths | Global | Male | 75-79 years | Colon and rectum cancer | High fasting plasma glucose | Number | 2001 | 4337.002043 | 6514.260985 | 2183.854748 |
| Deaths | Global | Female | 75-79 years | Colon and rectum cancer | High fasting plasma glucose | Number | 2001 | 3857.690335 | 5821.912418 | 1994.099636 |
| Deaths | Global | Both | 75-79 years | Colon and rectum cancer | High fasting plasma glucose | Number | 2001 | 8194.692378 | 12328.89638 | 4189.709911 |
| Deaths | Global | Male | 75-79 years | Colon and rectum cancer | High fasting plasma glucose | Rate | 2001 | 12.53383605 | 18.82606426 | 6.31128994 |
| Deaths | Global | Female | 75-79 years | Colon and rectum cancer | High fasting plasma glucose | Rate | 2001 | 8.213240529 | 12.39518024 | 4.245550712 |
| Deaths | Global | Both | 75-79 years | Colon and rectum cancer | High fasting plasma glucose | Rate | 2001 | 10.04602206 | 15.11421776 | 5.136241394 |
| Deaths | Global | Male | 75-79 years | Colon and rectum cancer | High fasting plasma glucose | Number | 2002 | 4534.814408 | 6833.02419 | 2268.790648 |
| Deaths | Global | Female | 75-79 years | Colon and rectum cancer | High fasting plasma glucose | Number | 2002 | 3957.637822 | 5987.843245 | 2019.419699 |
| Deaths | Global | Both | 75-79 years | Colon and rectum cancer | High fasting plasma glucose | Number | 2002 | 8492.45223 | 12771.76351 | 4282.327981 |
| Deaths | Global | Male | 75-79 years | Colon and rectum cancer | High fasting plasma glucose | Rate | 2002 | 12.57577061 | 18.94907643 | 6.291721818 |
| Deaths | Global | Female | 75-79 years | Colon and rectum cancer | High fasting plasma glucose | Rate | 2002 | 8.181338898 | 12.37823597 | 4.174600527 |
| Deaths | Global | Both | 75-79 years | Colon and rectum cancer | High fasting plasma glucose | Rate | 2002 | 10.05810783 | 15.12634645 | 5.071811467 |
| Deaths | Global | Male | 75-79 years | Colon and rectum cancer | High fasting plasma glucose | Number | 2003 | 4754.180582 | 7127.917392 | 2398.881864 |
| Deaths | Global | Female | 75-79 years | Colon and rectum cancer | High fasting plasma glucose | Number | 2003 | 4054.784919 | 6072.368966 | 2089.538084 |
| Deaths | Global | Both | 75-79 years | Colon and rectum cancer | High fasting plasma glucose | Number | 2003 | 8808.965501 | 13156.75812 | 4480.445715 |
| Deaths | Global | Male | 75-79 years | Colon and rectum cancer | High fasting plasma glucose | Rate | 2003 | 12.64252083 | 18.95486352 | 6.3792095 |
| Deaths | Global | Female | 75-79 years | Colon and rectum cancer | High fasting plasma glucose | Rate | 2003 | 8.151231329 | 12.20712939 | 4.200545439 |
| Deaths | Global | Both | 75-79 years | Colon and rectum cancer | High fasting plasma glucose | Rate | 2003 | 10.08477701 | 15.06226489 | 5.129353262 |
| Deaths | Global | Male | 75-79 years | Colon and rectum cancer | High fasting plasma glucose | Number | 2004 | 4944.21418 | 7460.278595 | 2471.760926 |
| Deaths | Global | Female | 75-79 years | Colon and rectum cancer | High fasting plasma glucose | Number | 2004 | 4122.924888 | 6174.800553 | 2084.62442 |
| Deaths | Global | Both | 75-79 years | Colon and rectum cancer | High fasting plasma glucose | Number | 2004 | 9067.139068 | 13517.13698 | 4556.385346 |
| Deaths | Global | Male | 75-79 years | Colon and rectum cancer | High fasting plasma glucose | Rate | 2004 | 12.64382577 | 19.0781506 | 6.321027638 |
| Deaths | Global | Female | 75-79 years | Colon and rectum cancer | High fasting plasma glucose | Rate | 2004 | 8.063515499 | 12.07652366 | 4.077057375 |
| Deaths | Global | Both | 75-79 years | Colon and rectum cancer | High fasting plasma glucose | Rate | 2004 | 10.04842887 | 14.98002715 | 5.049499484 |
| Deaths | Global | Male | 75-79 years | Colon and rectum cancer | High fasting plasma glucose | Number | 2005 | 5118.130483 | 7700.074464 | 2600.837119 |
| Deaths | Global | Female | 75-79 years | Colon and rectum cancer | High fasting plasma glucose | Number | 2005 | 4196.718824 | 6320.214516 | 2117.138607 |
| Deaths | Global | Both | 75-79 years | Colon and rectum cancer | High fasting plasma glucose | Number | 2005 | 9314.849306 | 13892.96659 | 4675.987405 |
| Deaths | Global | Male | 75-79 years | Colon and rectum cancer | High fasting plasma glucose | Rate | 2005 | 12.64189462 | 19.01935293 | 6.424124768 |
| Deaths | Global | Female | 75-79 years | Colon and rectum cancer | High fasting plasma glucose | Rate | 2005 | 8.015465777 | 12.07120736 | 4.043599955 |
| Deaths | Global | Both | 75-79 years | Colon and rectum cancer | High fasting plasma glucose | Rate | 2005 | 10.03287873 | 14.96389736 | 5.036433014 |
| Deaths | Global | Male | 75-79 years | Colon and rectum cancer | High fasting plasma glucose | Number | 2006 | 5226.220292 | 7805.008823 | 2647.638614 |
| Deaths | Global | Female | 75-79 years | Colon and rectum cancer | High fasting plasma glucose | Number | 2006 | 4230.435536 | 6350.082413 | 2131.363858 |
| Deaths | Global | Both | 75-79 years | Colon and rectum cancer | High fasting plasma glucose | Number | 2006 | 9456.655828 | 14058.85669 | 4772.252323 |
| Deaths | Global | Male | 75-79 years | Colon and rectum cancer | High fasting plasma glucose | Rate | 2006 | 12.50015141 | 18.66813616 | 6.332661407 |
| Deaths | Global | Female | 75-79 years | Colon and rectum cancer | High fasting plasma glucose | Rate | 2006 | 7.896585619 | 11.85314586 | 3.978431309 |
| Deaths | Global | Both | 75-79 years | Colon and rectum cancer | High fasting plasma glucose | Rate | 2006 | 9.914484195 | 14.73949301 | 5.003293035 |
| Deaths | Global | Male | 75-79 years | Colon and rectum cancer | High fasting plasma glucose | Number | 2007 | 5352.103968 | 7984.85868 | 2698.84298 |
| Deaths | Global | Female | 75-79 years | Colon and rectum cancer | High fasting plasma glucose | Number | 2007 | 4261.838511 | 6355.633491 | 2156.540875 |
| Deaths | Global | Both | 75-79 years | Colon and rectum cancer | High fasting plasma glucose | Number | 2007 | 9613.942479 | 14265.6368 | 4837.972812 |
| Deaths | Global | Male | 75-79 years | Colon and rectum cancer | High fasting plasma glucose | Rate | 2007 | 12.46321443 | 18.59399715 | 6.284679629 |
| Deaths | Global | Female | 75-79 years | Colon and rectum cancer | High fasting plasma glucose | Rate | 2007 | 7.80491096 | 11.63937896 | 3.949377591 |
| Deaths | Global | Both | 75-79 years | Colon and rectum cancer | High fasting plasma glucose | Rate | 2007 | 9.855623798 | 14.62425533 | 4.959592808 |
| Deaths | Global | Male | 75-79 years | Colon and rectum cancer | High fasting plasma glucose | Number | 2008 | 5519.621958 | 8256.487366 | 2783.933678 |
| Deaths | Global | Female | 75-79 years | Colon and rectum cancer | High fasting plasma glucose | Number | 2008 | 4341.091656 | 6543.618006 | 2218.977981 |
| Deaths | Global | Both | 75-79 years | Colon and rectum cancer | High fasting plasma glucose | Number | 2008 | 9860.713615 | 14727.17743 | 5006.055548 |
| Deaths | Global | Male | 75-79 years | Colon and rectum cancer | High fasting plasma glucose | Rate | 2008 | 12.55595041 | 18.78172939 | 6.332849147 |
| Deaths | Global | Female | 75-79 years | Colon and rectum cancer | High fasting plasma glucose | Rate | 2008 | 7.80340581 | 11.76259586 | 3.988762974 |
| Deaths | Global | Both | 75-79 years | Colon and rectum cancer | High fasting plasma glucose | Rate | 2008 | 9.901215643 | 14.78766804 | 5.026617488 |
| Deaths | Global | Male | 75-79 years | Colon and rectum cancer | High fasting plasma glucose | Number | 2009 | 5660.529463 | 8558.738166 | 2883.657651 |
| Deaths | Global | Female | 75-79 years | Colon and rectum cancer | High fasting plasma glucose | Number | 2009 | 4372.800904 | 6524.415423 | 2208.227312 |
| Deaths | Global | Both | 75-79 years | Colon and rectum cancer | High fasting plasma glucose | Number | 2009 | 10033.33037 | 15030.55925 | 5111.531316 |
| Deaths | Global | Male | 75-79 years | Colon and rectum cancer | High fasting plasma glucose | Rate | 2009 | 12.57662916 | 19.01590244 | 6.406943581 |
| Deaths | Global | Female | 75-79 years | Colon and rectum cancer | High fasting plasma glucose | Rate | 2009 | 7.718593529 | 11.51648835 | 3.897824168 |
| Deaths | Global | Both | 75-79 years | Colon and rectum cancer | High fasting plasma glucose | Rate | 2009 | 9.869386068 | 14.7849604 | 5.028009057 |
| Deaths | Global | Male | 75-79 years | Colon and rectum cancer | High fasting plasma glucose | Number | 2010 | 5826.124718 | 8725.657874 | 2902.693934 |
| Deaths | Global | Female | 75-79 years | Colon and rectum cancer | High fasting plasma glucose | Number | 2010 | 4421.990179 | 6637.291785 | 2239.043867 |
| Deaths | Global | Both | 75-79 years | Colon and rectum cancer | High fasting plasma glucose | Number | 2010 | 10248.1149 | 15313.91263 | 5173.237995 |
| Deaths | Global | Male | 75-79 years | Colon and rectum cancer | High fasting plasma glucose | Rate | 2010 | 12.63013648 | 18.9158755 | 6.292591101 |
| Deaths | Global | Female | 75-79 years | Colon and rectum cancer | High fasting plasma glucose | Rate | 2010 | 7.638226634 | 11.46477872 | 3.86756275 |
| Deaths | Global | Both | 75-79 years | Colon and rectum cancer | High fasting plasma glucose | Rate | 2010 | 9.851906156 | 14.72185193 | 4.973232224 |
| Deaths | Global | Male | 75-79 years | Colon and rectum cancer | High fasting plasma glucose | Number | 2011 | 5981.298886 | 8915.028199 | 3011.093214 |
| Deaths | Global | Female | 75-79 years | Colon and rectum cancer | High fasting plasma glucose | Number | 2011 | 4484.837001 | 6708.777978 | 2270.816294 |
| Deaths | Global | Both | 75-79 years | Colon and rectum cancer | High fasting plasma glucose | Number | 2011 | 10466.13589 | 15582.8628 | 5295.680169 |
| Deaths | Global | Male | 75-79 years | Colon and rectum cancer | High fasting plasma glucose | Rate | 2011 | 12.62313395 | 18.81457476 | 6.354712193 |
| Deaths | Global | Female | 75-79 years | Colon and rectum cancer | High fasting plasma glucose | Rate | 2011 | 7.550483367 | 11.29461707 | 3.823051017 |
| Deaths | Global | Both | 75-79 years | Colon and rectum cancer | High fasting plasma glucose | Rate | 2011 | 9.801437648 | 14.59320419 | 4.959354583 |
| Deaths | Global | Male | 75-79 years | Colon and rectum cancer | High fasting plasma glucose | Number | 2012 | 6153.402028 | 9281.930994 | 3076.03389 |
| Deaths | Global | Female | 75-79 years | Colon and rectum cancer | High fasting plasma glucose | Number | 2012 | 4561.543294 | 6897.47563 | 2295.635586 |
| Deaths | Global | Both | 75-79 years | Colon and rectum cancer | High fasting plasma glucose | Number | 2012 | 10714.94532 | 16111.56763 | 5386.333323 |
| Deaths | Global | Male | 75-79 years | Colon and rectum cancer | High fasting plasma glucose | Rate | 2012 | 12.61823602 | 19.03363301 | 6.307749997 |
| Deaths | Global | Female | 75-79 years | Colon and rectum cancer | High fasting plasma glucose | Rate | 2012 | 7.456384901 | 11.27474406 | 3.752489327 |
| Deaths | Global | Both | 75-79 years | Colon and rectum cancer | High fasting plasma glucose | Rate | 2012 | 9.745973024 | 14.65456881 | 4.899237251 |
| Deaths | Global | Male | 75-79 years | Colon and rectum cancer | High fasting plasma glucose | Number | 2013 | 6319.910774 | 9440.876896 | 3211.941324 |
| Deaths | Global | Female | 75-79 years | Colon and rectum cancer | High fasting plasma glucose | Number | 2013 | 4659.285383 | 6940.027793 | 2339.748579 |
| Deaths | Global | Both | 75-79 years | Colon and rectum cancer | High fasting plasma glucose | Number | 2013 | 10979.19616 | 16397.66502 | 5581.45723 |
| Deaths | Global | Male | 75-79 years | Colon and rectum cancer | High fasting plasma glucose | Rate | 2013 | 12.60512114 | 18.82991726 | 6.40624701 |
| Deaths | Global | Female | 75-79 years | Colon and rectum cancer | High fasting plasma glucose | Rate | 2013 | 7.404237357 | 11.02864685 | 3.718178307 |
| Deaths | Global | Both | 75-79 years | Colon and rectum cancer | High fasting plasma glucose | Rate | 2013 | 9.710523376 | 14.50287501 | 4.936506292 |
| Deaths | Global | Male | 75-79 years | Colon and rectum cancer | High fasting plasma glucose | Number | 2014 | 6442.162579 | 9683.013615 | 3222.216925 |
| Deaths | Global | Female | 75-79 years | Colon and rectum cancer | High fasting plasma glucose | Number | 2014 | 4759.930126 | 7126.578356 | 2393.900788 |
| Deaths | Global | Both | 75-79 years | Colon and rectum cancer | High fasting plasma glucose | Number | 2014 | 11202.0927 | 16879.56186 | 5642.918256 |
| Deaths | Global | Male | 75-79 years | Colon and rectum cancer | High fasting plasma glucose | Rate | 2014 | 12.5237425 | 18.82404668 | 6.264078956 |
| Deaths | Global | Female | 75-79 years | Colon and rectum cancer | High fasting plasma glucose | Rate | 2014 | 7.378130642 | 11.04655421 | 3.710666396 |
| Deaths | Global | Both | 75-79 years | Colon and rectum cancer | High fasting plasma glucose | Rate | 2014 | 9.660837816 | 14.55716479 | 4.86652981 |
| Deaths | Global | Male | 75-79 years | Colon and rectum cancer | High fasting plasma glucose | Number | 2015 | 6583.313962 | 9904.48962 | 3309.923317 |
| Deaths | Global | Female | 75-79 years | Colon and rectum cancer | High fasting plasma glucose | Number | 2015 | 4857.178496 | 7208.47947 | 2475.080944 |
| Deaths | Global | Both | 75-79 years | Colon and rectum cancer | High fasting plasma glucose | Number | 2015 | 11440.49246 | 16996.1916 | 5811.720155 |
| Deaths | Global | Male | 75-79 years | Colon and rectum cancer | High fasting plasma glucose | Rate | 2015 | 12.48390826 | 18.78183853 | 6.276592503 |
| Deaths | Global | Female | 75-79 years | Colon and rectum cancer | High fasting plasma glucose | Rate | 2015 | 7.354273773 | 10.91438817 | 3.747530153 |
| Deaths | Global | Both | 75-79 years | Colon and rectum cancer | High fasting plasma glucose | Rate | 2015 | 9.631661058 | 14.30896068 | 4.892841711 |
| Deaths | Global | Male | 75-79 years | Colon and rectum cancer | High fasting plasma glucose | Number | 2016 | 6709.621234 | 10140.77002 | 3422.683758 |
| Deaths | Global | Female | 75-79 years | Colon and rectum cancer | High fasting plasma glucose | Number | 2016 | 4950.12973 | 7424.237179 | 2543.328458 |
| Deaths | Global | Both | 75-79 years | Colon and rectum cancer | High fasting plasma glucose | Number | 2016 | 11659.75096 | 17614.47251 | 5947.831565 |
| Deaths | Global | Male | 75-79 years | Colon and rectum cancer | High fasting plasma glucose | Rate | 2016 | 12.42390338 | 18.77720703 | 6.33762933 |
| Deaths | Global | Female | 75-79 years | Colon and rectum cancer | High fasting plasma glucose | Rate | 2016 | 7.353701547 | 11.02913002 | 3.778260255 |
| Deaths | Global | Both | 75-79 years | Colon and rectum cancer | High fasting plasma glucose | Rate | 2016 | 9.610697774 | 14.51895261 | 4.902575686 |
| Deaths | Global | Male | 75-79 years | Colon and rectum cancer | High fasting plasma glucose | Number | 2017 | 6762.650343 | 10197.58519 | 3421.643808 |
| Deaths | Global | Female | 75-79 years | Colon and rectum cancer | High fasting plasma glucose | Number | 2017 | 5008.530515 | 7514.095243 | 2573.735017 |
| Deaths | Global | Both | 75-79 years | Colon and rectum cancer | High fasting plasma glucose | Number | 2017 | 11771.18086 | 17681.49877 | 5998.406483 |
| Deaths | Global | Male | 75-79 years | Colon and rectum cancer | High fasting plasma glucose | Rate | 2017 | 12.26304985 | 18.49178786 | 6.20463671 |
| Deaths | Global | Female | 75-79 years | Colon and rectum cancer | High fasting plasma glucose | Rate | 2017 | 7.336377226 | 11.00646927 | 3.769946277 |
| Deaths | Global | Both | 75-79 years | Colon and rectum cancer | High fasting plasma glucose | Rate | 2017 | 9.537779359 | 14.32670486 | 4.860300613 |
| Deaths | Global | Male | 75-79 years | Colon and rectum cancer | High fasting plasma glucose | Number | 2018 | 6868.277096 | 10244.10256 | 3429.668693 |
| Deaths | Global | Female | 75-79 years | Colon and rectum cancer | High fasting plasma glucose | Number | 2018 | 5057.109728 | 7636.246704 | 2531.889067 |
| Deaths | Global | Both | 75-79 years | Colon and rectum cancer | High fasting plasma glucose | Number | 2018 | 11925.38682 | 17942.98404 | 6002.388598 |
| Deaths | Global | Male | 75-79 years | Colon and rectum cancer | High fasting plasma glucose | Rate | 2018 | 12.17907822 | 18.16521446 | 6.081612999 |
| Deaths | Global | Female | 75-79 years | Colon and rectum cancer | High fasting plasma glucose | Rate | 2018 | 7.320779197 | 11.05439253 | 3.665216261 |
| Deaths | Global | Both | 75-79 years | Colon and rectum cancer | High fasting plasma glucose | Rate | 2018 | 9.504351939 | 14.30028541 | 4.783812429 |
| Deaths | Global | Male | 75-79 years | Colon and rectum cancer | High fasting plasma glucose | Number | 2019 | 6968.037116 | 10545.59731 | 3555.90176 |
| Deaths | Global | Female | 75-79 years | Colon and rectum cancer | High fasting plasma glucose | Number | 2019 | 5115.975117 | 7740.894029 | 2600.207612 |
| Deaths | Global | Both | 75-79 years | Colon and rectum cancer | High fasting plasma glucose | Number | 2019 | 12084.01223 | 18273.65387 | 6219.938395 |
| Deaths | Global | Male | 75-79 years | Colon and rectum cancer | High fasting plasma glucose | Rate | 2019 | 12.08901504 | 18.29581019 | 6.169219413 |
| Deaths | Global | Female | 75-79 years | Colon and rectum cancer | High fasting plasma glucose | Rate | 2019 | 7.305366993 | 11.05362525 | 3.712971706 |
| Deaths | Global | Both | 75-79 years | Colon and rectum cancer | High fasting plasma glucose | Rate | 2019 | 9.465053231 | 14.31321843 | 4.871895763 |
| Deaths | Global | Male | 75-79 years | Colon and rectum cancer | High fasting plasma glucose | Number | 2020 | 7038.260269 | 10749.30741 | 3528.161355 |
| Deaths | Global | Female | 75-79 years | Colon and rectum cancer | High fasting plasma glucose | Number | 2020 | 5121.247489 | 7682.770086 | 2545.671069 |
| Deaths | Global | Both | 75-79 years | Colon and rectum cancer | High fasting plasma glucose | Number | 2020 | 12159.50776 | 18466.7973 | 6083.997344 |
| Deaths | Global | Male | 75-79 years | Colon and rectum cancer | High fasting plasma glucose | Rate | 2020 | 12.00809706 | 18.33957851 | 6.019456852 |
| Deaths | Global | Female | 75-79 years | Colon and rectum cancer | High fasting plasma glucose | Rate | 2020 | 7.238739344 | 10.85937952 | 3.598234486 |
| Deaths | Global | Both | 75-79 years | Colon and rectum cancer | High fasting plasma glucose | Rate | 2020 | 9.399714015 | 14.27546385 | 4.703137351 |
| Deaths | Global | Male | 75-79 years | Colon and rectum cancer | High fasting plasma glucose | Number | 2021 | 7077.623009 | 10818.20826 | 3564.622511 |
| Deaths | Global | Female | 75-79 years | Colon and rectum cancer | High fasting plasma glucose | Number | 2021 | 5175.623458 | 7860.920216 | 2653.542592 |
| Deaths | Global | Both | 75-79 years | Colon and rectum cancer | High fasting plasma glucose | Number | 2021 | 12253.24647 | 18764.05127 | 6346.473434 |
| Deaths | Global | Male | 75-79 years | Colon and rectum cancer | High fasting plasma glucose | Rate | 2021 | 11.83811829 | 18.09466665 | 5.962230946 |
| Deaths | Global | Female | 75-79 years | Colon and rectum cancer | High fasting plasma glucose | Rate | 2021 | 7.178626771 | 10.90315259 | 3.680482562 |
| Deaths | Global | Both | 75-79 years | Colon and rectum cancer | High fasting plasma glucose | Rate | 2021 | 9.290898728 | 14.22764983 | 4.812148529 |
| Deaths | Global | Male | 80-84 years | Colon and rectum cancer | High fasting plasma glucose | Number | 1990 | 2018.23105 | 3056.986732 | 1011.328272 |
| Deaths | Global | Female | 80-84 years | Colon and rectum cancer | High fasting plasma glucose | Number | 1990 | 2437.481988 | 3721.300035 | 1224.729572 |
| Deaths | Global | Both | 80-84 years | Colon and rectum cancer | High fasting plasma glucose | Number | 1990 | 4455.713038 | 6791.820906 | 2276.363667 |
| Deaths | Global | Male | 80-84 years | Colon and rectum cancer | High fasting plasma glucose | Rate | 1990 | 15.19358771 | 23.01351772 | 7.613451788 |
| Deaths | Global | Female | 80-84 years | Colon and rectum cancer | High fasting plasma glucose | Rate | 1990 | 11.03308073 | 16.84418753 | 5.543647219 |
| Deaths | Global | Both | 80-84 years | Colon and rectum cancer | High fasting plasma glucose | Rate | 1990 | 12.59532511 | 19.19899052 | 6.434781637 |
| Deaths | Global | Male | 80-84 years | Colon and rectum cancer | High fasting plasma glucose | Number | 1991 | 2089.846418 | 3161.224875 | 1049.637267 |
| Deaths | Global | Female | 80-84 years | Colon and rectum cancer | High fasting plasma glucose | Number | 1991 | 2520.537745 | 3834.999721 | 1267.715943 |
| Deaths | Global | Both | 80-84 years | Colon and rectum cancer | High fasting plasma glucose | Number | 1991 | 4610.384164 | 6983.45571 | 2353.379934 |
| Deaths | Global | Male | 80-84 years | Colon and rectum cancer | High fasting plasma glucose | Rate | 1991 | 15.25222823 | 23.07141944 | 7.660518506 |
| Deaths | Global | Female | 80-84 years | Colon and rectum cancer | High fasting plasma glucose | Rate | 1991 | 11.06006505 | 16.82789573 | 5.562710105 |
| Deaths | Global | Both | 80-84 years | Colon and rectum cancer | High fasting plasma glucose | Rate | 1991 | 12.63415001 | 19.13723974 | 6.449127461 |
| Deaths | Global | Male | 80-84 years | Colon and rectum cancer | High fasting plasma glucose | Number | 1992 | 2179.889076 | 3283.242505 | 1111.225418 |
| Deaths | Global | Female | 80-84 years | Colon and rectum cancer | High fasting plasma glucose | Number | 1992 | 2620.557153 | 4004.236754 | 1316.115541 |
| Deaths | Global | Both | 80-84 years | Colon and rectum cancer | High fasting plasma glucose | Number | 1992 | 4800.446229 | 7275.793322 | 2441.174013 |
| Deaths | Global | Male | 80-84 years | Colon and rectum cancer | High fasting plasma glucose | Rate | 1992 | 15.45228648 | 23.27347953 | 7.87699415 |
| Deaths | Global | Female | 80-84 years | Colon and rectum cancer | High fasting plasma glucose | Rate | 1992 | 11.19906642 | 17.11228214 | 5.624477735 |
| Deaths | Global | Both | 80-84 years | Colon and rectum cancer | High fasting plasma glucose | Rate | 1992 | 12.79879781 | 19.39848989 | 6.508580895 |
| Deaths | Global | Male | 80-84 years | Colon and rectum cancer | High fasting plasma glucose | Number | 1993 | 2258.590563 | 3411.705138 | 1140.277735 |
| Deaths | Global | Female | 80-84 years | Colon and rectum cancer | High fasting plasma glucose | Number | 1993 | 2714.844003 | 4178.126826 | 1377.416682 |
| Deaths | Global | Both | 80-84 years | Colon and rectum cancer | High fasting plasma glucose | Number | 1993 | 4973.434566 | 7486.879878 | 2547.73806 |
| Deaths | Global | Male | 80-84 years | Colon and rectum cancer | High fasting plasma glucose | Rate | 1993 | 15.56225178 | 23.50749854 | 7.85679773 |
| Deaths | Global | Female | 80-84 years | Colon and rectum cancer | High fasting plasma glucose | Rate | 1993 | 11.28483068 | 17.3672792 | 5.72552751 |
| Deaths | Global | Both | 80-84 years | Colon and rectum cancer | High fasting plasma glucose | Rate | 1993 | 12.8943243 | 19.4107826 | 6.605367042 |
| Deaths | Global | Male | 80-84 years | Colon and rectum cancer | High fasting plasma glucose | Number | 1994 | 2343.994023 | 3544.093194 | 1192.779621 |
| Deaths | Global | Female | 80-84 years | Colon and rectum cancer | High fasting plasma glucose | Number | 1994 | 2808.476998 | 4281.585564 | 1426.655274 |
| Deaths | Global | Both | 80-84 years | Colon and rectum cancer | High fasting plasma glucose | Number | 1994 | 5152.471021 | 7801.116909 | 2621.261609 |
| Deaths | Global | Male | 80-84 years | Colon and rectum cancer | High fasting plasma glucose | Rate | 1994 | 15.76202369 | 23.83200654 | 8.020763049 |
| Deaths | Global | Female | 80-84 years | Colon and rectum cancer | High fasting plasma glucose | Rate | 1994 | 11.42844168 | 17.42291318 | 5.805440671 |
| Deaths | Global | Both | 80-84 years | Colon and rectum cancer | High fasting plasma glucose | Rate | 1994 | 13.06221936 | 19.77689926 | 6.645257002 |
| Deaths | Global | Male | 80-84 years | Colon and rectum cancer | High fasting plasma glucose | Number | 1995 | 2427.906452 | 3667.620206 | 1239.583893 |
| Deaths | Global | Female | 80-84 years | Colon and rectum cancer | High fasting plasma glucose | Number | 1995 | 2883.862781 | 4443.630997 | 1459.289858 |
| Deaths | Global | Both | 80-84 years | Colon and rectum cancer | High fasting plasma glucose | Number | 1995 | 5311.769232 | 8019.663348 | 2705.898116 |
| Deaths | Global | Male | 80-84 years | Colon and rectum cancer | High fasting plasma glucose | Rate | 1995 | 16.03568332 | 24.2236665 | 8.187125472 |
| Deaths | Global | Female | 80-84 years | Colon and rectum cancer | High fasting plasma glucose | Rate | 1995 | 11.56336944 | 17.81754223 | 5.851286637 |
| Deaths | Global | Both | 80-84 years | Colon and rectum cancer | High fasting plasma glucose | Rate | 1995 | 13.25282164 | 20.00899574 | 6.751194101 |
| Deaths | Global | Male | 80-84 years | Colon and rectum cancer | High fasting plasma glucose | Number | 1996 | 2472.036607 | 3723.479997 | 1261.01496 |
| Deaths | Global | Female | 80-84 years | Colon and rectum cancer | High fasting plasma glucose | Number | 1996 | 2908.609489 | 4447.220206 | 1476.593892 |
| Deaths | Global | Both | 80-84 years | Colon and rectum cancer | High fasting plasma glucose | Number | 1996 | 5380.646095 | 8130.485907 | 2763.39814 |
| Deaths | Global | Male | 80-84 years | Colon and rectum cancer | High fasting plasma glucose | Rate | 1996 | 16.15737932 | 24.33688827 | 8.242069306 |
| Deaths | Global | Female | 80-84 years | Colon and rectum cancer | High fasting plasma glucose | Rate | 1996 | 11.64992728 | 17.81256378 | 5.914238929 |
| Deaths | Global | Both | 80-84 years | Colon and rectum cancer | High fasting plasma glucose | Rate | 1996 | 13.36258757 | 20.19168851 | 6.862772422 |
| Deaths | Global | Male | 80-84 years | Colon and rectum cancer | High fasting plasma glucose | Number | 1997 | 2511.655107 | 3804.689357 | 1263.036785 |
| Deaths | Global | Female | 80-84 years | Colon and rectum cancer | High fasting plasma glucose | Number | 1997 | 2904.634774 | 4450.397155 | 1472.777032 |
| Deaths | Global | Both | 80-84 years | Colon and rectum cancer | High fasting plasma glucose | Number | 1997 | 5416.28988 | 8214.566196 | 2778.731009 |
| Deaths | Global | Male | 80-84 years | Colon and rectum cancer | High fasting plasma glucose | Rate | 1997 | 16.27245801 | 24.64974098 | 8.182936022 |
| Deaths | Global | Female | 80-84 years | Colon and rectum cancer | High fasting plasma glucose | Rate | 1997 | 11.65992964 | 17.86500602 | 5.912094949 |
| Deaths | Global | Both | 80-84 years | Colon and rectum cancer | High fasting plasma glucose | Rate | 1997 | 13.42451466 | 20.3601666 | 6.887208032 |
| Deaths | Global | Male | 80-84 years | Colon and rectum cancer | High fasting plasma glucose | Number | 1998 | 2563.646771 | 3842.865209 | 1299.152898 |
| Deaths | Global | Female | 80-84 years | Colon and rectum cancer | High fasting plasma glucose | Number | 1998 | 2908.419423 | 4477.185956 | 1478.807418 |
| Deaths | Global | Both | 80-84 years | Colon and rectum cancer | High fasting plasma glucose | Number | 1998 | 5472.066194 | 8287.264947 | 2822.685003 |
| Deaths | Global | Male | 80-84 years | Colon and rectum cancer | High fasting plasma glucose | Rate | 1998 | 16.43032591 | 24.62879385 | 8.326227222 |
| Deaths | Global | Female | 80-84 years | Colon and rectum cancer | High fasting plasma glucose | Rate | 1998 | 11.70394666 | 18.01691502 | 5.950958446 |
| Deaths | Global | Both | 80-84 years | Colon and rectum cancer | High fasting plasma glucose | Rate | 1998 | 13.52695802 | 20.48613468 | 6.977682685 |
| Deaths | Global | Male | 80-84 years | Colon and rectum cancer | High fasting plasma glucose | Number | 1999 | 2639.528175 | 3963.869752 | 1327.33874 |
| Deaths | Global | Female | 80-84 years | Colon and rectum cancer | High fasting plasma glucose | Number | 1999 | 2925.040146 | 4467.113379 | 1465.645748 |
| Deaths | Global | Both | 80-84 years | Colon and rectum cancer | High fasting plasma glucose | Number | 1999 | 5564.568321 | 8356.971671 | 2841.699073 |
| Deaths | Global | Male | 80-84 years | Colon and rectum cancer | High fasting plasma glucose | Rate | 1999 | 16.60030785 | 24.92925016 | 8.347791821 |
| Deaths | Global | Female | 80-84 years | Colon and rectum cancer | High fasting plasma glucose | Rate | 1999 | 11.63458641 | 17.76830881 | 5.829725832 |
| Deaths | Global | Both | 80-84 years | Colon and rectum cancer | High fasting plasma glucose | Rate | 1999 | 13.55843349 | 20.36230631 | 6.923985056 |
| Deaths | Global | Male | 80-84 years | Colon and rectum cancer | High fasting plasma glucose | Number | 2000 | 2772.867336 | 4167.689109 | 1387.26751 |
| Deaths | Global | Female | 80-84 years | Colon and rectum cancer | High fasting plasma glucose | Number | 2000 | 3012.550508 | 4585.644582 | 1511.792455 |
| Deaths | Global | Both | 80-84 years | Colon and rectum cancer | High fasting plasma glucose | Number | 2000 | 5785.417843 | 8746.117202 | 2925.499377 |
| Deaths | Global | Male | 80-84 years | Colon and rectum cancer | High fasting plasma glucose | Rate | 2000 | 16.79795627 | 25.24774933 | 8.404029533 |
| Deaths | Global | Female | 80-84 years | Colon and rectum cancer | High fasting plasma glucose | Rate | 2000 | 11.61619071 | 17.68193492 | 5.829369312 |
| Deaths | Global | Both | 80-84 years | Colon and rectum cancer | High fasting plasma glucose | Rate | 2000 | 13.63159593 | 20.6075929 | 6.893058806 |
| Deaths | Global | Male | 80-84 years | Colon and rectum cancer | High fasting plasma glucose | Number | 2001 | 2941.899985 | 4400.989751 | 1478.113784 |
| Deaths | Global | Female | 80-84 years | Colon and rectum cancer | High fasting plasma glucose | Number | 2001 | 3160.936409 | 4809.440071 | 1589.636598 |
| Deaths | Global | Both | 80-84 years | Colon and rectum cancer | High fasting plasma glucose | Number | 2001 | 6102.836395 | 9197.664187 | 3087.814093 |
| Deaths | Global | Male | 80-84 years | Colon and rectum cancer | High fasting plasma glucose | Rate | 2001 | 16.99249707 | 25.42024061 | 8.537626796 |
| Deaths | Global | Female | 80-84 years | Colon and rectum cancer | High fasting plasma glucose | Rate | 2001 | 11.6391431 | 17.70923359 | 5.853331244 |
| Deaths | Global | Both | 80-84 years | Colon and rectum cancer | High fasting plasma glucose | Rate | 2001 | 13.72326085 | 20.6825051 | 6.943472758 |
| Deaths | Global | Male | 80-84 years | Colon and rectum cancer | High fasting plasma glucose | Number | 2002 | 3141.528204 | 4746.462502 | 1579.39694 |
| Deaths | Global | Female | 80-84 years | Colon and rectum cancer | High fasting plasma glucose | Number | 2002 | 3357.998948 | 5139.677026 | 1672.015013 |
| Deaths | Global | Both | 80-84 years | Colon and rectum cancer | High fasting plasma glucose | Number | 2002 | 6499.527152 | 9799.296275 | 3280.750067 |
| Deaths | Global | Male | 80-84 years | Colon and rectum cancer | High fasting plasma glucose | Rate | 2002 | 17.25151603 | 26.06491766 | 8.673164733 |
| Deaths | Global | Female | 80-84 years | Colon and rectum cancer | High fasting plasma glucose | Rate | 2002 | 11.75590992 | 17.99332908 | 5.853503283 |
| Deaths | Global | Both | 80-84 years | Colon and rectum cancer | High fasting plasma glucose | Rate | 2002 | 13.89544838 | 20.95008027 | 7.013970728 |
| Deaths | Global | Male | 80-84 years | Colon and rectum cancer | High fasting plasma glucose | Number | 2003 | 3382.651081 | 5091.610306 | 1699.532564 |
| Deaths | Global | Female | 80-84 years | Colon and rectum cancer | High fasting plasma glucose | Number | 2003 | 3606.748818 | 5470.834048 | 1795.153835 |
| Deaths | Global | Both | 80-84 years | Colon and rectum cancer | High fasting plasma glucose | Number | 2003 | 6989.399899 | 10512.40606 | 3506.900214 |
| Deaths | Global | Male | 80-84 years | Colon and rectum cancer | High fasting plasma glucose | Rate | 2003 | 17.68774928 | 26.62382977 | 8.886788843 |
| Deaths | Global | Female | 80-84 years | Colon and rectum cancer | High fasting plasma glucose | Rate | 2003 | 11.99756354 | 18.19829504 | 5.971436683 |
| Deaths | Global | Both | 80-84 years | Colon and rectum cancer | High fasting plasma glucose | Rate | 2003 | 14.20996654 | 21.37249843 | 7.129787307 |
| Deaths | Global | Male | 80-84 years | Colon and rectum cancer | High fasting plasma glucose | Number | 2004 | 3611.188173 | 5449.509815 | 1798.25544 |
| Deaths | Global | Female | 80-84 years | Colon and rectum cancer | High fasting plasma glucose | Number | 2004 | 3823.370435 | 5828.02655 | 1895.833926 |
| Deaths | Global | Both | 80-84 years | Colon and rectum cancer | High fasting plasma glucose | Number | 2004 | 7434.558609 | 11246.06374 | 3700.431837 |
| Deaths | Global | Male | 80-84 years | Colon and rectum cancer | High fasting plasma glucose | Rate | 2004 | 18.00179825 | 27.16584447 | 8.964315921 |
| Deaths | Global | Female | 80-84 years | Colon and rectum cancer | High fasting plasma glucose | Rate | 2004 | 12.15231732 | 18.52397752 | 6.02577643 |
| Deaths | Global | Both | 80-84 years | Colon and rectum cancer | High fasting plasma glucose | Rate | 2004 | 14.42980995 | 21.82759879 | 7.182205558 |
| Deaths | Global | Male | 80-84 years | Colon and rectum cancer | High fasting plasma glucose | Number | 2005 | 3796.943867 | 5730.895593 | 1907.304513 |
| Deaths | Global | Female | 80-84 years | Colon and rectum cancer | High fasting plasma glucose | Number | 2005 | 3969.98684 | 6033.497696 | 1971.414404 |
| Deaths | Global | Both | 80-84 years | Colon and rectum cancer | High fasting plasma glucose | Number | 2005 | 7766.930707 | 11689.155 | 3899.745538 |
| Deaths | Global | Male | 80-84 years | Colon and rectum cancer | High fasting plasma glucose | Rate | 2005 | 18.08332327 | 27.29396095 | 9.083727672 |
| Deaths | Global | Female | 80-84 years | Colon and rectum cancer | High fasting plasma glucose | Rate | 2005 | 12.12588524 | 18.42865067 | 6.021467018 |
| Deaths | Global | Both | 80-84 years | Colon and rectum cancer | High fasting plasma glucose | Rate | 2005 | 14.45367861 | 21.75264541 | 7.257135514 |
| Deaths | Global | Male | 80-84 years | Colon and rectum cancer | High fasting plasma glucose | Number | 2006 | 3940.982821 | 5888.340621 | 1962.771423 |
| Deaths | Global | Female | 80-84 years | Colon and rectum cancer | High fasting plasma glucose | Number | 2006 | 4056.931477 | 6193.313116 | 2007.009365 |
| Deaths | Global | Both | 80-84 years | Colon and rectum cancer | High fasting plasma glucose | Number | 2006 | 7997.914298 | 12059.28835 | 3993.895711 |
| Deaths | Global | Male | 80-84 years | Colon and rectum cancer | High fasting plasma glucose | Rate | 2006 | 17.9436621 | 26.81016367 | 8.936681226 |
| Deaths | Global | Female | 80-84 years | Colon and rectum cancer | High fasting plasma glucose | Rate | 2006 | 11.95903638 | 18.25666943 | 5.916269018 |
| Deaths | Global | Both | 80-84 years | Colon and rectum cancer | High fasting plasma glucose | Rate | 2006 | 14.31095522 | 21.57811764 | 7.146420996 |
| Deaths | Global | Male | 80-84 years | Colon and rectum cancer | High fasting plasma glucose | Number | 2007 | 4131.875044 | 6188.612284 | 2047.997576 |
| Deaths | Global | Female | 80-84 years | Colon and rectum cancer | High fasting plasma glucose | Number | 2007 | 4164.112549 | 6332.12913 | 2069.025846 |
| Deaths | Global | Both | 80-84 years | Colon and rectum cancer | High fasting plasma glucose | Number | 2007 | 8295.987593 | 12446.65159 | 4148.943422 |
| Deaths | Global | Male | 80-84 years | Colon and rectum cancer | High fasting plasma glucose | Rate | 2007 | 17.93717359 | 26.86582039 | 8.890706429 |
| Deaths | Global | Female | 80-84 years | Colon and rectum cancer | High fasting plasma glucose | Rate | 2007 | 11.85747431 | 18.03098682 | 5.891632495 |
| Deaths | Global | Both | 80-84 years | Colon and rectum cancer | High fasting plasma glucose | Rate | 2007 | 14.26572051 | 21.40317241 | 7.134493223 |
| Deaths | Global | Male | 80-84 years | Colon and rectum cancer | High fasting plasma glucose | Number | 2008 | 4370.791874 | 6606.64854 | 2168.233657 |
| Deaths | Global | Female | 80-84 years | Colon and rectum cancer | High fasting plasma glucose | Number | 2008 | 4308.437068 | 6563.920173 | 2146.440058 |
| Deaths | Global | Both | 80-84 years | Colon and rectum cancer | High fasting plasma glucose | Number | 2008 | 8679.228943 | 13079.03685 | 4339.841215 |
| Deaths | Global | Male | 80-84 years | Colon and rectum cancer | High fasting plasma glucose | Rate | 2008 | 18.08193538 | 27.33165875 | 8.96996746 |
| Deaths | Global | Female | 80-84 years | Colon and rectum cancer | High fasting plasma glucose | Rate | 2008 | 11.87164741 | 18.08649975 | 5.914390568 |
| Deaths | Global | Both | 80-84 years | Colon and rectum cancer | High fasting plasma glucose | Rate | 2008 | 14.35438209 | 21.63112572 | 7.177566053 |
| Deaths | Global | Male | 80-84 years | Colon and rectum cancer | High fasting plasma glucose | Number | 2009 | 4590.126441 | 6981.752795 | 2304.486367 |
| Deaths | Global | Female | 80-84 years | Colon and rectum cancer | High fasting plasma glucose | Number | 2009 | 4404.143782 | 6711.914405 | 2190.941965 |
| Deaths | Global | Both | 80-84 years | Colon and rectum cancer | High fasting plasma glucose | Number | 2009 | 8994.270223 | 13611.17213 | 4506.095029 |
| Deaths | Global | Male | 80-84 years | Colon and rectum cancer | High fasting plasma glucose | Rate | 2009 | 18.15735796 | 27.61801582 | 9.115954518 |
| Deaths | Global | Female | 80-84 years | Colon and rectum cancer | High fasting plasma glucose | Rate | 2009 | 11.75662147 | 17.9170892 | 5.848600006 |
| Deaths | Global | Both | 80-84 years | Colon and rectum cancer | High fasting plasma glucose | Rate | 2009 | 14.33563011 | 21.69433697 | 7.182095932 |
| Deaths | Global | Male | 80-84 years | Colon and rectum cancer | High fasting plasma glucose | Number | 2010 | 4832.612968 | 7326.472294 | 2422.003652 |
| Deaths | Global | Female | 80-84 years | Colon and rectum cancer | High fasting plasma glucose | Number | 2010 | 4512.763924 | 6863.49802 | 2224.938045 |
| Deaths | Global | Both | 80-84 years | Colon and rectum cancer | High fasting plasma glucose | Number | 2010 | 9345.376892 | 14113.09169 | 4660.98132 |
| Deaths | Global | Male | 80-84 years | Colon and rectum cancer | High fasting plasma glucose | Rate | 2010 | 18.36334672 | 27.83971153 | 9.203321913 |
| Deaths | Global | Female | 80-84 years | Colon and rectum cancer | High fasting plasma glucose | Rate | 2010 | 11.71737126 | 17.82104179 | 5.777041643 |
| Deaths | Global | Both | 80-84 years | Colon and rectum cancer | High fasting plasma glucose | Rate | 2010 | 14.41518769 | 21.76935911 | 7.189535672 |
| Deaths | Global | Male | 80-84 years | Colon and rectum cancer | High fasting plasma glucose | Number | 2011 | 5018.982493 | 7559.116125 | 2508.852779 |
| Deaths | Global | Female | 80-84 years | Colon and rectum cancer | High fasting plasma glucose | Number | 2011 | 4658.473023 | 7146.58403 | 2290.048112 |
| Deaths | Global | Both | 80-84 years | Colon and rectum cancer | High fasting plasma glucose | Number | 2011 | 9677.455516 | 14611.27677 | 4830.77519 |
| Deaths | Global | Male | 80-84 years | Colon and rectum cancer | High fasting plasma glucose | Rate | 2011 | 18.36368715 | 27.65764652 | 9.179507521 |
| Deaths | Global | Female | 80-84 years | Colon and rectum cancer | High fasting plasma glucose | Rate | 2011 | 11.77598661 | 18.06559304 | 5.788930355 |
| Deaths | Global | Both | 80-84 years | Colon and rectum cancer | High fasting plasma glucose | Rate | 2011 | 14.46769345 | 21.84370395 | 7.221957722 |
| Deaths | Global | Male | 80-84 years | Colon and rectum cancer | High fasting plasma glucose | Number | 2012 | 5163.704012 | 7844.360193 | 2590.295407 |
| Deaths | Global | Female | 80-84 years | Colon and rectum cancer | High fasting plasma glucose | Number | 2012 | 4723.716149 | 7188.357229 | 2348.87425 |
| Deaths | Global | Both | 80-84 years | Colon and rectum cancer | High fasting plasma glucose | Number | 2012 | 9887.420161 | 15024.51366 | 4951.985216 |
| Deaths | Global | Male | 80-84 years | Colon and rectum cancer | High fasting plasma glucose | Rate | 2012 | 18.2909002 | 27.78633497 | 9.175358359 |
| Deaths | Global | Female | 80-84 years | Colon and rectum cancer | High fasting plasma glucose | Rate | 2012 | 11.67541329 | 17.76716442 | 5.805615062 |
| Deaths | Global | Both | 80-84 years | Colon and rectum cancer | High fasting plasma glucose | Rate | 2012 | 14.39433491 | 21.87303441 | 7.209214586 |
| Deaths | Global | Male | 80-84 years | Colon and rectum cancer | High fasting plasma glucose | Number | 2013 | 5317.718798 | 8107.998651 | 2691.893932 |
| Deaths | Global | Female | 80-84 years | Colon and rectum cancer | High fasting plasma glucose | Number | 2013 | 4783.106078 | 7305.449057 | 2363.429306 |
| Deaths | Global | Both | 80-84 years | Colon and rectum cancer | High fasting plasma glucose | Number | 2013 | 10100.82488 | 15378.64577 | 5137.455277 |
| Deaths | Global | Male | 80-84 years | Colon and rectum cancer | High fasting plasma glucose | Rate | 2013 | 18.29719648 | 27.89798597 | 9.262263398 |
| Deaths | Global | Female | 80-84 years | Colon and rectum cancer | High fasting plasma glucose | Rate | 2013 | 11.55847409 | 17.65376771 | 5.711275464 |
| Deaths | Global | Both | 80-84 years | Colon and rectum cancer | High fasting plasma glucose | Rate | 2013 | 14.33863037 | 21.83076332 | 7.292876886 |
| Deaths | Global | Male | 80-84 years | Colon and rectum cancer | High fasting plasma glucose | Number | 2014 | 5471.912992 | 8330.404643 | 2753.108648 |
| Deaths | Global | Female | 80-84 years | Colon and rectum cancer | High fasting plasma glucose | Number | 2014 | 4845.246698 | 7403.702575 | 2333.670835 |
| Deaths | Global | Both | 80-84 years | Colon and rectum cancer | High fasting plasma glucose | Number | 2014 | 10317.15969 | 15942.65035 | 5174.884884 |
| Deaths | Global | Male | 80-84 years | Colon and rectum cancer | High fasting plasma glucose | Rate | 2014 | 18.30187724 | 27.86265852 | 9.208307327 |
| Deaths | Global | Female | 80-84 years | Colon and rectum cancer | High fasting plasma glucose | Rate | 2014 | 11.44963499 | 17.49543363 | 5.514616882 |
| Deaths | Global | Both | 80-84 years | Colon and rectum cancer | High fasting plasma glucose | Rate | 2014 | 14.2865267 | 22.07633754 | 7.165841498 |
| Deaths | Global | Male | 80-84 years | Colon and rectum cancer | High fasting plasma glucose | Number | 2015 | 5638.743149 | 8562.814401 | 2852.113755 |
| Deaths | Global | Female | 80-84 years | Colon and rectum cancer | High fasting plasma glucose | Number | 2015 | 4935.033464 | 7517.26861 | 2405.916615 |
| Deaths | Global | Both | 80-84 years | Colon and rectum cancer | High fasting plasma glucose | Number | 2015 | 10573.77661 | 16102.2536 | 5343.296845 |
| Deaths | Global | Male | 80-84 years | Colon and rectum cancer | High fasting plasma glucose | Rate | 2015 | 18.30407703 | 27.79598401 | 9.258323797 |
| Deaths | Global | Female | 80-84 years | Colon and rectum cancer | High fasting plasma glucose | Rate | 2015 | 11.36434512 | 17.31069008 | 5.540320434 |
| Deaths | Global | Both | 80-84 years | Colon and rectum cancer | High fasting plasma glucose | Rate | 2015 | 14.24432052 | 21.69193371 | 7.19815026 |
| Deaths | Global | Male | 80-84 years | Colon and rectum cancer | High fasting plasma glucose | Number | 2016 | 5802.968856 | 8881.949499 | 2960.17034 |
| Deaths | Global | Female | 80-84 years | Colon and rectum cancer | High fasting plasma glucose | Number | 2016 | 5049.989617 | 7723.282258 | 2480.524671 |
| Deaths | Global | Both | 80-84 years | Colon and rectum cancer | High fasting plasma glucose | Number | 2016 | 10852.95847 | 16653.85119 | 5455.913022 |
| Deaths | Global | Male | 80-84 years | Colon and rectum cancer | High fasting plasma glucose | Rate | 2016 | 18.2276252 | 27.8989687 | 9.298150105 |
| Deaths | Global | Female | 80-84 years | Colon and rectum cancer | High fasting plasma glucose | Rate | 2016 | 11.28719103 | 17.26224584 | 5.544200672 |
| Deaths | Global | Both | 80-84 years | Colon and rectum cancer | High fasting plasma glucose | Rate | 2016 | 14.17260668 | 21.74784721 | 7.124740182 |
| Deaths | Global | Male | 80-84 years | Colon and rectum cancer | High fasting plasma glucose | Number | 2017 | 5929.742963 | 9086.629074 | 2980.845271 |
| Deaths | Global | Female | 80-84 years | Colon and rectum cancer | High fasting plasma glucose | Number | 2017 | 5133.404557 | 7949.97781 | 2528.50698 |
| Deaths | Global | Both | 80-84 years | Colon and rectum cancer | High fasting plasma glucose | Number | 2017 | 11063.14752 | 16877.43353 | 5571.627732 |
| Deaths | Global | Male | 80-84 years | Colon and rectum cancer | High fasting plasma glucose | Rate | 2017 | 17.99315998 | 27.57238748 | 9.04505072 |
| Deaths | Global | Female | 80-84 years | Colon and rectum cancer | High fasting plasma glucose | Rate | 2017 | 11.09919155 | 17.1890459 | 5.467011804 |
| Deaths | Global | Both | 80-84 years | Colon and rectum cancer | High fasting plasma glucose | Rate | 2017 | 13.96759842 | 21.30833142 | 7.034368707 |
| Deaths | Global | Male | 80-84 years | Colon and rectum cancer | High fasting plasma glucose | Number | 2018 | 6096.85962 | 9298.123119 | 3081.682586 |
| Deaths | Global | Female | 80-84 years | Colon and rectum cancer | High fasting plasma glucose | Number | 2018 | 5260.117773 | 8105.620659 | 2542.209579 |
| Deaths | Global | Both | 80-84 years | Colon and rectum cancer | High fasting plasma glucose | Number | 2018 | 11356.97739 | 17363.3982 | 5701.000149 |
| Deaths | Global | Male | 80-84 years | Colon and rectum cancer | High fasting plasma glucose | Rate | 2018 | 17.89263784 | 27.28748239 | 9.043906844 |
| Deaths | Global | Female | 80-84 years | Colon and rectum cancer | High fasting plasma glucose | Rate | 2018 | 11.02281079 | 16.98568868 | 5.327313265 |
| Deaths | Global | Both | 80-84 years | Colon and rectum cancer | High fasting plasma glucose | Rate | 2018 | 13.88468796 | 21.22795157 | 6.969865785 |
| Deaths | Global | Male | 80-84 years | Colon and rectum cancer | High fasting plasma glucose | Number | 2019 | 6246.654212 | 9568.05001 | 3204.358313 |
| Deaths | Global | Female | 80-84 years | Colon and rectum cancer | High fasting plasma glucose | Number | 2019 | 5406.013044 | 8353.494045 | 2664.545245 |
| Deaths | Global | Both | 80-84 years | Colon and rectum cancer | High fasting plasma glucose | Number | 2019 | 11652.66726 | 17910.86762 | 5908.237043 |
| Deaths | Global | Male | 80-84 years | Colon and rectum cancer | High fasting plasma glucose | Rate | 2019 | 17.77015395 | 27.21868634 | 9.115590297 |
| Deaths | Global | Female | 80-84 years | Colon and rectum cancer | High fasting plasma glucose | Rate | 2019 | 11.0226518 | 17.03245172 | 5.432904844 |
| Deaths | Global | Both | 80-84 years | Colon and rectum cancer | High fasting plasma glucose | Rate | 2019 | 13.8397518 | 21.27255134 | 7.017151735 |
| Deaths | Global | Male | 80-84 years | Colon and rectum cancer | High fasting plasma glucose | Number | 2020 | 6304.287747 | 9739.282117 | 3205.561155 |
| Deaths | Global | Female | 80-84 years | Colon and rectum cancer | High fasting plasma glucose | Number | 2020 | 5495.957086 | 8406.833805 | 2693.896072 |
| Deaths | Global | Both | 80-84 years | Colon and rectum cancer | High fasting plasma glucose | Number | 2020 | 11800.24483 | 18332.07736 | 5935.167456 |
| Deaths | Global | Male | 80-84 years | Colon and rectum cancer | High fasting plasma glucose | Rate | 2020 | 17.48948979 | 27.01892458 | 8.892936259 |
| Deaths | Global | Female | 80-84 years | Colon and rectum cancer | High fasting plasma glucose | Rate | 2020 | 10.94879898 | 16.7477169 | 5.366658819 |
| Deaths | Global | Both | 80-84 years | Colon and rectum cancer | High fasting plasma glucose | Rate | 2020 | 13.68254665 | 21.25629657 | 6.881908533 |
| Deaths | Global | Male | 80-84 years | Colon and rectum cancer | High fasting plasma glucose | Number | 2021 | 6316.950083 | 9766.562392 | 3272.531488 |
| Deaths | Global | Female | 80-84 years | Colon and rectum cancer | High fasting plasma glucose | Number | 2021 | 5522.11448 | 8534.330134 | 2765.46311 |
| Deaths | Global | Both | 80-84 years | Colon and rectum cancer | High fasting plasma glucose | Number | 2021 | 11839.06456 | 18257.46867 | 6073.429261 |
| Deaths | Global | Male | 80-84 years | Colon and rectum cancer | High fasting plasma glucose | Rate | 2021 | 17.23500445 | 26.64683812 | 8.928690906 |
| Deaths | Global | Female | 80-84 years | Colon and rectum cancer | High fasting plasma glucose | Rate | 2021 | 10.84230306 | 16.75658736 | 5.429802159 |
| Deaths | Global | Both | 80-84 years | Colon and rectum cancer | High fasting plasma glucose | Rate | 2021 | 13.51752837 | 20.84589111 | 6.934479652 |
| Deaths | Global | Male | 85-89 years | Colon and rectum cancer | High fasting plasma glucose | Number | 1990 | 1017.880547 | 1552.935595 | 518.9541132 |
| Deaths | Global | Female | 85-89 years | Colon and rectum cancer | High fasting plasma glucose | Number | 1990 | 1483.714119 | 2295.737752 | 754.6096017 |
| Deaths | Global | Both | 85-89 years | Colon and rectum cancer | High fasting plasma glucose | Number | 1990 | 2501.594666 | 3838.538398 | 1271.61213 |
| Deaths | Global | Male | 85-89 years | Colon and rectum cancer | High fasting plasma glucose | Rate | 1990 | 20.10008589 | 30.66581727 | 10.24778623 |
| Deaths | Global | Female | 85-89 years | Colon and rectum cancer | High fasting plasma glucose | Rate | 1990 | 14.76771547 | 22.84995572 | 7.510786442 |
| Deaths | Global | Both | 85-89 years | Colon and rectum cancer | High fasting plasma glucose | Rate | 1990 | 16.55471288 | 25.40215724 | 8.415101773 |
| Deaths | Global | Male | 85-89 years | Colon and rectum cancer | High fasting plasma glucose | Number | 1991 | 1068.744613 | 1627.398295 | 541.4438965 |
| Deaths | Global | Female | 85-89 years | Colon and rectum cancer | High fasting plasma glucose | Number | 1991 | 1552.700772 | 2387.144607 | 780.8137827 |
| Deaths | Global | Both | 85-89 years | Colon and rectum cancer | High fasting plasma glucose | Number | 1991 | 2621.445385 | 3988.785237 | 1327.564265 |
| Deaths | Global | Male | 85-89 years | Colon and rectum cancer | High fasting plasma glucose | Rate | 1991 | 20.10967847 | 30.62140015 | 10.18789946 |
| Deaths | Global | Female | 85-89 years | Colon and rectum cancer | High fasting plasma glucose | Rate | 1991 | 14.79982947 | 22.75347171 | 7.442458351 |
| Deaths | Global | Both | 85-89 years | Colon and rectum cancer | High fasting plasma glucose | Rate | 1991 | 16.5852116 | 25.23601963 | 8.399158105 |
| Deaths | Global | Male | 85-89 years | Colon and rectum cancer | High fasting plasma glucose | Number | 1992 | 1131.337428 | 1725.587589 | 567.539628 |
| Deaths | Global | Female | 85-89 years | Colon and rectum cancer | High fasting plasma glucose | Number | 1992 | 1640.968132 | 2491.480231 | 823.7405991 |
| Deaths | Global | Both | 85-89 years | Colon and rectum cancer | High fasting plasma glucose | Number | 1992 | 2772.30556 | 4243.929257 | 1404.211216 |
| Deaths | Global | Male | 85-89 years | Colon and rectum cancer | High fasting plasma glucose | Rate | 1992 | 20.27335086 | 30.92220035 | 10.17020185 |
| Deaths | Global | Female | 85-89 years | Colon and rectum cancer | High fasting plasma glucose | Rate | 1992 | 14.93947437 | 22.6825886 | 7.49938486 |
| Deaths | Global | Both | 85-89 years | Colon and rectum cancer | High fasting plasma glucose | Rate | 1992 | 16.73640195 | 25.62059064 | 8.477219705 |
| Deaths | Global | Male | 85-89 years | Colon and rectum cancer | High fasting plasma glucose | Number | 1993 | 1184.229669 | 1810.010269 | 592.479573 |
| Deaths | Global | Female | 85-89 years | Colon and rectum cancer | High fasting plasma glucose | Number | 1993 | 1733.30349 | 2640.201825 | 866.2836972 |
| Deaths | Global | Both | 85-89 years | Colon and rectum cancer | High fasting plasma glucose | Number | 1993 | 2917.533159 | 4452.902408 | 1470.707393 |
| Deaths | Global | Male | 85-89 years | Colon and rectum cancer | High fasting plasma glucose | Rate | 1993 | 20.38594462 | 31.15845691 | 10.19925111 |
| Deaths | Global | Female | 85-89 years | Colon and rectum cancer | High fasting plasma glucose | Rate | 1993 | 15.20362127 | 23.1584537 | 7.598582313 |
| Deaths | Global | Both | 85-89 years | Colon and rectum cancer | High fasting plasma glucose | Rate | 1993 | 16.95289456 | 25.87445656 | 8.545831703 |
| Deaths | Global | Male | 85-89 years | Colon and rectum cancer | High fasting plasma glucose | Number | 1994 | 1239.747301 | 1891.536209 | 627.4084835 |
| Deaths | Global | Female | 85-89 years | Colon and rectum cancer | High fasting plasma glucose | Number | 1994 | 1824.071313 | 2801.287459 | 911.6380528 |
| Deaths | Global | Both | 85-89 years | Colon and rectum cancer | High fasting plasma glucose | Number | 1994 | 3063.818614 | 4709.851946 | 1542.290704 |
| Deaths | Global | Male | 85-89 years | Colon and rectum cancer | High fasting plasma glucose | Rate | 1994 | 20.5796627 | 31.39928365 | 10.41490871 |
| Deaths | Global | Female | 85-89 years | Colon and rectum cancer | High fasting plasma glucose | Rate | 1994 | 15.43750909 | 23.70790018 | 7.715389537 |
| Deaths | Global | Both | 85-89 years | Colon and rectum cancer | High fasting plasma glucose | Rate | 1994 | 17.17389262 | 26.40054839 | 8.645138071 |
| Deaths | Global | Male | 85-89 years | Colon and rectum cancer | High fasting plasma glucose | Number | 1995 | 1311.839076 | 1987.191842 | 661.1816296 |
| Deaths | Global | Female | 85-89 years | Colon and rectum cancer | High fasting plasma glucose | Number | 1995 | 1916.350371 | 2954.968027 | 963.9704184 |
| Deaths | Global | Both | 85-89 years | Colon and rectum cancer | High fasting plasma glucose | Number | 1995 | 3228.189447 | 4980.708959 | 1633.558261 |
| Deaths | Global | Male | 85-89 years | Colon and rectum cancer | High fasting plasma glucose | Rate | 1995 | 21.04902987 | 31.88535943 | 10.60894749 |
| Deaths | Global | Female | 85-89 years | Colon and rectum cancer | High fasting plasma glucose | Rate | 1995 | 15.71965362 | 24.23934294 | 7.907364596 |
| Deaths | Global | Both | 85-89 years | Colon and rectum cancer | High fasting plasma glucose | Rate | 1995 | 17.52251478 | 27.03513774 | 8.866904883 |
| Deaths | Global | Male | 85-89 years | Colon and rectum cancer | High fasting plasma glucose | Number | 1996 | 1385.467937 | 2114.263167 | 702.0808852 |
| Deaths | Global | Female | 85-89 years | Colon and rectum cancer | High fasting plasma glucose | Number | 1996 | 2008.807445 | 3104.213434 | 1011.617033 |
| Deaths | Global | Both | 85-89 years | Colon and rectum cancer | High fasting plasma glucose | Number | 1996 | 3394.275382 | 5203.131095 | 1711.194674 |
| Deaths | Global | Male | 85-89 years | Colon and rectum cancer | High fasting plasma glucose | Rate | 1996 | 21.50636558 | 32.81932074 | 10.89827327 |
| Deaths | Global | Female | 85-89 years | Colon and rectum cancer | High fasting plasma glucose | Rate | 1996 | 15.94085161 | 24.63342408 | 8.027666882 |
| Deaths | Global | Both | 85-89 years | Colon and rectum cancer | High fasting plasma glucose | Rate | 1996 | 17.82355541 | 27.32197153 | 8.98559181 |
| Deaths | Global | Male | 85-89 years | Colon and rectum cancer | High fasting plasma glucose | Number | 1997 | 1461.639759 | 2216.084532 | 734.9002739 |
| Deaths | Global | Female | 85-89 years | Colon and rectum cancer | High fasting plasma glucose | Number | 1997 | 2092.672336 | 3209.313385 | 1029.724845 |
| Deaths | Global | Both | 85-89 years | Colon and rectum cancer | High fasting plasma glucose | Number | 1997 | 3554.312095 | 5403.17283 | 1789.171242 |
| Deaths | Global | Male | 85-89 years | Colon and rectum cancer | High fasting plasma glucose | Rate | 1997 | 21.95871179 | 33.2929925 | 11.04065705 |
| Deaths | Global | Female | 85-89 years | Colon and rectum cancer | High fasting plasma glucose | Rate | 1997 | 16.10213382 | 24.69416387 | 7.923250554 |
| Deaths | Global | Both | 85-89 years | Colon and rectum cancer | High fasting plasma glucose | Rate | 1997 | 18.0857538 | 27.49349267 | 9.104014989 |
| Deaths | Global | Male | 85-89 years | Colon and rectum cancer | High fasting plasma glucose | Number | 1998 | 1559.094053 | 2366.226422 | 788.1969856 |
| Deaths | Global | Female | 85-89 years | Colon and rectum cancer | High fasting plasma glucose | Number | 1998 | 2210.624685 | 3384.479572 | 1084.499773 |
| Deaths | Global | Both | 85-89 years | Colon and rectum cancer | High fasting plasma glucose | Number | 1998 | 3769.718738 | 5753.048892 | 1892.371759 |
| Deaths | Global | Male | 85-89 years | Colon and rectum cancer | High fasting plasma glucose | Rate | 1998 | 22.66879322 | 34.40427302 | 11.4601646 |
| Deaths | Global | Female | 85-89 years | Colon and rectum cancer | High fasting plasma glucose | Rate | 1998 | 16.44556461 | 25.17825746 | 8.067950749 |
| Deaths | Global | Both | 85-89 years | Colon and rectum cancer | High fasting plasma glucose | Rate | 1998 | 18.55196342 | 28.31255061 | 9.312952527 |
| Deaths | Global | Male | 85-89 years | Colon and rectum cancer | High fasting plasma glucose | Number | 1999 | 1655.282487 | 2508.409938 | 832.5771758 |
| Deaths | Global | Female | 85-89 years | Colon and rectum cancer | High fasting plasma glucose | Number | 1999 | 2325.610825 | 3555.555173 | 1139.562437 |
| Deaths | Global | Both | 85-89 years | Colon and rectum cancer | High fasting plasma glucose | Number | 1999 | 3980.893311 | 6060.964508 | 1997.923944 |
| Deaths | Global | Male | 85-89 years | Colon and rectum cancer | High fasting plasma glucose | Rate | 1999 | 23.34910707 | 35.38316433 | 11.74417888 |
| Deaths | Global | Female | 85-89 years | Colon and rectum cancer | High fasting plasma glucose | Rate | 1999 | 16.82134465 | 25.71763872 | 8.242553867 |
| Deaths | Global | Both | 85-89 years | Colon and rectum cancer | High fasting plasma glucose | Rate | 1999 | 19.03401117 | 28.97954231 | 9.552757055 |
| Deaths | Global | Male | 85-89 years | Colon and rectum cancer | High fasting plasma glucose | Number | 2000 | 1746.299141 | 2625.228709 | 873.8258256 |
| Deaths | Global | Female | 85-89 years | Colon and rectum cancer | High fasting plasma glucose | Number | 2000 | 2412.16075 | 3687.178372 | 1186.120504 |
| Deaths | Global | Both | 85-89 years | Colon and rectum cancer | High fasting plasma glucose | Number | 2000 | 4158.45989 | 6331.167891 | 2078.860715 |
| Deaths | Global | Male | 85-89 years | Colon and rectum cancer | High fasting plasma glucose | Rate | 2000 | 24.02091624 | 36.11088013 | 12.01976023 |
| Deaths | Global | Female | 85-89 years | Colon and rectum cancer | High fasting plasma glucose | Rate | 2000 | 17.07879083 | 26.10628176 | 8.398073797 |
| Deaths | Global | Both | 85-89 years | Colon and rectum cancer | High fasting plasma glucose | Rate | 2000 | 19.43784012 | 29.59370356 | 9.717194172 |
| Deaths | Global | Male | 85-89 years | Colon and rectum cancer | High fasting plasma glucose | Number | 2001 | 1812.85121 | 2733.989361 | 903.6959701 |
| Deaths | Global | Female | 85-89 years | Colon and rectum cancer | High fasting plasma glucose | Number | 2001 | 2465.318424 | 3771.451174 | 1206.058988 |
| Deaths | Global | Both | 85-89 years | Colon and rectum cancer | High fasting plasma glucose | Number | 2001 | 4278.169633 | 6514.426751 | 2135.953988 |
| Deaths | Global | Male | 85-89 years | Colon and rectum cancer | High fasting plasma glucose | Rate | 2001 | 24.49221703 | 36.93709691 | 12.20923025 |
| Deaths | Global | Female | 85-89 years | Colon and rectum cancer | High fasting plasma glucose | Rate | 2001 | 17.31175474 | 26.48357191 | 8.469087481 |
| Deaths | Global | Both | 85-89 years | Colon and rectum cancer | High fasting plasma glucose | Rate | 2001 | 19.76747998 | 30.10020907 | 9.869273855 |
| Deaths | Global | Male | 85-89 years | Colon and rectum cancer | High fasting plasma glucose | Number | 2002 | 1833.83469 | 2775.541906 | 923.7638285 |
| Deaths | Global | Female | 85-89 years | Colon and rectum cancer | High fasting plasma glucose | Number | 2002 | 2471.99501 | 3805.234485 | 1204.888181 |
| Deaths | Global | Both | 85-89 years | Colon and rectum cancer | High fasting plasma glucose | Number | 2002 | 4305.829699 | 6603.416167 | 2148.01093 |
| Deaths | Global | Male | 85-89 years | Colon and rectum cancer | High fasting plasma glucose | Rate | 2002 | 24.39130756 | 36.91668428 | 12.28671689 |
| Deaths | Global | Female | 85-89 years | Colon and rectum cancer | High fasting plasma glucose | Rate | 2002 | 17.29507802 | 26.62296123 | 8.429885582 |
| Deaths | Global | Both | 85-89 years | Colon and rectum cancer | High fasting plasma glucose | Rate | 2002 | 19.74114436 | 30.27499946 | 9.848088944 |
| Deaths | Global | Male | 85-89 years | Colon and rectum cancer | High fasting plasma glucose | Number | 2003 | 1871.448609 | 2813.800574 | 945.194754 |
| Deaths | Global | Female | 85-89 years | Colon and rectum cancer | High fasting plasma glucose | Number | 2003 | 2475.364899 | 3750.950847 | 1205.728218 |
| Deaths | Global | Both | 85-89 years | Colon and rectum cancer | High fasting plasma glucose | Number | 2003 | 4346.813509 | 6608.325049 | 2163.544531 |
| Deaths | Global | Male | 85-89 years | Colon and rectum cancer | High fasting plasma glucose | Rate | 2003 | 24.49050849 | 36.82249489 | 12.36918825 |
| Deaths | Global | Female | 85-89 years | Colon and rectum cancer | High fasting plasma glucose | Rate | 2003 | 17.25981076 | 26.15400333 | 8.407100258 |
| Deaths | Global | Both | 85-89 years | Colon and rectum cancer | High fasting plasma glucose | Rate | 2003 | 19.77324316 | 30.06064507 | 9.841759262 |
| Deaths | Global | Male | 85-89 years | Colon and rectum cancer | High fasting plasma glucose | Number | 2004 | 1892.758448 | 2849.441004 | 947.5422705 |
| Deaths | Global | Female | 85-89 years | Colon and rectum cancer | High fasting plasma glucose | Number | 2004 | 2476.768941 | 3799.061294 | 1202.29242 |
| Deaths | Global | Both | 85-89 years | Colon and rectum cancer | High fasting plasma glucose | Number | 2004 | 4369.527389 | 6655.124464 | 2156.452479 |
| Deaths | Global | Male | 85-89 years | Colon and rectum cancer | High fasting plasma glucose | Rate | 2004 | 24.18442396 | 36.40828515 | 12.10707262 |
| Deaths | Global | Female | 85-89 years | Colon and rectum cancer | High fasting plasma glucose | Rate | 2004 | 16.95576586 | 26.00807557 | 8.230799586 |
| Deaths | Global | Both | 85-89 years | Colon and rectum cancer | High fasting plasma glucose | Rate | 2004 | 19.47761059 | 29.66589087 | 9.612605182 |
| Deaths | Global | Male | 85-89 years | Colon and rectum cancer | High fasting plasma glucose | Number | 2005 | 1980.653548 | 2973.578427 | 986.8041554 |
| Deaths | Global | Female | 85-89 years | Colon and rectum cancer | High fasting plasma glucose | Number | 2005 | 2552.409532 | 3923.635504 | 1240.958943 |
| Deaths | Global | Both | 85-89 years | Colon and rectum cancer | High fasting plasma glucose | Number | 2005 | 4533.063079 | 6908.981933 | 2236.995259 |
| Deaths | Global | Male | 85-89 years | Colon and rectum cancer | High fasting plasma glucose | Rate | 2005 | 24.18764445 | 36.31319461 | 12.05080418 |
[truncated: 1,604,580 more chars]
