# Supplementary material for: Global, regional, and national burden of hyperglycemia-associated colorectal cancer, 1990-2021: a systemic analysis for the Global Burden of Disease study
Source: Front Oncol. 2025 Sep 25;15:1633508. doi: 10.3389/fonc.2025.1633508 (PMC12507591; doi:10.3389/fonc.2025.1633508)
Supplement: Supplementary file 1 [file DataSheet1.zip › Table S3.docx]

**Table S3.** The EAPC of hyperglycemia-associated colorectal cancer-related ASRs of deaths, YLDs, YLLs and DALYs for different SDI regions between 1990 and 2021. Abbreviations: EAPC, estimated annual percentage change; ASR, age-standardized rate; YLDs, Years Lived with Disability; YLLs, Years of Life Lost; DALYs, disability-adjusted-life-years.

|  | **location** | **measure** | **sex** | **cause** | **age** | **EAPC** | **LCI** | **UCI** | **EAPC_95CI** |
| --- | --- | --- | --- | --- | --- | --- | --- | --- | --- |
| 1 | High SDI | Deaths | Both | Colon and rectum cancer | Age-standardized | -0.079477981 | -0.181863428 | 0.023012484 | -0.08(-0.18,0.02) |
| 2 | High SDI | DALYs | Both | Colon and rectum cancer | Age-standardized | -0.084662465 | -0.182258024 | 0.013028517 | -0.08(-0.18,0.01) |
| 3 | High SDI | YLDs | Both | Colon and rectum cancer | Age-standardized | 1.120678135 | 0.932820979 | 1.308884933 | 1.12(0.93,1.31) |
| 4 | High SDI | YLLs | Both | Colon and rectum cancer | Age-standardized | -0.156959068 | -0.250993698 | -0.062835791 | -0.16(-0.25,-0.06) |
| 5 | Middle SDI | Deaths | Both | Colon and rectum cancer | Age-standardized | 0.843890397 | 0.787185983 | 0.900626715 | 0.84(0.79,0.9) |
| 6 | Middle SDI | DALYs | Both | Colon and rectum cancer | Age-standardized | 0.825301634 | 0.7765062 | 0.874120694 | 0.83(0.78,0.87) |
| 7 | Middle SDI | YLDs | Both | Colon and rectum cancer | Age-standardized | 2.868912621 | 2.782858256 | 2.955039034 | 2.87(2.78,2.96) |
| 8 | Middle SDI | YLLs | Both | Colon and rectum cancer | Age-standardized | 0.769637799 | 0.721241362 | 0.81805749 | 0.77(0.72,0.82) |
| 9 | Low SDI | Deaths | Both | Colon and rectum cancer | Age-standardized | 0.843999221 | 0.734233981 | 0.953884068 | 0.84(0.73,0.95) |
| 10 | Low SDI | DALYs | Both | Colon and rectum cancer | Age-standardized | 0.599382979 | 0.485897299 | 0.712996827 | 0.6 (0.49,0.71) |
| 11 | Low SDI | YLDs | Both | Colon and rectum cancer | Age-standardized | 1.114689202 | 0.985065693 | 1.244479096 | 1.11 (0.99,1.24) |
| 12 | Low SDI | YLLs | Both | Colon and rectum cancer | Age-standardized | 0.590889695 | 0.477691366 | 0.704215553 | 0.59 (0.48,0.7) |
| 13 | High-middle SDI | Deaths | Both | Colon and rectum cancer | Age-standardized | 0.700939934 | 0.551699587 | 0.850401786 | 0.7 (0.55,0.85) |
| 14 | High-middle SDI | DALYs | Both | Colon and rectum cancer | Age-standardized | 0.630242305 | 0.5081362 | 0.752496756 | 0.63 (0.51,0.75) |
| 15 | High-middle SDI | YLDs | Both | Colon and rectum cancer | Age-standardized | 2.61695896 | 2.469492799 | 2.764637342 | 2.62 (2.47,2.76) |
| 16 | High-middle SDI | YLLs | Both | Colon and rectum cancer | Age-standardized | 0.55558659 | 0.432130247 | 0.679194693 | 0.56 (0.43,0.68) |
| 17 | Low-middle SDI | Deaths | Both | Colon and rectum cancer | Age-standardized | 1.860300193 | 1.821308138 | 1.89930718 | 1.86 (1.82,1.9) |
| 18 | Low-middle SDI | DALYs | Both | Colon and rectum cancer | Age-standardized | 1.791311218 | 1.760518516 | 1.822113237 | 1.79 (1.76,1.82) |
| 19 | Low-middle SDI | YLDs | Both | Colon and rectum cancer | Age-standardized | 2.56549181 | 2.52412104 | 2.606879275 | 2.57 (2.52,2.61) |
| 20 | Low-middle SDI | YLLs | Both | Colon and rectum cancer | Age-standardized | 1.776744407 | 1.745729664 | 1.807768604 | 1.78 (1.75,1.81) |
