# Supplementary material for: Global, regional, and national burden of hyperglycemia-associated colorectal cancer, 1990-2021: a systemic analysis for the Global Burden of Disease study
Source: Front Oncol. 2025 Sep 25;15:1633508. doi: 10.3389/fonc.2025.1633508 (PMC12507591; doi:10.3389/fonc.2025.1633508)
Supplement: Supplementary file 1 [file DataSheet1.zip › Table S4.docx]

**Table S4.** The EAPC of hyperglycemia-associated colorectal cancer-related ASRs of deaths, YLDs, YLLs and DALYs for different GBD regions between 1990 and 2021. Abbreviations: EAPC, estimated annual percentage change; ASR, age-standardized rate; YLDs, Years Lived with Disability; YLLs, Years of Life Lost; DALYs, disability-adjusted-life-years.

|  | **location** | **measure** | **sex** | **cause** | **age** | **EAPC** | **LCI** | **UCI** | **EAPC_95CI** |
| --- | --- | --- | --- | --- | --- | --- | --- | --- | --- |
| 1 | Central Asia | Deaths | Both | Colon and rectum cancer | Age-standardized | 1.956846824 | 1.726560498 | 2.187654467 | 1.96(1.73,2.19) |
| 2 | Central Asia | DALYs | Both | Colon and rectum cancer | Age-standardized | 1.565019385 | 1.382787492 | 1.747578833 | 1.57(1.38,1.75) |
| 3 | Central Asia | YLDs | Both | Colon and rectum cancer | Age-standardized | 2.379769797 | 2.129108739 | 2.631046066 | 2.38(2.13,2.63) |
| 4 | Central Asia | YLLs | Both | Colon and rectum cancer | Age-standardized | 1.546888975 | 1.365839028 | 1.728262297 | 1.55(1.37,1.73) |
| 5 | East Asia | Deaths | Both | Colon and rectum cancer | Age-standardized | 0.360964931 | 0.216603307 | 0.505534506 | 0.36(0.22,0.51) |
| 6 | East Asia | DALYs | Both | Colon and rectum cancer | Age-standardized | 0.357865718 | 0.253336375 | 0.46250405 | 0.36(0.25,0.46) |
| 7 | East Asia | YLDs | Both | Colon and rectum cancer | Age-standardized | 3.352604444 | 3.228720097 | 3.476637465 | 3.35(3.23,3.48) |
| 8 | East Asia | YLLs | Both | Colon and rectum cancer | Age-standardized | 0.258499897 | 0.151333002 | 0.365781466 | 0.26(0.15,0.37) |
| 9 | Oceania | Deaths | Both | Colon and rectum cancer | Age-standardized | 0.289613817 | 0.213051738 | 0.366234388 | 0.29(0.21,0.37) |
| 10 | Oceania | DALYs | Both | Colon and rectum cancer | Age-standardized | 0.298517946 | 0.247618831 | 0.349442904 | 0.3(0.25,0.35) |
| 11 | Oceania | YLDs | Both | Colon and rectum cancer | Age-standardized | 0.604944766 | 0.549422089 | 0.660498102 | 0.6(0.55,0.66) |
| 12 | Oceania | YLLs | Both | Colon and rectum cancer | Age-standardized | 0.292686016 | 0.241845373 | 0.343552444 | 0.29(0.24,0.34) |
| 13 | Southeast Asia | Deaths | Both | Colon and rectum cancer | Age-standardized | 1.881849787 | 1.802123371 | 1.961638641 | 1.88(1.8,1.96) |
| 14 | Southeast Asia | DALYs | Both | Colon and rectum cancer | Age-standardized | 1.811090386 | 1.753443158 | 1.868770273 | 1.81(1.75,1.87) |
| 15 | Southeast Asia | YLDs | Both | Colon and rectum cancer | Age-standardized | 2.952421747 | 2.899922879 | 3.004947399 | 2.95(2.9,3) |
| 16 | Southeast Asia | YLLs | Both | Colon and rectum cancer | Age-standardized | 1.786464616 | 1.728334999 | 1.844627449 | 1.79(1.73,1.84) |
| 17 | Central Europe | Deaths | Both | Colon and rectum cancer | Age-standardized | 1.288066917 | 1.125089839 | 1.451306655 | 1.29(1.13,1.45) |
| 18 | Central Europe | DALYs | Both | Colon and rectum cancer | Age-standardized | 1.260307513 | 1.096402148 | 1.424478614 | 1.26(1.1,1.42) |
| 19 | Central Europe | YLDs | Both | Colon and rectum cancer | Age-standardized | 2.787881112 | 2.568141642 | 3.008091346 | 2.79(2.57,3.01) |
| 20 | Central Europe | YLLs | Both | Colon and rectum cancer | Age-standardized | 1.215625 | 1.052346672 | 1.379167149 | 1.22(1.05,1.38) |
| 21 | Eastern Europe | Deaths | Both | Colon and rectum cancer | Age-standardized | 1.490464036 | 1.387244683 | 1.593788474 | 1.49(1.39,1.59) |
| 22 | Eastern Europe | DALYs | Both | Colon and rectum cancer | Age-standardized | 1.184771332 | 1.071520398 | 1.298149164 | 1.18(1.07,1.3) |
| 23 | Eastern Europe | YLDs | Both | Colon and rectum cancer | Age-standardized | 2.51097269 | 2.357642981 | 2.664532085 | 2.51(2.36,2.66) |
| 24 | Eastern Europe | YLLs | Both | Colon and rectum cancer | Age-standardized | 1.146055595 | 1.029382863 | 1.262863066 | 1.15(1.03,1.26) |
| 25 | Australasia | Deaths | Both | Colon and rectum cancer | Age-standardized | -0.732521729 | -0.796432242 | -0.668570042 | -0.73(-0.8,-0.67) |
| 26 | Australasia | DALYs | Both | Colon and rectum cancer | Age-standardized | -0.86778467 | -0.93807157 | -0.797447899 | -0.87(-0.94,-0.8) |
| 27 | Australasia | YLDs | Both | Colon and rectum cancer | Age-standardized | 0.978407902 | 0.767088727 | 1.190170237 | 0.98(0.77,1.19) |
| 28 | Australasia | YLLs | Both | Colon and rectum cancer | Age-standardized | -0.988021689 | -1.060625564 | -0.915364536 | -0.99(-1.06,-0.92) |
| 29 | High-income Asia Pacific | Deaths | Both | Colon and rectum cancer | Age-standardized | -0.46438744 | -0.559666436 | -0.369017152 | -0.46(-0.56,-0.37) |
| 30 | High-income Asia Pacific | DALYs | Both | Colon and rectum cancer | Age-standardized | -0.643825993 | -0.752737504 | -0.534794966 | -0.64(-0.75,-0.53) |
| 31 | High-income Asia Pacific | YLDs | Both | Colon and rectum cancer | Age-standardized | 0.717516678 | 0.56522067 | 0.870043323 | 0.72(0.57,0.87) |
| 32 | High-income Asia Pacific | YLLs | Both | Colon and rectum cancer | Age-standardized | -0.739466192 | -0.847815415 | -0.630998569 | -0.74(-0.85,-0.63) |
| 33 | Southern Latin America | Deaths | Both | Colon and rectum cancer | Age-standardized | 1.354290588 | 1.146688943 | 1.562318331 | 1.35(1.15,1.56) |
| 34 | Southern Latin America | DALYs | Both | Colon and rectum cancer | Age-standardized | 1.384280944 | 1.192521593 | 1.576403678 | 1.38(1.19,1.58) |
| 35 | Southern Latin America | YLDs | Both | Colon and rectum cancer | Age-standardized | 2.604512473 | 2.406219966 | 2.80318894 | 2.6(2.41,2.8) |
| 36 | Southern Latin America | YLLs | Both | Colon and rectum cancer | Age-standardized | 1.35073933 | 1.158561305 | 1.543282448 | 1.35(1.16,1.54) |
| 37 | High-income North America | Deaths | Both | Colon and rectum cancer | Age-standardized | -0.012622928 | -0.181596256 | 0.156636439 | -0.01(-0.18,0.16) |
| 38 | High-income North America | DALYs | Both | Colon and rectum cancer | Age-standardized | 0.080088294 | -0.060749404 | 0.221124465 | 0.08(-0.06,0.22) |
| 39 | High-income North America | YLDs | Both | Colon and rectum cancer | Age-standardized | 0.869779328 | 0.628836534 | 1.111299028 | 0.87(0.63,1.11) |
| 40 | High-income North America | YLLs | Both | Colon and rectum cancer | Age-standardized | 0.027382444 | -0.107854029 | 0.162802004 | 0.03(-0.11,0.16) |
| 41 | Western Europe | Deaths | Both | Colon and rectum cancer | Age-standardized | -0.278155381 | -0.376733014 | -0.179480206 | -0.28(-0.38,-0.18) |
| 42 | Western Europe | DALYs | Both | Colon and rectum cancer | Age-standardized | -0.291610127 | -0.402548375 | -0.180548308 | -0.29(-0.4,-0.18) |
| 43 | Western Europe | YLDs | Both | Colon and rectum cancer | Age-standardized | 1.383365232 | 1.150048051 | 1.617220592 | 1.38(1.15,1.62) |
| 44 | Western Europe | YLLs | Both | Colon and rectum cancer | Age-standardized | -0.389789549 | -0.496567299 | -0.282897216 | -0.39(-0.5,-0.28) |
| 45 | Caribbean | Deaths | Both | Colon and rectum cancer | Age-standardized | 0.972767242 | 0.929341637 | 1.016211532 | 0.97(0.93,1.02) |
| 46 | Caribbean | DALYs | Both | Colon and rectum cancer | Age-standardized | 1.121962525 | 1.077289507 | 1.166655288 | 1.12(1.08,1.17) |
| 47 | Caribbean | YLDs | Both | Colon and rectum cancer | Age-standardized | 2.502443341 | 2.394309996 | 2.610690879 | 2.5(2.39,2.61) |
| 48 | Caribbean | YLLs | Both | Colon and rectum cancer | Age-standardized | 1.062728753 | 1.017779402 | 1.107698104 | 1.06(1.02,1.11) |
| 49 | Andean Latin America | Deaths | Both | Colon and rectum cancer | Age-standardized | 2.422102089 | 2.256844756 | 2.587626495 | 2.42(2.26,2.59) |
| 50 | Andean Latin America | DALYs | Both | Colon and rectum cancer | Age-standardized | 2.333053442 | 2.19243176 | 2.473868627 | 2.33(2.19,2.47) |
| 51 | Andean Latin America | YLDs | Both | Colon and rectum cancer | Age-standardized | 3.878214196 | 3.737647609 | 4.018971254 | 3.88(3.74,4.02) |
| 52 | Andean Latin America | YLLs | Both | Colon and rectum cancer | Age-standardized | 2.294997156 | 2.153737455 | 2.436452194 | 2.29(2.15,2.44) |
| 53 | Central Latin America | Deaths | Both | Colon and rectum cancer | Age-standardized | 1.319568606 | 1.232412139 | 1.406800112 | 1.32(1.23,1.41) |
| 54 | Central Latin America | DALYs | Both | Colon and rectum cancer | Age-standardized | 1.612528408 | 1.531568533 | 1.693552839 | 1.61(1.53,1.69) |
| 55 | Central Latin America | YLDs | Both | Colon and rectum cancer | Age-standardized | 2.939803143 | 2.866484379 | 3.013174165 | 2.94(2.87,3.01) |
| 56 | Central Latin America | YLLs | Both | Colon and rectum cancer | Age-standardized | 1.574392024 | 1.492591577 | 1.6562584 | 1.57(1.49,1.66) |
| 57 | Tropical Latin America | Deaths | Both | Colon and rectum cancer | Age-standardized | 1.656647699 | 1.560927487 | 1.752458127 | 1.66(1.56,1.75) |
| 58 | Tropical Latin America | DALYs | Both | Colon and rectum cancer | Age-standardized | 1.839704511 | 1.743403633 | 1.936096538 | 1.84(1.74,1.94) |
| 59 | Tropical Latin America | YLDs | Both | Colon and rectum cancer | Age-standardized | 2.819261965 | 2.720188396 | 2.918431089 | 2.82(2.72,2.92) |
| 60 | Tropical Latin America | YLLs | Both | Colon and rectum cancer | Age-standardized | 1.817634468 | 1.721041208 | 1.914319452 | 1.82(1.72,1.91) |
| 61 | North Africa and Middle East | Deaths | Both | Colon and rectum cancer | Age-standardized | 2.129764978 | 1.951597768 | 2.308243547 | 2.13(1.95,2.31) |
| 62 | North Africa and Middle East | DALYs | Both | Colon and rectum cancer | Age-standardized | 2.00288021 | 1.839952811 | 2.166068266 | 2(1.84,2.17) |
| 63 | North Africa and Middle East | YLDs | Both | Colon and rectum cancer | Age-standardized | 3.715916232 | 3.536263521 | 3.895880671 | 3.72(3.54,3.9) |
| 64 | North Africa and Middle East | YLLs | Both | Colon and rectum cancer | Age-standardized | 1.955341166 | 1.79351775 | 2.117421836 | 1.96(1.79,2.12) |
| 65 | South Asia | Deaths | Both | Colon and rectum cancer | Age-standardized | 1.176349652 | 1.070325038 | 1.282485487 | 1.18(1.07,1.28) |
| 66 | South Asia | DALYs | Both | Colon and rectum cancer | Age-standardized | 1.098747409 | 0.988087173 | 1.209528904 | 1.1(0.99,1.21) |
| 67 | South Asia | YLDs | Both | Colon and rectum cancer | Age-standardized | 1.807230775 | 1.67696939 | 1.937659042 | 1.81(1.68,1.94) |
| 68 | South Asia | YLLs | Both | Colon and rectum cancer | Age-standardized | 1.086185108 | 0.975773821 | 1.196717124 | 1.09(0.98,1.2) |
| 69 | Eastern Sub-Saharan Africa | Deaths | Both | Colon and rectum cancer | Age-standardized | 0.753137664 | 0.62383077 | 0.882610723 | 0.75(0.62,0.88) |
| 70 | Eastern Sub-Saharan Africa | DALYs | Both | Colon and rectum cancer | Age-standardized | 0.410389742 | 0.257635646 | 0.563376578 | 0.41(0.26,0.56) |
| 71 | Eastern Sub-Saharan Africa | YLDs | Both | Colon and rectum cancer | Age-standardized | 0.959958163 | 0.793371593 | 1.126820059 | 0.96(0.79,1.13) |
| 72 | Eastern Sub-Saharan Africa | YLLs | Both | Colon and rectum cancer | Age-standardized | 0.401540767 | 0.249056877 | 0.554256592 | 0.4(0.25,0.55) |
| 73 | Central Sub-Saharan Africa | Deaths | Both | Colon and rectum cancer | Age-standardized | 0.627682076 | 0.443032525 | 0.812671077 | 0.63(0.44,0.81) |
| 74 | Central Sub-Saharan Africa | DALYs | Both | Colon and rectum cancer | Age-standardized | 0.622387375 | 0.437069174 | 0.808047511 | 0.62(0.44,0.81) |
| 75 | Central Sub-Saharan Africa | YLDs | Both | Colon and rectum cancer | Age-standardized | 0.949212278 | 0.722457673 | 1.176477371 | 0.95(0.72,1.18) |
| 76 | Central Sub-Saharan Africa | YLLs | Both | Colon and rectum cancer | Age-standardized | 0.617170408 | 0.432525159 | 0.802155127 | 0.62(0.43,0.8) |
| 77 | Western Sub-Saharan Africa | Deaths | Both | Colon and rectum cancer | Age-standardized | 2.101430261 | 1.995351981 | 2.207618865 | 2.1(2,2.21) |
| 78 | Western Sub-Saharan Africa | DALYs | Both | Colon and rectum cancer | Age-standardized | 2.004860105 | 1.912154829 | 2.097649711 | 2(1.91,2.1) |
| 79 | Western Sub-Saharan Africa | YLDs | Both | Colon and rectum cancer | Age-standardized | 2.333191106 | 2.232671478 | 2.433809568 | 2.33(2.23,2.43) |
| 80 | Western Sub-Saharan Africa | YLLs | Both | Colon and rectum cancer | Age-standardized | 1.999249132 | 1.906541591 | 2.092041012 | 2(1.91,2.09) |
| 81 | Southern Sub-Saharan Africa | Deaths | Both | Colon and rectum cancer | Age-standardized | 2.262819449 | 1.962519343 | 2.564003999 | 2.26(1.96,2.56) |
| 82 | Southern Sub-Saharan Africa | DALYs | Both | Colon and rectum cancer | Age-standardized | 2.606413583 | 2.281059234 | 2.932802878 | 2.61(2.28,2.93) |
| 83 | Southern Sub-Saharan Africa | YLDs | Both | Colon and rectum cancer | Age-standardized | 2.816851981 | 2.598922784 | 3.035244079 | 2.82(2.6,3.04) |
| 84 | Southern Sub-Saharan Africa | YLLs | Both | Colon and rectum cancer | Age-standardized | 2.60242755 | 2.274996283 | 2.930907082 | 2.6(2.27,2.93) |
