# Supplementary material for: Global, regional, and national burden of hyperglycemia-associated colorectal cancer, 1990-2021: a systemic analysis for the Global Burden of Disease study
Source: Front Oncol. 2025 Sep 25;15:1633508. doi: 10.3389/fonc.2025.1633508 (PMC12507591; doi:10.3389/fonc.2025.1633508)
Supplement: Supplementary file 1 [file DataSheet1.zip › Table S5.docx]

**Table S5.** The EAPC of hyperglycemia-associated colorectal cancer-related ASRs of deaths, YLDs, YLLs and DALYs for different countries between 1990 and 2021. Abbreviations: EAPC, estimated annual percentage change; ASR, age-standardized rate; YLDs, Years Lived with Disability; YLLs, Years of Life Lost; DALYs, disability-adjusted-life-years.

|  | **location** | **measure** | **sex** | **cause** | **age** | **EAPC** | **LCI** | **UCI** | **EAPC_95CI** |
| --- | --- | --- | --- | --- | --- | --- | --- | --- | --- |
| 1 | Taiwan (Province of China) | Deaths | Both | Colon and rectum cancer | Age-standardized | 1.597980004 | 1.232810566 | 1.964466691 | 1.6 (1.23 to 1.96) |
| 2 | Taiwan (Province of China) | DALYs | Both | Colon and rectum cancer | Age-standardized | 1.279007298 | 0.965986443 | 1.592998599 | 1.28 (0.97 to 1.59) |
| 3 | Taiwan (Province of China) | YLDs | Both | Colon and rectum cancer | Age-standardized | 3.12335672 | 2.620831009 | 3.628343258 | 3.12 (2.62 to 3.63) |
| 4 | Taiwan (Province of China) | YLLs | Both | Colon and rectum cancer | Age-standardized | 1.197676737 | 0.890320394 | 1.505969424 | 1.2 (0.89 to 1.51) |
| 5 | China | Deaths | Both | Colon and rectum cancer | Age-standardized | 0.294290283 | 0.155191151 | 0.433582601 | 0.29 (0.16 to 0.43) |
| 6 | China | DALYs | Both | Colon and rectum cancer | Age-standardized | 0.314150399 | 0.210152268 | 0.418256459 | 0.31 (0.21 to 0.42) |
| 7 | China | YLDs | Both | Colon and rectum cancer | Age-standardized | 3.370021437 | 3.241303892 | 3.498899463 | 3.37 (3.24 to 3.5) |
| 8 | China | YLLs | Both | Colon and rectum cancer | Age-standardized | 0.213410581 | 0.106943821 | 0.319990571 | 0.21 (0.11 to 0.32) |
| 9 | Cambodia | Deaths | Both | Colon and rectum cancer | Age-standardized | 2.401038797 | 2.277210405 | 2.525017109 | 2.4 (2.28 to 2.53) |
| 10 | Cambodia | DALYs | Both | Colon and rectum cancer | Age-standardized | 2.233426091 | 2.153152033 | 2.313763229 | 2.23 (2.15 to 2.31) |
| 11 | Cambodia | YLDs | Both | Colon and rectum cancer | Age-standardized | 3.081527227 | 3.016501107 | 3.146594394 | 3.08 (3.02 to 3.15) |
| 12 | Cambodia | YLLs | Both | Colon and rectum cancer | Age-standardized | 2.218414405 | 2.137689288 | 2.299203324 | 2.22 (2.14 to 2.3) |
| 13 | Democratic People's Republic of Korea | Deaths | Both | Colon and rectum cancer | Age-standardized | 0.624952577 | 0.52767273 | 0.722326561 | 0.62 (0.53 to 0.72) |
| 14 | Democratic People's Republic of Korea | DALYs | Both | Colon and rectum cancer | Age-standardized | 0.681893478 | 0.593400381 | 0.770464424 | 0.68 (0.59 to 0.77) |
| 15 | Democratic People's Republic of Korea | YLDs | Both | Colon and rectum cancer | Age-standardized | 1.615410456 | 1.50806155 | 1.722872887 | 1.62 (1.51 to 1.72) |
| 16 | Democratic People's Republic of Korea | YLLs | Both | Colon and rectum cancer | Age-standardized | 0.661261539 | 0.572433971 | 0.750167561 | 0.66 (0.57 to 0.75) |
| 17 | Indonesia | Deaths | Both | Colon and rectum cancer | Age-standardized | 2.71007085 | 2.596883726 | 2.823382845 | 2.71 (2.6 to 2.82) |
| 18 | Indonesia | DALYs | Both | Colon and rectum cancer | Age-standardized | 2.420802727 | 2.345612409 | 2.496048285 | 2.42 (2.35 to 2.5) |
| 19 | Indonesia | YLDs | Both | Colon and rectum cancer | Age-standardized | 3.052487639 | 2.971426528 | 3.133612562 | 3.05 (2.97 to 3.13) |
| 20 | Indonesia | YLLs | Both | Colon and rectum cancer | Age-standardized | 2.408740538 | 2.333412286 | 2.484124239 | 2.41 (2.33 to 2.48) |
| 21 | Lao People's Democratic Republic | Deaths | Both | Colon and rectum cancer | Age-standardized | 0.629729899 | 0.559470297 | 0.70003859 | 0.63 (0.56 to 0.7) |
| 22 | Lao People's Democratic Republic | DALYs | Both | Colon and rectum cancer | Age-standardized | 0.546913572 | 0.475339136 | 0.618538995 | 0.55 (0.48 to 0.62) |
| 23 | Lao People's Democratic Republic | YLDs | Both | Colon and rectum cancer | Age-standardized | 1.213701142 | 1.172618206 | 1.254800761 | 1.21 (1.17 to 1.25) |
| 24 | Lao People's Democratic Republic | YLLs | Both | Colon and rectum cancer | Age-standardized | 0.535861895 | 0.46366856 | 0.608107108 | 0.54 (0.46 to 0.61) |
| 25 | Maldives | Deaths | Both | Colon and rectum cancer | Age-standardized | -0.854265128 | -0.973066188 | -0.735321544 | -0.85 (-0.97 to -0.74) |
| 26 | Maldives | DALYs | Both | Colon and rectum cancer | Age-standardized | -1.297375756 | -1.4330025 | -1.161562392 | -1.3 (-1.43 to -1.16) |
| 27 | Maldives | YLDs | Both | Colon and rectum cancer | Age-standardized | 0.861929743 | 0.755365404 | 0.96860679 | 0.86 (0.76 to 0.97) |
| 28 | Maldives | YLLs | Both | Colon and rectum cancer | Age-standardized | -1.351428434 | -1.489078959 | -1.213585569 | -1.35 (-1.49 to -1.21) |
| 29 | Philippines | Deaths | Both | Colon and rectum cancer | Age-standardized | 1.543219459 | 1.454811104 | 1.631704853 | 1.54 (1.45 to 1.63) |
| 30 | Philippines | DALYs | Both | Colon and rectum cancer | Age-standardized | 1.716300586 | 1.641909213 | 1.790746406 | 1.72 (1.64 to 1.79) |
| 31 | Philippines | YLDs | Both | Colon and rectum cancer | Age-standardized | 1.950666772 | 1.890358104 | 2.011011136 | 1.95 (1.89 to 2.01) |
| 32 | Philippines | YLLs | Both | Colon and rectum cancer | Age-standardized | 1.711866524 | 1.6369212 | 1.786867111 | 1.71 (1.64 to 1.79) |
| 33 | Thailand | Deaths | Both | Colon and rectum cancer | Age-standardized | 1.061870075 | 0.92230272 | 1.20163044 | 1.06 (0.92 to 1.2) |
| 34 | Thailand | DALYs | Both | Colon and rectum cancer | Age-standardized | 1.233902954 | 1.101639851 | 1.366339086 | 1.23 (1.1 to 1.37) |
| 35 | Thailand | YLDs | Both | Colon and rectum cancer | Age-standardized | 2.984122549 | 2.850576829 | 3.117841671 | 2.98 (2.85 to 3.12) |
| 36 | Thailand | YLLs | Both | Colon and rectum cancer | Age-standardized | 1.187041676 | 1.054188927 | 1.320069083 | 1.19 (1.05 to 1.32) |
| 37 | Malaysia | Deaths | Both | Colon and rectum cancer | Age-standardized | 0.934324553 | 0.784365915 | 1.084506316 | 0.93 (0.78 to 1.08) |
| 38 | Malaysia | DALYs | Both | Colon and rectum cancer | Age-standardized | 0.839525526 | 0.695701153 | 0.983555325 | 0.84 (0.7 to 0.98) |
| 39 | Malaysia | YLDs | Both | Colon and rectum cancer | Age-standardized | 2.112245363 | 1.98375025 | 2.240902374 | 2.11 (1.98 to 2.24) |
| 40 | Malaysia | YLLs | Both | Colon and rectum cancer | Age-standardized | 0.810327267 | 0.666271453 | 0.954589228 | 0.81 (0.67 to 0.95) |
| 41 | Myanmar | Deaths | Both | Colon and rectum cancer | Age-standardized | 0.929934047 | 0.834669179 | 1.025288918 | 0.93 (0.83 to 1.03) |
| 42 | Myanmar | DALYs | Both | Colon and rectum cancer | Age-standardized | 0.807022241 | 0.702155185 | 0.911998502 | 0.81 (0.7 to 0.91) |
| 43 | Myanmar | YLDs | Both | Colon and rectum cancer | Age-standardized | 1.681137104 | 1.629411754 | 1.73288878 | 1.68 (1.63 to 1.73) |
| 44 | Myanmar | YLLs | Both | Colon and rectum cancer | Age-standardized | 0.79189523 | 0.685843666 | 0.898058497 | 0.79 (0.69 to 0.9) |
| 45 | Sri Lanka | Deaths | Both | Colon and rectum cancer | Age-standardized | 1.353634424 | 1.121032818 | 1.586771067 | 1.35 (1.12 to 1.59) |
| 46 | Sri Lanka | DALYs | Both | Colon and rectum cancer | Age-standardized | 1.542907518 | 1.2782942 | 1.808212201 | 1.54 (1.28 to 1.81) |
| 47 | Sri Lanka | YLDs | Both | Colon and rectum cancer | Age-standardized | 3.157679791 | 2.931572044 | 3.384284225 | 3.16 (2.93 to 3.38) |
| 48 | Sri Lanka | YLLs | Both | Colon and rectum cancer | Age-standardized | 1.499728616 | 1.233026032 | 1.767133839 | 1.5 (1.23 to 1.77) |
| 49 | Viet Nam | Deaths | Both | Colon and rectum cancer | Age-standardized | 2.620176786 | 2.401086652 | 2.839735671 | 2.62 (2.4 to 2.84) |
| 50 | Viet Nam | DALYs | Both | Colon and rectum cancer | Age-standardized | 2.662973103 | 2.48346032 | 2.842800326 | 2.66 (2.48 to 2.84) |
| 51 | Viet Nam | YLDs | Both | Colon and rectum cancer | Age-standardized | 4.120700384 | 3.97076605 | 4.270850936 | 4.12 (3.97 to 4.27) |
| 52 | Viet Nam | YLLs | Both | Colon and rectum cancer | Age-standardized | 2.627701934 | 2.446751615 | 2.808971863 | 2.63 (2.45 to 2.81) |
| 53 | Timor-Leste | Deaths | Both | Colon and rectum cancer | Age-standardized | 2.231743956 | 2.076986599 | 2.386735938 | 2.23 (2.08 to 2.39) |
| 54 | Timor-Leste | DALYs | Both | Colon and rectum cancer | Age-standardized | 2.230970974 | 2.12491728 | 2.337134802 | 2.23 (2.12 to 2.34) |
| 55 | Timor-Leste | YLDs | Both | Colon and rectum cancer | Age-standardized | 2.803759706 | 2.687596842 | 2.920053976 | 2.8 (2.69 to 2.92) |
| 56 | Timor-Leste | YLLs | Both | Colon and rectum cancer | Age-standardized | 2.220818934 | 2.114691304 | 2.327056862 | 2.22 (2.11 to 2.33) |
| 57 | Fiji | Deaths | Both | Colon and rectum cancer | Age-standardized | 0.995804187 | 0.84589176 | 1.145939466 | 1 (0.85 to 1.15) |
| 58 | Fiji | DALYs | Both | Colon and rectum cancer | Age-standardized | 0.985889166 | 0.82109077 | 1.150956936 | 0.99 (0.82 to 1.15) |
| 59 | Fiji | YLDs | Both | Colon and rectum cancer | Age-standardized | 1.16580493 | 0.992104129 | 1.339804488 | 1.17 (0.99 to 1.34) |
| 60 | Fiji | YLLs | Both | Colon and rectum cancer | Age-standardized | 0.982501943 | 0.817834284 | 1.147438557 | 0.98 (0.82 to 1.15) |
| 61 | Marshall Islands | Deaths | Both | Colon and rectum cancer | Age-standardized | 0.707777068 | 0.655797008 | 0.759783971 | 0.71 (0.66 to 0.76) |
| 62 | Marshall Islands | DALYs | Both | Colon and rectum cancer | Age-standardized | 0.711769367 | 0.65821419 | 0.765353038 | 0.71 (0.66 to 0.77) |
| 63 | Marshall Islands | YLDs | Both | Colon and rectum cancer | Age-standardized | 0.944741986 | 0.883194667 | 1.006326855 | 0.94 (0.88 to 1.01) |
| 64 | Marshall Islands | YLLs | Both | Colon and rectum cancer | Age-standardized | 0.707862504 | 0.654164981 | 0.761588674 | 0.71 (0.65 to 0.76) |
| 65 | Kiribati | Deaths | Both | Colon and rectum cancer | Age-standardized | 0.436585553 | 0.340814079 | 0.532448437 | 0.44 (0.34 to 0.53) |
| 66 | Kiribati | DALYs | Both | Colon and rectum cancer | Age-standardized | 0.302084214 | 0.210275953 | 0.393976585 | 0.3 (0.21 to 0.39) |
| 67 | Kiribati | YLDs | Both | Colon and rectum cancer | Age-standardized | 0.556197599 | 0.467739965 | 0.644733117 | 0.56 (0.47 to 0.64) |
| 68 | Kiribati | YLLs | Both | Colon and rectum cancer | Age-standardized | 0.298141135 | 0.206263486 | 0.390103024 | 0.3 (0.21 to 0.39) |
| 69 | Micronesia (Federated States of) | Deaths | Both | Colon and rectum cancer | Age-standardized | 0.232938784 | 0.151620272 | 0.314323323 | 0.23 (0.15 to 0.31) |
| 70 | Micronesia (Federated States of) | DALYs | Both | Colon and rectum cancer | Age-standardized | 0.226883103 | 0.142445379 | 0.311392022 | 0.23 (0.14 to 0.31) |
| 71 | Micronesia (Federated States of) | YLDs | Both | Colon and rectum cancer | Age-standardized | 0.745873354 | 0.664450082 | 0.827362485 | 0.75 (0.66 to 0.83) |
| 72 | Micronesia (Federated States of) | YLLs | Both | Colon and rectum cancer | Age-standardized | 0.218143333 | 0.133574234 | 0.302783857 | 0.22 (0.13 to 0.3) |
| 73 | Papua New Guinea | Deaths | Both | Colon and rectum cancer | Age-standardized | 0.36533064 | 0.319664733 | 0.411017335 | 0.37 (0.32 to 0.41) |
| 74 | Papua New Guinea | DALYs | Both | Colon and rectum cancer | Age-standardized | 0.269387785 | 0.214697144 | 0.324108273 | 0.27 (0.21 to 0.32) |
| 75 | Papua New Guinea | YLDs | Both | Colon and rectum cancer | Age-standardized | 0.435592368 | 0.366456228 | 0.504776131 | 0.44 (0.37 to 0.5) |
| 76 | Papua New Guinea | YLLs | Both | Colon and rectum cancer | Age-standardized | 0.266427466 | 0.211933232 | 0.320951334 | 0.27 (0.21 to 0.32) |
| 77 | Solomon Islands | Deaths | Both | Colon and rectum cancer | Age-standardized | 0.548366331 | 0.469295216 | 0.627499676 | 0.55 (0.47 to 0.63) |
| 78 | Solomon Islands | DALYs | Both | Colon and rectum cancer | Age-standardized | 0.541276907 | 0.461647752 | 0.620969179 | 0.54 (0.46 to 0.62) |
| 79 | Solomon Islands | YLDs | Both | Colon and rectum cancer | Age-standardized | 0.775214675 | 0.693806296 | 0.856688872 | 0.78 (0.69 to 0.86) |
| 80 | Solomon Islands | YLLs | Both | Colon and rectum cancer | Age-standardized | 0.537370338 | 0.457712634 | 0.617091206 | 0.54 (0.46 to 0.62) |
| 81 | Samoa | Deaths | Both | Colon and rectum cancer | Age-standardized | 0.706922112 | 0.639824442 | 0.774064516 | 0.71 (0.64 to 0.77) |
| 82 | Samoa | DALYs | Both | Colon and rectum cancer | Age-standardized | 0.820463865 | 0.7482888 | 0.892690635 | 0.82 (0.75 to 0.89) |
| 83 | Samoa | YLDs | Both | Colon and rectum cancer | Age-standardized | 1.344011502 | 1.291816354 | 1.396233547 | 1.34 (1.29 to 1.4) |
| 84 | Samoa | YLLs | Both | Colon and rectum cancer | Age-standardized | 0.809999156 | 0.737460449 | 0.882590095 | 0.81 (0.74 to 0.88) |
| 85 | Vanuatu | Deaths | Both | Colon and rectum cancer | Age-standardized | 0.869327072 | 0.811301668 | 0.927385873 | 0.87 (0.81 to 0.93) |
| 86 | Vanuatu | DALYs | Both | Colon and rectum cancer | Age-standardized | 0.868252211 | 0.794892922 | 0.941664891 | 0.87 (0.79 to 0.94) |
| 87 | Vanuatu | YLDs | Both | Colon and rectum cancer | Age-standardized | 1.018569522 | 0.947529701 | 1.089659336 | 1.02 (0.95 to 1.09) |
| 88 | Vanuatu | YLLs | Both | Colon and rectum cancer | Age-standardized | 0.865741128 | 0.79231083 | 0.939224922 | 0.87 (0.79 to 0.94) |
| 89 | Tonga | Deaths | Both | Colon and rectum cancer | Age-standardized | 0.728512697 | 0.594451076 | 0.86275298 | 0.73 (0.59 to 0.86) |
| 90 | Tonga | DALYs | Both | Colon and rectum cancer | Age-standardized | 0.625491394 | 0.484824249 | 0.766355457 | 0.63 (0.48 to 0.77) |
| 91 | Tonga | YLDs | Both | Colon and rectum cancer | Age-standardized | 1.069916795 | 0.952539098 | 1.187430967 | 1.07 (0.95 to 1.19) |
| 92 | Tonga | YLLs | Both | Colon and rectum cancer | Age-standardized | 0.616187917 | 0.474938167 | 0.757636239 | 0.62 (0.47 to 0.76) |
| 93 | Azerbaijan | Deaths | Both | Colon and rectum cancer | Age-standardized | 2.170323361 | 1.783510665 | 2.55860608 | 2.17 (1.78 to 2.56) |
| 94 | Azerbaijan | DALYs | Both | Colon and rectum cancer | Age-standardized | 1.74382118 | 1.404043631 | 2.084737231 | 1.74 (1.4 to 2.08) |
| 95 | Azerbaijan | YLDs | Both | Colon and rectum cancer | Age-standardized | 2.69110851 | 2.266644956 | 3.117333824 | 2.69 (2.27 to 3.12) |
| 96 | Azerbaijan | YLLs | Both | Colon and rectum cancer | Age-standardized | 1.723893176 | 1.385711022 | 2.06320337 | 1.72 (1.39 to 2.06) |
| 97 | Armenia | Deaths | Both | Colon and rectum cancer | Age-standardized | 2.062130525 | 1.845163217 | 2.279560053 | 2.06 (1.85 to 2.28) |
| 98 | Armenia | DALYs | Both | Colon and rectum cancer | Age-standardized | 1.589285284 | 1.405973652 | 1.772928289 | 1.59 (1.41 to 1.77) |
| 99 | Armenia | YLDs | Both | Colon and rectum cancer | Age-standardized | 2.643366227 | 2.454845213 | 2.832234126 | 2.64 (2.45 to 2.83) |
| 100 | Armenia | YLLs | Both | Colon and rectum cancer | Age-standardized | 1.562697047 | 1.379283754 | 1.746442168 | 1.56 (1.38 to 1.75) |
| 101 | Kazakhstan | Deaths | Both | Colon and rectum cancer | Age-standardized | 1.555347849 | 1.198911892 | 1.913039221 | 1.56 (1.2 to 1.91) |
| 102 | Kazakhstan | DALYs | Both | Colon and rectum cancer | Age-standardized | 1.223544805 | 0.931413935 | 1.516521205 | 1.22 (0.93 to 1.52) |
| 103 | Kazakhstan | YLDs | Both | Colon and rectum cancer | Age-standardized | 2.330043376 | 2.059734931 | 2.601067741 | 2.33 (2.06 to 2.6) |
| 104 | Kazakhstan | YLLs | Both | Colon and rectum cancer | Age-standardized | 1.198182108 | 0.903494221 | 1.493730629 | 1.2 (0.9 to 1.49) |
| 105 | Georgia | Deaths | Both | Colon and rectum cancer | Age-standardized | 5.109452002 | 4.522233742 | 5.699969324 | 5.11 (4.52 to 5.7) |
| 106 | Georgia | DALYs | Both | Colon and rectum cancer | Age-standardized | 4.568244568 | 4.05261654 | 5.086427768 | 4.57 (4.05 to 5.09) |
| 107 | Georgia | YLDs | Both | Colon and rectum cancer | Age-standardized | 4.734904594 | 4.246122734 | 5.225978219 | 4.73 (4.25 to 5.23) |
| 108 | Georgia | YLLs | Both | Colon and rectum cancer | Age-standardized | 4.56417263 | 4.047868984 | 5.083038266 | 4.56 (4.05 to 5.08) |
| 109 | Mongolia | Deaths | Both | Colon and rectum cancer | Age-standardized | 1.951321513 | 1.72260325 | 2.180554038 | 1.95 (1.72 to 2.18) |
| 110 | Mongolia | DALYs | Both | Colon and rectum cancer | Age-standardized | 1.809770123 | 1.574009612 | 2.046077852 | 1.81 (1.57 to 2.05) |
| 111 | Mongolia | YLDs | Both | Colon and rectum cancer | Age-standardized | 2.69172153 | 2.416922279 | 2.967258106 | 2.69 (2.42 to 2.97) |
| 112 | Mongolia | YLLs | Both | Colon and rectum cancer | Age-standardized | 1.794197913 | 1.559080174 | 2.02985997 | 1.79 (1.56 to 2.03) |
| 113 | Kyrgyzstan | Deaths | Both | Colon and rectum cancer | Age-standardized | 0.440340541 | 0.241540479 | 0.639534865 | 0.44 (0.24 to 0.64) |
| 114 | Kyrgyzstan | DALYs | Both | Colon and rectum cancer | Age-standardized | 0.225841958 | 0.073927629 | 0.377986897 | 0.23 (0.07 to 0.38) |
| 115 | Kyrgyzstan | YLDs | Both | Colon and rectum cancer | Age-standardized | 1.077893472 | 0.866705204 | 1.289523912 | 1.08 (0.87 to 1.29) |
| 116 | Kyrgyzstan | YLLs | Both | Colon and rectum cancer | Age-standardized | 0.207598004 | 0.056773909 | 0.358649449 | 0.21 (0.06 to 0.36) |
| 117 | Turkmenistan | Deaths | Both | Colon and rectum cancer | Age-standardized | 1.317119999 | 0.785749918 | 1.851291607 | 1.32 (0.79 to 1.85) |
| 118 | Turkmenistan | DALYs | Both | Colon and rectum cancer | Age-standardized | 1.341067436 | 0.82187341 | 1.862935111 | 1.34 (0.82 to 1.86) |
| 119 | Turkmenistan | YLDs | Both | Colon and rectum cancer | Age-standardized | 1.879703845 | 1.3525745 | 2.409574762 | 1.88 (1.35 to 2.41) |
| 120 | Turkmenistan | YLLs | Both | Colon and rectum cancer | Age-standardized | 1.330226422 | 0.811241557 | 1.851883065 | 1.33 (0.81 to 1.85) |
| 121 | Tajikistan | Deaths | Both | Colon and rectum cancer | Age-standardized | -0.105265118 | -0.359161553 | 0.149278275 | -0.11 (-0.36 to 0.15) |
| 122 | Tajikistan | DALYs | Both | Colon and rectum cancer | Age-standardized | -0.335755927 | -0.566137116 | -0.104840962 | -0.34 (-0.57 to -0.1) |
| 123 | Tajikistan | YLDs | Both | Colon and rectum cancer | Age-standardized | 0.045988154 | -0.193069755 | 0.285618655 | 0.05 (-0.19 to 0.29) |
| 124 | Tajikistan | YLLs | Both | Colon and rectum cancer | Age-standardized | -0.34297728 | -0.573289066 | -0.112132 | -0.34 (-0.57 to -0.11) |
| 125 | Uzbekistan | Deaths | Both | Colon and rectum cancer | Age-standardized | 2.365330565 | 1.924337945 | 2.808231214 | 2.37 (1.92 to 2.81) |
| 126 | Uzbekistan | DALYs | Both | Colon and rectum cancer | Age-standardized | 2.091579645 | 1.661618967 | 2.523358769 | 2.09 (1.66 to 2.52) |
| 127 | Uzbekistan | YLDs | Both | Colon and rectum cancer | Age-standardized | 2.58509985 | 2.125634605 | 3.046632239 | 2.59 (2.13 to 3.05) |
| 128 | Uzbekistan | YLLs | Both | Colon and rectum cancer | Age-standardized | 2.081389666 | 1.652002658 | 2.512590443 | 2.08 (1.65 to 2.51) |
| 129 | Bosnia and Herzegovina | Deaths | Both | Colon and rectum cancer | Age-standardized | 3.542593169 | 3.1884041 | 3.897997975 | 3.54 (3.19 to 3.9) |
| 130 | Bosnia and Herzegovina | DALYs | Both | Colon and rectum cancer | Age-standardized | 3.411505753 | 3.026605399 | 3.797844069 | 3.41 (3.03 to 3.8) |
| 131 | Bosnia and Herzegovina | YLDs | Both | Colon and rectum cancer | Age-standardized | 4.859156312 | 4.368567009 | 5.352051653 | 4.86 (4.37 to 5.35) |
| 132 | Bosnia and Herzegovina | YLLs | Both | Colon and rectum cancer | Age-standardized | 3.371391298 | 2.988731108 | 3.755473282 | 3.37 (2.99 to 3.76) |
| 133 | Albania | Deaths | Both | Colon and rectum cancer | Age-standardized | 1.635181549 | 1.437902534 | 1.832844238 | 1.64 (1.44 to 1.83) |
| 134 | Albania | DALYs | Both | Colon and rectum cancer | Age-standardized | 1.537933321 | 1.335011712 | 1.741261277 | 1.54 (1.34 to 1.74) |
| 135 | Albania | YLDs | Both | Colon and rectum cancer | Age-standardized | 3.078436977 | 2.864920183 | 3.292396967 | 3.08 (2.86 to 3.29) |
| 136 | Albania | YLLs | Both | Colon and rectum cancer | Age-standardized | 1.494525169 | 1.291250499 | 1.698207777 | 1.49 (1.29 to 1.7) |
| 137 | Croatia | Deaths | Both | Colon and rectum cancer | Age-standardized | 1.683420261 | 1.468137382 | 1.899159901 | 1.68 (1.47 to 1.9) |
| 138 | Croatia | DALYs | Both | Colon and rectum cancer | Age-standardized | 1.660683962 | 1.422576567 | 1.899350356 | 1.66 (1.42 to 1.9) |
| 139 | Croatia | YLDs | Both | Colon and rectum cancer | Age-standardized | 3.252028215 | 3.019288811 | 3.48529342 | 3.25 (3.02 to 3.49) |
| 140 | Croatia | YLLs | Both | Colon and rectum cancer | Age-standardized | 1.600264822 | 1.360693036 | 1.84040285 | 1.6 (1.36 to 1.84) |
| 141 | Bulgaria | Deaths | Both | Colon and rectum cancer | Age-standardized | 2.334834188 | 2.096609581 | 2.57361465 | 2.33 (2.1 to 2.57) |
| 142 | Bulgaria | DALYs | Both | Colon and rectum cancer | Age-standardized | 2.11754849 | 1.926929414 | 2.308524054 | 2.12 (1.93 to 2.31) |
| 143 | Bulgaria | YLDs | Both | Colon and rectum cancer | Age-standardized | 3.361800707 | 3.192297011 | 3.531582829 | 3.36 (3.19 to 3.53) |
| 144 | Bulgaria | YLLs | Both | Colon and rectum cancer | Age-standardized | 2.079026296 | 1.887244816 | 2.271168764 | 2.08 (1.89 to 2.27) |
| 145 | Hungary | Deaths | Both | Colon and rectum cancer | Age-standardized | 0.436506697 | 0.067304643 | 0.807070937 | 0.44 (0.07 to 0.81) |
| 146 | Hungary | DALYs | Both | Colon and rectum cancer | Age-standardized | 0.657529953 | 0.293871554 | 1.022506951 | 0.66 (0.29 to 1.02) |
| 147 | Hungary | YLDs | Both | Colon and rectum cancer | Age-standardized | 2.184830637 | 1.743017612 | 2.62856221 | 2.18 (1.74 to 2.63) |
| 148 | Hungary | YLLs | Both | Colon and rectum cancer | Age-standardized | 0.609008181 | 0.246584339 | 0.972742302 | 0.61 (0.25 to 0.97) |
| 149 | Czechia | Deaths | Both | Colon and rectum cancer | Age-standardized | -0.299753931 | -0.537407147 | -0.061532873 | -0.3 (-0.54 to -0.06) |
| 150 | Czechia | DALYs | Both | Colon and rectum cancer | Age-standardized | -0.41451049 | -0.652238231 | -0.176213893 | -0.41 (-0.65 to -0.18) |
| 151 | Czechia | YLDs | Both | Colon and rectum cancer | Age-standardized | 1.439216108 | 1.046528166 | 1.833430118 | 1.44 (1.05 to 1.83) |
| 152 | Czechia | YLLs | Both | Colon and rectum cancer | Age-standardized | -0.48021197 | -0.71462381 | -0.245246687 | -0.48 (-0.71 to -0.25) |
| 153 | Montenegro | Deaths | Both | Colon and rectum cancer | Age-standardized | 2.304636788 | 2.1766837 | 2.432750108 | 2.3 (2.18 to 2.43) |
| 154 | Montenegro | DALYs | Both | Colon and rectum cancer | Age-standardized | 2.000538582 | 1.921493097 | 2.079645371 | 2 (1.92 to 2.08) |
| 155 | Montenegro | YLDs | Both | Colon and rectum cancer | Age-standardized | 2.725027113 | 2.532945685 | 2.917468379 | 2.73 (2.53 to 2.92) |
| 156 | Montenegro | YLLs | Both | Colon and rectum cancer | Age-standardized | 1.974622278 | 1.896007369 | 2.053297841 | 1.97 (1.9 to 2.05) |
| 157 | North Macedonia | Deaths | Both | Colon and rectum cancer | Age-standardized | 2.01874583 | 1.540416203 | 2.499328739 | 2.02 (1.54 to 2.5) |
| 158 | North Macedonia | DALYs | Both | Colon and rectum cancer | Age-standardized | 1.770188377 | 1.352098646 | 2.190002779 | 1.77 (1.35 to 2.19) |
| 159 | North Macedonia | YLDs | Both | Colon and rectum cancer | Age-standardized | 3.038967054 | 2.599220418 | 3.480598471 | 3.04 (2.6 to 3.48) |
| 160 | North Macedonia | YLLs | Both | Colon and rectum cancer | Age-standardized | 1.735730638 | 1.317543588 | 2.155643751 | 1.74 (1.32 to 2.16) |
| 161 | Romania | Deaths | Both | Colon and rectum cancer | Age-standardized | 3.121924547 | 2.893487935 | 3.350868317 | 3.12 (2.89 to 3.35) |
| 162 | Romania | DALYs | Both | Colon and rectum cancer | Age-standardized | 3.002718305 | 2.769694567 | 3.23627041 | 3 (2.77 to 3.24) |
| 163 | Romania | YLDs | Both | Colon and rectum cancer | Age-standardized | 4.842227849 | 4.621532046 | 5.063389202 | 4.84 (4.62 to 5.06) |
| 164 | Romania | YLLs | Both | Colon and rectum cancer | Age-standardized | 2.949138513 | 2.715320954 | 3.183488326 | 2.95 (2.72 to 3.18) |
| 165 | Poland | Deaths | Both | Colon and rectum cancer | Age-standardized | 1.449665509 | 1.328652387 | 1.570823153 | 1.45 (1.33 to 1.57) |
| 166 | Poland | DALYs | Both | Colon and rectum cancer | Age-standardized | 1.336220389 | 1.2160195 | 1.456564026 | 1.34 (1.22 to 1.46) |
| 167 | Poland | YLDs | Both | Colon and rectum cancer | Age-standardized | 2.852302548 | 2.68312312 | 3.021760715 | 2.85 (2.68 to 3.02) |
| 168 | Poland | YLLs | Both | Colon and rectum cancer | Age-standardized | 1.300529632 | 1.180588899 | 1.420612544 | 1.3 (1.18 to 1.42) |
| 169 | Slovakia | Deaths | Both | Colon and rectum cancer | Age-standardized | 0.788282971 | 0.632244848 | 0.944563043 | 0.79 (0.63 to 0.94) |
| 170 | Slovakia | DALYs | Both | Colon and rectum cancer | Age-standardized | 0.620274053 | 0.457343251 | 0.783469111 | 0.62 (0.46 to 0.78) |
| 171 | Slovakia | YLDs | Both | Colon and rectum cancer | Age-standardized | 2.292023025 | 2.14241703 | 2.441848144 | 2.29 (2.14 to 2.44) |
| 172 | Slovakia | YLLs | Both | Colon and rectum cancer | Age-standardized | 0.555209868 | 0.390319032 | 0.720371536 | 0.56 (0.39 to 0.72) |
| 173 | Serbia | Deaths | Both | Colon and rectum cancer | Age-standardized | 0.72225386 | 0.589894335 | 0.85478755 | 0.72 (0.59 to 0.85) |
| 174 | Serbia | DALYs | Both | Colon and rectum cancer | Age-standardized | 0.828219713 | 0.661333874 | 0.995382231 | 0.83 (0.66 to 1) |
| 175 | Serbia | YLDs | Both | Colon and rectum cancer | Age-standardized | 2.437116604 | 2.243098441 | 2.631502939 | 2.44 (2.24 to 2.63) |
| 176 | Serbia | YLLs | Both | Colon and rectum cancer | Age-standardized | 0.785018056 | 0.617685229 | 0.952629166 | 0.79 (0.62 to 0.95) |
| 177 | Slovenia | Deaths | Both | Colon and rectum cancer | Age-standardized | -0.00470549 | -0.38302563 | 0.375051415 | 0 (-0.38 to 0.38) |
| 178 | Slovenia | DALYs | Both | Colon and rectum cancer | Age-standardized | -0.264285916 | -0.663758186 | 0.136792799 | -0.26 (-0.66 to 0.14) |
| 179 | Slovenia | YLDs | Both | Colon and rectum cancer | Age-standardized | 1.879735037 | 1.418669245 | 2.342896909 | 1.88 (1.42 to 2.34) |
| 180 | Slovenia | YLLs | Both | Colon and rectum cancer | Age-standardized | -0.34673121 | -0.746311156 | 0.054457382 | -0.35 (-0.75 to 0.05) |
| 181 | Estonia | Deaths | Both | Colon and rectum cancer | Age-standardized | 1.649806872 | 1.529254641 | 1.770502242 | 1.65 (1.53 to 1.77) |
| 182 | Estonia | DALYs | Both | Colon and rectum cancer | Age-standardized | 1.207311028 | 1.081534894 | 1.333243666 | 1.21 (1.08 to 1.33) |
| 183 | Estonia | YLDs | Both | Colon and rectum cancer | Age-standardized | 3.380272073 | 3.206032984 | 3.554805323 | 3.38 (3.21 to 3.55) |
| 184 | Estonia | YLLs | Both | Colon and rectum cancer | Age-standardized | 1.115670196 | 0.988091153 | 1.243410411 | 1.12 (0.99 to 1.24) |
| 185 | Belarus | Deaths | Both | Colon and rectum cancer | Age-standardized | 0.750847122 | 0.476360487 | 1.026083613 | 0.75 (0.48 to 1.03) |
| 186 | Belarus | DALYs | Both | Colon and rectum cancer | Age-standardized | 0.546086321 | 0.266567511 | 0.826384362 | 0.55 (0.27 to 0.83) |
| 187 | Belarus | YLDs | Both | Colon and rectum cancer | Age-standardized | 2.252861274 | 1.933864965 | 2.572855864 | 2.25 (1.93 to 2.57) |
| 188 | Belarus | YLLs | Both | Colon and rectum cancer | Age-standardized | 0.486981777 | 0.207244172 | 0.767500295 | 0.49 (0.21 to 0.77) |
| 189 | Lithuania | Deaths | Both | Colon and rectum cancer | Age-standardized | 1.603794744 | 1.390816637 | 1.817220227 | 1.6 (1.39 to 1.82) |
| 190 | Lithuania | DALYs | Both | Colon and rectum cancer | Age-standardized | 1.355906135 | 1.133211858 | 1.579090783 | 1.36 (1.13 to 1.58) |
| 191 | Lithuania | YLDs | Both | Colon and rectum cancer | Age-standardized | 2.237495048 | 2.084093725 | 2.391126886 | 2.24 (2.08 to 2.39) |
| 192 | Lithuania | YLLs | Both | Colon and rectum cancer | Age-standardized | 1.32601575 | 1.100472133 | 1.552062528 | 1.33 (1.1 to 1.55) |
| 193 | Latvia | Deaths | Both | Colon and rectum cancer | Age-standardized | 1.72671254 | 1.481062745 | 1.972956966 | 1.73 (1.48 to 1.97) |
| 194 | Latvia | DALYs | Both | Colon and rectum cancer | Age-standardized | 1.455037548 | 1.226814467 | 1.683775174 | 1.46 (1.23 to 1.68) |
| 195 | Latvia | YLDs | Both | Colon and rectum cancer | Age-standardized | 2.612636056 | 2.426201163 | 2.799410295 | 2.61 (2.43 to 2.8) |
| 196 | Latvia | YLLs | Both | Colon and rectum cancer | Age-standardized | 1.417770069 | 1.186985294 | 1.649081212 | 1.42 (1.19 to 1.65) |
| 197 | Russian Federation | Deaths | Both | Colon and rectum cancer | Age-standardized | 1.960111736 | 1.819488878 | 2.100928808 | 1.96 (1.82 to 2.1) |
| 198 | Russian Federation | DALYs | Both | Colon and rectum cancer | Age-standardized | 1.599793687 | 1.448845727 | 1.750966246 | 1.6 (1.45 to 1.75) |
| 199 | Russian Federation | YLDs | Both | Colon and rectum cancer | Age-standardized | 3.112132601 | 2.928215431 | 3.296378403 | 3.11 (2.93 to 3.3) |
| 200 | Russian Federation | YLLs | Both | Colon and rectum cancer | Age-standardized | 1.555844573 | 1.400583758 | 1.711343117 | 1.56 (1.4 to 1.71) |
| 201 | Republic of Moldova | Deaths | Both | Colon and rectum cancer | Age-standardized | 1.945874361 | 1.423374777 | 2.471065689 | 1.95 (1.42 to 2.47) |
| 202 | Republic of Moldova | DALYs | Both | Colon and rectum cancer | Age-standardized | 1.988856147 | 1.507088244 | 2.472910594 | 1.99 (1.51 to 2.47) |
| 203 | Republic of Moldova | YLDs | Both | Colon and rectum cancer | Age-standardized | 3.174480744 | 2.646360895 | 3.705317792 | 3.17 (2.65 to 3.71) |
| 204 | Republic of Moldova | YLLs | Both | Colon and rectum cancer | Age-standardized | 1.956233354 | 1.474878539 | 2.439871517 | 1.96 (1.47 to 2.44) |
| 205 | Ukraine | Deaths | Both | Colon and rectum cancer | Age-standardized | 0.097312667 | -0.031621854 | 0.226413482 | 0.1 (-0.03 to 0.23) |
| 206 | Ukraine | DALYs | Both | Colon and rectum cancer | Age-standardized | 0.008186097 | -0.121411158 | 0.137951511 | 0.01 (-0.12 to 0.14) |
| 207 | Ukraine | YLDs | Both | Colon and rectum cancer | Age-standardized | 0.657219404 | 0.534279761 | 0.780309385 | 0.66 (0.53 to 0.78) |
| 208 | Ukraine | YLLs | Both | Colon and rectum cancer | Age-standardized | -0.009627448 | -0.140529959 | 0.121446658 | -0.01 (-0.14 to 0.12) |
| 209 | Brunei Darussalam | Deaths | Both | Colon and rectum cancer | Age-standardized | -0.101486595 | -0.388147245 | 0.185999 | -0.1 (-0.39 to 0.19) |
| 210 | Brunei Darussalam | DALYs | Both | Colon and rectum cancer | Age-standardized | -0.312274751 | -0.582316296 | -0.041499711 | -0.31 (-0.58 to -0.04) |
| 211 | Brunei Darussalam | YLDs | Both | Colon and rectum cancer | Age-standardized | 0.542080813 | 0.345953715 | 0.738591243 | 0.54 (0.35 to 0.74) |
| 212 | Brunei Darussalam | YLLs | Both | Colon and rectum cancer | Age-standardized | -0.335101493 | -0.606986034 | -0.062473226 | -0.34 (-0.61 to -0.06) |
| 213 | Republic of Korea | Deaths | Both | Colon and rectum cancer | Age-standardized | 0.589789987 | 0.383097221 | 0.796908341 | 0.59 (0.38 to 0.8) |
| 214 | Republic of Korea | DALYs | Both | Colon and rectum cancer | Age-standardized | 0.32891644 | 0.106624647 | 0.551701843 | 0.33 (0.11 to 0.55) |
| 215 | Republic of Korea | YLDs | Both | Colon and rectum cancer | Age-standardized | 4.035985337 | 3.540629754 | 4.533710783 | 4.04 (3.54 to 4.53) |
| 216 | Republic of Korea | YLLs | Both | Colon and rectum cancer | Age-standardized | 0.140996945 | -0.076223436 | 0.358689534 | 0.14 (-0.08 to 0.36) |
| 217 | Japan | Deaths | Both | Colon and rectum cancer | Age-standardized | -0.482241455 | -0.570623537 | -0.393780812 | -0.48 (-0.57 to -0.39) |
| 218 | Japan | DALYs | Both | Colon and rectum cancer | Age-standardized | -0.657332056 | -0.761554641 | -0.553000015 | -0.66 (-0.76 to -0.55) |
| 219 | Japan | YLDs | Both | Colon and rectum cancer | Age-standardized | 0.545542625 | 0.402833914 | 0.688454176 | 0.55 (0.4 to 0.69) |
| 220 | Japan | YLLs | Both | Colon and rectum cancer | Age-standardized | -0.744771731 | -0.848272357 | -0.641163064 | -0.74 (-0.85 to -0.64) |
| 221 | Singapore | Deaths | Both | Colon and rectum cancer | Age-standardized | -1.964338154 | -2.167107506 | -1.761148541 | -1.96 (-2.17 to -1.76) |
| 222 | Singapore | DALYs | Both | Colon and rectum cancer | Age-standardized | -2.121874547 | -2.328918969 | -1.914391231 | -2.12 (-2.33 to -1.91) |
| 223 | Singapore | YLDs | Both | Colon and rectum cancer | Age-standardized | 0.508142397 | 0.32955251 | 0.68705018 | 0.51 (0.33 to 0.69) |
| 224 | Singapore | YLLs | Both | Colon and rectum cancer | Age-standardized | -2.268059971 | -2.478963423 | -2.05670041 | -2.27 (-2.48 to -2.06) |
| 225 | Australia | Deaths | Both | Colon and rectum cancer | Age-standardized | -0.770896211 | -0.857211209 | -0.684506067 | -0.77 (-0.86 to -0.68) |
| 226 | Australia | DALYs | Both | Colon and rectum cancer | Age-standardized | -0.861330028 | -0.952971119 | -0.769604148 | -0.86 (-0.95 to -0.77) |
| 227 | Australia | YLDs | Both | Colon and rectum cancer | Age-standardized | 1.018153844 | 0.763414872 | 1.273536819 | 1.02 (0.76 to 1.27) |
| 228 | Australia | YLLs | Both | Colon and rectum cancer | Age-standardized | -0.98539499 | -1.076974039 | -0.89373116 | -0.99 (-1.08 to -0.89) |
| 229 | New Zealand | Deaths | Both | Colon and rectum cancer | Age-standardized | -0.51287563 | -0.619657568 | -0.405978958 | -0.51 (-0.62 to -0.41) |
| 230 | New Zealand | DALYs | Both | Colon and rectum cancer | Age-standardized | -0.845417641 | -0.947034845 | -0.743696189 | -0.85 (-0.95 to -0.74) |
| 231 | New Zealand | YLDs | Both | Colon and rectum cancer | Age-standardized | 0.870603081 | 0.8079364 | 0.933308719 | 0.87 (0.81 to 0.93) |
| 232 | New Zealand | YLLs | Both | Colon and rectum cancer | Age-standardized | -0.951602804 | -1.05708766 | -0.846005488 | -0.95 (-1.06 to -0.85) |
| 233 | Andorra | Deaths | Both | Colon and rectum cancer | Age-standardized | 0.079661722 | -0.212636818 | 0.372816467 | 0.08 (-0.21 to 0.37) |
| 234 | Andorra | DALYs | Both | Colon and rectum cancer | Age-standardized | 0.022086971 | -0.252869391 | 0.29780126 | 0.02 (-0.25 to 0.3) |
| 235 | Andorra | YLDs | Both | Colon and rectum cancer | Age-standardized | 1.463676603 | 1.223128062 | 1.704796789 | 1.46 (1.22 to 1.7) |
| 236 | Andorra | YLLs | Both | Colon and rectum cancer | Age-standardized | -0.067114957 | -0.345764192 | 0.212313426 | -0.07 (-0.35 to 0.21) |
| 237 | Austria | Deaths | Both | Colon and rectum cancer | Age-standardized | -0.872032286 | -1.021057636 | -0.722782559 | -0.87 (-1.02 to -0.72) |
| 238 | Austria | DALYs | Both | Colon and rectum cancer | Age-standardized | -0.803876715 | -0.959184716 | -0.648325172 | -0.8 (-0.96 to -0.65) |
| 239 | Austria | YLDs | Both | Colon and rectum cancer | Age-standardized | 0.933028121 | 0.652133625 | 1.214706523 | 0.93 (0.65 to 1.21) |
| 240 | Austria | YLLs | Both | Colon and rectum cancer | Age-standardized | -0.896757656 | -1.048191399 | -0.745092161 | -0.9 (-1.05 to -0.75) |
| 241 | Belgium | Deaths | Both | Colon and rectum cancer | Age-standardized | -0.726203302 | -0.818190662 | -0.634130629 | -0.73 (-0.82 to -0.63) |
| 242 | Belgium | DALYs | Both | Colon and rectum cancer | Age-standardized | -0.650169396 | -0.753145626 | -0.547086319 | -0.65 (-0.75 to -0.55) |
| 243 | Belgium | YLDs | Both | Colon and rectum cancer | Age-standardized | 0.877975615 | 0.743763953 | 1.012366074 | 0.88 (0.74 to 1.01) |
| 244 | Belgium | YLLs | Both | Colon and rectum cancer | Age-standardized | -0.736355564 | -0.838916209 | -0.633688843 | -0.74 (-0.84 to -0.63) |
| 245 | Cyprus | Deaths | Both | Colon and rectum cancer | Age-standardized | -1.080581768 | -1.226333237 | -0.934615227 | -1.08 (-1.23 to -0.93) |
| 246 | Cyprus | DALYs | Both | Colon and rectum cancer | Age-standardized | -0.801569358 | -0.933468877 | -0.669494224 | -0.8 (-0.93 to -0.67) |
| 247 | Cyprus | YLDs | Both | Colon and rectum cancer | Age-standardized | 1.955812819 | 1.599582082 | 2.313292579 | 1.96 (1.6 to 2.31) |
| 248 | Cyprus | YLLs | Both | Colon and rectum cancer | Age-standardized | -0.962499907 | -1.084822615 | -0.840025929 | -0.96 (-1.08 to -0.84) |
| 249 | Denmark | Deaths | Both | Colon and rectum cancer | Age-standardized | 0.743181661 | 0.404889054 | 1.082614072 | 0.74 (0.4 to 1.08) |
| 250 | Denmark | DALYs | Both | Colon and rectum cancer | Age-standardized | 0.511158691 | 0.180343179 | 0.843066622 | 0.51 (0.18 to 0.84) |
| 251 | Denmark | YLDs | Both | Colon and rectum cancer | Age-standardized | 2.791820327 | 2.415760036 | 3.169261473 | 2.79 (2.42 to 3.17) |
| 252 | Denmark | YLLs | Both | Colon and rectum cancer | Age-standardized | 0.402098786 | 0.069508261 | 0.735794708 | 0.4 (0.07 to 0.74) |
| 253 | Finland | Deaths | Both | Colon and rectum cancer | Age-standardized | 0.190823316 | 0.021943906 | 0.359987866 | 0.19 (0.02 to 0.36) |
| 254 | Finland | DALYs | Both | Colon and rectum cancer | Age-standardized | 0.198456687 | 0.069446293 | 0.327633403 | 0.2 (0.07 to 0.33) |
| 255 | Finland | YLDs | Both | Colon and rectum cancer | Age-standardized | 2.118915627 | 2.031075247 | 2.206831629 | 2.12 (2.03 to 2.21) |
| 256 | Finland | YLLs | Both | Colon and rectum cancer | Age-standardized | 0.089085626 | -0.042026055 | 0.220369283 | 0.09 (-0.04 to 0.22) |
| 257 | France | Deaths | Both | Colon and rectum cancer | Age-standardized | -0.504004232 | -0.569912039 | -0.438052738 | -0.5 (-0.57 to -0.44) |
| 258 | France | DALYs | Both | Colon and rectum cancer | Age-standardized | -0.402982023 | -0.47552622 | -0.330384947 | -0.4 (-0.48 to -0.33) |
| 259 | France | YLDs | Both | Colon and rectum cancer | Age-standardized | 1.81389245 | 1.629667066 | 1.998451782 | 1.81 (1.63 to 2) |
| 260 | France | YLLs | Both | Colon and rectum cancer | Age-standardized | -0.530525824 | -0.601498488 | -0.459502485 | -0.53 (-0.6 to -0.46) |
| 261 | Germany | Deaths | Both | Colon and rectum cancer | Age-standardized | -0.986957937 | -1.102256384 | -0.871525071 | -0.99 (-1.1 to -0.87) |
| 262 | Germany | DALYs | Both | Colon and rectum cancer | Age-standardized | -0.930334149 | -1.041313574 | -0.819230263 | -0.93 (-1.04 to -0.82) |
| 263 | Germany | YLDs | Both | Colon and rectum cancer | Age-standardized | 0.579159463 | 0.409509948 | 0.749095613 | 0.58 (0.41 to 0.75) |
| 264 | Germany | YLLs | Both | Colon and rectum cancer | Age-standardized | -1.01473065 | -1.126207351 | -0.903128262 | -1.01 (-1.13 to -0.9) |
| 265 | Greece | Deaths | Both | Colon and rectum cancer | Age-standardized | -0.140213069 | -0.344220459 | 0.064211948 | -0.14 (-0.34 to 0.06) |
| 266 | Greece | DALYs | Both | Colon and rectum cancer | Age-standardized | 0.070375123 | -0.095137302 | 0.236161752 | 0.07 (-0.1 to 0.24) |
| 267 | Greece | YLDs | Both | Colon and rectum cancer | Age-standardized | 0.792264621 | 0.608378356 | 0.976486983 | 0.79 (0.61 to 0.98) |
| 268 | Greece | YLLs | Both | Colon and rectum cancer | Age-standardized | 0.027961511 | -0.139640674 | 0.195844993 | 0.03 (-0.14 to 0.2) |
| 269 | Iceland | Deaths | Both | Colon and rectum cancer | Age-standardized | 0.353638814 | 0.237635432 | 0.469776445 | 0.35 (0.24 to 0.47) |
| 270 | Iceland | DALYs | Both | Colon and rectum cancer | Age-standardized | 0.20253619 | 0.10652448 | 0.298639985 | 0.2 (0.11 to 0.3) |
| 271 | Iceland | YLDs | Both | Colon and rectum cancer | Age-standardized | 1.614000353 | 1.407791331 | 1.820628693 | 1.61 (1.41 to 1.82) |
| 272 | Iceland | YLLs | Both | Colon and rectum cancer | Age-standardized | 0.105735933 | 0.010865289 | 0.200696572 | 0.11 (0.01 to 0.2) |
| 273 | Ireland | Deaths | Both | Colon and rectum cancer | Age-standardized | -0.637766073 | -0.730932553 | -0.544512155 | -0.64 (-0.73 to -0.54) |
| 274 | Ireland | DALYs | Both | Colon and rectum cancer | Age-standardized | -0.7466711 | -0.828305448 | -0.664969554 | -0.75 (-0.83 to -0.66) |
| 275 | Ireland | YLDs | Both | Colon and rectum cancer | Age-standardized | 1.605737603 | 1.48882056 | 1.722789337 | 1.61 (1.49 to 1.72) |
| 276 | Ireland | YLLs | Both | Colon and rectum cancer | Age-standardized | -0.885265495 | -0.969996969 | -0.800461523 | -0.89 (-0.97 to -0.8) |
| 277 | Israel | Deaths | Both | Colon and rectum cancer | Age-standardized | -0.7686971 | -1.22004009 | -0.315291845 | -0.77 (-1.22 to -0.32) |
| 278 | Israel | DALYs | Both | Colon and rectum cancer | Age-standardized | -0.847384747 | -1.303907059 | -0.388750776 | -0.85 (-1.3 to -0.39) |
| 279 | Israel | YLDs | Both | Colon and rectum cancer | Age-standardized | 1.000805149 | 0.47294398 | 1.531439576 | 1 (0.47 to 1.53) |
| 280 | Israel | YLLs | Both | Colon and rectum cancer | Age-standardized | -0.937382556 | -1.392923514 | -0.479737109 | -0.94 (-1.39 to -0.48) |
| 281 | Italy | Deaths | Both | Colon and rectum cancer | Age-standardized | -0.30728988 | -0.473234732 | -0.141068341 | -0.31 (-0.47 to -0.14) |
| 282 | Italy | DALYs | Both | Colon and rectum cancer | Age-standardized | -0.376025261 | -0.570462061 | -0.181208234 | -0.38 (-0.57 to -0.18) |
| 283 | Italy | YLDs | Both | Colon and rectum cancer | Age-standardized | 1.349350451 | 0.946646337 | 1.753661064 | 1.35 (0.95 to 1.75) |
| 284 | Italy | YLLs | Both | Colon and rectum cancer | Age-standardized | -0.481362836 | -0.665963001 | -0.296419614 | -0.48 (-0.67 to -0.3) |
| 285 | Malta | Deaths | Both | Colon and rectum cancer | Age-standardized | 0.441775422 | 0.288321255 | 0.595464394 | 0.44 (0.29 to 0.6) |
| 286 | Malta | DALYs | Both | Colon and rectum cancer | Age-standardized | 0.381639169 | 0.257785811 | 0.505645529 | 0.38 (0.26 to 0.51) |
| 287 | Malta | YLDs | Both | Colon and rectum cancer | Age-standardized | 2.037406231 | 1.943907918 | 2.130990297 | 2.04 (1.94 to 2.13) |
| 288 | Malta | YLLs | Both | Colon and rectum cancer | Age-standardized | 0.295042236 | 0.169561053 | 0.420680608 | 0.3 (0.17 to 0.42) |
| 289 | Luxembourg | Deaths | Both | Colon and rectum cancer | Age-standardized | 0.743104568 | 0.626845393 | 0.859498063 | 0.74 (0.63 to 0.86) |
| 290 | Luxembourg | DALYs | Both | Colon and rectum cancer | Age-standardized | 0.528567413 | 0.401656929 | 0.655638315 | 0.53 (0.4 to 0.66) |
| 291 | Luxembourg | YLDs | Both | Colon and rectum cancer | Age-standardized | 2.71954062 | 2.448319945 | 2.991479323 | 2.72 (2.45 to 2.99) |
| 292 | Luxembourg | YLLs | Both | Colon and rectum cancer | Age-standardized | 0.407173533 | 0.284798306 | 0.529698092 | 0.41 (0.28 to 0.53) |
| 293 | Norway | Deaths | Both | Colon and rectum cancer | Age-standardized | -0.523148497 | -0.61317885 | -0.433036589 | -0.52 (-0.61 to -0.43) |
| 294 | Norway | DALYs | Both | Colon and rectum cancer | Age-standardized | -0.687091882 | -0.760063616 | -0.614066492 | -0.69 (-0.76 to -0.61) |
| 295 | Norway | YLDs | Both | Colon and rectum cancer | Age-standardized | 1.22631224 | 1.10550606 | 1.347262766 | 1.23 (1.11 to 1.35) |
| 296 | Norway | YLLs | Both | Colon and rectum cancer | Age-standardized | -0.797821883 | -0.87405009 | -0.721535055 | -0.8 (-0.87 to -0.72) |
| 297 | Netherlands | Deaths | Both | Colon and rectum cancer | Age-standardized | 0.341479504 | 0.183616691 | 0.499591066 | 0.34 (0.18 to 0.5) |
| 298 | Netherlands | DALYs | Both | Colon and rectum cancer | Age-standardized | 0.411164789 | 0.233103711 | 0.589542186 | 0.41 (0.23 to 0.59) |
| 299 | Netherlands | YLDs | Both | Colon and rectum cancer | Age-standardized | 0.88365524 | 0.654320607 | 1.113512399 | 0.88 (0.65 to 1.11) |
| 300 | Netherlands | YLLs | Both | Colon and rectum cancer | Age-standardized | 0.366345448 | 0.191862256 | 0.5411325 | 0.37 (0.19 to 0.54) |
| 301 | Portugal | Deaths | Both | Colon and rectum cancer | Age-standardized | 0.447014686 | 0.148217779 | 0.746703068 | 0.45 (0.15 to 0.75) |
| 302 | Portugal | DALYs | Both | Colon and rectum cancer | Age-standardized | 0.534723821 | 0.188939106 | 0.881701952 | 0.53 (0.19 to 0.88) |
| 303 | Portugal | YLDs | Both | Colon and rectum cancer | Age-standardized | 2.904364796 | 2.402190605 | 3.40900162 | 2.9 (2.4 to 3.41) |
| 304 | Portugal | YLLs | Both | Colon and rectum cancer | Age-standardized | 0.425248756 | 0.083246984 | 0.768419208 | 0.43 (0.08 to 0.77) |
| 305 | Spain | Deaths | Both | Colon and rectum cancer | Age-standardized | 0.261504843 | -0.013052705 | 0.536816307 | 0.26 (-0.01 to 0.54) |
| 306 | Spain | DALYs | Both | Colon and rectum cancer | Age-standardized | 0.215844665 | -0.06203466 | 0.49449664 | 0.22 (-0.06 to 0.49) |
| 307 | Spain | YLDs | Both | Colon and rectum cancer | Age-standardized | 1.993288021 | 1.628255882 | 2.359631295 | 1.99 (1.63 to 2.36) |
| 308 | Spain | YLLs | Both | Colon and rectum cancer | Age-standardized | 0.108487601 | -0.166966214 | 0.384701434 | 0.11 (-0.17 to 0.38) |
| 309 | Sweden | Deaths | Both | Colon and rectum cancer | Age-standardized | 0.592336055 | 0.427415764 | 0.757527176 | 0.59 (0.43 to 0.76) |
| 310 | Sweden | DALYs | Both | Colon and rectum cancer | Age-standardized | 0.47359005 | 0.294907431 | 0.652591005 | 0.47 (0.29 to 0.65) |
| 311 | Sweden | YLDs | Both | Colon and rectum cancer | Age-standardized | 1.748994883 | 1.463315599 | 2.035478523 | 1.75 (1.46 to 2.04) |
| 312 | Sweden | YLLs | Both | Colon and rectum cancer | Age-standardized | 0.400252552 | 0.226389711 | 0.574416993 | 0.4 (0.23 to 0.57) |
| 313 | Switzerland | Deaths | Both | Colon and rectum cancer | Age-standardized | -0.212793908 | -0.431703034 | 0.006596508 | -0.21 (-0.43 to 0.01) |
| 314 | Switzerland | DALYs | Both | Colon and rectum cancer | Age-standardized | -0.381564992 | -0.604525665 | -0.158104182 | -0.38 (-0.6 to -0.16) |
| 315 | Switzerland | YLDs | Both | Colon and rectum cancer | Age-standardized | 0.958984881 | 0.608132363 | 1.311060932 | 0.96 (0.61 to 1.31) |
| 316 | Switzerland | YLLs | Both | Colon and rectum cancer | Age-standardized | -0.4701132 | -0.687459287 | -0.25229145 | -0.47 (-0.69 to -0.25) |
| 317 | United Kingdom | Deaths | Both | Colon and rectum cancer | Age-standardized | 0.093980799 | 0.013667329 | 0.174358763 | 0.09 (0.01 to 0.17) |
| 318 | United Kingdom | DALYs | Both | Colon and rectum cancer | Age-standardized | -0.079057521 | -0.153204886 | -0.004855093 | -0.08 (-0.15 to 0) |
| 319 | United Kingdom | YLDs | Both | Colon and rectum cancer | Age-standardized | 1.662920524 | 1.51340838 | 1.812652875 | 1.66 (1.51 to 1.81) |
| 320 | United Kingdom | YLLs | Both | Colon and rectum cancer | Age-standardized | -0.173934702 | -0.25210264 | -0.095705507 | -0.17 (-0.25 to -0.1) |
| 321 | Argentina | Deaths | Both | Colon and rectum cancer | Age-standardized | 1.205876776 | 0.951933239 | 1.460459106 | 1.21 (0.95 to 1.46) |
| 322 | Argentina | DALYs | Both | Colon and rectum cancer | Age-standardized | 1.267890013 | 1.027508331 | 1.508843652 | 1.27 (1.03 to 1.51) |
| 323 | Argentina | YLDs | Both | Colon and rectum cancer | Age-standardized | 2.213181151 | 1.957285216 | 2.469719343 | 2.21 (1.96 to 2.47) |
| 324 | Argentina | YLLs | Both | Colon and rectum cancer | Age-standardized | 1.243278091 | 1.002917909 | 1.484210267 | 1.24 (1 to 1.48) |
| 325 | Chile | Deaths | Both | Colon and rectum cancer | Age-standardized | 2.268473417 | 2.090499387 | 2.446757709 | 2.27 (2.09 to 2.45) |
| 326 | Chile | DALYs | Both | Colon and rectum cancer | Age-standardized | 2.25861479 | 2.101915551 | 2.415554521 | 2.26 (2.1 to 2.42) |
| 327 | Chile | YLDs | Both | Colon and rectum cancer | Age-standardized | 4.179215806 | 3.988426684 | 4.370354973 | 4.18 (3.99 to 4.37) |
| 328 | Chile | YLLs | Both | Colon and rectum cancer | Age-standardized | 2.197367882 | 2.040798624 | 2.354177378 | 2.2 (2.04 to 2.35) |
| 329 | Uruguay | Deaths | Both | Colon and rectum cancer | Age-standardized | 2.110616167 | 2.029308121 | 2.191989007 | 2.11 (2.03 to 2.19) |
| 330 | Uruguay | DALYs | Both | Colon and rectum cancer | Age-standardized | 2.01035908 | 1.922589039 | 2.098204703 | 2.01 (1.92 to 2.1) |
| 331 | Uruguay | YLDs | Both | Colon and rectum cancer | Age-standardized | 3.102666061 | 2.985705906 | 3.219759047 | 3.1 (2.99 to 3.22) |
| 332 | Uruguay | YLLs | Both | Colon and rectum cancer | Age-standardized | 1.978463649 | 1.891129538 | 2.065872617 | 1.98 (1.89 to 2.07) |
| 333 | Canada | Deaths | Both | Colon and rectum cancer | Age-standardized | 0.995048693 | 0.894801983 | 1.095395006 | 1 (0.89 to 1.1) |
| 334 | Canada | DALYs | Both | Colon and rectum cancer | Age-standardized | 0.87623902 | 0.780600926 | 0.971967871 | 0.88 (0.78 to 0.97) |
| 335 | Canada | YLDs | Both | Colon and rectum cancer | Age-standardized | 2.216181517 | 2.025373571 | 2.407346312 | 2.22 (2.03 to 2.41) |
| 336 | Canada | YLLs | Both | Colon and rectum cancer | Age-standardized | 0.779088261 | 0.685570435 | 0.872692948 | 0.78 (0.69 to 0.87) |
| 337 | United States of America | Deaths | Both | Colon and rectum cancer | Age-standardized | -0.06528473 | -0.249693841 | 0.119465298 | -0.07 (-0.25 to 0.12) |
| 338 | United States of America | DALYs | Both | Colon and rectum cancer | Age-standardized | 0.043782413 | -0.108066899 | 0.195862556 | 0.04 (-0.11 to 0.2) |
| 339 | United States of America | YLDs | Both | Colon and rectum cancer | Age-standardized | 0.790404534 | 0.538248383 | 1.043193109 | 0.79 (0.54 to 1.04) |
| 340 | United States of America | YLLs | Both | Colon and rectum cancer | Age-standardized | -0.005707816 | -0.151882381 | 0.140680745 | -0.01 (-0.15 to 0.14) |
| 341 | Antigua and Barbuda | Deaths | Both | Colon and rectum cancer | Age-standardized | 1.349900094 | 1.149928251 | 1.550267278 | 1.35 (1.15 to 1.55) |
| 342 | Antigua and Barbuda | DALYs | Both | Colon and rectum cancer | Age-standardized | 1.349517063 | 1.165815686 | 1.533552014 | 1.35 (1.17 to 1.53) |
| 343 | Antigua and Barbuda | YLDs | Both | Colon and rectum cancer | Age-standardized | 2.371295018 | 2.178900171 | 2.564052131 | 2.37 (2.18 to 2.56) |
| 344 | Antigua and Barbuda | YLLs | Both | Colon and rectum cancer | Age-standardized | 1.310038659 | 1.126234456 | 1.494176939 | 1.31 (1.13 to 1.49) |
| 345 | Bahamas | Deaths | Both | Colon and rectum cancer | Age-standardized | 1.790229798 | 1.613262215 | 1.967505584 | 1.79 (1.61 to 1.97) |
| 346 | Bahamas | DALYs | Both | Colon and rectum cancer | Age-standardized | 1.723869496 | 1.571032701 | 1.876936269 | 1.72 (1.57 to 1.88) |
| 347 | Bahamas | YLDs | Both | Colon and rectum cancer | Age-standardized | 2.729043036 | 2.476618615 | 2.982089239 | 2.73 (2.48 to 2.98) |
| 348 | Bahamas | YLLs | Both | Colon and rectum cancer | Age-standardized | 1.692086469 | 1.541650283 | 1.842745529 | 1.69 (1.54 to 1.84) |
| 349 | Barbados | Deaths | Both | Colon and rectum cancer | Age-standardized | 1.386203853 | 1.117633964 | 1.655487068 | 1.39 (1.12 to 1.66) |
| 350 | Barbados | DALYs | Both | Colon and rectum cancer | Age-standardized | 1.442041243 | 1.198674351 | 1.685993394 | 1.44 (1.2 to 1.69) |
| 351 | Barbados | YLDs | Both | Colon and rectum cancer | Age-standardized | 2.555763273 | 2.252818189 | 2.859605895 | 2.56 (2.25 to 2.86) |
| 352 | Barbados | YLLs | Both | Colon and rectum cancer | Age-standardized | 1.397141748 | 1.155426966 | 1.639434118 | 1.4 (1.16 to 1.64) |
| 353 | Belize | Deaths | Both | Colon and rectum cancer | Age-standardized | 2.246547078 | 1.689167523 | 2.806981746 | 2.25 (1.69 to 2.81) |
| 354 | Belize | DALYs | Both | Colon and rectum cancer | Age-standardized | 2.418996833 | 1.87568475 | 2.965206448 | 2.42 (1.88 to 2.97) |
| 355 | Belize | YLDs | Both | Colon and rectum cancer | Age-standardized | 3.045124455 | 2.610763065 | 3.48132454 | 3.05 (2.61 to 3.48) |
| 356 | Belize | YLLs | Both | Colon and rectum cancer | Age-standardized | 2.400024083 | 1.853350885 | 2.949631416 | 2.4 (1.85 to 2.95) |
| 357 | Cuba | Deaths | Both | Colon and rectum cancer | Age-standardized | 1.309204421 | 1.169198555 | 1.449404038 | 1.31 (1.17 to 1.45) |
| 358 | Cuba | DALYs | Both | Colon and rectum cancer | Age-standardized | 1.444184906 | 1.283531862 | 1.605092773 | 1.44 (1.28 to 1.61) |
| 359 | Cuba | YLDs | Both | Colon and rectum cancer | Age-standardized | 2.841519091 | 2.724674703 | 2.958496385 | 2.84 (2.72 to 2.96) |
| 360 | Cuba | YLLs | Both | Colon and rectum cancer | Age-standardized | 1.374425857 | 1.211902718 | 1.537209971 | 1.37 (1.21 to 1.54) |
| 361 | Dominica | Deaths | Both | Colon and rectum cancer | Age-standardized | 1.352455097 | 1.26050362 | 1.444490072 | 1.35 (1.26 to 1.44) |
| 362 | Dominica | DALYs | Both | Colon and rectum cancer | Age-standardized | 1.506216381 | 1.416605792 | 1.595906149 | 1.51 (1.42 to 1.6) |
| 363 | Dominica | YLDs | Both | Colon and rectum cancer | Age-standardized | 1.855352326 | 1.72499682 | 1.985874876 | 1.86 (1.72 to 1.99) |
| 364 | Dominica | YLLs | Both | Colon and rectum cancer | Age-standardized | 1.496226863 | 1.407393913 | 1.585137631 | 1.5 (1.41 to 1.59) |
| 365 | Grenada | Deaths | Both | Colon and rectum cancer | Age-standardized | 1.790330826 | 1.511745346 | 2.069680847 | 1.79 (1.51 to 2.07) |
| 366 | Grenada | DALYs | Both | Colon and rectum cancer | Age-standardized | 1.732438918 | 1.535574322 | 1.929685209 | 1.73 (1.54 to 1.93) |
| 367 | Grenada | YLDs | Both | Colon and rectum cancer | Age-standardized | 2.492337272 | 2.237132445 | 2.748179143 | 2.49 (2.24 to 2.75) |
| 368 | Grenada | YLLs | Both | Colon and rectum cancer | Age-standardized | 1.709858587 | 1.513837683 | 1.906258002 | 1.71 (1.51 to 1.91) |
| 369 | Dominican Republic | Deaths | Both | Colon and rectum cancer | Age-standardized | 2.621270287 | 2.367343901 | 2.875826549 | 2.62 (2.37 to 2.88) |
| 370 | Dominican Republic | DALYs | Both | Colon and rectum cancer | Age-standardized | 2.976103446 | 2.773648651 | 3.17895706 | 2.98 (2.77 to 3.18) |
| 371 | Dominican Republic | YLDs | Both | Colon and rectum cancer | Age-standardized | 3.837304877 | 3.637543801 | 4.037450992 | 3.84 (3.64 to 4.04) |
| 372 | Dominican Republic | YLLs | Both | Colon and rectum cancer | Age-standardized | 2.953194215 | 2.749934343 | 3.156856176 | 2.95 (2.75 to 3.16) |
| 373 | Haiti | Deaths | Both | Colon and rectum cancer | Age-standardized | 0.521256932 | 0.436170769 | 0.606415177 | 0.52 (0.44 to 0.61) |
| 374 | Haiti | DALYs | Both | Colon and rectum cancer | Age-standardized | 0.531946612 | 0.442245231 | 0.621728101 | 0.53 (0.44 to 0.62) |
| 375 | Haiti | YLDs | Both | Colon and rectum cancer | Age-standardized | 0.936699848 | 0.858795124 | 1.014664747 | 0.94 (0.86 to 1.01) |
| 376 | Haiti | YLLs | Both | Colon and rectum cancer | Age-standardized | 0.524917458 | 0.434993187 | 0.614922242 | 0.52 (0.43 to 0.61) |
| 377 | Guyana | Deaths | Both | Colon and rectum cancer | Age-standardized | 1.454716979 | 1.181797435 | 1.728372675 | 1.45 (1.18 to 1.73) |
| 378 | Guyana | DALYs | Both | Colon and rectum cancer | Age-standardized | 1.588964636 | 1.32321609 | 1.855410183 | 1.59 (1.32 to 1.86) |
| 379 | Guyana | YLDs | Both | Colon and rectum cancer | Age-standardized | 2.021600334 | 1.758874887 | 2.285004098 | 2.02 (1.76 to 2.29) |
| 380 | Guyana | YLLs | Both | Colon and rectum cancer | Age-standardized | 1.579729291 | 1.313744341 | 1.846412548 | 1.58 (1.31 to 1.85) |
| 381 | Jamaica | Deaths | Both | Colon and rectum cancer | Age-standardized | 1.78501187 | 1.486462862 | 2.084439137 | 1.79 (1.49 to 2.08) |
| 382 | Jamaica | DALYs | Both | Colon and rectum cancer | Age-standardized | 2.006735274 | 1.663365246 | 2.351265042 | 2.01 (1.66 to 2.35) |
| 383 | Jamaica | YLDs | Both | Colon and rectum cancer | Age-standardized | 2.798696067 | 2.44671043 | 3.151891054 | 2.8 (2.45 to 3.15) |
| 384 | Jamaica | YLLs | Both | Colon and rectum cancer | Age-standardized | 1.977182252 | 1.633535112 | 2.321991345 | 1.98 (1.63 to 2.32) |
| 385 | Saint Lucia | Deaths | Both | Colon and rectum cancer | Age-standardized | -0.389735959 | -0.630854566 | -0.148032279 | -0.39 (-0.63 to -0.15) |
| 386 | Saint Lucia | DALYs | Both | Colon and rectum cancer | Age-standardized | -0.029821775 | -0.226777304 | 0.167522551 | -0.03 (-0.23 to 0.17) |
| 387 | Saint Lucia | YLDs | Both | Colon and rectum cancer | Age-standardized | 1.134317369 | 1.019688571 | 1.249076239 | 1.13 (1.02 to 1.25) |
| 388 | Saint Lucia | YLLs | Both | Colon and rectum cancer | Age-standardized | -0.066184981 | -0.265888064 | 0.133917978 | -0.07 (-0.27 to 0.13) |
| 389 | Saint Vincent and the Grenadines | Deaths | Both | Colon and rectum cancer | Age-standardized | 0.963041335 | 0.796595094 | 1.129762429 | 0.96 (0.8 to 1.13) |
| 390 | Saint Vincent and the Grenadines | DALYs | Both | Colon and rectum cancer | Age-standardized | 1.118058031 | 0.964278241 | 1.272072046 | 1.12 (0.96 to 1.27) |
| 391 | Saint Vincent and the Grenadines | YLDs | Both | Colon and rectum cancer | Age-standardized | 1.713186745 | 1.572957945 | 1.853609141 | 1.71 (1.57 to 1.85) |
| 392 | Saint Vincent and the Grenadines | YLLs | Both | Colon and rectum cancer | Age-standardized | 1.100958711 | 0.946626829 | 1.255526544 | 1.1 (0.95 to 1.26) |
| 393 | Suriname | Deaths | Both | Colon and rectum cancer | Age-standardized | 1.392282832 | 1.162899839 | 1.622185942 | 1.39 (1.16 to 1.62) |
| 394 | Suriname | DALYs | Both | Colon and rectum cancer | Age-standardized | 1.469210992 | 1.239664626 | 1.69927782 | 1.47 (1.24 to 1.7) |
| 395 | Suriname | YLDs | Both | Colon and rectum cancer | Age-standardized | 2.182657101 | 1.947541912 | 2.418314521 | 2.18 (1.95 to 2.42) |
| 396 | Suriname | YLLs | Both | Colon and rectum cancer | Age-standardized | 1.451695708 | 1.222195018 | 1.681716744 | 1.45 (1.22 to 1.68) |
| 397 | Trinidad and Tobago | Deaths | Both | Colon and rectum cancer | Age-standardized | 0.201784503 | 0.061528215 | 0.342237388 | 0.2 (0.06 to 0.34) |
| 398 | Trinidad and Tobago | DALYs | Both | Colon and rectum cancer | Age-standardized | 0.47262748 | 0.349641803 | 0.595763885 | 0.47 (0.35 to 0.6) |
| 399 | Trinidad and Tobago | YLDs | Both | Colon and rectum cancer | Age-standardized | 1.858358235 | 1.724683756 | 1.992208373 | 1.86 (1.72 to 1.99) |
| 400 | Trinidad and Tobago | YLLs | Both | Colon and rectum cancer | Age-standardized | 0.428814059 | 0.305722137 | 0.552057036 | 0.43 (0.31 to 0.55) |
| 401 | Bolivia (Plurinational State of) | Deaths | Both | Colon and rectum cancer | Age-standardized | 1.863137421 | 1.784342016 | 1.941993825 | 1.86 (1.78 to 1.94) |
| 402 | Bolivia (Plurinational State of) | DALYs | Both | Colon and rectum cancer | Age-standardized | 1.763841528 | 1.701691628 | 1.826029408 | 1.76 (1.7 to 1.83) |
| 403 | Bolivia (Plurinational State of) | YLDs | Both | Colon and rectum cancer | Age-standardized | 2.705840072 | 2.631608809 | 2.780125024 | 2.71 (2.63 to 2.78) |
| 404 | Bolivia (Plurinational State of) | YLLs | Both | Colon and rectum cancer | Age-standardized | 1.745750326 | 1.683528449 | 1.808010277 | 1.75 (1.68 to 1.81) |
| 405 | Ecuador | Deaths | Both | Colon and rectum cancer | Age-standardized | 2.992597349 | 2.598189726 | 3.388521152 | 2.99 (2.6 to 3.39) |
| 406 | Ecuador | DALYs | Both | Colon and rectum cancer | Age-standardized | 2.962001325 | 2.60325282 | 3.322004181 | 2.96 (2.6 to 3.32) |
| 407 | Ecuador | YLDs | Both | Colon and rectum cancer | Age-standardized | 4.065408205 | 3.709922784 | 4.422112119 | 4.07 (3.71 to 4.42) |
| 408 | Ecuador | YLLs | Both | Colon and rectum cancer | Age-standardized | 2.935096295 | 2.57585447 | 3.295596259 | 2.94 (2.58 to 3.3) |
| 409 | Peru | Deaths | Both | Colon and rectum cancer | Age-standardized | 2.31292287 | 2.148198807 | 2.477912568 | 2.31 (2.15 to 2.48) |
| 410 | Peru | DALYs | Both | Colon and rectum cancer | Age-standardized | 2.148823213 | 1.967651093 | 2.330317233 | 2.15 (1.97 to 2.33) |
| 411 | Peru | YLDs | Both | Colon and rectum cancer | Age-standardized | 4.214542054 | 4.05050411 | 4.378838607 | 4.21 (4.05 to 4.38) |
| 412 | Peru | YLLs | Both | Colon and rectum cancer | Age-standardized | 2.090527965 | 1.909190863 | 2.272187738 | 2.09 (1.91 to 2.27) |
| 413 | Colombia | Deaths | Both | Colon and rectum cancer | Age-standardized | 0.716212739 | 0.50163113 | 0.931252503 | 0.72 (0.5 to 0.93) |
| 414 | Colombia | DALYs | Both | Colon and rectum cancer | Age-standardized | 0.863816847 | 0.645485093 | 1.082622231 | 0.86 (0.65 to 1.08) |
| 415 | Colombia | YLDs | Both | Colon and rectum cancer | Age-standardized | 2.832968797 | 2.647714329 | 3.018557606 | 2.83 (2.65 to 3.02) |
| 416 | Colombia | YLLs | Both | Colon and rectum cancer | Age-standardized | 0.801639592 | 0.583320154 | 1.020432899 | 0.8 (0.58 to 1.02) |
| 417 | Costa Rica | Deaths | Both | Colon and rectum cancer | Age-standardized | 2.864402776 | 2.664090999 | 3.065105389 | 2.86 (2.66 to 3.07) |
| 418 | Costa Rica | DALYs | Both | Colon and rectum cancer | Age-standardized | 3.030564809 | 2.840116786 | 3.221365519 | 3.03 (2.84 to 3.22) |
| 419 | Costa Rica | YLDs | Both | Colon and rectum cancer | Age-standardized | 4.469504029 | 4.256935601 | 4.68250586 | 4.47 (4.26 to 4.68) |
| 420 | Costa Rica | YLLs | Both | Colon and rectum cancer | Age-standardized | 2.972530724 | 2.78278767 | 3.162624055 | 2.97 (2.78 to 3.16) |
| 421 | El Salvador | Deaths | Both | Colon and rectum cancer | Age-standardized | 3.287777872 | 3.038053315 | 3.538107666 | 3.29 (3.04 to 3.54) |
| 422 | El Salvador | DALYs | Both | Colon and rectum cancer | Age-standardized | 3.312302041 | 3.059577515 | 3.565646304 | 3.31 (3.06 to 3.57) |
| 423 | El Salvador | YLDs | Both | Colon and rectum cancer | Age-standardized | 5.063705042 | 4.666134836 | 5.462785403 | 5.06 (4.67 to 5.46) |
| 424 | El Salvador | YLLs | Both | Colon and rectum cancer | Age-standardized | 3.265321801 | 3.014948683 | 3.51630344 | 3.27 (3.01 to 3.52) |
| 425 | Guatemala | Deaths | Both | Colon and rectum cancer | Age-standardized | 2.619698822 | 2.085700594 | 3.156490332 | 2.62 (2.09 to 3.16) |
| 426 | Guatemala | DALYs | Both | Colon and rectum cancer | Age-standardized | 2.899721507 | 2.415807124 | 3.385922384 | 2.9 (2.42 to 3.39) |
| 427 | Guatemala | YLDs | Both | Colon and rectum cancer | Age-standardized | 3.88606582 | 3.34532874 | 4.429632216 | 3.89 (3.35 to 4.43) |
| 428 | Guatemala | YLLs | Both | Colon and rectum cancer | Age-standardized | 2.878886664 | 2.395860623 | 3.364191255 | 2.88 (2.4 to 3.36) |
| 429 | Honduras | Deaths | Both | Colon and rectum cancer | Age-standardized | 2.833806821 | 2.541095305 | 3.127353906 | 2.83 (2.54 to 3.13) |
| 430 | Honduras | DALYs | Both | Colon and rectum cancer | Age-standardized | 2.746285121 | 2.488917303 | 3.004299234 | 2.75 (2.49 to 3) |
| 431 | Honduras | YLDs | Both | Colon and rectum cancer | Age-standardized | 3.364048231 | 3.108551853 | 3.620177712 | 3.36 (3.11 to 3.62) |
| 432 | Honduras | YLLs | Both | Colon and rectum cancer | Age-standardized | 2.733351143 | 2.47581752 | 2.991531979 | 2.73 (2.48 to 2.99) |
| 433 | Mexico | Deaths | Both | Colon and rectum cancer | Age-standardized | 1.138432946 | 1.00184132 | 1.275209294 | 1.14 (1 to 1.28) |
| 434 | Mexico | DALYs | Both | Colon and rectum cancer | Age-standardized | 1.596559523 | 1.481060862 | 1.712189637 | 1.6 (1.48 to 1.71) |
| 435 | Mexico | YLDs | Both | Colon and rectum cancer | Age-standardized | 2.680161446 | 2.593893163 | 2.766502268 | 2.68 (2.59 to 2.77) |
| 436 | Mexico | YLLs | Both | Colon and rectum cancer | Age-standardized | 1.566902163 | 1.45016858 | 1.683770067 | 1.57 (1.45 to 1.68) |
| 437 | Panama | Deaths | Both | Colon and rectum cancer | Age-standardized | 2.435131094 | 2.341195496 | 2.529152912 | 2.44 (2.34 to 2.53) |
| 438 | Panama | DALYs | Both | Colon and rectum cancer | Age-standardized | 2.558317953 | 2.475006863 | 2.641696774 | 2.56 (2.48 to 2.64) |
| 439 | Panama | YLDs | Both | Colon and rectum cancer | Age-standardized | 4.026114999 | 3.918107249 | 4.134235007 | 4.03 (3.92 to 4.13) |
| 440 | Panama | YLLs | Both | Colon and rectum cancer | Age-standardized | 2.501995309 | 2.417199291 | 2.586861533 | 2.5 (2.42 to 2.59) |
| 441 | Nicaragua | Deaths | Both | Colon and rectum cancer | Age-standardized | 2.12325511 | 1.805128234 | 2.442376089 | 2.12 (1.81 to 2.44) |
| 442 | Nicaragua | DALYs | Both | Colon and rectum cancer | Age-standardized | 2.21183937 | 1.915633967 | 2.508905657 | 2.21 (1.92 to 2.51) |
| 443 | Nicaragua | YLDs | Both | Colon and rectum cancer | Age-standardized | 3.416038377 | 3.122696923 | 3.710214266 | 3.42 (3.12 to 3.71) |
| 444 | Nicaragua | YLLs | Both | Colon and rectum cancer | Age-standardized | 2.178934392 | 1.882164423 | 2.476568814 | 2.18 (1.88 to 2.48) |
| 445 | Venezuela (Bolivarian Republic of) | Deaths | Both | Colon and rectum cancer | Age-standardized | 1.575349886 | 1.431480707 | 1.719423128 | 1.58 (1.43 to 1.72) |
| 446 | Venezuela (Bolivarian Republic of) | DALYs | Both | Colon and rectum cancer | Age-standardized | 1.583565662 | 1.43542202 | 1.731925664 | 1.58 (1.44 to 1.73) |
| 447 | Venezuela (Bolivarian Republic of) | YLDs | Both | Colon and rectum cancer | Age-standardized | 2.865261042 | 2.588942706 | 3.142323628 | 2.87 (2.59 to 3.14) |
| 448 | Venezuela (Bolivarian Republic of) | YLLs | Both | Colon and rectum cancer | Age-standardized | 1.54616021 | 1.400076009 | 1.692454869 | 1.55 (1.4 to 1.69) |
| 449 | Brazil | Deaths | Both | Colon and rectum cancer | Age-standardized | 1.616258887 | 1.521126459 | 1.711480461 | 1.62 (1.52 to 1.71) |
| 450 | Brazil | DALYs | Both | Colon and rectum cancer | Age-standardized | 1.806527818 | 1.711198761 | 1.901946224 | 1.81 (1.71 to 1.9) |
| 451 | Brazil | YLDs | Both | Colon and rectum cancer | Age-standardized | 2.790790331 | 2.692576314 | 2.88909828 | 2.79 (2.69 to 2.89) |
| 452 | Brazil | YLLs | Both | Colon and rectum cancer | Age-standardized | 1.784353168 | 1.68873784 | 1.8800584 | 1.78 (1.69 to 1.88) |
| 453 | Paraguay | Deaths | Both | Colon and rectum cancer | Age-standardized | 3.697853929 | 3.487405294 | 3.908730525 | 3.7 (3.49 to 3.91) |
| 454 | Paraguay | DALYs | Both | Colon and rectum cancer | Age-standardized | 3.687158287 | 3.473201002 | 3.901557983 | 3.69 (3.47 to 3.9) |
| 455 | Paraguay | YLDs | Both | Colon and rectum cancer | Age-standardized | 4.349374418 | 4.149996632 | 4.54913388 | 4.35 (4.15 to 4.55) |
| 456 | Paraguay | YLLs | Both | Colon and rectum cancer | Age-standardized | 3.672083288 | 3.457424526 | 3.887187434 | 3.67 (3.46 to 3.89) |
| 457 | Algeria | Deaths | Both | Colon and rectum cancer | Age-standardized | 1.431499015 | 1.24849271 | 1.614836102 | 1.43 (1.25 to 1.61) |
| 458 | Algeria | DALYs | Both | Colon and rectum cancer | Age-standardized | 1.310794429 | 1.173885219 | 1.447888906 | 1.31 (1.17 to 1.45) |
| 459 | Algeria | YLDs | Both | Colon and rectum cancer | Age-standardized | 2.488899834 | 2.332697013 | 2.645341085 | 2.49 (2.33 to 2.65) |
| 460 | Algeria | YLLs | Both | Colon and rectum cancer | Age-standardized | 1.276661782 | 1.140303931 | 1.413203472 | 1.28 (1.14 to 1.41) |
| 461 | Bahrain | Deaths | Both | Colon and rectum cancer | Age-standardized | 0.034684316 | -0.28836745 | 0.358782724 | 0.03 (-0.29 to 0.36) |
| 462 | Bahrain | DALYs | Both | Colon and rectum cancer | Age-standardized | -0.169488152 | -0.440164822 | 0.101924416 | -0.17 (-0.44 to 0.1) |
| 463 | Bahrain | YLDs | Both | Colon and rectum cancer | Age-standardized | 1.849134836 | 1.595242618 | 2.103661545 | 1.85 (1.6 to 2.1) |
| 464 | Bahrain | YLLs | Both | Colon and rectum cancer | Age-standardized | -0.233915887 | -0.506702224 | 0.039618363 | -0.23 (-0.51 to 0.04) |
| 465 | Egypt | Deaths | Both | Colon and rectum cancer | Age-standardized | 5.501806322 | 5.075391533 | 5.929951579 | 5.5 (5.08 to 5.93) |
| 466 | Egypt | DALYs | Both | Colon and rectum cancer | Age-standardized | 5.222518321 | 4.859113036 | 5.587183043 | 5.22 (4.86 to 5.59) |
| 467 | Egypt | YLDs | Both | Colon and rectum cancer | Age-standardized | 6.197132967 | 5.873637783 | 6.521616585 | 6.2 (5.87 to 6.52) |
| 468 | Egypt | YLLs | Both | Colon and rectum cancer | Age-standardized | 5.199223496 | 4.8346073 | 5.565107832 | 5.2 (4.83 to 5.57) |
| 469 | Iran (Islamic Republic of) | Deaths | Both | Colon and rectum cancer | Age-standardized | 2.345990901 | 2.143800075 | 2.548581958 | 2.35 (2.14 to 2.55) |
| 470 | Iran (Islamic Republic of) | DALYs | Both | Colon and rectum cancer | Age-standardized | 2.276112121 | 2.066020345 | 2.486636349 | 2.28 (2.07 to 2.49) |
| 471 | Iran (Islamic Republic of) | YLDs | Both | Colon and rectum cancer | Age-standardized | 3.711607223 | 3.495195114 | 3.928471856 | 3.71 (3.5 to 3.93) |
| 472 | Iran (Islamic Republic of) | YLLs | Both | Colon and rectum cancer | Age-standardized | 2.228185252 | 2.018362364 | 2.438439685 | 2.23 (2.02 to 2.44) |
| 473 | Iraq | Deaths | Both | Colon and rectum cancer | Age-standardized | 2.229297489 | 1.932000041 | 2.527462043 | 2.23 (1.93 to 2.53) |
| 474 | Iraq | DALYs | Both | Colon and rectum cancer | Age-standardized | 1.853981313 | 1.594945563 | 2.113677525 | 1.85 (1.59 to 2.11) |
| 475 | Iraq | YLDs | Both | Colon and rectum cancer | Age-standardized | 3.362536136 | 2.987690961 | 3.738745638 | 3.36 (2.99 to 3.74) |
| 476 | Iraq | YLLs | Both | Colon and rectum cancer | Age-standardized | 1.815180591 | 1.559520642 | 2.071484124 | 1.82 (1.56 to 2.07) |
| 477 | Jordan | Deaths | Both | Colon and rectum cancer | Age-standardized | 0.522908921 | 0.253566111 | 0.792975352 | 0.52 (0.25 to 0.79) |
| 478 | Jordan | DALYs | Both | Colon and rectum cancer | Age-standardized | 0.270335975 | -0.026740713 | 0.568295446 | 0.27 (-0.03 to 0.57) |
| 479 | Jordan | YLDs | Both | Colon and rectum cancer | Age-standardized | 2.266550416 | 1.920174214 | 2.614103779 | 2.27 (1.92 to 2.61) |
| 480 | Jordan | YLLs | Both | Colon and rectum cancer | Age-standardized | 0.209151578 | -0.088104723 | 0.507292271 | 0.21 (-0.09 to 0.51) |
| 481 | Kuwait | Deaths | Both | Colon and rectum cancer | Age-standardized | 2.606962722 | 2.118272852 | 3.097991231 | 2.61 (2.12 to 3.1) |
| 482 | Kuwait | DALYs | Both | Colon and rectum cancer | Age-standardized | 2.467250418 | 1.958209982 | 2.978832309 | 2.47 (1.96 to 2.98) |
| 483 | Kuwait | YLDs | Both | Colon and rectum cancer | Age-standardized | 4.070705237 | 3.570296475 | 4.573531767 | 4.07 (3.57 to 4.57) |
| 484 | Kuwait | YLLs | Both | Colon and rectum cancer | Age-standardized | 2.396172662 | 1.88540267 | 2.909503237 | 2.4 (1.89 to 2.91) |
| 485 | Lebanon | Deaths | Both | Colon and rectum cancer | Age-standardized | 0.904763942 | 0.64753019 | 1.162655128 | 0.9 (0.65 to 1.16) |
| 486 | Lebanon | DALYs | Both | Colon and rectum cancer | Age-standardized | 0.822054931 | 0.580711945 | 1.063977018 | 0.82 (0.58 to 1.06) |
| 487 | Lebanon | YLDs | Both | Colon and rectum cancer | Age-standardized | 2.922856169 | 2.636906544 | 3.209602459 | 2.92 (2.64 to 3.21) |
| 488 | Lebanon | YLLs | Both | Colon and rectum cancer | Age-standardized | 0.749917271 | 0.50919254 | 0.99121855 | 0.75 (0.51 to 0.99) |
| 489 | Libya | Deaths | Both | Colon and rectum cancer | Age-standardized | 1.819044455 | 1.545063897 | 2.093764245 | 1.82 (1.55 to 2.09) |
| 490 | Libya | DALYs | Both | Colon and rectum cancer | Age-standardized | 1.85768395 | 1.586263895 | 2.129829189 | 1.86 (1.59 to 2.13) |
| 491 | Libya | YLDs | Both | Colon and rectum cancer | Age-standardized | 2.911245877 | 2.566143239 | 3.257509677 | 2.91 (2.57 to 3.26) |
| 492 | Libya | YLLs | Both | Colon and rectum cancer | Age-standardized | 1.826776293 | 1.556985076 | 2.097284225 | 1.83 (1.56 to 2.1) |
| 493 | Morocco | Deaths | Both | Colon and rectum cancer | Age-standardized | 3.465070915 | 3.284935696 | 3.645520302 | 3.47 (3.28 to 3.65) |
| 494 | Morocco | DALYs | Both | Colon and rectum cancer | Age-standardized | 3.33724466 | 3.165189313 | 3.509586955 | 3.34 (3.17 to 3.51) |
| 495 | Morocco | YLDs | Both | Colon and rectum cancer | Age-standardized | 4.33176187 | 4.129994605 | 4.53392009 | 4.33 (4.13 to 4.53) |
| 496 | Morocco | YLLs | Both | Colon and rectum cancer | Age-standardized | 3.31472325 | 3.143134025 | 3.486597932 | 3.31 (3.14 to 3.49) |
| 497 | Oman | Deaths | Both | Colon and rectum cancer | Age-standardized | 1.570733688 | 1.295271284 | 1.846945185 | 1.57 (1.3 to 1.85) |
| 498 | Oman | DALYs | Both | Colon and rectum cancer | Age-standardized | 1.279133313 | 0.992115262 | 1.566967066 | 1.28 (0.99 to 1.57) |
| 499 | Oman | YLDs | Both | Colon and rectum cancer | Age-standardized | 2.9444258 | 2.697054131 | 3.192393325 | 2.94 (2.7 to 3.19) |
| 500 | Oman | YLLs | Both | Colon and rectum cancer | Age-standardized | 1.224285195 | 0.934254646 | 1.515149135 | 1.22 (0.93 to 1.52) |
| 501 | Palestine | Deaths | Both | Colon and rectum cancer | Age-standardized | 0.359679482 | 0.174820674 | 0.544879421 | 0.36 (0.17 to 0.54) |
| 502 | Palestine | DALYs | Both | Colon and rectum cancer | Age-standardized | 0.369681637 | 0.221023506 | 0.518560273 | 0.37 (0.22 to 0.52) |
| 503 | Palestine | YLDs | Both | Colon and rectum cancer | Age-standardized | 1.597744622 | 1.484405625 | 1.711210198 | 1.6 (1.48 to 1.71) |
| 504 | Palestine | YLLs | Both | Colon and rectum cancer | Age-standardized | 0.336594849 | 0.185972063 | 0.487444087 | 0.34 (0.19 to 0.49) |
| 505 | Qatar | Deaths | Both | Colon and rectum cancer | Age-standardized | 0.633585117 | -0.061049766 | 1.333048123 | 0.63 (-0.06 to 1.33) |
| 506 | Qatar | DALYs | Both | Colon and rectum cancer | Age-standardized | 0.681390264 | 0.02861213 | 1.338428372 | 0.68 (0.03 to 1.34) |
| 507 | Qatar | YLDs | Both | Colon and rectum cancer | Age-standardized | 3.016010161 | 2.464164935 | 3.570827481 | 3.02 (2.46 to 3.57) |
| 508 | Qatar | YLLs | Both | Colon and rectum cancer | Age-standardized | 0.594486377 | -0.064398021 | 1.257714859 | 0.59 (-0.06 to 1.26) |
| 509 | Saudi Arabia | Deaths | Both | Colon and rectum cancer | Age-standardized | 1.998402533 | 1.641940328 | 2.356114866 | 2 (1.64 to 2.36) |
| 510 | Saudi Arabia | DALYs | Both | Colon and rectum cancer | Age-standardized | 2.203030781 | 1.859342317 | 2.5478789 | 2.2 (1.86 to 2.55) |
| 511 | Saudi Arabia | YLDs | Both | Colon and rectum cancer | Age-standardized | 4.133008905 | 3.911765468 | 4.354723403 | 4.13 (3.91 to 4.35) |
| 512 | Saudi Arabia | YLLs | Both | Colon and rectum cancer | Age-standardized | 2.141078922 | 1.791725197 | 2.491631645 | 2.14 (1.79 to 2.49) |
| 513 | Syrian Arab Republic | Deaths | Both | Colon and rectum cancer | Age-standardized | 1.373190255 | 1.213627461 | 1.5330046 | 1.37 (1.21 to 1.53) |
| 514 | Syrian Arab Republic | DALYs | Both | Colon and rectum cancer | Age-standardized | 1.144180469 | 0.98866746 | 1.299932953 | 1.14 (0.99 to 1.3) |
| 515 | Syrian Arab Republic | YLDs | Both | Colon and rectum cancer | Age-standardized | 2.707752985 | 2.534865335 | 2.880932146 | 2.71 (2.53 to 2.88) |
| 516 | Syrian Arab Republic | YLLs | Both | Colon and rectum cancer | Age-standardized | 1.09868468 | 0.942591399 | 1.255019336 | 1.1 (0.94 to 1.26) |
| 517 | Tunisia | Deaths | Both | Colon and rectum cancer | Age-standardized | 0.817205046 | 0.677247949 | 0.957356705 | 0.82 (0.68 to 0.96) |
| 518 | Tunisia | DALYs | Both | Colon and rectum cancer | Age-standardized | 0.988843237 | 0.842728824 | 1.135169359 | 0.99 (0.84 to 1.14) |
| 519 | Tunisia | YLDs | Both | Colon and rectum cancer | Age-standardized | 2.402896654 | 2.269754322 | 2.536212321 | 2.4 (2.27 to 2.54) |
| 520 | Tunisia | YLLs | Both | Colon and rectum cancer | Age-standardized | 0.943637416 | 0.796274574 | 1.0912157 | 0.94 (0.8 to 1.09) |
| 521 | Turkey | Deaths | Both | Colon and rectum cancer | Age-standardized | 1.359203954 | 0.911853125 | 1.808537928 | 1.36 (0.91 to 1.81) |
| 522 | Turkey | DALYs | Both | Colon and rectum cancer | Age-standardized | 1.158629108 | 0.733816806 | 1.585232919 | 1.16 (0.73 to 1.59) |
| 523 | Turkey | YLDs | Both | Colon and rectum cancer | Age-standardized | 3.565476606 | 3.211031603 | 3.921138837 | 3.57 (3.21 to 3.92) |
| 524 | Turkey | YLLs | Both | Colon and rectum cancer | Age-standardized | 1.088309571 | 0.662995337 | 1.515420814 | 1.09 (0.66 to 1.52) |
| 525 | United Arab Emirates | Deaths | Both | Colon and rectum cancer | Age-standardized | 2.050337685 | 1.430348462 | 2.67411657 | 2.05 (1.43 to 2.67) |
| 526 | United Arab Emirates | DALYs | Both | Colon and rectum cancer | Age-standardized | 1.241085154 | 0.710665205 | 1.774298703 | 1.24 (0.71 to 1.77) |
| 527 | United Arab Emirates | YLDs | Both | Colon and rectum cancer | Age-standardized | 2.496700594 | 2.010072478 | 2.985650117 | 2.5 (2.01 to 2.99) |
| 528 | United Arab Emirates | YLLs | Both | Colon and rectum cancer | Age-standardized | 1.207420877 | 0.675335474 | 1.742318436 | 1.21 (0.68 to 1.74) |
| 529 | Yemen | Deaths | Both | Colon and rectum cancer | Age-standardized | 1.830267857 | 1.695000461 | 1.965715176 | 1.83 (1.7 to 1.97) |
| 530 | Yemen | DALYs | Both | Colon and rectum cancer | Age-standardized | 1.615576981 | 1.493371627 | 1.73792948 | 1.62 (1.49 to 1.74) |
| 531 | Yemen | YLDs | Both | Colon and rectum cancer | Age-standardized | 2.391089984 | 2.226647842 | 2.555796649 | 2.39 (2.23 to 2.56) |
| 532 | Yemen | YLLs | Both | Colon and rectum cancer | Age-standardized | 1.600105728 | 1.478675996 | 1.721680764 | 1.6 (1.48 to 1.72) |
| 533 | Afghanistan | Deaths | Both | Colon and rectum cancer | Age-standardized | 1.850750622 | 1.722376993 | 1.979286259 | 1.85 (1.72 to 1.98) |
| 534 | Afghanistan | DALYs | Both | Colon and rectum cancer | Age-standardized | 1.824745359 | 1.685725569 | 1.963955209 | 1.82 (1.69 to 1.96) |
| 535 | Afghanistan | YLDs | Both | Colon and rectum cancer | Age-standardized | 2.360707335 | 2.158890211 | 2.562923154 | 2.36 (2.16 to 2.56) |
| 536 | Afghanistan | YLLs | Both | Colon and rectum cancer | Age-standardized | 1.815877527 | 1.677890114 | 1.954052203 | 1.82 (1.68 to 1.95) |
| 537 | Bangladesh | Deaths | Both | Colon and rectum cancer | Age-standardized | 0.709512397 | 0.553413552 | 0.86585357 | 0.71 (0.55 to 0.87) |
| 538 | Bangladesh | DALYs | Both | Colon and rectum cancer | Age-standardized | 0.670871464 | 0.559475302 | 0.782391028 | 0.67 (0.56 to 0.78) |
| 539 | Bangladesh | YLDs | Both | Colon and rectum cancer | Age-standardized | 1.776477176 | 1.669631777 | 1.883434859 | 1.78 (1.67 to 1.88) |
| 540 | Bangladesh | YLLs | Both | Colon and rectum cancer | Age-standardized | 0.650967269 | 0.539488041 | 0.762570105 | 0.65 (0.54 to 0.76) |
| 541 | Bhutan | Deaths | Both | Colon and rectum cancer | Age-standardized | 1.519597042 | 1.414671159 | 1.624631484 | 1.52 (1.41 to 1.62) |
| 542 | Bhutan | DALYs | Both | Colon and rectum cancer | Age-standardized | 1.132521434 | 1.04410406 | 1.221016176 | 1.13 (1.04 to 1.22) |
| 543 | Bhutan | YLDs | Both | Colon and rectum cancer | Age-standardized | 2.165434274 | 2.047382359 | 2.283622756 | 2.17 (2.05 to 2.28) |
| 544 | Bhutan | YLLs | Both | Colon and rectum cancer | Age-standardized | 1.11427616 | 1.026583184 | 1.202045256 | 1.11 (1.03 to 1.2) |
| 545 | India | Deaths | Both | Colon and rectum cancer | Age-standardized | 1.145421836 | 1.004750551 | 1.286289037 | 1.15 (1 to 1.29) |
| 546 | India | DALYs | Both | Colon and rectum cancer | Age-standardized | 1.048817464 | 0.904861072 | 1.192979232 | 1.05 (0.9 to 1.19) |
| 547 | India | YLDs | Both | Colon and rectum cancer | Age-standardized | 1.783292922 | 1.613377915 | 1.953492056 | 1.78 (1.61 to 1.95) |
| 548 | India | YLLs | Both | Colon and rectum cancer | Age-standardized | 1.035621 | 0.89211631 | 1.179329804 | 1.04 (0.89 to 1.18) |
| 549 | Nepal | Deaths | Both | Colon and rectum cancer | Age-standardized | 1.849336546 | 1.542205196 | 2.157396865 | 1.85 (1.54 to 2.16) |
| 550 | Nepal | DALYs | Both | Colon and rectum cancer | Age-standardized | 1.661450633 | 1.335784992 | 1.988162875 | 1.66 (1.34 to 1.99) |
| 551 | Nepal | YLDs | Both | Colon and rectum cancer | Age-standardized | 2.450958198 | 2.187891698 | 2.714701921 | 2.45 (2.19 to 2.71) |
| 552 | Nepal | YLLs | Both | Colon and rectum cancer | Age-standardized | 1.647955617 | 1.321345715 | 1.975618349 | 1.65 (1.32 to 1.98) |
| 553 | Pakistan | Deaths | Both | Colon and rectum cancer | Age-standardized | 2.170579129 | 1.905024375 | 2.436825893 | 2.17 (1.91 to 2.44) |
| 554 | Pakistan | DALYs | Both | Colon and rectum cancer | Age-standardized | 2.043167141 | 1.770417286 | 2.31664798 | 2.04 (1.77 to 2.32) |
| 555 | Pakistan | YLDs | Both | Colon and rectum cancer | Age-standardized | 2.357628421 | 2.145373647 | 2.570324253 | 2.36 (2.15 to 2.57) |
| 556 | Pakistan | YLLs | Both | Colon and rectum cancer | Age-standardized | 2.038096481 | 1.764365824 | 2.312563433 | 2.04 (1.76 to 2.31) |
| 557 | Angola | Deaths | Both | Colon and rectum cancer | Age-standardized | 1.123603966 | 1.022567739 | 1.224741243 | 1.12 (1.02 to 1.22) |
| 558 | Angola | DALYs | Both | Colon and rectum cancer | Age-standardized | 1.083204389 | 0.973359352 | 1.193168922 | 1.08 (0.97 to 1.19) |
| 559 | Angola | YLDs | Both | Colon and rectum cancer | Age-standardized | 1.499878397 | 1.34469751 | 1.655296899 | 1.5 (1.34 to 1.66) |
| 560 | Angola | YLLs | Both | Colon and rectum cancer | Age-standardized | 1.076514758 | 0.967390345 | 1.18575711 | 1.08 (0.97 to 1.19) |
| 561 | Central African Republic | Deaths | Both | Colon and rectum cancer | Age-standardized | 0.75942535 | 0.676692759 | 0.842225928 | 0.76 (0.68 to 0.84) |
| 562 | Central African Republic | DALYs | Both | Colon and rectum cancer | Age-standardized | 0.740564957 | 0.658341643 | 0.822855435 | 0.74 (0.66 to 0.82) |
| 563 | Central African Republic | YLDs | Both | Colon and rectum cancer | Age-standardized | 0.825634647 | 0.758574119 | 0.892739807 | 0.83 (0.76 to 0.89) |
| 564 | Central African Republic | YLLs | Both | Colon and rectum cancer | Age-standardized | 0.739356472 | 0.656897522 | 0.821882974 | 0.74 (0.66 to 0.82) |
| 565 | Congo | Deaths | Both | Colon and rectum cancer | Age-standardized | 0.975908394 | 0.901921643 | 1.049949397 | 0.98 (0.9 to 1.05) |
| 566 | Congo | DALYs | Both | Colon and rectum cancer | Age-standardized | 0.991416513 | 0.905278657 | 1.0776279 | 0.99 (0.91 to 1.08) |
| 567 | Congo | YLDs | Both | Colon and rectum cancer | Age-standardized | 1.44351265 | 1.337358268 | 1.549778232 | 1.44 (1.34 to 1.55) |
| 568 | Congo | YLLs | Both | Colon and rectum cancer | Age-standardized | 0.983982739 | 0.898075789 | 1.069962832 | 0.98 (0.9 to 1.07) |
| 569 | Equatorial Guinea | Deaths | Both | Colon and rectum cancer | Age-standardized | 3.156998735 | 2.961507752 | 3.352860893 | 3.16 (2.96 to 3.35) |
| 570 | Equatorial Guinea | DALYs | Both | Colon and rectum cancer | Age-standardized | 3.121701821 | 2.890191909 | 3.353732646 | 3.12 (2.89 to 3.35) |
| 571 | Equatorial Guinea | YLDs | Both | Colon and rectum cancer | Age-standardized | 4.240579322 | 3.968738783 | 4.513130626 | 4.24 (3.97 to 4.51) |
| 572 | Equatorial Guinea | YLLs | Both | Colon and rectum cancer | Age-standardized | 3.102711425 | 2.872010216 | 3.333930005 | 3.1 (2.87 to 3.33) |
| 573 | Democratic Republic of the Congo | Deaths | Both | Colon and rectum cancer | Age-standardized | 0.434832575 | 0.183192304 | 0.687104916 | 0.43 (0.18 to 0.69) |
| 574 | Democratic Republic of the Congo | DALYs | Both | Colon and rectum cancer | Age-standardized | 0.418845809 | 0.163007386 | 0.675337701 | 0.42 (0.16 to 0.68) |
| 575 | Democratic Republic of the Congo | YLDs | Both | Colon and rectum cancer | Age-standardized | 0.692638298 | 0.402941823 | 0.983170646 | 0.69 (0.4 to 0.98) |
| 576 | Democratic Republic of the Congo | YLLs | Both | Colon and rectum cancer | Age-standardized | 0.414493434 | 0.1591994 | 0.670438182 | 0.41 (0.16 to 0.67) |
| 577 | Gabon | Deaths | Both | Colon and rectum cancer | Age-standardized | 1.188767646 | 1.106700764 | 1.27090114 | 1.19 (1.11 to 1.27) |
| 578 | Gabon | DALYs | Both | Colon and rectum cancer | Age-standardized | 1.27922385 | 1.190337693 | 1.368188085 | 1.28 (1.19 to 1.37) |
| 579 | Gabon | YLDs | Both | Colon and rectum cancer | Age-standardized | 1.795197686 | 1.747986093 | 1.842431185 | 1.8 (1.75 to 1.84) |
| 580 | Gabon | YLLs | Both | Colon and rectum cancer | Age-standardized | 1.27042624 | 1.18046637 | 1.360466094 | 1.27 (1.18 to 1.36) |
| 581 | Burundi | Deaths | Both | Colon and rectum cancer | Age-standardized | 0.409570084 | 0.262047658 | 0.557309571 | 0.41 (0.26 to 0.56) |
| 582 | Burundi | DALYs | Both | Colon and rectum cancer | Age-standardized | 0.119003349 | -0.066386579 | 0.304737198 | 0.12 (-0.07 to 0.3) |
| 583 | Burundi | YLDs | Both | Colon and rectum cancer | Age-standardized | 0.50380314 | 0.336281512 | 0.671604462 | 0.5 (0.34 to 0.67) |
| 584 | Burundi | YLLs | Both | Colon and rectum cancer | Age-standardized | 0.112876708 | -0.072794055 | 0.298892458 | 0.11 (-0.07 to 0.3) |
| 585 | Comoros | Deaths | Both | Colon and rectum cancer | Age-standardized | 1.282381001 | 1.218878971 | 1.345922871 | 1.28 (1.22 to 1.35) |
| 586 | Comoros | DALYs | Both | Colon and rectum cancer | Age-standardized | 1.24062323 | 1.153311438 | 1.328010386 | 1.24 (1.15 to 1.33) |
| 587 | Comoros | YLDs | Both | Colon and rectum cancer | Age-standardized | 1.518537737 | 1.434449752 | 1.602695431 | 1.52 (1.43 to 1.6) |
| 588 | Comoros | YLLs | Both | Colon and rectum cancer | Age-standardized | 1.23591714 | 1.148461705 | 1.323448191 | 1.24 (1.15 to 1.32) |
| 589 | Djibouti | Deaths | Both | Colon and rectum cancer | Age-standardized | 2.647786383 | 2.578583396 | 2.717036056 | 2.65 (2.58 to 2.72) |
| 590 | Djibouti | DALYs | Both | Colon and rectum cancer | Age-standardized | 2.51817181 | 2.464005826 | 2.572366428 | 2.52 (2.46 to 2.57) |
| 591 | Djibouti | YLDs | Both | Colon and rectum cancer | Age-standardized | 2.79502568 | 2.708671687 | 2.881452277 | 2.8 (2.71 to 2.88) |
| 592 | Djibouti | YLLs | Both | Colon and rectum cancer | Age-standardized | 2.513485962 | 2.459128655 | 2.567872108 | 2.51 (2.46 to 2.57) |
| 593 | Eritrea | Deaths | Both | Colon and rectum cancer | Age-standardized | 2.33639845 | 2.251330223 | 2.421537449 | 2.34 (2.25 to 2.42) |
| 594 | Eritrea | DALYs | Both | Colon and rectum cancer | Age-standardized | 1.986972962 | 1.932670319 | 2.041304532 | 1.99 (1.93 to 2.04) |
| 595 | Eritrea | YLDs | Both | Colon and rectum cancer | Age-standardized | 2.274041754 | 2.235569695 | 2.312528291 | 2.27 (2.24 to 2.31) |
| 596 | Eritrea | YLLs | Both | Colon and rectum cancer | Age-standardized | 1.982510907 | 1.927869292 | 2.037181814 | 1.98 (1.93 to 2.04) |
| 597 | Ethiopia | Deaths | Both | Colon and rectum cancer | Age-standardized | -0.87947997 | -1.118857944 | -0.639522494 | -0.88 (-1.12 to -0.64) |
| 598 | Ethiopia | DALYs | Both | Colon and rectum cancer | Age-standardized | -1.376669426 | -1.65452433 | -1.0980295 | -1.38 (-1.65 to -1.1) |
| 599 | Ethiopia | YLDs | Both | Colon and rectum cancer | Age-standardized | -0.625383703 | -0.903321528 | -0.346666342 | -0.63 (-0.9 to -0.35) |
| 600 | Ethiopia | YLLs | Both | Colon and rectum cancer | Age-standardized | -1.388473148 | -1.666237691 | -1.109924001 | -1.39 (-1.67 to -1.11) |
| 601 | Kenya | Deaths | Both | Colon and rectum cancer | Age-standardized | 3.02856792 | 2.788617478 | 3.269078503 | 3.03 (2.79 to 3.27) |
| 602 | Kenya | DALYs | Both | Colon and rectum cancer | Age-standardized | 2.960869174 | 2.74749797 | 3.174683476 | 2.96 (2.75 to 3.17) |
| 603 | Kenya | YLDs | Both | Colon and rectum cancer | Age-standardized | 3.019925032 | 2.827954803 | 3.212253652 | 3.02 (2.83 to 3.21) |
| 604 | Kenya | YLLs | Both | Colon and rectum cancer | Age-standardized | 2.959798207 | 2.745587511 | 3.174455504 | 2.96 (2.75 to 3.17) |
| 605 | Madagascar | Deaths | Both | Colon and rectum cancer | Age-standardized | 1.256487809 | 1.114028075 | 1.399148255 | 1.26 (1.11 to 1.4) |
| 606 | Madagascar | DALYs | Both | Colon and rectum cancer | Age-standardized | 1.142374212 | 0.992935353 | 1.292034196 | 1.14 (0.99 to 1.29) |
| 607 | Madagascar | YLDs | Both | Colon and rectum cancer | Age-standardized | 1.362154262 | 1.199455475 | 1.52511462 | 1.36 (1.2 to 1.53) |
| 608 | Madagascar | YLLs | Both | Colon and rectum cancer | Age-standardized | 1.138737897 | 0.989520666 | 1.288175604 | 1.14 (0.99 to 1.29) |
| 609 | Malawi | Deaths | Both | Colon and rectum cancer | Age-standardized | 1.382404969 | 1.266626002 | 1.498316308 | 1.38 (1.27 to 1.5) |
| 610 | Malawi | DALYs | Both | Colon and rectum cancer | Age-standardized | 1.435445683 | 1.295502751 | 1.57558195 | 1.44 (1.3 to 1.58) |
| 611 | Malawi | YLDs | Both | Colon and rectum cancer | Age-standardized | 1.709765385 | 1.604627873 | 1.81501169 | 1.71 (1.6 to 1.82) |
| 612 | Malawi | YLLs | Both | Colon and rectum cancer | Age-standardized | 1.430792376 | 1.290071919 | 1.571708333 | 1.43 (1.29 to 1.57) |
| 613 | Mauritius | Deaths | Both | Colon and rectum cancer | Age-standardized | 1.692160506 | 1.495350873 | 1.889351773 | 1.69 (1.5 to 1.89) |
| 614 | Mauritius | DALYs | Both | Colon and rectum cancer | Age-standardized | 1.775687955 | 1.564741689 | 1.98707235 | 1.78 (1.56 to 1.99) |
| 615 | Mauritius | YLDs | Both | Colon and rectum cancer | Age-standardized | 2.481239698 | 2.256053377 | 2.706921919 | 2.48 (2.26 to 2.71) |
| 616 | Mauritius | YLLs | Both | Colon and rectum cancer | Age-standardized | 1.758147429 | 1.547212014 | 1.969521003 | 1.76 (1.55 to 1.97) |
| 617 | Mozambique | Deaths | Both | Colon and rectum cancer | Age-standardized | 2.477167007 | 2.283578038 | 2.671122377 | 2.48 (2.28 to 2.67) |
| 618 | Mozambique | DALYs | Both | Colon and rectum cancer | Age-standardized | 2.825759923 | 2.615993854 | 3.035954793 | 2.83 (2.62 to 3.04) |
| 619 | Mozambique | YLDs | Both | Colon and rectum cancer | Age-standardized | 2.740766222 | 2.542166752 | 2.939750331 | 2.74 (2.54 to 2.94) |
| 620 | Mozambique | YLLs | Both | Colon and rectum cancer | Age-standardized | 2.827228275 | 2.617126713 | 3.037760006 | 2.83 (2.62 to 3.04) |
| 621 | Rwanda | Deaths | Both | Colon and rectum cancer | Age-standardized | 0.220573425 | -0.055323291 | 0.497231753 | 0.22 (-0.06 to 0.5) |
| 622 | Rwanda | DALYs | Both | Colon and rectum cancer | Age-standardized | -0.130852593 | -0.461532054 | 0.200925426 | -0.13 (-0.46 to 0.2) |
| 623 | Rwanda | YLDs | Both | Colon and rectum cancer | Age-standardized | 0.567083219 | 0.264255252 | 0.870825816 | 0.57 (0.26 to 0.87) |
| 624 | Rwanda | YLLs | Both | Colon and rectum cancer | Age-standardized | -0.142222205 | -0.47333371 | 0.189990862 | -0.14 (-0.47 to 0.19) |
| 625 | Seychelles | Deaths | Both | Colon and rectum cancer | Age-standardized | 1.641239511 | 1.308953237 | 1.974615661 | 1.64 (1.31 to 1.97) |
| 626 | Seychelles | DALYs | Both | Colon and rectum cancer | Age-standardized | 1.545169626 | 1.207311653 | 1.884155463 | 1.55 (1.21 to 1.88) |
| 627 | Seychelles | YLDs | Both | Colon and rectum cancer | Age-standardized | 2.451071116 | 2.073369512 | 2.830170328 | 2.45 (2.07 to 2.83) |
| 628 | Seychelles | YLLs | Both | Colon and rectum cancer | Age-standardized | 1.524783079 | 1.187520122 | 1.86317015 | 1.52 (1.19 to 1.86) |
| 629 | Somalia | Deaths | Both | Colon and rectum cancer | Age-standardized | 1.467416258 | 1.37319025 | 1.561729848 | 1.47 (1.37 to 1.56) |
| 630 | Somalia | DALYs | Both | Colon and rectum cancer | Age-standardized | 1.307658428 | 1.226617687 | 1.38876405 | 1.31 (1.23 to 1.39) |
| 631 | Somalia | YLDs | Both | Colon and rectum cancer | Age-standardized | 1.440935625 | 1.355658902 | 1.526284097 | 1.44 (1.36 to 1.53) |
| 632 | Somalia | YLLs | Both | Colon and rectum cancer | Age-standardized | 1.30564791 | 1.224647017 | 1.386713621 | 1.31 (1.22 to 1.39) |
| 633 | United Republic of Tanzania | Deaths | Both | Colon and rectum cancer | Age-standardized | 1.958472138 | 1.874704883 | 2.042308271 | 1.96 (1.87 to 2.04) |
| 634 | United Republic of Tanzania | DALYs | Both | Colon and rectum cancer | Age-standardized | 1.989101044 | 1.895564071 | 2.082723881 | 1.99 (1.9 to 2.08) |
| 635 | United Republic of Tanzania | YLDs | Both | Colon and rectum cancer | Age-standardized | 2.252686806 | 2.145914503 | 2.359570717 | 2.25 (2.15 to 2.36) |
| 636 | United Republic of Tanzania | YLLs | Both | Colon and rectum cancer | Age-standardized | 1.984595022 | 1.891139551 | 2.078136211 | 1.98 (1.89 to 2.08) |
| 637 | Uganda | Deaths | Both | Colon and rectum cancer | Age-standardized | 0.817108735 | 0.619931501 | 1.014672363 | 0.82 (0.62 to 1.01) |
| 638 | Uganda | DALYs | Both | Colon and rectum cancer | Age-standardized | 0.917254453 | 0.691517969 | 1.143497008 | 0.92 (0.69 to 1.14) |
| 639 | Uganda | YLDs | Both | Colon and rectum cancer | Age-standardized | 1.240688329 | 1.074573358 | 1.407076309 | 1.24 (1.07 to 1.41) |
| 640 | Uganda | YLLs | Both | Colon and rectum cancer | Age-standardized | 0.911904738 | 0.685172083 | 1.139147973 | 0.91 (0.69 to 1.14) |
| 641 | Zambia | Deaths | Both | Colon and rectum cancer | Age-standardized | 2.057042808 | 1.979433738 | 2.134710941 | 2.06 (1.98 to 2.13) |
| 642 | Zambia | DALYs | Both | Colon and rectum cancer | Age-standardized | 2.08200179 | 1.992730538 | 2.171351178 | 2.08 (1.99 to 2.17) |
| 643 | Zambia | YLDs | Both | Colon and rectum cancer | Age-standardized | 2.386668478 | 2.272601747 | 2.50086243 | 2.39 (2.27 to 2.5) |
| 644 | Zambia | YLLs | Both | Colon and rectum cancer | Age-standardized | 2.077107514 | 1.987813169 | 2.166480041 | 2.08 (1.99 to 2.17) |
| 645 | Botswana | Deaths | Both | Colon and rectum cancer | Age-standardized | 1.553437543 | 1.299039519 | 1.808474451 | 1.55 (1.3 to 1.81) |
| 646 | Botswana | DALYs | Both | Colon and rectum cancer | Age-standardized | 1.62009647 | 1.343104566 | 1.89784545 | 1.62 (1.34 to 1.9) |
| 647 | Botswana | YLDs | Both | Colon and rectum cancer | Age-standardized | 1.924939132 | 1.724010969 | 2.126264173 | 1.92 (1.72 to 2.13) |
| 648 | Botswana | YLLs | Both | Colon and rectum cancer | Age-standardized | 1.614750791 | 1.33618224 | 1.894085115 | 1.61 (1.34 to 1.89) |
| 649 | Lesotho | Deaths | Both | Colon and rectum cancer | Age-standardized | 5.182877041 | 4.640976727 | 5.727583674 | 5.18 (4.64 to 5.73) |
| 650 | Lesotho | DALYs | Both | Colon and rectum cancer | Age-standardized | 5.586370681 | 5.022792586 | 6.152973074 | 5.59 (5.02 to 6.15) |
| 651 | Lesotho | YLDs | Both | Colon and rectum cancer | Age-standardized | 5.128287578 | 4.67051015 | 5.5880671 | 5.13 (4.67 to 5.59) |
| 652 | Lesotho | YLLs | Both | Colon and rectum cancer | Age-standardized | 5.593920588 | 5.028598167 | 6.16228589 | 5.59 (5.03 to 6.16) |
| 653 | Namibia | Deaths | Both | Colon and rectum cancer | Age-standardized | 1.710115608 | 1.503150383 | 1.917502836 | 1.71 (1.5 to 1.92) |
| 654 | Namibia | DALYs | Both | Colon and rectum cancer | Age-standardized | 1.757657138 | 1.530201404 | 1.985622435 | 1.76 (1.53 to 1.99) |
| 655 | Namibia | YLDs | Both | Colon and rectum cancer | Age-standardized | 2.211065032 | 2.078931065 | 2.343370037 | 2.21 (2.08 to 2.34) |
| 656 | Namibia | YLLs | Both | Colon and rectum cancer | Age-standardized | 1.749522038 | 1.520294961 | 1.979266698 | 1.75 (1.52 to 1.98) |
| 657 | South Africa | Deaths | Both | Colon and rectum cancer | Age-standardized | 2.305160814 | 2.008089128 | 2.603097643 | 2.31 (2.01 to 2.6) |
| 658 | South Africa | DALYs | Both | Colon and rectum cancer | Age-standardized | 2.64251914 | 2.329916496 | 2.956076738 | 2.64 (2.33 to 2.96) |
| 659 | South Africa | YLDs | Both | Colon and rectum cancer | Age-standardized | 2.951577262 | 2.73577894 | 3.167828872 | 2.95 (2.74 to 3.17) |
| 660 | South Africa | YLLs | Both | Colon and rectum cancer | Age-standardized | 2.6365394 | 2.3219333 | 2.952112809 | 2.64 (2.32 to 2.95) |
| 661 | Eswatini | Deaths | Both | Colon and rectum cancer | Age-standardized | 2.630453165 | 2.119490139 | 3.143972835 | 2.63 (2.12 to 3.14) |
| 662 | Eswatini | DALYs | Both | Colon and rectum cancer | Age-standardized | 2.910666943 | 2.3327438 | 3.491853902 | 2.91 (2.33 to 3.49) |
| 663 | Eswatini | YLDs | Both | Colon and rectum cancer | Age-standardized | 2.804433193 | 2.373074394 | 3.237609563 | 2.8 (2.37 to 3.24) |
| 664 | Eswatini | YLLs | Both | Colon and rectum cancer | Age-standardized | 2.912427249 | 2.332126484 | 3.496018758 | 2.91 (2.33 to 3.5) |
| 665 | Zimbabwe | Deaths | Both | Colon and rectum cancer | Age-standardized | 1.950359233 | 1.536352481 | 2.366054065 | 1.95 (1.54 to 2.37) |
| 666 | Zimbabwe | DALYs | Both | Colon and rectum cancer | Age-standardized | 2.37642759 | 1.897262435 | 2.857845987 | 2.38 (1.9 to 2.86) |
| 667 | Zimbabwe | YLDs | Both | Colon and rectum cancer | Age-standardized | 1.947629683 | 1.568621739 | 2.328051913 | 1.95 (1.57 to 2.33) |
| 668 | Zimbabwe | YLLs | Both | Colon and rectum cancer | Age-standardized | 2.383789553 | 1.902838397 | 2.867010656 | 2.38 (1.9 to 2.87) |
| 669 | Benin | Deaths | Both | Colon and rectum cancer | Age-standardized | 2.330461556 | 2.211605502 | 2.44945582 | 2.33 (2.21 to 2.45) |
| 670 | Benin | DALYs | Both | Colon and rectum cancer | Age-standardized | 2.269699913 | 2.160100304 | 2.379417103 | 2.27 (2.16 to 2.38) |
| 671 | Benin | YLDs | Both | Colon and rectum cancer | Age-standardized | 2.527319537 | 2.41672544 | 2.638033059 | 2.53 (2.42 to 2.64) |
| 672 | Benin | YLLs | Both | Colon and rectum cancer | Age-standardized | 2.265278089 | 2.15566913 | 2.375004655 | 2.27 (2.16 to 2.38) |
| 673 | Burkina Faso | Deaths | Both | Colon and rectum cancer | Age-standardized | 2.429446519 | 2.333171114 | 2.5258125 | 2.43 (2.33 to 2.53) |
| 674 | Burkina Faso | DALYs | Both | Colon and rectum cancer | Age-standardized | 2.402211874 | 2.303509383 | 2.501009594 | 2.4 (2.3 to 2.5) |
| 675 | Burkina Faso | YLDs | Both | Colon and rectum cancer | Age-standardized | 2.597634318 | 2.49847855 | 2.696886009 | 2.6 (2.5 to 2.7) |
| 676 | Burkina Faso | YLLs | Both | Colon and rectum cancer | Age-standardized | 2.398840589 | 2.300086408 | 2.497690101 | 2.4 (2.3 to 2.5) |
| 677 | Cameroon | Deaths | Both | Colon and rectum cancer | Age-standardized | 2.298308198 | 2.179749216 | 2.417004744 | 2.3 (2.18 to 2.42) |
| 678 | Cameroon | DALYs | Both | Colon and rectum cancer | Age-standardized | 2.296772557 | 2.18225388 | 2.411419579 | 2.3 (2.18 to 2.41) |
| 679 | Cameroon | YLDs | Both | Colon and rectum cancer | Age-standardized | 2.525070693 | 2.424808476 | 2.625431056 | 2.53 (2.42 to 2.63) |
| 680 | Cameroon | YLLs | Both | Colon and rectum cancer | Age-standardized | 2.292870876 | 2.177677231 | 2.408194389 | 2.29 (2.18 to 2.41) |
| 681 | Cabo Verde | Deaths | Both | Colon and rectum cancer | Age-standardized | 4.230182796 | 3.787703314 | 4.674548708 | 4.23 (3.79 to 4.67) |
| 682 | Cabo Verde | DALYs | Both | Colon and rectum cancer | Age-standardized | 4.108099217 | 3.672571417 | 4.545456666 | 4.11 (3.67 to 4.55) |
| 683 | Cabo Verde | YLDs | Both | Colon and rectum cancer | Age-standardized | 4.762714473 | 4.422790905 | 5.103744581 | 4.76 (4.42 to 5.1) |
| 684 | Cabo Verde | YLLs | Both | Colon and rectum cancer | Age-standardized | 4.094021232 | 3.656434303 | 4.53345544 | 4.09 (3.66 to 4.53) |
| 685 | Chad | Deaths | Both | Colon and rectum cancer | Age-standardized | 2.767357197 | 2.583740547 | 2.951302506 | 2.77 (2.58 to 2.95) |
| 686 | Chad | DALYs | Both | Colon and rectum cancer | Age-standardized | 2.718253594 | 2.535584368 | 2.901248249 | 2.72 (2.54 to 2.9) |
| 687 | Chad | YLDs | Both | Colon and rectum cancer | Age-standardized | 2.762411604 | 2.623832502 | 2.901177838 | 2.76 (2.62 to 2.9) |
| 688 | Chad | YLLs | Both | Colon and rectum cancer | Age-standardized | 2.717538544 | 2.534082329 | 2.901323004 | 2.72 (2.53 to 2.9) |
| 689 | Coted'Ivoire | Deaths | Both | Colon and rectum cancer | Age-standardized | 1.496938865 | 1.337124198 | 1.65700557 | 1.5 (1.34 to 1.66) |
| 690 | Coted'Ivoire | DALYs | Both | Colon and rectum cancer | Age-standardized | 1.478681786 | 1.330244328 | 1.627336689 | 1.48 (1.33 to 1.63) |
| 691 | Coted'Ivoire | YLDs | Both | Colon and rectum cancer | Age-standardized | 1.829073222 | 1.730523822 | 1.92771809 | 1.83 (1.73 to 1.93) |
| 692 | Coted'Ivoire | YLLs | Both | Colon and rectum cancer | Age-standardized | 1.472578232 | 1.323195891 | 1.622180809 | 1.47 (1.32 to 1.62) |
| 693 | Gambia | Deaths | Both | Colon and rectum cancer | Age-standardized | 2.18140892 | 2.10146877 | 2.261411659 | 2.18 (2.1 to 2.26) |
| 694 | Gambia | DALYs | Both | Colon and rectum cancer | Age-standardized | 2.131516594 | 2.021086512 | 2.242066209 | 2.13 (2.02 to 2.24) |
| 695 | Gambia | YLDs | Both | Colon and rectum cancer | Age-standardized | 2.34953436 | 2.240733098 | 2.458451405 | 2.35 (2.24 to 2.46) |
| 696 | Gambia | YLLs | Both | Colon and rectum cancer | Age-standardized | 2.127521692 | 2.016824325 | 2.238339177 | 2.13 (2.02 to 2.24) |
| 697 | Ghana | Deaths | Both | Colon and rectum cancer | Age-standardized | 3.105180915 | 2.914679074 | 3.296035388 | 3.11 (2.91 to 3.3) |
| 698 | Ghana | DALYs | Both | Colon and rectum cancer | Age-standardized | 3.189945446 | 3.001815053 | 3.378419454 | 3.19 (3 to 3.38) |
| 699 | Ghana | YLDs | Both | Colon and rectum cancer | Age-standardized | 3.416040004 | 3.273021126 | 3.559256943 | 3.42 (3.27 to 3.56) |
| 700 | Ghana | YLLs | Both | Colon and rectum cancer | Age-standardized | 3.185812113 | 2.996795604 | 3.3751755 | 3.19 (3 to 3.38) |
| 701 | Guinea | Deaths | Both | Colon and rectum cancer | Age-standardized | 1.796935525 | 1.62431914 | 1.969845111 | 1.8 (1.62 to 1.97) |
| 702 | Guinea | DALYs | Both | Colon and rectum cancer | Age-standardized | 1.83221203 | 1.689287029 | 1.975337912 | 1.83 (1.69 to 1.98) |
| 703 | Guinea | YLDs | Both | Colon and rectum cancer | Age-standardized | 1.966470234 | 1.842503167 | 2.0905882 | 1.97 (1.84 to 2.09) |
| 704 | Guinea | YLLs | Both | Colon and rectum cancer | Age-standardized | 1.829983811 | 1.686707809 | 1.973461688 | 1.83 (1.69 to 1.97) |
| 705 | Guinea-Bissau | Deaths | Both | Colon and rectum cancer | Age-standardized | 2.172844104 | 2.054773252 | 2.291051557 | 2.17 (2.05 to 2.29) |
| 706 | Guinea-Bissau | DALYs | Both | Colon and rectum cancer | Age-standardized | 1.972205741 | 1.864430295 | 2.080095217 | 1.97 (1.86 to 2.08) |
| 707 | Guinea-Bissau | YLDs | Both | Colon and rectum cancer | Age-standardized | 2.19427666 | 2.09724214 | 2.291403403 | 2.19 (2.1 to 2.29) |
| 708 | Guinea-Bissau | YLLs | Both | Colon and rectum cancer | Age-standardized | 1.968741079 | 1.860758658 | 2.076837972 | 1.97 (1.86 to 2.08) |
| 709 | Liberia | Deaths | Both | Colon and rectum cancer | Age-standardized | 2.006892849 | 1.756017961 | 2.258386258 | 2.01 (1.76 to 2.26) |
| 710 | Liberia | DALYs | Both | Colon and rectum cancer | Age-standardized | 2.082067619 | 1.812179856 | 2.352670811 | 2.08 (1.81 to 2.35) |
| 711 | Liberia | YLDs | Both | Colon and rectum cancer | Age-standardized | 2.518801063 | 2.251709532 | 2.786590264 | 2.52 (2.25 to 2.79) |
| 712 | Liberia | YLLs | Both | Colon and rectum cancer | Age-standardized | 2.074594948 | 1.804665675 | 2.345239924 | 2.07 (1.8 to 2.35) |
| 713 | Mali | Deaths | Both | Colon and rectum cancer | Age-standardized | 1.180846711 | 1.086256894 | 1.275525039 | 1.18 (1.09 to 1.28) |
| 714 | Mali | DALYs | Both | Colon and rectum cancer | Age-standardized | 1.045183237 | 0.951730052 | 1.138722935 | 1.05 (0.95 to 1.14) |
| 715 | Mali | YLDs | Both | Colon and rectum cancer | Age-standardized | 1.389687409 | 1.299752436 | 1.479702227 | 1.39 (1.3 to 1.48) |
| 716 | Mali | YLLs | Both | Colon and rectum cancer | Age-standardized | 1.039720298 | 0.946209187 | 1.133318033 | 1.04 (0.95 to 1.13) |
| 717 | Mauritania | Deaths | Both | Colon and rectum cancer | Age-standardized | 1.506475895 | 1.284958115 | 1.728478151 | 1.51 (1.28 to 1.73) |
| 718 | Mauritania | DALYs | Both | Colon and rectum cancer | Age-standardized | 1.414728058 | 1.199351584 | 1.630562904 | 1.41 (1.2 to 1.63) |
| 719 | Mauritania | YLDs | Both | Colon and rectum cancer | Age-standardized | 2.075779037 | 1.824918752 | 2.327257353 | 2.08 (1.82 to 2.33) |
| 720 | Mauritania | YLLs | Both | Colon and rectum cancer | Age-standardized | 1.402662956 | 1.188004296 | 1.61777699 | 1.4 (1.19 to 1.62) |
| 721 | Niger | Deaths | Both | Colon and rectum cancer | Age-standardized | 1.850862063 | 1.73372536 | 1.968133637 | 1.85 (1.73 to 1.97) |
| 722 | Niger | DALYs | Both | Colon and rectum cancer | Age-standardized | 1.596643306 | 1.493237099 | 1.700154867 | 1.6 (1.49 to 1.7) |
| 723 | Niger | YLDs | Both | Colon and rectum cancer | Age-standardized | 1.899000849 | 1.784001893 | 2.014129735 | 1.9 (1.78 to 2.01) |
| 724 | Niger | YLLs | Both | Colon and rectum cancer | Age-standardized | 1.591706236 | 1.488455244 | 1.695062272 | 1.59 (1.49 to 1.7) |
| 725 | Nigeria | Deaths | Both | Colon and rectum cancer | Age-standardized | 1.863566855 | 1.734554259 | 1.992743056 | 1.86 (1.73 to 1.99) |
| 726 | Nigeria | DALYs | Both | Colon and rectum cancer | Age-standardized | 1.721919866 | 1.61328675 | 1.83066912 | 1.72 (1.61 to 1.83) |
| 727 | Nigeria | YLDs | Both | Colon and rectum cancer | Age-standardized | 2.09215302 | 1.954829575 | 2.229661427 | 2.09 (1.95 to 2.23) |
| 728 | Nigeria | YLLs | Both | Colon and rectum cancer | Age-standardized | 1.715625322 | 1.607343702 | 1.824022336 | 1.72 (1.61 to 1.82) |
| 729 | Sao Tome and Principe | Deaths | Both | Colon and rectum cancer | Age-standardized | 3.14141031 | 3.046228227 | 3.236680312 | 3.14 (3.05 to 3.24) |
| 730 | Sao Tome and Principe | DALYs | Both | Colon and rectum cancer | Age-standardized | 3.066637073 | 2.995570979 | 3.137752203 | 3.07 (3 to 3.14) |
| 731 | Sao Tome and Principe | YLDs | Both | Colon and rectum cancer | Age-standardized | 3.589391823 | 3.487278606 | 3.691605796 | 3.59 (3.49 to 3.69) |
| 732 | Sao Tome and Principe | YLLs | Both | Colon and rectum cancer | Age-standardized | 3.05690113 | 2.985886015 | 3.127965215 | 3.06 (2.99 to 3.13) |
| 733 | Senegal | Deaths | Both | Colon and rectum cancer | Age-standardized | 2.013067196 | 1.923524145 | 2.102688914 | 2.01 (1.92 to 2.1) |
| 734 | Senegal | DALYs | Both | Colon and rectum cancer | Age-standardized | 1.948909283 | 1.847345246 | 2.050574602 | 1.95 (1.85 to 2.05) |
| 735 | Senegal | YLDs | Both | Colon and rectum cancer | Age-standardized | 2.19306215 | 2.106632514 | 2.279564946 | 2.19 (2.11 to 2.28) |
| 736 | Senegal | YLLs | Both | Colon and rectum cancer | Age-standardized | 1.94459903 | 1.842746721 | 2.0465532 | 1.94 (1.84 to 2.05) |
| 737 | Sierra Leone | Deaths | Both | Colon and rectum cancer | Age-standardized | 2.476664611 | 2.304269866 | 2.649349861 | 2.48 (2.3 to 2.65) |
| 738 | Sierra Leone | DALYs | Both | Colon and rectum cancer | Age-standardized | 2.454475073 | 2.282525624 | 2.626713589 | 2.45 (2.28 to 2.63) |
| 739 | Sierra Leone | YLDs | Both | Colon and rectum cancer | Age-standardized | 2.629360957 | 2.468769789 | 2.790203807 | 2.63 (2.47 to 2.79) |
| 740 | Sierra Leone | YLLs | Both | Colon and rectum cancer | Age-standardized | 2.451462477 | 2.279161271 | 2.624053945 | 2.45 (2.28 to 2.62) |
| 741 | Togo | Deaths | Both | Colon and rectum cancer | Age-standardized | 2.222027939 | 2.135352349 | 2.308777085 | 2.22 (2.14 to 2.31) |
| 742 | Togo | DALYs | Both | Colon and rectum cancer | Age-standardized | 2.201842031 | 2.118903726 | 2.284847697 | 2.2 (2.12 to 2.28) |
| 743 | Togo | YLDs | Both | Colon and rectum cancer | Age-standardized | 2.371678994 | 2.29781569 | 2.445595631 | 2.37 (2.3 to 2.45) |
| 744 | Togo | YLLs | Both | Colon and rectum cancer | Age-standardized | 2.198853143 | 2.115365794 | 2.282408749 | 2.2 (2.12 to 2.28) |
| 745 | American Samoa | Deaths | Both | Colon and rectum cancer | Age-standardized | 0.82428331 | 0.733201216 | 0.91544776 | 0.82 (0.73 to 0.92) |
| 746 | American Samoa | DALYs | Both | Colon and rectum cancer | Age-standardized | 0.916454008 | 0.834536834 | 0.99843773 | 0.92 (0.83 to 1) |
| 747 | American Samoa | YLDs | Both | Colon and rectum cancer | Age-standardized | 1.260131854 | 1.190426391 | 1.329885333 | 1.26 (1.19 to 1.33) |
| 748 | American Samoa | YLLs | Both | Colon and rectum cancer | Age-standardized | 0.9095409 | 0.827260209 | 0.991888735 | 0.91 (0.83 to 0.99) |
| 749 | Bermuda | Deaths | Both | Colon and rectum cancer | Age-standardized | -0.165639766 | -0.395113412 | 0.06436255 | -0.17 (-0.4 to 0.06) |
| 750 | Bermuda | DALYs | Both | Colon and rectum cancer | Age-standardized | -0.117558668 | -0.346226449 | 0.111633819 | -0.12 (-0.35 to 0.11) |
| 751 | Bermuda | YLDs | Both | Colon and rectum cancer | Age-standardized | 2.024510778 | 1.847614213 | 2.201714591 | 2.02 (1.85 to 2.2) |
| 752 | Bermuda | YLLs | Both | Colon and rectum cancer | Age-standardized | -0.254179347 | -0.494358356 | -0.013420613 | -0.25 (-0.49 to -0.01) |
| 753 | Cook Islands | Deaths | Both | Colon and rectum cancer | Age-standardized | -0.576947351 | -0.737537911 | -0.416096981 | -0.58 (-0.74 to -0.42) |
| 754 | Cook Islands | DALYs | Both | Colon and rectum cancer | Age-standardized | -0.498180368 | -0.66500623 | -0.331074335 | -0.5 (-0.67 to -0.33) |
| 755 | Cook Islands | YLDs | Both | Colon and rectum cancer | Age-standardized | 0.966701222 | 0.851220084 | 1.082314592 | 0.97 (0.85 to 1.08) |
| 756 | Cook Islands | YLLs | Both | Colon and rectum cancer | Age-standardized | -0.539191912 | -0.707099807 | -0.371000079 | -0.54 (-0.71 to -0.37) |
| 757 | Greenland | Deaths | Both | Colon and rectum cancer | Age-standardized | 0.912384069 | 0.790548116 | 1.034367297 | 0.91 (0.79 to 1.03) |
| 758 | Greenland | DALYs | Both | Colon and rectum cancer | Age-standardized | 0.798285604 | 0.714165484 | 0.882475985 | 0.8 (0.71 to 0.88) |
| 759 | Greenland | YLDs | Both | Colon and rectum cancer | Age-standardized | 2.19298793 | 2.077963532 | 2.30814194 | 2.19 (2.08 to 2.31) |
| 760 | Greenland | YLLs | Both | Colon and rectum cancer | Age-standardized | 0.762136473 | 0.679046666 | 0.845294854 | 0.76 (0.68 to 0.85) |
| 761 | Guam | Deaths | Both | Colon and rectum cancer | Age-standardized | 0.03374814 | -0.3615712 | 0.430635925 | 0.03 (-0.36 to 0.43) |
| 762 | Guam | DALYs | Both | Colon and rectum cancer | Age-standardized | 0.617180042 | 0.285594207 | 0.949862238 | 0.62 (0.29 to 0.95) |
| 763 | Guam | YLDs | Both | Colon and rectum cancer | Age-standardized | 1.189437007 | 0.946840233 | 1.432616792 | 1.19 (0.95 to 1.43) |
| 764 | Guam | YLLs | Both | Colon and rectum cancer | Age-standardized | 0.602205165 | 0.267232061 | 0.938297349 | 0.6 (0.27 to 0.94) |
| 765 | Monaco | Deaths | Both | Colon and rectum cancer | Age-standardized | 1.285914842 | 1.206127961 | 1.365764623 | 1.29 (1.21 to 1.37) |
| 766 | Monaco | DALYs | Both | Colon and rectum cancer | Age-standardized | 1.242254257 | 1.150412742 | 1.334179161 | 1.24 (1.15 to 1.33) |
| 767 | Monaco | YLDs | Both | Colon and rectum cancer | Age-standardized | 2.306877151 | 2.160206343 | 2.453758533 | 2.31 (2.16 to 2.45) |
| 768 | Monaco | YLLs | Both | Colon and rectum cancer | Age-standardized | 1.175018918 | 1.085551116 | 1.264565906 | 1.18 (1.09 to 1.26) |
| 769 | Nauru | Deaths | Both | Colon and rectum cancer | Age-standardized | 0.01846686 | -0.093430163 | 0.130489209 | 0.02 (-0.09 to 0.13) |
| 770 | Nauru | DALYs | Both | Colon and rectum cancer | Age-standardized | 0.017974207 | -0.102052145 | 0.13814477 | 0.02 (-0.1 to 0.14) |
| 771 | Nauru | YLDs | Both | Colon and rectum cancer | Age-standardized | 0.387385503 | 0.185004431 | 0.590175401 | 0.39 (0.19 to 0.59) |
| 772 | Nauru | YLLs | Both | Colon and rectum cancer | Age-standardized | 0.011699181 | -0.106999815 | 0.130539223 | 0.01 (-0.11 to 0.13) |
| 773 | Niue | Deaths | Both | Colon and rectum cancer | Age-standardized | 0.841751704 | 0.805772312 | 0.877743937 | 0.84 (0.81 to 0.88) |
| 774 | Niue | DALYs | Both | Colon and rectum cancer | Age-standardized | 0.825883934 | 0.781615748 | 0.870171564 | 0.83 (0.78 to 0.87) |
| 775 | Niue | YLDs | Both | Colon and rectum cancer | Age-standardized | 1.49883992 | 1.459674237 | 1.538020722 | 1.5 (1.46 to 1.54) |
| 776 | Niue | YLLs | Both | Colon and rectum cancer | Age-standardized | 0.811669225 | 0.767190131 | 0.856167952 | 0.81 (0.77 to 0.86) |
| 777 | Northern Mariana Islands | Deaths | Both | Colon and rectum cancer | Age-standardized | 0.40743347 | 0.221292411 | 0.593920249 | 0.41 (0.22 to 0.59) |
| 778 | Northern Mariana Islands | DALYs | Both | Colon and rectum cancer | Age-standardized | 0.312591854 | 0.109328463 | 0.516267955 | 0.31 (0.11 to 0.52) |
| 779 | Northern Mariana Islands | YLDs | Both | Colon and rectum cancer | Age-standardized | 0.734488933 | 0.588855328 | 0.880333388 | 0.73 (0.59 to 0.88) |
| 780 | Northern Mariana Islands | YLLs | Both | Colon and rectum cancer | Age-standardized | 0.301996483 | 0.096034369 | 0.508382394 | 0.3 (0.1 to 0.51) |
| 781 | Palau | Deaths | Both | Colon and rectum cancer | Age-standardized | 0.351957642 | 0.275292222 | 0.428681676 | 0.35 (0.28 to 0.43) |
| 782 | Palau | DALYs | Both | Colon and rectum cancer | Age-standardized | 0.117944635 | 0.055164953 | 0.180763709 | 0.12 (0.06 to 0.18) |
| 783 | Palau | YLDs | Both | Colon and rectum cancer | Age-standardized | 0.723913765 | 0.670558564 | 0.777297245 | 0.72 (0.67 to 0.78) |
| 784 | Palau | YLLs | Both | Colon and rectum cancer | Age-standardized | 0.104861553 | 0.041506572 | 0.168256657 | 0.1 (0.04 to 0.17) |
| 785 | Puerto Rico | Deaths | Both | Colon and rectum cancer | Age-standardized | 0.425817282 | 0.159713647 | 0.6926279 | 0.43 (0.16 to 0.69) |
| 786 | Puerto Rico | DALYs | Both | Colon and rectum cancer | Age-standardized | 0.782302805 | 0.529879546 | 1.03535988 | 0.78 (0.53 to 1.04) |
| 787 | Puerto Rico | YLDs | Both | Colon and rectum cancer | Age-standardized | 2.859020481 | 2.462775194 | 3.256798134 | 2.86 (2.46 to 3.26) |
| 788 | Puerto Rico | YLLs | Both | Colon and rectum cancer | Age-standardized | 0.665806542 | 0.418118045 | 0.91410598 | 0.67 (0.42 to 0.91) |
| 789 | Saint Kitts and Nevis | Deaths | Both | Colon and rectum cancer | Age-standardized | 1.279227618 | 1.10435498 | 1.45440272 | 1.28 (1.1 to 1.45) |
| 790 | Saint Kitts and Nevis | DALYs | Both | Colon and rectum cancer | Age-standardized | 1.260640106 | 1.097767739 | 1.423774867 | 1.26 (1.1 to 1.42) |
| 791 | Saint Kitts and Nevis | YLDs | Both | Colon and rectum cancer | Age-standardized | 2.903282242 | 2.682148544 | 3.124892168 | 2.9 (2.68 to 3.12) |
| 792 | Saint Kitts and Nevis | YLLs | Both | Colon and rectum cancer | Age-standardized | 1.21288755 | 1.050000045 | 1.37603762 | 1.21 (1.05 to 1.38) |
| 793 | San Marino | Deaths | Both | Colon and rectum cancer | Age-standardized | -0.402199399 | -0.75704866 | -0.046081354 | -0.4 (-0.76 to -0.05) |
| 794 | San Marino | DALYs | Both | Colon and rectum cancer | Age-standardized | -0.319612016 | -0.65382078 | 0.015721054 | -0.32 (-0.65 to 0.02) |
| 795 | San Marino | YLDs | Both | Colon and rectum cancer | Age-standardized | 0.667912096 | 0.295592121 | 1.041614207 | 0.67 (0.3 to 1.04) |
| 796 | San Marino | YLLs | Both | Colon and rectum cancer | Age-standardized | -0.385976064 | -0.719441874 | -0.051390201 | -0.39 (-0.72 to -0.05) |
| 797 | Tokelau | Deaths | Both | Colon and rectum cancer | Age-standardized | 0.378599639 | 0.344859862 | 0.41235076 | 0.38 (0.34 to 0.41) |
| 798 | Tokelau | DALYs | Both | Colon and rectum cancer | Age-standardized | 0.286384754 | 0.245724189 | 0.327061812 | 0.29 (0.25 to 0.33) |
| 799 | Tokelau | YLDs | Both | Colon and rectum cancer | Age-standardized | 1.130149015 | 1.055361621 | 1.204991757 | 1.13 (1.06 to 1.2) |
| 800 | Tokelau | YLLs | Both | Colon and rectum cancer | Age-standardized | 0.269736181 | 0.229848125 | 0.309640112 | 0.27 (0.23 to 0.31) |
| 801 | Tuvalu | Deaths | Both | Colon and rectum cancer | Age-standardized | 0.084284029 | 0.034484818 | 0.13410803 | 0.08 (0.03 to 0.13) |
| 802 | Tuvalu | DALYs | Both | Colon and rectum cancer | Age-standardized | -0.008634203 | -0.065091698 | 0.047855186 | -0.01 (-0.07 to 0.05) |
| 803 | Tuvalu | YLDs | Both | Colon and rectum cancer | Age-standardized | 0.620617954 | 0.554726328 | 0.686552756 | 0.62 (0.55 to 0.69) |
| 804 | Tuvalu | YLLs | Both | Colon and rectum cancer | Age-standardized | -0.01974979 | -0.076133084 | 0.036665319 | -0.02 (-0.08 to 0.04) |
| 805 | United States Virgin Islands | Deaths | Both | Colon and rectum cancer | Age-standardized | -0.572202808 | -0.827323801 | -0.316425518 | -0.57 (-0.83 to -0.32) |
| 806 | United States Virgin Islands | DALYs | Both | Colon and rectum cancer | Age-standardized | -0.350620253 | -0.589135945 | -0.111532293 | -0.35 (-0.59 to -0.11) |
| 807 | United States Virgin Islands | YLDs | Both | Colon and rectum cancer | Age-standardized | 0.496258363 | 0.21895827 | 0.77432573 | 0.5 (0.22 to 0.77) |
| 808 | United States Virgin Islands | YLLs | Both | Colon and rectum cancer | Age-standardized | -0.382382922 | -0.620114629 | -0.144082525 | -0.38 (-0.62 to -0.14) |
| 809 | South Sudan | Deaths | Both | Colon and rectum cancer | Age-standardized | 1.952675029 | 1.863519425 | 2.041908666 | 1.95 (1.86 to 2.04) |
| 810 | South Sudan | DALYs | Both | Colon and rectum cancer | Age-standardized | 1.895007774 | 1.762047356 | 2.028141915 | 1.9 (1.76 to 2.03) |
| 811 | South Sudan | YLDs | Both | Colon and rectum cancer | Age-standardized | 2.041744577 | 1.918553258 | 2.165084799 | 2.04 (1.92 to 2.17) |
| 812 | South Sudan | YLLs | Both | Colon and rectum cancer | Age-standardized | 1.892644041 | 1.759517189 | 2.025945056 | 1.89 (1.76 to 2.03) |
| 813 | Sudan | Deaths | Both | Colon and rectum cancer | Age-standardized | 1.876923488 | 1.827339403 | 1.926531718 | 1.88 (1.83 to 1.93) |
| 814 | Sudan | DALYs | Both | Colon and rectum cancer | Age-standardized | 1.868523625 | 1.816836101 | 1.920237388 | 1.87 (1.82 to 1.92) |
| 815 | Sudan | YLDs | Both | Colon and rectum cancer | Age-standardized | 2.780674955 | 2.704230373 | 2.857176435 | 2.78 (2.7 to 2.86) |
| 816 | Sudan | YLLs | Both | Colon and rectum cancer | Age-standardized | 1.849730162 | 1.797477211 | 1.902009934 | 1.85 (1.8 to 1.9) |
