# Supplementary figures and images for: Global, regional, and national burden of hyperglycemia-associated colorectal cancer, 1990-2021: a systemic analysis for the Global Burden of Disease study
Source: Front Oncol. 2025 Sep 25;15:1633508. doi: 10.3389/fonc.2025.1633508 (PMC12507591; doi:10.3389/fonc.2025.1633508)

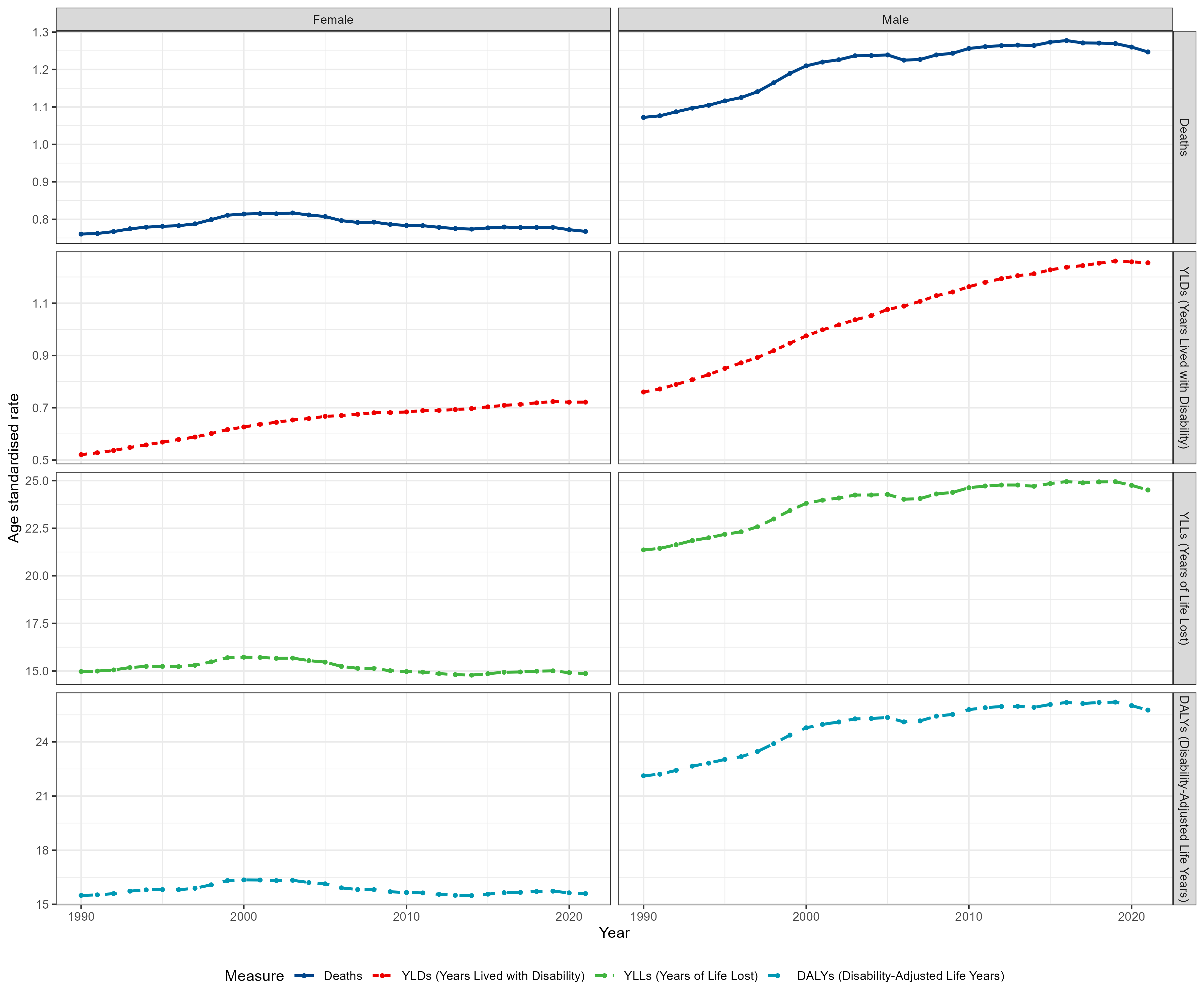

Supplement: Supplementary file 1 [file DataSheet1.zip › Fig.S1.tiff]

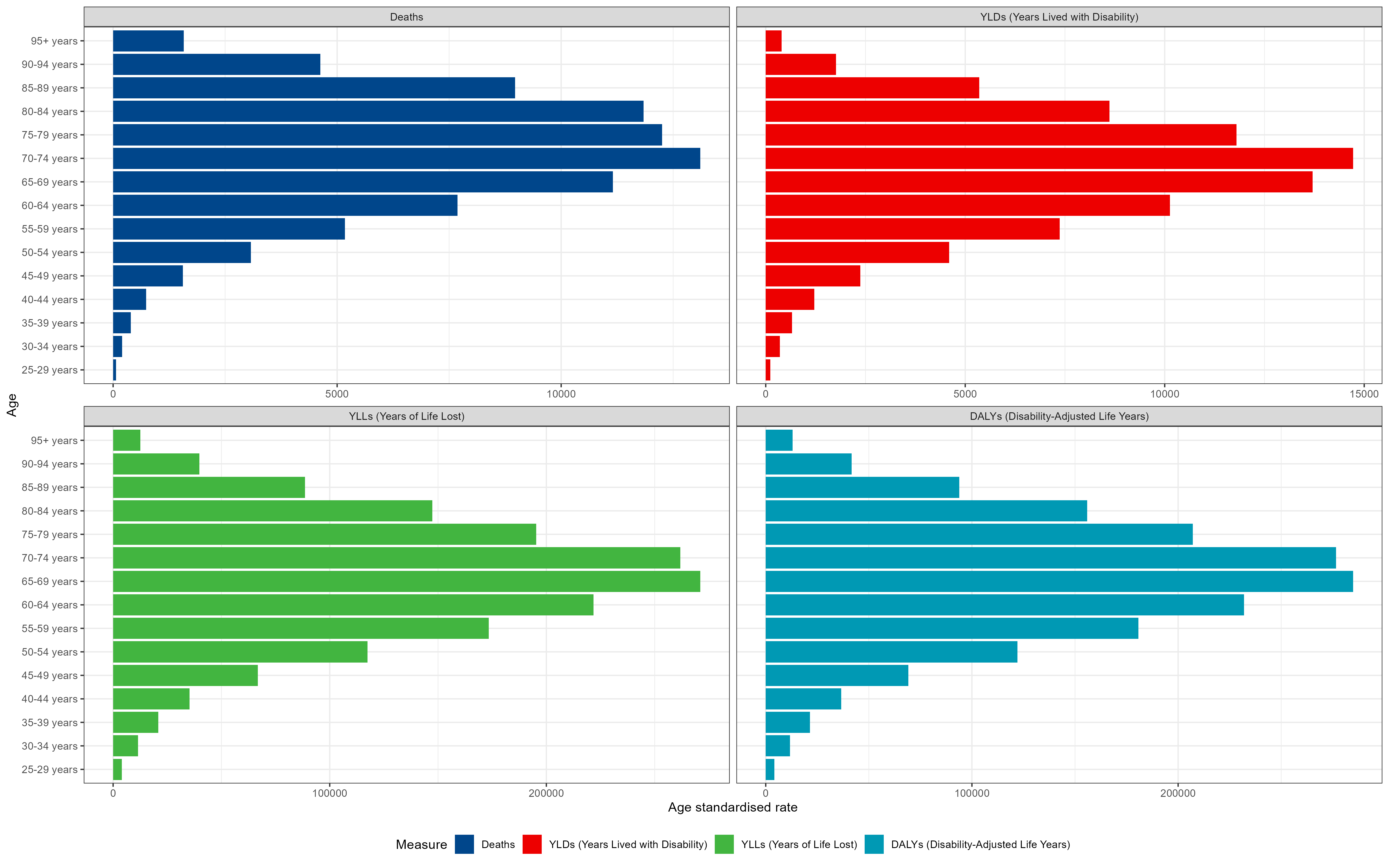

Supplement: Supplementary file 1 [file DataSheet1.zip › Fig.S2.tiff]

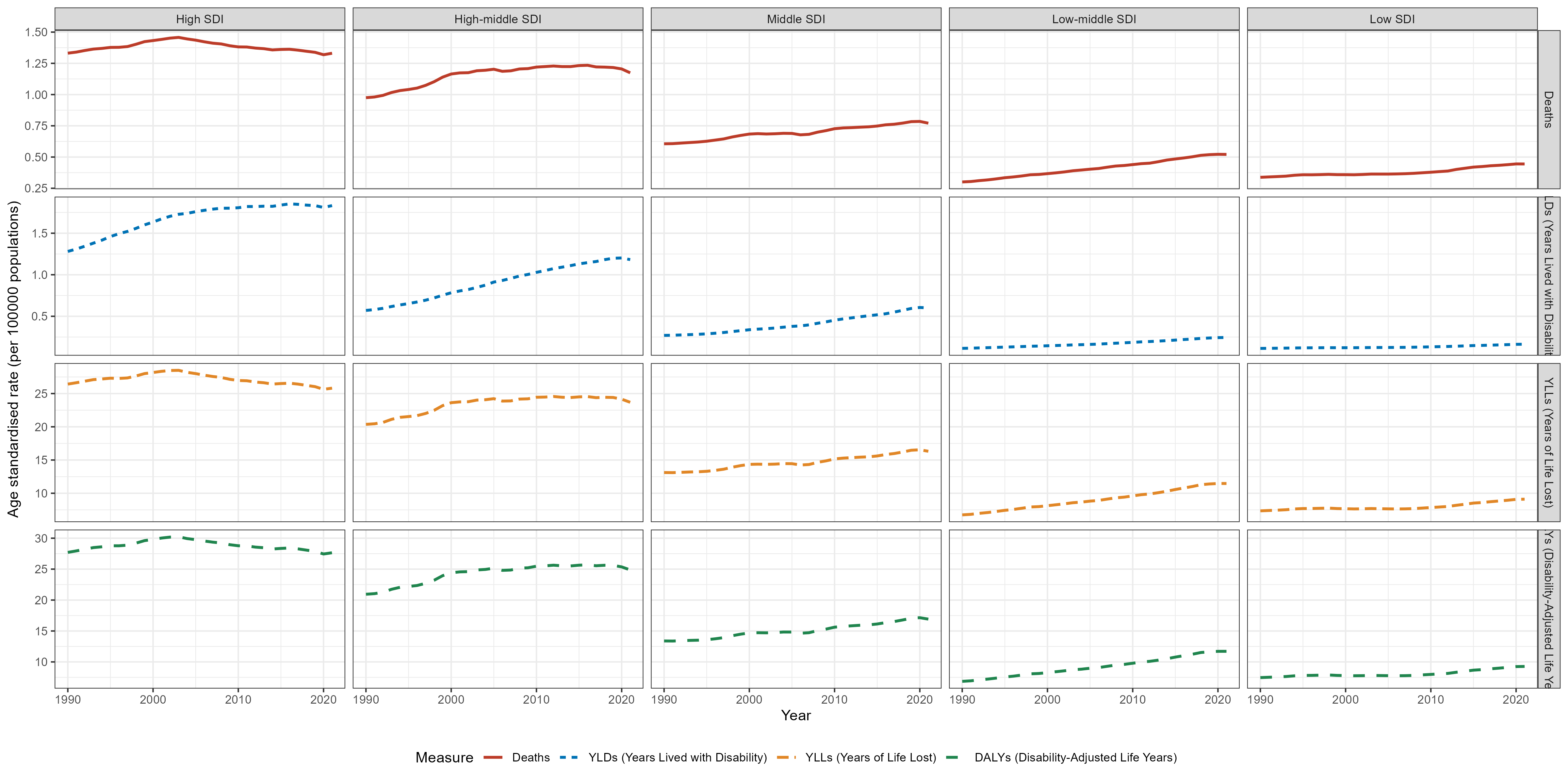

Supplement: Supplementary file 1 [file DataSheet1.zip › Fig.S3.tiff]
